# Synthesis of 2,3-(Diheterocyclyl)Propanoic Acid Esters as New Building Blocks via Complementary Meldrum’s Acid and Aza-Michael Strategies

**DOI:** 10.3390/molecules31162909

**Published:** 2026-08-20

**Authors:** Paulina Voznikaitė, Greta Račkauskienė, Miglė Dagilienė, Frank A. Sløk, Algirdas Šačkus

**Affiliations:** 1 Institute of Synthetic Chemistry, Kaunas University of Technology, K. Baršausko g. 59, LT-51423 Kaunas, Lithuania; paulina.voznikaite@ktu.lt (P.V.); migle.burinskaite@ktu.lt (M.D.); 2 Department of Organic Chemistry, Kaunas University of Technology, Radvilėnų pl. 19, LT-50254 Kaunas, Lithuania; 3 Vipergen ApS, Gammel Kongevej 23A, DK-1610 Copenhagen, Denmark; fas@vipergen.com

**Keywords:** propanoic acid derivatives, piperidine, azetidine, heteroaryl, *α*,*β*-unsaturated esters, aza-Michael addition, building blocks

## Abstract

In this study, we developed two complementary synthetic routes to novel piperidine- and azetidine-containing 2,3-disubstituted propanoic acid derivatives as heterocyclic amino acid building blocks. The strategy employs ketone- and carboxylic acid-derived Meldrum’s acid intermediates, which are converted into common *α*,*β*-unsaturated methyl esters through methanolysis; they are subsequently diversified via DBU-promoted aza-Michael addition with saturated cyclic amines and aromatic NH-heterocycles. The ketone-derived approach provided piperidine-containing derivatives in 35–89% yield and the corresponding azetidine analogues in 61–92% yield, whereas the complementary acid-derived route afforded regioisomeric products in 59–85% and 61–89% yield, respectively. Both synthetic sequences tolerated a broad range of nitrogen nucleophiles, and no alternative regioisomeric aza-Michael products were detected for heterocycles containing multiple nitrogen atoms. The structures of the synthesized compounds were established via ^1^H, ^13^C, ^15^N, and ^19^F NMR spectroscopy together with HRMS, including detailed multidimensional NMR analysis of representative products. The developed methodology provides efficient access to structurally diverse heterocyclic propanoic acid derivatives and expands the repertoire of amino acid building blocks available for peptide chemistry, medicinal chemistry, and DNA-encoded library synthesis.

## 1. Introduction

Substituted propanoic acid derivatives are an important and versatile class of compounds in organic synthesis and medicinal chemistry owing to the broad opportunities for structural diversification provided by functionalization at the C-2 and C-3 positions adjacent to the carboxyl group [1,2]. Moreover, substitution at C-2 often creates chiral centers that are important for the preparation of certain drug forms and serve as precursors for pharmaceuticals and natural products [3,4]. These applications have stimulated the development of efficient synthetic methodologies that expand the accessible structural diversity of the propanoic acid scaffold [5,6].

**Figure 1:**
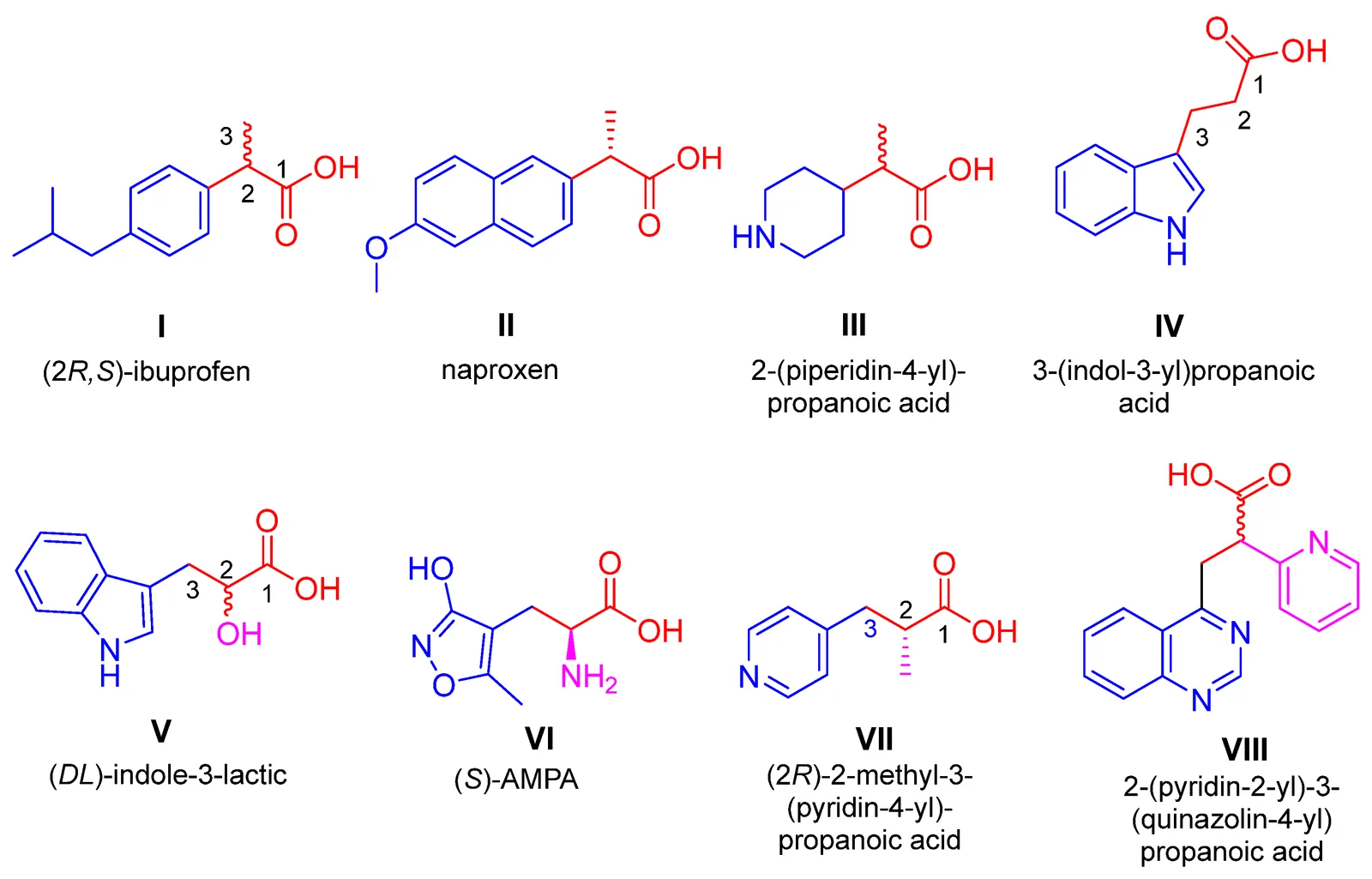
Examples of C-2-substituted, C-3-substituted, and C-2,3-disubstituted propanoic acid derivatives.

Monosubstituted propanoic acid derivatives comprise two principal structural classes depending on whether substitution occurs at C-2 or C-3. Among C-2-substituted derivatives, synthetic 2-arylpropionic acids are particularly important, as exemplified by the non-steroidal anti-inflammatory drugs ibuprofen **I** and naproxen **II** [7] (Figure 1). Because their biological activity strongly depends on stereochemistry, numerous asymmetric synthetic approaches have been developed, including nickel-catalyzed asymmetric hydrogenation of 2-substituted acrylic acids reported by Li et al. [8] and photoredox/nickel-catalyzed reductive alkyl–aryl coupling of acrylates described by Qian et al. [9]. Freifelder studied the reduction of 2-(2-, 3-, and 4-pyridyl)propionic acids to the corresponding 2-(piperidinyl)propionic acids, including compound **III** [10].

C-3-substituted propanoic acid has been extensively studied in medicinal chemistry. This propanoic acid structure is widespread in natural products and has been found to exhibit various biological activities. 3-Aminopropanoic acid, *β*-alanine, and its 3-substituted derivatives have also been widely used as building blocks to prepare oligomers [11,12], while *β*-peptide-based antibiotics are being explored as ways of evading antibiotic resistance [13]. Indole-3-propionic acid **IV** is a tryptophan-derived metabolite, which is widely reported for neuroprotection under various stress conditions such as neuronal injury [14]. 3-(4,5-Diphenyl-1,3-oxazol-2-yl)propanoic acid, the drug oxaprozin, is a non-steroidal anti-inflammatory agent (NSAID), used to relieve inflammation, swelling, stiffness, and joint pain associated with osteoarthritis and rheumatoid arthritis [15].

Among 2,3-disubstituted propanoic acid derivatives, compounds bearing hydroxyl and amino substituents at the C-2 position constitute two major structural classes represented by both naturally occurring and synthetic molecules. For example, naturally occurring *DL*-indole-3-lactic acid **V** is a versatile compound recognized for its probiotic functions in the intestine and its involvement in various biological processes and applications [16]. Additionally, *DL*-indole-3-lactic acid has garnered attention in the field of pharmaceuticals, where it is being investigated for its potential anti-inflammatory and antioxidant properties, as well as its role in immune regulation [17], making it a valuable candidate for therapeutic applications [18]. The essential proteinogenic amino acids histidine and tryptophan are exclusively heteroaromatic amino acids, highlighting their potential application in the development of bioactive compounds, including alkaloids [19]. However, naturally occurring 2-amino-3-substituted propanoic acids represent a vast class of non-proteinogenic amino acids, among which *L*-mimosine is a characteristic heterocyclic example [20,21]. Willardiine is a non-protein amino acid containing uracil and is thus classified as a nucleobase amino acid that acts as an agonist at the *α*-amino-3-hydroxy-5-methyl-4-isoxazole-propionic acid (AMPA) receptor **VI** [22]. Among synthetic heterocyclic 3-substituted amino acids are marked 2-amino-3-(2-, 3-, and 4-pyridyl)propanoic acids, where a pyridine ring replaces the phenyl ring of phenylalanine, widely used in medicinal chemistry and pharmacology [23,24,25,26]. Kolodiazhna et al. developed a method for synthesizing compounds with potential biological activity, such as enantiomerically pure 3-pyridine-2-methylpropanoic acids, including compound **VII**. The dynamic kinetic enzymatic resolution methodology was applied at key synthesis stages [27].

Biheterocyclic compounds are widely represented in both pharmaceutical [28] and agrochemical [29] fields. Their development involves combining two pharmacophore structures in a single molecule. The presence of two pharmacophores in a single unit results in pharmacological activity exceeding the sum of the activities of each individual part [30]. However, 2,3-diheterocyclic propanoic acids have been rarely described in the literature. Recently, Aksenov et al. reported the synthesis of 2-(pyridin-2-yl)-3-(quinazolin-4-yl)propionic acid **VIII** via an acid-catalyzed rearrangement of 4-oxobutyronitriles [31].

In general, heterocyclic amino acids have been applied as scaffolds, heterocyclic hybrids, and building blocks for the preparation of various biologically active compounds [32], peptides [33,34], and DNA-encoded chemical libraries [35,36]. However, the DNA-encoded library of target component molecules should have a high degree of structural and functional diversity, allowing for diversity-oriented synthesis (DOS) [37,38]. Previously, we have reported efficient protocols for synthesizing highly functionalized amino acid building blocks by including bicyclic heterocyclic compounds [39,40,41,42,43].

In this study, we developed a modular synthetic approach to novel 2,3-disubstituted propanoic acid derivatives, containing heterocycles and heteroaryls, as amino acid building blocks. This strategy employs two complementary synthetic routes originating from ketone- and acid-derived Meldrum’s acid intermediates that converge on common *α*,*β*-unsaturated intermediates, which are subsequently diversified through aza-Michael addition with various nitrogen heterocycles. By combining complementary synthetic entry points with late-stage diversification from common *α*,*β*-unsaturated intermediates, this approach provides efficient access to structurally diverse racemic amino acid building blocks while expanding the repertoire of propanoic acid derivatives incorporating both saturated cyclic amines and aromatic NH-heterocycles.

## 2. Results and Discussion

**Scheme 1:**
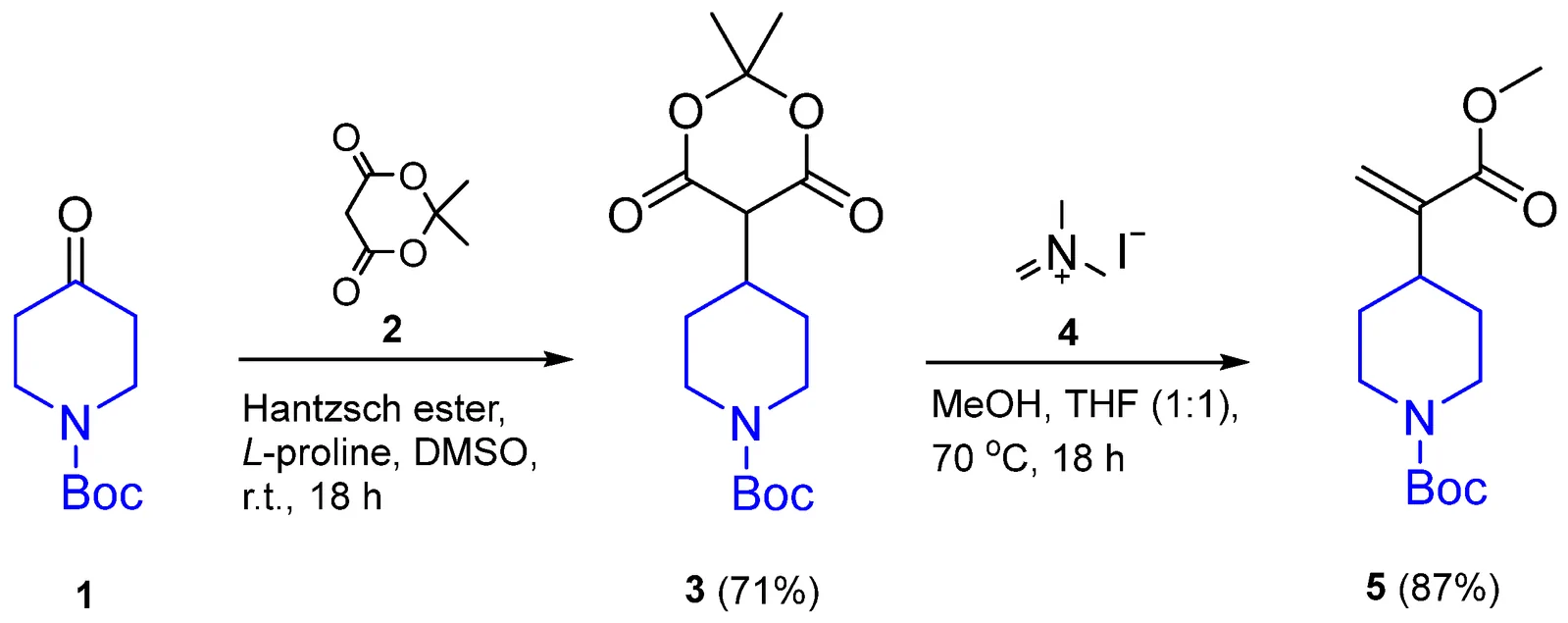
Preparation of piperidine-derived Michael acceptor **5** from *N*-Boc-4-piperidone **1**.

To access methyl 2-(*N*-Boc-piperidin-4-yl)-3-(*N*-heterocyclyl/heteroaryl)propionates, the key *α*,*β*-unsaturated ester **5** was first prepared from commercially available 1-(tert-butoxycarbonyl)-4-piperidone **1**, as outlined in Scheme 1. The synthesis commenced with an organocatalytic transformation adapted from literature procedures describing the reaction of carbonyl compounds with Meldrum’s acid in the presence of Hantzsch ester as a mild hydride donor for transfer hydrogenation [44,45,46,47]. Accordingly, ketone **1** was reacted with Meldrum’s acid **2** and Hantzsch ester in DMSO at room temperature using *L*-proline as the organocatalyst. Under the optimized conditions, the reaction reached completion within 18 h, affording Meldrum’s acid derivative **3** in 71% isolated yield after precipitation from methanol.

To optimize this transformation, several solvents of different polarity were evaluated (Table 1). Among the solvents examined, DMSO proved to be the most suitable, providing the highest isolated yield of **3**, whereas THF afforded the product in 59% yield. In the remaining solvents, incomplete conversion of ketone **1**, accompanied by the formation of side products, resulted in lower isolated yields. Therefore, DMSO was selected for all subsequent experiments. After removal of DMSO, addition of methanol induced precipitation of compound **3**, which was isolated by filtration and used directly in the subsequent step without further purification.

Then, Meldrum’s acid derivative **3** was converted into methyl *α*,*β*-unsaturated ester **5** via treatment with 2 equiv. *N*,*N*-dimethylmethyleneiminium iodide **4** (Eschenmoser’s salt) in a THF/MeOH mixture (1:1) at 70 °C for 18 h. Under these conditions, compound **5** was obtained in 87% yield after aqueous work-up and purification via flash column chromatography. This transformation involves methylenation of the Meldrum’s acid derivative followed by methanolysis, resulting in the formation of the corresponding methyl acrylate derivative **5**. It should be noted that to improve the efficiency of this methylenation/methanolysis step, the amount of Eschenmoser’s salt was subsequently optimized. For instance, a related Mannich-type cycloelimination of Meldrum’s acid derivatives with Eschenmoser’s salt has been reported for the preparation of methyl acrylate intermediates [48]. Following this literature procedure, Meldrum’s acid derivative **3** was initially treated with 3.5 equiv. *N*,*N*-dimethylmethyleneiminium iodide **4** in methanol at 70 °C for 18 h. Under these conditions, the desired *α*,*β*-unsaturated ester **5** was formed via methylenation followed by methanolysis. Lowering the reagent loading from 3.5 to 2.0 equiv. had only a minor effect on the isolated yield of compound **5**. In contrast, further reduction to 1.5 or 1.0 equiv. resulted in incomplete conversion of compound **3** and afforded product **5** in less than 50% yield. Therefore, 2.0 equiv. of Eschenmoser’s salt was selected as the optimal loading and was applied to the synthesis of compound **5**, which has been proven to be sufficiently stable and pure for further aza-Michael addition reactions.

**Scheme 2:**
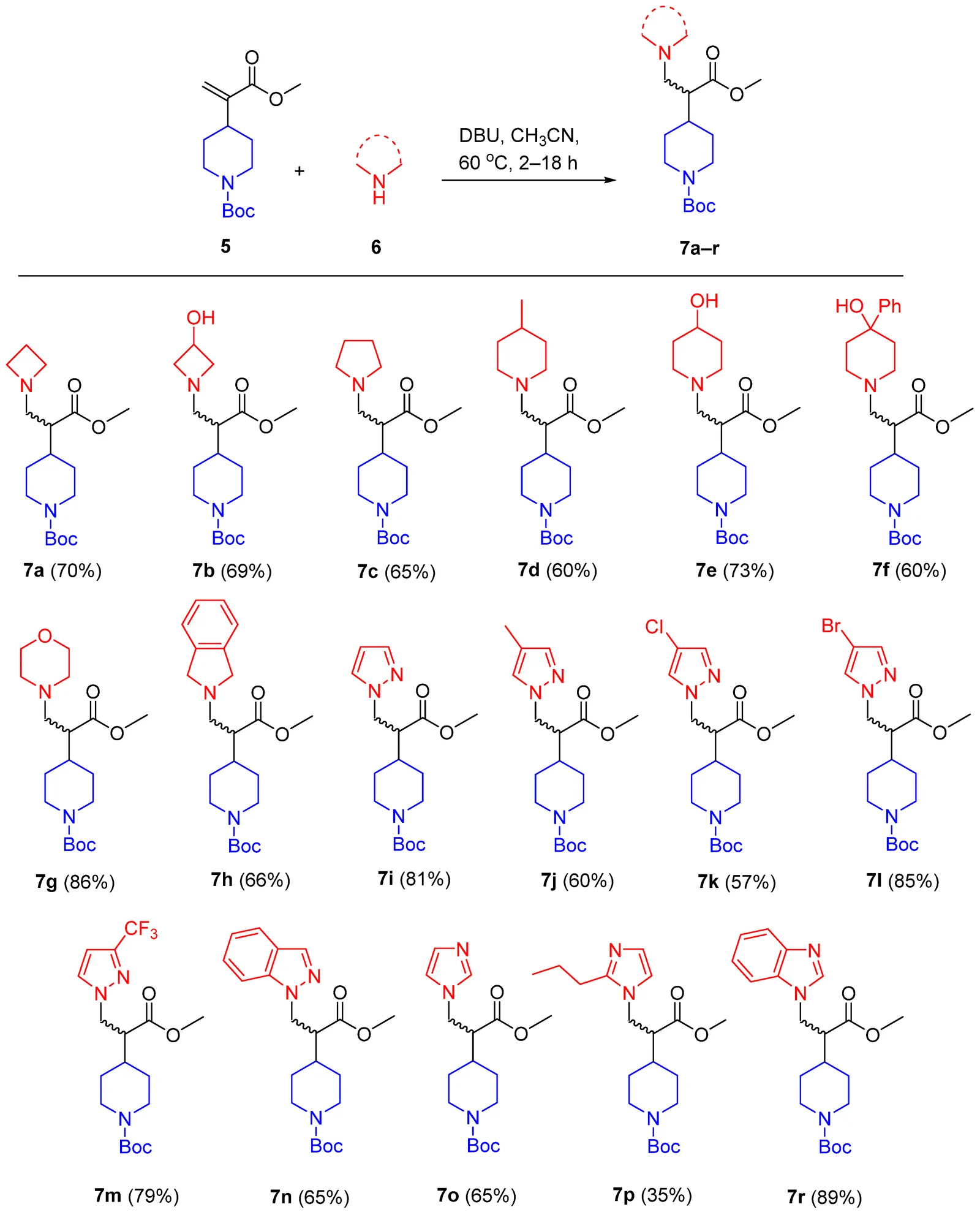
Synthesis of methyl 2-(*N*-Boc-piperidin-4-yl)-3-(*N*-heterocyclyl/heteroaryl)propionates **7a**–**r** via aza-Michael addition.

The aza-Michael addition of nitrogen nucleophiles to methyl *α*,*β*-unsaturated ester **5** was investigated as the key diversification step for the synthesis of methyl 2-(*N*-Boc-piperidin-4-yl)-3-(*N*-heterocyclyl/heteroaryl)propionate derivatives **7a**–**r** (Scheme 2). In the general procedure, Michael acceptor **5** was reacted with the corresponding *N*-heterocyclic nucleophile **6** in acetonitrile at 60 °C in the presence of DBU. Depending on the nucleophile, the reactions were carried out for 4–18 h and afforded the desired methyl propionate derivatives **7a**–**r** in 35–89% yield.

Several bases were initially screened using azetidine and pyrazole as representative nucleophiles in acetonitrile (Table 2). Reaction with DBU at 60 °C (Entry 5) gave the best results in both cases, affording azetidine derivative **7a** in 70% yield after 4 h and pyrazole derivative **7i** in 81% yield after 18 h. Inorganic bases generally provided lower yields, whereas NaH was ineffective for both **7a** and **7i**, with no detectable formation of the desired products. In the absence of a base, no detectable formation of either product was observed. Therefore, DBU was selected as the optimal base for further reactions.

After DBU had been identified as the most effective base, acetonitrile, dioxane, ethanol, and tetrahydrofuran were evaluated as solvents based on literature reports [39]. Acetonitrile provided the best results, whereas the other solvents resulted in markedly lower yields and, in some cases, no detectable formation of the desired products.

Finally, the influence of reaction temperature was investigated using DBU in acetonitrile. At room temperature, formation of both **7a** and **7i** was observed, although the reactions proceeded more slowly and less efficiently. At 40 °C, a substantial amount of starting material remained unreacted, whereas at 80 °C increased formation of side products was observed. Accordingly, 60 °C was established as the optimal reaction temperature, providing the most favorable balance between reaction rate and product formation.

**Scheme 3:**
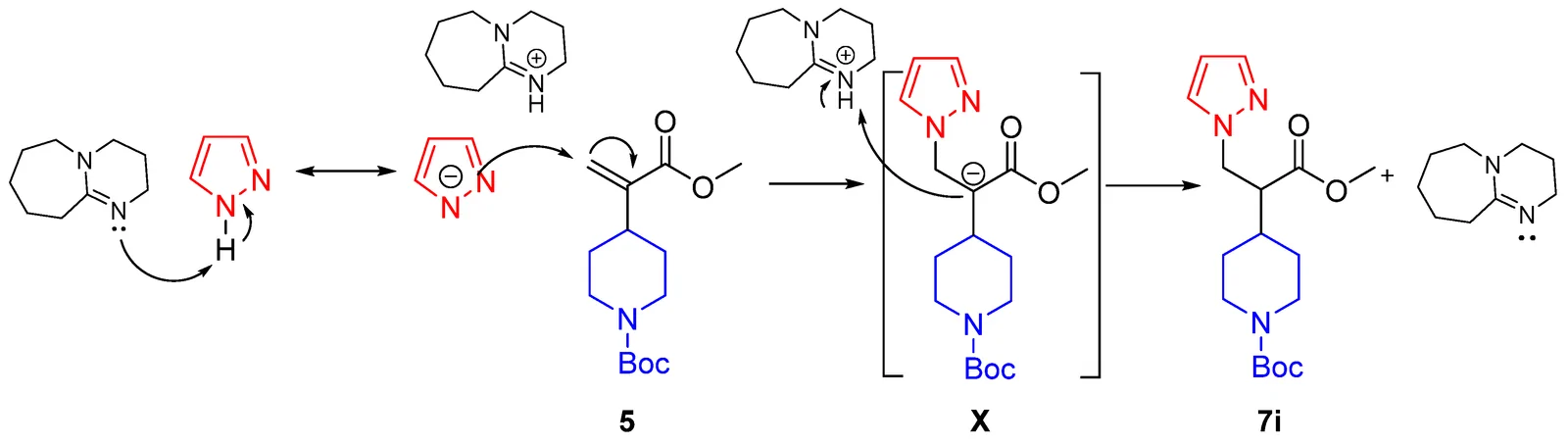
Proposed mechanism for the DBU-promoted aza-Michael addition of pyrazole to *α*,*β*-unsaturated ester **5**.

A plausible mechanism for the formation of pyrazole derivative **7i** under the employed DBU-promoted reaction conditions is proposed in Scheme 3. Initially, DBU deprotonates pyrazole to generate the corresponding pyrazolate anion and DBUH^+^. The pyrazolate anion then undergoes conjugate addition to the *β*-carbon of *α*,*β*-unsaturated ester **5**, generating intermediate X. Subsequent proton transfer from DBUH^+^ affords product **7i** and regenerates DBU. This proposal is consistent with the general mechanistic principles of aza-Michael reactions, for which different mechanistic pathways may operate depending on the Michael donor, Michael acceptor, and reaction conditions [49], as well as with reported base-promoted aza-Michael additions of pyrazoles [50] and DBU-promoted aza-Michael reactions [51].

Under the optimized DBU-promoted aza-Michael conditions, Michael acceptor **5** was reacted with various nitrogen nucleophiles (Scheme 2), affording the corresponding methyl 2-(*N*-Boc-piperidin-4-yl)-3-(*N*-heterocyclyl/heteroaryl)propionates **7a**–**r** in 35–89% yields. Saturated cyclic amines reacted smoothly to give products **7a**–**g** in good yields (60–86%) within 2–12 h, with most reactions reaching completion within 6 h. Isoindoline also proved to be a suitable nucleophile, affording product **7h** in 66% yield. In contrast, aza-heteroaromatic nucleophiles generally required longer reaction times (12–16 h) and afforded products **7i**–**r** in more variable yields (35–89%), consistent with their lower nucleophilicity [51,52,53,54,55]. Although isoindoline reacted readily under the applied conditions, indole remained unreactive, and no desired aza-Michael adduct was detected even after prolonged reaction times, increased DBU loading, or the use of alternative bases. This lack of reactivity is consistent with the relatively low *N*-nucleophilicity of indole and its preference for electrophilic substitution at the C-3 position; *N*-1- or C-2-functionalization generally becomes more favourable when the C-3 position is blocked [52,53,56].

**Scheme 4:**
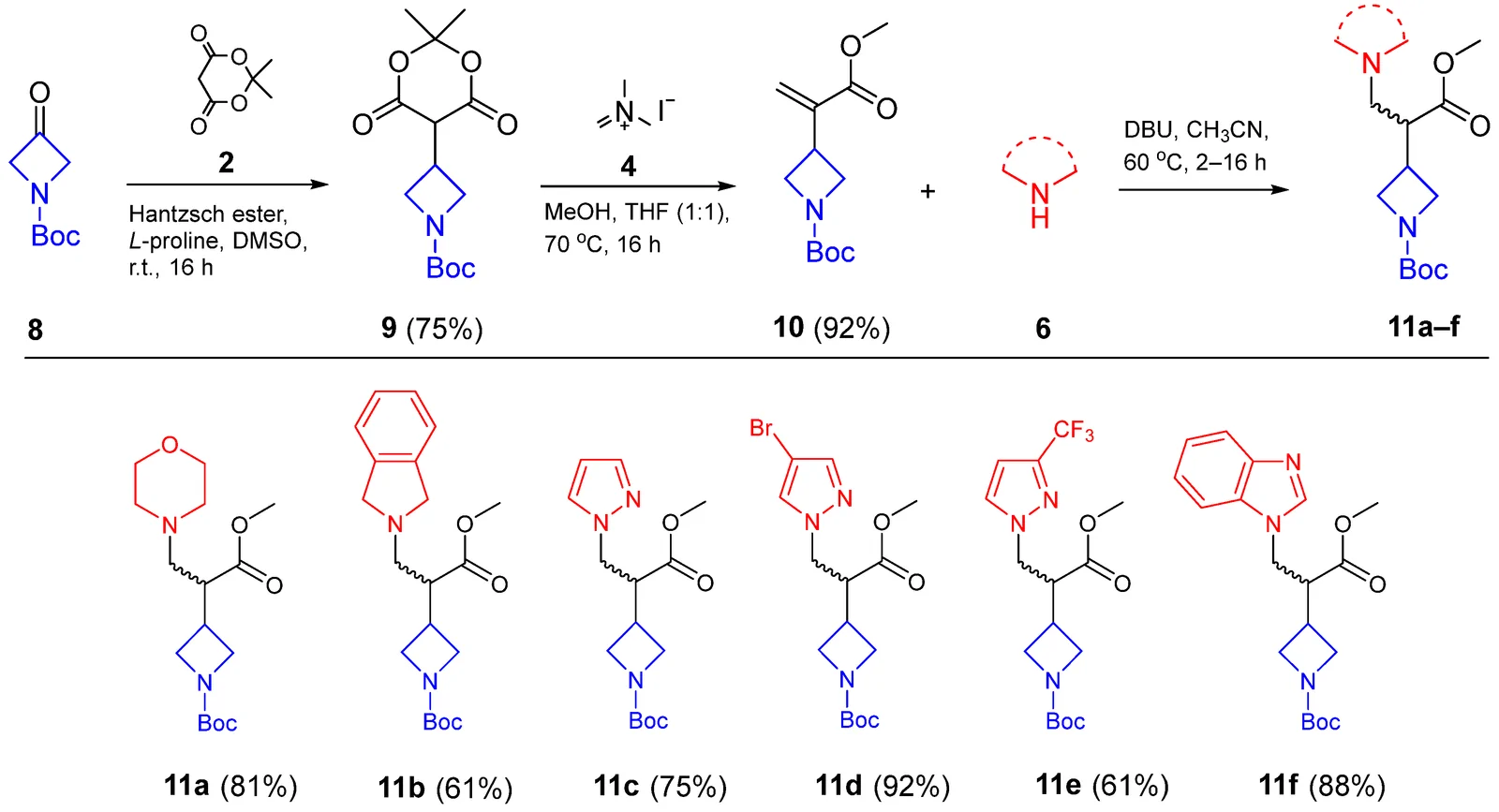
Synthesis of methyl 2-(*N*-Boc-azetidin-3-yl)-3-(*N*-heterocyclyl/heteroaryl)propionates **11a**–**f** via aza-Michael addition.

To further evaluate the applicability of the developed sequence, tert-butyl 3-oxoazetidine-1-carboxylate **8**, a strained nitrogen heterocycle relevant to medicinal chemistry [57,58,59], was employed as an additional ketone substrate (Scheme 4). Application of the optimized *L*-proline/Hantzsch ester-mediated conditions to ketone **8** and Meldrum’s acid afforded the corresponding azetidine-based Meldrum’s acid derivative **9** in 75% yield. Unlike the piperidine analogue **3**, compound **9** remained soluble in methanol and could not be isolated via precipitation; therefore, purification via column chromatography was required. Subsequent reaction of purified **9** with Eschenmoser’s salt, followed by methanolysis, furnished the corresponding methyl *α*,*β*-unsaturated ester **10** in 92% yield.

After the successful preparation of azetidine-derived methyl *α*,*β*-unsaturated ester **10**, its reactivity in the aza-Michael addition was examined using selected nitrogen nucleophiles that had performed well in the piperidine series (Scheme 4). In analogy to the optimized conditions developed for Michael acceptor **5**, compound **10** was reacted with the corresponding *N*-heterocyclic nucleophile in acetonitrile at 60 °C in the presence of DBU [39,51,52,53]. Depending on the nucleophile, the reactions proceeded within 4–18 h and afforded the corresponding methyl 2-(*N*-Boc-azetidin-3-yl)-3-(*N*-heterocyclyl/heteroaryl)propionate derivatives **11a**–**f** in 61–92% yields. Similar reactivity trends were observed for the azetidine series. Importantly, the strained azetidine scaffold remained intact under the applied reaction conditions throughout the multistep sequence.

Both piperidine- and azetidine-derived Michael acceptors reacted smoothly under the developed aza-Michael conditions and were compatible with structurally diverse nitrogen nucleophiles. In reactions involving aromatic NH-heterocycles containing multiple potentially nucleophilic nitrogen centers, no alternative regioisomeric products were detected via chromatographic analysis. Collectively, these results demonstrate that the developed methodology provides efficient access to structurally diverse piperidine- and azetidine-containing propanoic acid building blocks. The structures of the isolated compounds were assigned and confirmed via NMR spectroscopy.

**Figure 2:**
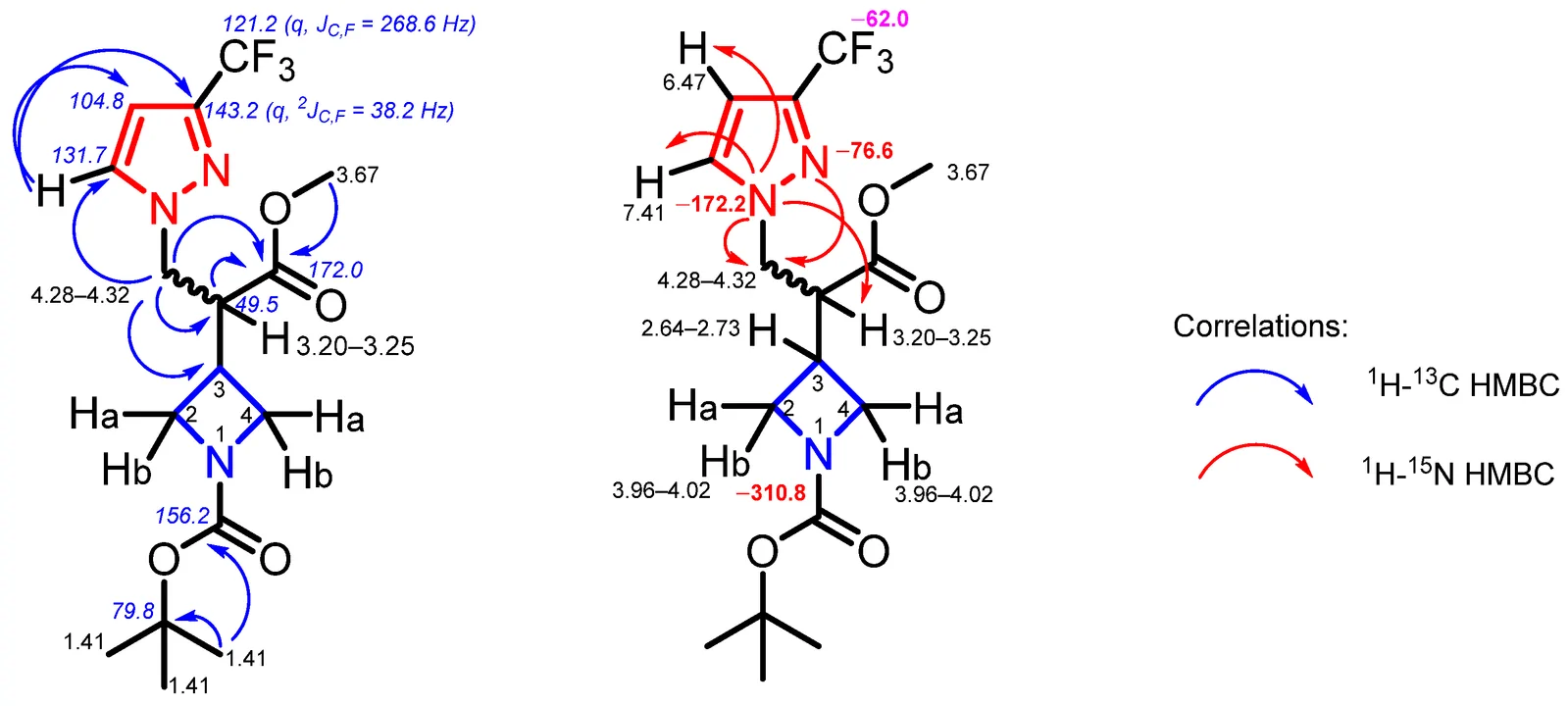
Relevant ^1^H-^13^C HMBC and ^1^H-^15^N HMBC correlations, as well as ^1^H-NMR (black), ^13^C-NMR (blue), ^15^N-NMR (red), and ^19^F-NMR (pink) chemical shifts, of compound **11e**.

Compound **11e** was subjected to a detailed spectral analysis. The molecular structure of the synthesized trifluoromethylated pyrazole derivative was unambiguously established through a comprehensive analysis of 1D (^1^H, ^13^C, ^19^F) and 2D (^1^H-^1^H COSY, ^1^H-^13^C HSQC, ^1^H-^13^C HMBC and ^1^H-^15^N HMBC) NMR spectra. In the ^1^H-NMR spectrum, the pyrazole ring system is clearly identified by two singlets at *δ*_H_ 7.41 ppm (for 5-H) and *δ*_H_ 6.47 ppm (for 4-H), characteristic of the pyrazole core protons. The methyl ester function is confirmed by a three-proton singlet at *δ*_H_ 3.67 ppm (COOCH_3_), which correlates with the ester carbonyl carbon resonance at *δ*_C_ 172.0 ppm in the ^13^C-NMR spectrum. Additionally, the *tert*-butoxycarbonyl (Boc) protecting group on the azetidine nitrogen is indicated by a highly intense singlet at *δ*_H_ 1.41 ppm integrating for nine protons (C(CH_3_)_3_), matching the quaternary carbon signal at *δ*_C_ 79.8 ppm and the carbamate carbonyl peak at *δ*_C_ 156.2 ppm (Figure 2).

The central core consists of an azetidine ring and a conformationally flexible aliphatic chain. Due to the diastereotopic environment generated by the adjacent ester-bearing chiral center (*δ*_H_ 3.20–3.25 ppm, *δ*_C_ 49.5 ppm), the methylene protons adjacent to the pyrazole ring appear as two distinct multiplets in the *δ*_H_ 4.28–4.43 ppm region. The azetidine protons appeared as three distinct signals due to their diastereotopic nature. A multiplet at *δ*_H_ 3.96–4.02 ppm (2H) was assigned to one pair of the diastereotopic protons adjacent to the nitrogen atom (from 2-H and 4-H), while the remaining two resonances were observed as separate multiplets at *δ*_H_ 3.73–3.76 ppm (1H) and *δ*_H_ 3.59–3.63 ppm (1H) (Figure 2).

The presence of the trifluoromethyl substituent (CF_3_) is conclusively demonstrated by both ^19^F- and ^13^C-NMR data. The ^19^F-NMR spectrum exhibits a sharp, single resonance at *δ*_F_ −62.0 ppm, typical for a pyrazole-bound CF_3_ group. In the ^13^C-NMR spectrum, this fluorine attachment induces characteristic heteronuclear carbon-fluorine coupling (*J*_C,F_); the CF_3_ carbon splits into a well-defined quartet centred at *δ*_C_ 121.2 ppm with a distinct one-bond coupling constant of *J*_C_,_F_ = 268.6 Hz. Furthermore, the adjacent pyrazole ring carbon appears as a quartet at *δ*_C_ 143.1 ppm with a two-bond coupling constant of ^2^*J*_C,F_ = 38.3 Hz. Finally, the connectivity of the nitrogen atoms within the heterocycles was corroborated using ^1^H-^15^N HMBC couplings, exhibiting distinctive cross-peaks that verify the site-selective conjugation of the pyrazole scaffold.

**Scheme 5:**
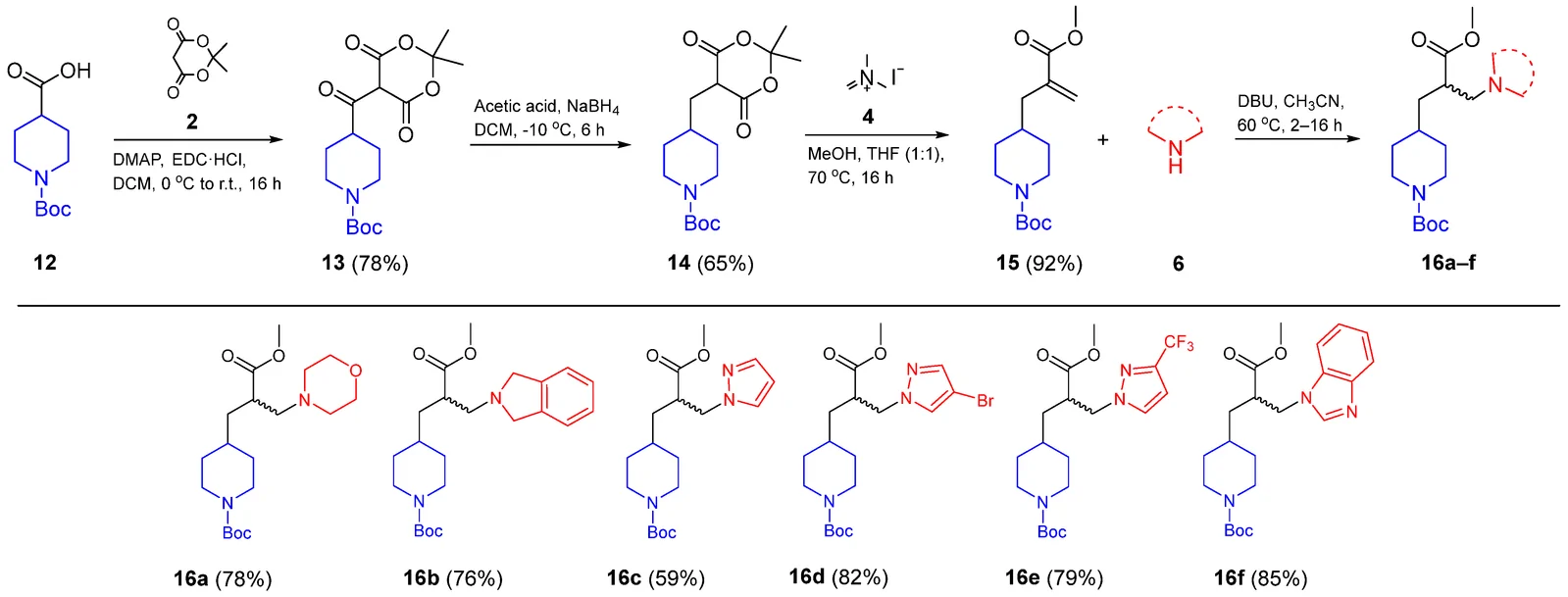
Synthesis of methyl 2-(*N*-heterocyclyl/heteroaryl)-3-(*N*-Boc-piperidin-4-yl)propionates **16a**–**f**.

**Scheme 6:**
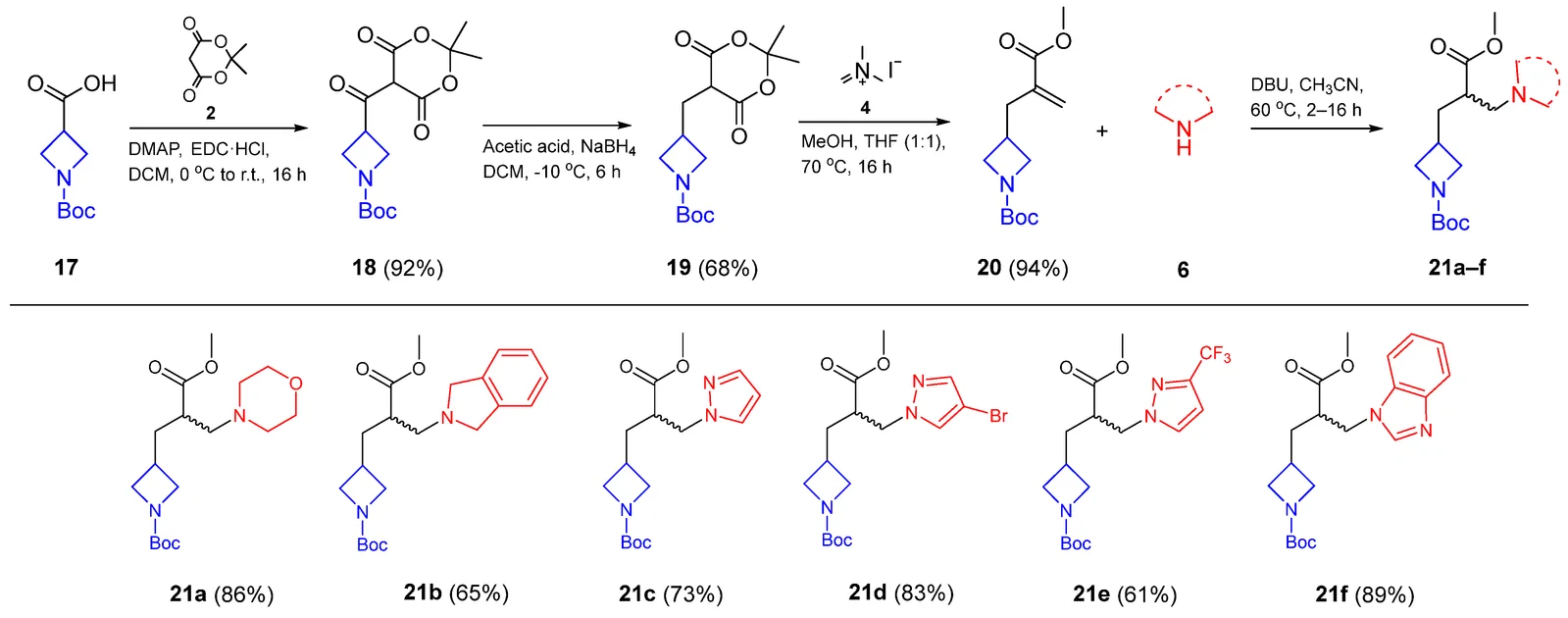
Acid-derived synthesis of methyl 2-(*N*-heterocyclylmethyl)-3-(*N*-Boc-azetidin-3-yl)propionates **21a**–**f**.

To complement the previously described ketone-derived series and further expand the scope of the developed methodology, a second series of propionate derivatives was prepared from *N*-Boc-piperidine-4-carboxylic acid (**12**) and *N*-Boc-azetidine-3-carboxylic acid (**17**) (Scheme 5 and Scheme 6). This approach provided homologated derivatives with substitution patterns complementary to those of the ketone-derived series.

*N*-Boc-piperidine-4-carboxylic acid **12** was first coupled with Meldrum’s acid 2 under EDC·HCl/DMAP-mediated conditions in DCM. The reaction was carried out from 0 °C to room temperature over 16 h and afforded acyl Meldrum’s acid derivative **13** in 78% yield [60,61]. In comparison with previously reported DCC-based procedures [62,63], the use of EDC·HCl was advantageous because the corresponding urea by-product could be readily removed during aqueous work-up, thereby simplifying purification [61]. Subsequent reduction of acyl Meldrum’s acid derivative **13** with NaBH_4_ in the presence of acetic acid in DCM at −10 °C for 6 h provided alkylated Meldrum’s acid derivative **14** in 65% yield. Intermediate **14** was then treated with Eschenmoser’s salt in a THF/MeOH mixture at 70 °C for 16 h, and the resulting methylenation/methanolysis sequence furnished piperidine-derived methyl *α*,*β*-unsaturated ester **15** in 92% yield [62].

Michael acceptor **15** was next used in the aza-Michael addition step with selected nitrogen nucleophiles that had previously shown good performance in the ketone-derived series. The reactions were performed under the optimized DBU-promoted conditions, using the corresponding *N*-heterocyclic nucleophile in acetonitrile at 60 °C. Depending on the nucleophile, the reactions proceeded within 2–16 h and afforded methyl 2-(*N*-heterocyclyl/heteroaryl)-3-(*N*-Boc-piperidin-4-yl)propionate derivatives **16a**–**f** in 59–85% yields. As observed for the ketone-derived series, saturated cyclic amines reacted smoothly, whereas aromatic NH-heterocycles generally required longer reaction times and provided more variable yields. For heterocyclic nucleophiles containing more than one potentially reactive nitrogen atom, no alternative regioisomeric products were detected by chromatographic analysis.

The same acid-derived sequence was subsequently applied to *N*-Boc-azetidine-3-carboxylic acid **17** to access the corresponding azetidine-containing analogues (Scheme 6). Coupling of acid **17** with Meldrum’s acid under EDC·HCl/DMAP-mediated conditions in DCM afforded acyl Meldrum’s acid derivative **18** in 92% yield. Reduction of compound **18** with NaBH_4_/AcOH in DCM at −10 °C gave alkylated Meldrum’s acid derivative **19** in 68% yield. Subsequent treatment of **19** with Eschenmoser’s salt in THF/MeOH at 70 °C, followed by methanolysis, furnished azetidine-derived methyl *α*,*β*-unsaturated ester **20** in 94% yield.

Michael acceptor **20** was then subjected to the optimized DBU-promoted aza-Michael addition conditions with representative nitrogen nucleophiles. Thus, compound **20** was reacted with the appropriate *N*-heterocycle in acetonitrile at 60 °C in the presence of DBU, providing methyl 2-(*N*-heterocyclyl/heteroaryl)-3-(*N*-Boc-azetidin-3-yl)propionate derivatives **21a**–**f** in 61–89% yields [39,51,52,53]. The reactivity profile of azetidine-derived acceptor **20** was comparable to that of the piperidine analogue **15**, and the strained azetidine scaffold was retained throughout the multistep sequence. Together, the ketone- and acid-derived strategies provide complementary access to regioisomeric series of structurally diverse piperidine- and azetidine-containing propanoate building blocks, while the remaining position is diversified through aza-Michael addition. The structures of the isolated compounds were assigned and confirmed via NMR analysis.

**Figure 3:**
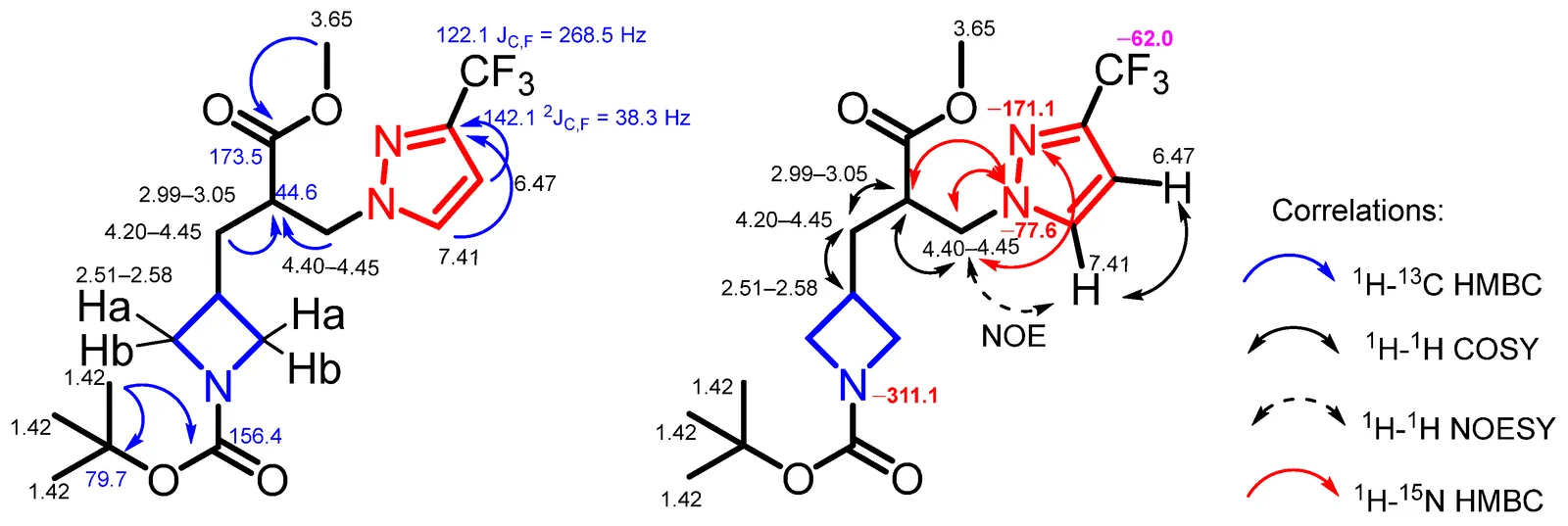
Relevant ^1^H-^13^C HMBC and ^1^H-^15^N HMBC, ^1^H-^1^H COSY and ^1^H-^1^H NOESY correlations, as well as ^1^H-NMR (black), ^13^C-NMR (blue), ^15^N-NMR (red) and ^19^F-NMR (pink) chemical shifts, of compound **21e**.

The structural assignment of compound **21e** was deduced via detailed spectral data analysis. In the ^1^H-NMR spectrum, the *tert*-butoxycarbonyl (Boc) protecting group was readily identified by a sharp 9H singlet at *δ*_H_ 1.42 ppm, which correlated with the quaternary carbon at *δ*_C_ 79.7 ppm and a carbamoyl carbonyl at *δ*_C_ 156.4 ppm. The methyl ester group appeared as a characteristic singlet at *δ*_H_ 3.65 ppm, exhibiting a diagnostic long-range HMBC correlation to the ester carbonyl at *δ*_C_ 173.5 ppm. The 3-(trifluoromethyl)-1*H*-pyrazole moiety displayed two characteristic aromatic singlets at *δ*_H_ 7.41 and 6.47 ppm. The presence of the trifluoromethyl substituent at the C-3 position was confirmed by characteristic resonances in the ^13^C-NMR spectrum, including a quartet at *δ*_C_ 122.1 ppm assigned to -CF_3_ and a quartet at *δ*_C_ 142.1 ppm assigned to C-3. Additionally, the ^1^H-^1^H NOESY correlation between pyrazole-linked CH_2_ protons *δ*_H_ 4.40–4.45 ppm and pyrazole H-5 at *δ*_H_ 7.41 ppm was observed (Figure 3).

The connectivity of the aliphatic backbone was traced via the ^1^H-^1^H COSY spectrum. The central (CH) methine proton at *δ*_H_ 2.99–3.05 ppm showed clear cross-peaks with both the adjacent (CH_2_-) methylene spacer leading to the azetidine core and the (CH_2_-N) group attached to the pyrazole ring.

The CH_2_-N methylene protons *δ*_H_ 4.40–4.45 ppm exhibited ^1^H-^13^C HMBC cross-peaks with the central methine carbon at *δ*_C_ 44.6 ppm, while the methylene protons connecting the methine centre to the azetidine ring showed correlations to the same carbon. Pyrazole ring protons H-4 *δ*_H_ 6.47 ppm and H-5 *δ*_H_ 7.41 showed 2- or 3-bond correlations with C-3 carbon at *δ*_C_ 142.1 ppm. The ^1^H-^15^N HMBC spectrum revealed three distinct nitrogen environments. A strong cross-peak was observed between the CH_2_-N protons and the pyrrole-like nitrogen *N*-2 at *δ*_N_ −171.1 ppm, confirming the regioselective attachment. The remaining nitrogen signals were unambiguously assigned to the pyridine-like pyrazole *N*-1 at *δ*_N_ −77.6 ppm and the carbamate nitrogen of the azetidine ring at *δ*_N_ −311.1 ppm (Figure 3).

To evaluate the possibility of direct enantiomeric separation, chiral HPLC analyses were performed for four representative compounds (**7r**, **11f**, **16f**, and **21f**), including one example from each synthetic series (Figures S139–S142). Under the chromatographic conditions employed, compounds **7r**, **16f**, and **21f** showed a single unresolved peak. For compound **11f**, partial enantiomeric resolution was observed; however, baseline separation was not achieved, precluding reliable determination of the relative peak areas and enantiomeric composition. These results indicate that direct chiral HPLC resolution of the synthesized compounds would require further optimization of the chromatographic conditions and/or evaluation of alternative chiral stationary phases.

An alternative approach to the resolution of structurally related racemic propanoic acid derivatives was previously demonstrated by Gudelis et al. [64]. Racemic 2-(*N*-Boc-azetidin-3-yl)-2-alkylpropanoic acids were converted into diastereomeric derivatives using (*S*)-4-benzyl-2-oxazolidinone as a resolving agent. The resulting diastereomers were successfully separated by conventional column chromatography and subsequently converted into the corresponding enantiomerically pure carboxylic acids. Thus, derivatization followed by diastereomeric separation may represent an alternative strategy for the stereochemical resolution of the compounds described herein.

## 3. Materials and Methods

### 3.1. General Information

All starting materials were purchased from commercial suppliers and were used as received. Flash column chromatography was performed on Silica Gel 60 Å (Merck KGaA, Darmstadt, Germany). Vacuum distillation was performed in a Büchi Model B580 GKR oven (Büchi Labortechnik AG, Flawil, Switzerland). Thin-layer chromatography was carried out on Silica Gel plates (Merck Kieselgel 60 F254) and visualised by UV light (254 nm) (Merck KGaA, Darmstadt, Germany). Melting points were determined using a Büchi M-565 melting point apparatus (Büchi Labortechnik AG, Flawil, Switzerland) and were uncorrected. The IR spectra were recorded on a Bruker Vertex 70v FT-IR spectrometer (Bruker Optik GmbH, Ettlingen, Germany) using neat samples and are reported in the frequency of absorption (cm^−1^). Mass spectra were obtained using a Shimadzu LCMS-2020 (ESI^+^) spectrometer (Shimadzu Corporation, Kyoto, Japan). High-resolution mass spectra were measured using a Bruker MicrOTOF-Q III (ESI^+^) apparatus (Bruker Daltonik GmbH, Bremen, Germany). Accurate measurements were achieved using the internal mass calibration of each sample using sodium formate calibration solution as a standard procedure, with a standard deviation always less than 1 ppm. In addition, all data files were recalibrated with an internal standard of sodium formate injected prior to initial sample elution for each sample. ^1^H-NMR and ^13^C-NMR spectra were recorded from CDCl_3_ solutions at 25 °C on a Bruker Avance III 400 instrument (400 MHz for ^1^H, 100 MHz for ^13^C) using a directly detecting BBO probe (Bruker Bio Spin International AG, Faellanden, Switzerland). ^15^N-NMR spectra were recorded from CDCl_3_ solutions at 25 °C on a Bruker Avance III 400 instrument (40 MHz for ^15^N) using a directly detecting BBO probe. The chemical shifts (*δ*), expressed in ppm, were relative to tetramethylsilane (TMS). ^15^N-NMR spectra were referenced against neat external nitromethane (coaxial capillary). ^19^F-NMR spectra (376.46 MHz, absolute referencing via Ξ ratio) were obtained on a Bruker Avance III 400 instrument with a directly detecting broadband observe probe (BBO). The following abbreviations were used in reporting the NMR data: Pip, piperidine; Ind, indazole; Pyr, pyrazole; Imid, imidazole; Az, azetidine; *i*-Ind, isoindole; Mor, morpholine; OH-Pip, hydroxypiperidine; Ph-Pip, phenylpiperidine; Pyrr, pyrrolidine; Bim, benzimidazole; Pim, propylimidazole.

### 3.2. Synthetic Procedures

#### 3.2.1. General Procedure for Compound **3**

Corresponding ketone (1 mmol), 2,2-dimethyl-1,3-dioxane-4,6-dione **2** (1 mmol) and diethyl 1,4-dihydro-2,6-dimethyl-3,5-pyridinedicarboxylate (1 mmol) were dissolved in DMSO (1 mL). After 10 min, *L*-proline (0.2 mmol) was added, and the resulting mixture was stirred at room temperature for 18 h. After completion of the reaction, DMSO was removed under reduced pressure at elevated temperature, and methanol was added to the residue. The resulting precipitate was collected by filtration through a Büchner funnel and dried under reduced pressure to give product **3**.

*Tert*-butyl 4-(2,2-dimethyl-4,6-dioxo-1,3-dioxan-5-yl)piperidine-1-carboxylate (**3**)

1-(*tert*-butoxycarbonyl)-4-piperidone **1** (0.2 g, 1 mmol), 2,2-dimethyl-1,3-dioxane-4,6-dione **2** (0.14 g, 1 mmol) and diethyl 1,4-dihydro-2,6-dimethyl-3,5-pyridinedicarboxylate (0.25 g, 1 mmol) were dissolved in DMSO (1 mL). After 10 min, *L*-proline (0.02 g, 0.2 mmol) was added, and the resulting mixture was stirred at room temperature for 18 h. After completion of the reaction, DMSO was removed under reduced pressure, and methanol was added to the residue. The resulting precipitate was collected by filtration through a Büchner funnel and dried under reduced pressure to give **3** (0.232 g, 71%) as a white solid, mp 160–161 °C. ^1^H-NMR (400 MHz, CDCl_3_): *δ*_H_ ppm 1.44 (s, 9H, C(CH_3_)_3_), 1.53–1.56 (m, 2H, Pip 3,5-H), 1.74 (s, 3H, CH_3_), 1.76 (s, 3H, CH_3_), 1.82–1.93 (m, 2H, Pip 3,5-H), 2.48–2.57 (m, 1H, Pip 4-H), 2.60–2.78 (m, 2H, Pip 2,6-H), 3.41–3.42 (m, 1H, CH), 4.12–4.31 (m, 2H, Pip 4,6-H). ^13^C-NMR (101 MHz, CDCl_3_): *δ*_C_ ppm 27.5 (CH_3_), 28.3 (Pip 2 × CH_2_), 28.4 (CH_3_), 28.6 (COOC(CH_3_)_3_), 37.1 (Pip C-4), 44.2 (Pip 2 × CH_2_), 50.5 (CH), 79.7 (COOC(CH_3_)_3_), 105.1 (C), 154.7 (COOC(CH_3_)_3_), 164.6 (2 × C=O). ^15^N-NMR (71 MHz, CDCl_3_): *δ*_N_ ppm −295.0 (*N*-Boc). IR (FT-IR, *ν*_max_, cm^−1^): 2979, 2850 (CH_aliph_), 1789 (C=O), 1737 (C=O), 1674 (C=O), 1425, 1167, 1135 (C=C, C–N, C–O–C). HRMS (ESI^+^) C_16_H_25_NNaO_6_ ([M + Na]^+^) calcd. 350.1574, found 350.1574.

#### 3.2.2. General Procedure for Compound **5**

A 50-mL round-bottomed flask was charged under nitrogen atmosphere with compound **3** (1 mmol) and *N*,*N*-dimethylmethyleneiminium iodide **4** (2 mmol). The solids were dissolved in THF and MeOH (1:1). The reaction mixture was then heated to 70 °C and stirred for 18 h. Upon cooling of the mixture to room temperature, the solvents were removed in vacuo, and the residue was taken up in ethyl acetate, washed with sat. NaHCO_3_ soln, 10% aq. KHSO_4_ and brine. The organic layer was dried with anhydrous sodium sulfate, filtered, and then concentrated under reduced pressure. The resulting mixture was purified via flash column chromatography to give product **5**.

*Tert*-butyl 4-(3-methoxy-3-oxoprop-1-en-2-yl)piperidine-1-carboxylate (**5**)

A 50-mL round-bottomed flask was charged under nitrogen atmosphere with compound **3** (0.33 g, 1 mmol) and *N*,*N*-dimethylmethyleneiminium iodide **4** (0.37 g, 2 mmol). The solids were dissolved in THF (6 mL) and MeOH (6 mL). The reaction mixture was then heated to 70 °C and stirred for 18 h. Upon cooling of the mixture to room temperature, the solvents were removed in vacuo, and the yellow residue was taken up in ethyl acetate (15 mL), washed with sat. NaHCO_3_ soln (15 mL), 10% aq. KHSO_4_ (15 mL) and brine (15 mL). The organic layer was dried with anhydrous sodium sulfate, filtered, and then concentrated under reduced pressure. The resulting mixture was purified via flash column chromatography (eluent *n*-hexane/ethyl acetate, *v*/*v*, 4:1) to give the corresponding product **5** (0.234 g, 87%) as a colorless liquid. ^1^H-NMR (400 MHz, CDCl_3_): *δ*_H_ ppm 1.24–1.35 (m, 2H, Pip 3,5-H), 1.44 (s, 9H, C(CH_3_)_3_), 1.73–1.76 (m, 2H, Pip 3,5-H), 2.55–2.61 (m, 1H, Pip 4-H), 2.70–2.77 (m, 2H, Pip 2,6-H), 3.75 (s, 3H, OCH_3_), 4.16 (s, 2H, Pip 2,6-H), 5.50 (s, 1H, C=CH_2_), 6.17 (s, 1H, C=CH_2_). ^13^C-NMR (101 MHz, CDCl_3_): *δ*_C_ ppm 28.5 (COOC(CH_3_)_3_), 31.3 (Pip 2 × CH_2_), 37.4 (Pip C-4), 44.1 (Pip 2 × CH_2_), 51.9 (OCH_3_), 79.4 (COOC(CH_3_)_3_), 123.2 (C=CH_2_), 144.3 (C=CH_2_), 154.8 (COOC(CH_3_)_3_), 167.4 (COOCH_3_). ^15^N-NMR (71 MHz, CDCl_3_): *δ*_N_ ppm −292.4 (*N*-Boc). IR (FT-IR, *ν*_max_, cm^−1^): 2949, 2854 (CH_aliph_), 1720 (C=O), 1689 (C=O), 1419, 1144 (C=C, C–N, C–O–C). HRMS (ESI^+^) C_14_H_23_NNaO_4_ ([M + Na]^+^) calcd. 292.1519, found 292.1519.

#### 3.2.3. General Procedure for Compounds **7a**–**r**

An appropriate *N*-heterocyclic compound **6** (1.1 mmol), DBU (1.15 mmol), and *tert*-butyl 4-(3-methoxy-3-oxoprop-1-en-2-yl)piperidine-1-carboxylate **5** (1 mmol) were dissolved in acetonitrile (1 mL) and stirred at 60 °C for 4–18 h. The reaction mixture was diluted with water (10 mL) and extracted with ethyl acetate (2 × 15 mL). The organic layer was dried with anhydrous sodium sulfate, filtered, and then concentrated under reduced pressure. The resulting mixtures were purified via flash column chromatography to provide products **7a**–**r**.

*Tert*-butyl 4-[3-(azetidin-1-yl)-1-methoxy-1-oxopropan-2-yl]piperidine-1-carboxylate (**7a**)

Compound **7a** was obtained from **5** (0.27 g, 1 mmol), azetidine hydrochloride (0.1 g, 1.1 mmol), and DBU (0.17 mL, 1.15 mmol). The reaction was stirred at 60 °C for 4 h. The obtained residue was purified via flash column chromatography (eluent methylene chloride/methanol, *v*/*v*, 100:1) to give **7a** (0.228 g, 70%) as a yellowish liquid. ^1^H-NMR (400 MHz, CDCl_3_): *δ*_H_ ppm 1.13–1.27 (m, 2H, Pip 3,5-H), 1.43 (s, 9H, C(CH_3_)_3_), 1.45–1.49 (m, 1H, Pip 4-H), 1.63–1.70 (m, 2H, Pip 3,5-H), 2.01 (p, *J* = 7.0 Hz, 2H, Az 3-CH_2_), 2.20–2.26 (m, 1H, CHCOOCH_3_), 2.44–2.48 (m, 1H, Az-CH_2_), 2.59–2.66 (m, 2H, Pip 2,6-H), 2.70–2.75 (m, 1H, Az-CH_2_), 3.08–3.19 (m, 4H, Az 2,4-CH_2_), 3.67 (s, 3H, OCH_3_), 4.07 (s, 2H, Pip 2,6-H). ^13^C-NMR (101 MHz, CDCl_3_): *δ*_C_ ppm 17.7 (Az C-3), 28.6 (COOC(CH_3_)_3_), 29.8 (Pip C-4), 30.2 (Pip CH_2_), 37.2 (Pip CH_2_), 44.0 (Pip 2 × CH_2_), 49.9 (CHCOOCH_3_), 51.6 (OCH_3_), 55.6 (Az C-2,4), 59.5 (Az-CH_2_), 79.5 (COOC(CH_3_)_3_), 154.8 (COOC(CH_3_)_3_), 175.0 (COOCH_3_). ^15^N-NMR (71 MHz, CDCl_3_): *δ*_N_ ppm −342.0 (*N*-1), *N*-Boc was not found. IR (FT-IR, *ν*_max_, cm^−1^): 2930, 2820 (CH_aliph_), 1735 (C=O), 1689 (C=O), 1422, 1159 (C=C, C–N, C–O–C). HRMS (ESI^+^) C_17_H_31_N_2_O_4_ ([M + H]^+^) calcd. 327.2278, found 327.2278.

*Tert*-butyl 4-[3-(3-hydroxyazetidin-1-yl)-1-methoxy-1-oxopropan-2-yl]piperidine-1-carboxylate (**7b**)

Compound **7b** was obtained from **5** (0.27 g, 1 mmol), 3-hydroxyazetidine hydrochloride (0.12 g, 1.1 mmol), and DBU (0.17 mL, 1.15 mmol). The reaction was stirred at 60 °C for 6 h. The obtained residue was purified via flash column chromatography (eluent methylene chloride/methanol, *v*/*v*, 100:2) to give **7b** (0.236 g, 69%) as a yellowish liquid. ^1^H-NMR (400 MHz, CDCl_3_): *δ*_H_ ppm 1.12–1.23 (m, 2H, Pip 3,5-H), 1.42 (s, 9H, C(CH_3_)_3_), 1.43–1.47 (m, 1H, Pip 4-H), 1.61–1.68 (m, 2H, Pip 3,5-H), 2.20–2.26 (m, 1H, CHCOOCH_3_), 2.48–2.52 (m, 1H, Az-CH_2_), 2.59–2.63 (m, 2H, Pip 2,6-H), 2.78–2.89 (m, 3H, Az CH_2_, Az-CH_2_), 3.28 (s, 1H, OH), 3.54–3.63 (m, 2H, Az CH_2_), 3.66 (s, 3H, OCH_3_), 4.05 (s, 2H, Pip 2,6-H), 4.33–4.36 (m, 1H, Az 3-H). ^13^C-NMR (101 MHz, CDCl_3_): *δ*_C_ ppm 28.5 (COOC(CH_3_)_3_), 29.7 (Pip C-4), 30.1 (Pip CH_2_), 37.2 (Pip CH_2_), 43.9 (Pip 2 × CH_2_), 50.1 (CHCOOCH_3_), 51.7 (OCH_3_), 59.4 (Az-CH_2_), 62.4 (Az C-3), 64.5 (Az CH_2_), 64.7 (Az CH_2_), 79.6 (COOC(CH_3_)_3_), 154.8 (COOC(CH_3_)_3_), 174.8 (COOCH_3_). ^15^N-NMR (71 MHz, CDCl_3_): *δ*_N_ ppm −356.2 (*N*-1), −292.9 (*N*-Boc). IR (FT-IR, *ν*_max_, cm^−1^): 3420 (O-H), 2940, 2847 (CH_aliph_), 1732 (C=O), 1688 (C=O), 1424, 1160 (C=C, C–N, C–O–C). HRMS (ESI^+^) C_17_H_31_N_2_O_5_ ([M + H]^+^) calcd. 343.2227, found 343.2227.

*Tert*-butyl 4-[1-methoxy-1-oxo-3-(pyrrolidin-1-yl)propan-2-yl]piperidine-1-carboxylate (**7c**)

Compound **7c** was obtained from **5** (0.27 g, 1 mmol), pyrrolidine (0.09 mL, 1.1 mmol), and DBU (0.17 mL, 1.15 mmol). The reaction was stirred at 60 °C for 12 h. The obtained residue was purified via flash column chromatography (eluent methylene chloride/methanol, *v*/*v*, 100:2) to give **7c** (0.221 g, 65%) as a yellowish liquid. ^1^H-NMR (400 MHz, CDCl_3_): *δ*_H_ ppm 1.14–1.29 (m, 3H, Pip), 1.43 (s, 9H, C(CH_3_)_3_), 1.49–1.53 (m, 1H, Pip), 1.69–1.73 (m, 5H, Pip, Pyrr), 2.43–2.67 (m, 8H, Pip, Pyrr, CHCOOCH_3_), 2.84–2.90 (m, 1H, Pyrr), 3.67 (s, 3H, OCH_3_), 4.08 (s, 2H, Pip). ^13^C-NMR (101 MHz, CDCl_3_): *δ*_C_ ppm 23.6 (Pyrr 2 × CH_2_), 28.6 (COOC(CH_3_)_3_), 29.8 (Pip C-4), 30.2 (Pip CH_2_), 37.8 (Pip CH_2_), 43.8 (Pip 2 × CH_2_), 50.8 (CHCOOCH_3_), 51.6 (OCH_3_), 54.4 (Pyrr CH_2_), 56.1 (Pyrr 2 × CH_2_), 79.5 (COOC(CH_3_)_3_), 154.8 (COOC(CH_3_)_3_), 175.2 (COOCH_3_). ^15^N-NMR (71 MHz, CDCl_3_): *δ*_N_ ppm −329.1 (*N*-1), *N*-Boc was not found. IR (FT-IR, *ν*_max_, cm^−1^): 2934, 2854 (CH_aliph_), 1734 (C=O), 1689 (C=O), 1422, 1160 (C=C, C–N, C–O–C). HRMS (ESI^+^) C_18_H_33_N_2_O_4_ ([M + H]^+^) calcd. 341.2435, found 341.2435.

*Tert*-butyl 4-[1-methoxy-3-(4-methylpiperidin-1-yl)-1-oxopropan-2-yl]piperidine-1-carboxylate (**7d**)

Compound **7d** was obtained from **5** (0.27 g, 1 mmol), 4-methylpiperidine (0.13 mL, 1.1 mmol), and DBU (0.17 mL, 1.15 mmol). The reaction was stirred at 60 °C for 12 h. The obtained residue was purified via flash column chromatography (eluent methylene chloride/methanol, *v*/*v*, 100:2) to give **7d** (0.221 g, 60%) as a yellowish liquid. ^1^H-NMR (400 MHz, CDCl_3_): *δ*_H_ ppm 0.88 (d, *J* = 6.3 Hz, 3H, Pip-CH_3_), 1.11–1.28 (m, 5H, Pip), 1.44 (s, 9H, C(CH_3_)_3_), 1.48–1.56 (m, 3H, Pip), 1.65–1.72 (m, 2H, Pip), 1.79–1.85 (m, 1H, CHCOOCH_3_), 1.95–2.01 (m, 1H, Pip), 2.38–2.42 (m, 1H, Pip), 2.46–2.52 (m, 1H, Pip-CH_2_), 2.61–2.71 (m, 4H, Pip), 2.86–2.89 (m, 1H, Pip-CH_2_), 3.66 (s, 3H, OCH_3_), 4.08 (s, 2H, Pip). ^13^C-NMR (101 MHz, CDCl_3_): *δ*_C_ ppm 22.0 (Pip-CH_3_), 28.6 (COOC(CH_3_)_3_), 30.0 (Pip CH_2_), 30.2 (Pip CH_2_), 30.9 (Pip CH), 34.5 (Pip CH_2_), 34.6 (Pip CH_2_), 37.8 (Pip CH_2_), 43.9 (Pip 2 × CH_2_), 49.5 (CHCOOCH_3_), 51.5 (OCH_3_), 53.6 (Pip-CH_2_), 55.0 (Pip CH), 58.8 (Pip CH_2_), 79.5 (COOC(CH_3_)_3_), 154.9 (COOC(CH_3_)_3_), 175.4 (COOCH_3_). ^15^N-NMR (71 MHz, CDCl_3_): *δ*_N_ ppm −226.3 (*N*-1), *N*-Boc was not found. IR (FT-IR, *ν*_max_, cm^−1^): 2924, 2847 (CH_aliph_), 1736 (C=O), 1691 (C=O), 1421, 1159 (C=C, C–N, C–O–C). HRMS (ESI^+^) C_20_H_37_N_2_O_4_ ([M + H]^+^) calcd. 369.2748, found 369.2748.

*Tert*-butyl 4-[3-(4-hydroxypiperidin-1-yl)-1-methoxy-1-oxopropan-2-yl]piperidine-1-carboxylate (**7e**)

Compound **7e** was obtained from **5** (0.27 g, 1 mmol), 4-hydroxypiperidine (0.11 g, 1.1 mmol), and DBU (0.17 mL, 1.15 mmol). The reaction was stirred at 60 °C for 12 h. The obtained residue was purified via flash column chromatography (eluent methylene chloride/methanol, *v*/*v*, 100:2) to give **7e** (0.270 g, 73%) as a yellowish liquid. ^1^H-NMR (400 MHz, CDCl_3_): *δ*_H_ ppm 1.14–1.26 (m, 2H, Pip), 1.44 (s, 9H, C(CH_3_)_3_), 1.50–1.52 (m, 2H, Pip), 1.68–1.73 (m, 2H, Pip), 1.79–1.86 (m, 3H, Pip), 2.00–2.05 (m, 1H, Pip), 2.18–2.23 (m, 1H, Pip), 2.40–2.51 (m, 2H, OH-Pip-CH_2_, CHCOOCH_3_), 2.61–2.69 (m, 4H, Pip, OH-Pip-CH_2_), 2.79–2.84 (m, 1H, Pip), 3.61–3.66 (m, 1H, CH-OH), 3.68 (s, 3H, OCH_3_), 4.08 (s, 2H, Pip). ^13^C-NMR (101 MHz, CDCl_3_): *δ*_C_ ppm 28.5 (COOC(CH_3_)_3_), 30.0 (Pip CH_2_), 30.1 (Pip CH_2_), 34.6 (Pip CH_2_), 34.7 (Pip CH_2_), 37.6 (Pip CH_2_), 43.8 (Pip 2 × CH_2_), 49.7 (CHCOOCH_3_), 50.9 (Pip CH_2_), 51.5 (OCH_3_), 51.9 (Pip 2 × CH_2_), 58.2 (OH-Pip-CH_2_), 68.1 (Pip-OH C-4), 79.5 (COOC(CH_3_)_3_), 154.8 (COOC(CH_3_)_3_), 175.2 (COOCH_3_). ^15^N-NMR (71 MHz, CDCl_3_): *δ*_N_ ppm −336.5 (*N*-1), *N*-Boc was not found. IR (FT-IR, *ν*_max_, cm^−1^): 3447 (O-H), 2939, 2852 (CH_aliph_), 1733 (C=O), 1688 (C=O), 1424, 1160 (C=C, C–N, C–O–C). HRMS (ESI^+^) C_19_H_35_N_2_O_5_ ([M + H]^+^) calcd. 371.2540, found 371.2541.

*Tert*-butyl 4-[3-(4-hydroxy-4-phenylpiperidin-1-yl)-1-methoxy-1-oxopropan-2-yl]piperidine-1-carboxylate (**7f**)

Compound **7f** was obtained from **5** (0.27 g, 1 mmol), 4-hydroxy-4-phenylpiperidine (0.19 g, 1.1 mmol), and DBU (0.17 mL, 1.15 mmol). The reaction was stirred at 60 °C for 8 h. The obtained residue was purified via flash column chromatography (eluent methylene chloride/methanol, *v*/*v*, 100:2) to give **7f** (0.268 g, 60%) as a yellowish liquid. ^1^H-NMR (400 MHz, CDCl_3_): *δ*_H_ ppm 1.16–1.30 (m, 2H, Pip), 1.44 (s, 9H, C(CH_3_)_3_), 1.50–1.53 (m, 1H, Pip), 1.71–1.77 (m, 4H, Pip), 2.00–2.15 (m, 2H, Pip), 2.36–2.42 (m, 1H, Pip), 2.50–2.68 (m, 6H, Pip 2 × CH_2_, Ph-Pip-CH_2_, CHCOOCH_3_), 2.73–2.78 (m, 1H, Ph-Pip-CH_2_), 2.82–2.85 (m, 1H, Pip), 3.69 (s, 3H, OCH_3_), 4.10 (s, 2H, Pip), 7.22–7.28 (m, 1H, Ph 4-H), 7.32–7.36 (m, 2H, Ph 3,5-H), 7.47–7.49 (m, 2H, Ph 2,6-H). ^13^C-NMR (101 MHz, CDCl_3_): *δ*_C_ ppm 28.5 (COOC(CH_3_)_3_), 30.0 (Ph-Pip CH_2_), 30.1 (Ph-Pip CH_2_), 37.7 (Pip CH_2_), 38.5 (Ph-Pip CH_2_), 38.5 (Ph-Pip CH_2_), 44.0 (Pip 2 × CH_2_), 48.9 (Pip CH_2_), 49.4 (CHCOOCH_3_), 50.5 (Pip CH_2_), 51.5 (OCH_3_), 58.4 (Ph-Pip-CH_2_), 71.1 (Ph-Pip C-4), 79.5 (COOC(CH_3_)_3_), 124.7 (Ph C-2,6), 127.0 (Ph C-4), 128.4 (Ph C-3,5), 148.5 (Ph C-1), 154.8 (COOC(CH_3_)_3_), 175.2 (COOCH_3_). ^15^N-NMR (71 MHz, CDCl_3_): *δ*_N_ ppm −336.4 (*N*-1), -292.5 (*N*-Boc). IR (FT-IR, *ν*_max_, cm^−1^): 3445 (O-H), 2944, 2820 (CH_aliph_), 1733 (C=O), 1689 (C=O), 1425, 1159 (C=C, C–N, C–O–C). HRMS (ESI^+^) C_25_H_39_N_2_O_5_ ([M + H]^+^) calcd. 447.2853, found 447.2854.

*Tert*-butyl 4-[1-methoxy-3-(morpholin-4-yl)-1-oxopropan-2-yl]piperidine-1-carboxylate (**7g**)

Compound **7g** was obtained from **5** (0.27 g, 1 mmol), morpholine (0.09 mL, 1.1 mmol), and DBU (0.17 mL, 1.15 mmol). The reaction was stirred at 60 °C for 4 h. The obtained residue was purified via flash column chromatography (eluent methylene chloride/methanol, *v*/*v*, 100:2) to give **7g** (0.306 g, 86%) as a colorless liquid. ^1^H-NMR (400 MHz, CDCl_3_): *δ*_H_ ppm 1.14–1.27 (m, 2H, Pip), 1.43 (s, 9H, C(CH_3_)_3_), 1.48–1.51 (m, 1H, Pip), 1.64–1.73 (m, 2H, Pip), 2.30–2.35 (m, 2H, Mor), 2.42–2.54 (m, 4H, Mor CH_2_, Mor-CH_2_), 2.61–2.71 (m, 3H, Pip CH_2_, CHCOOCH_3_), 3.59–3.66 (m, 4H, Mor), 3.67 (s, 3H, OCH_3_), 4.09 (s, 2H, Pip). ^13^C-NMR (101 MHz, CDCl_3_): *δ*_C_ ppm 28.6 (COOC(CH_3_)_3_), 30.0 (Pip CH_2_), 30.1 (Pip C-4), 37.6 (Pip CH_2_), 43.8 (Pip 2 × CH_2_), 48.9 (CHCOOCH_3_), 51.6 (OCH_3_), 53.7 (Mor 2 × CH_2_), 58.7 (Mor-CH_2_), 67.0 (Mor 2 × CH_2_), 79.6 (COOC(CH_3_)_3_), 154.9 (COOC(CH_3_)_3_), 175.0 (COOCH_3_). ^15^N-NMR (71 MHz, CDCl_3_): *δ*_N_ ppm −337.6 (*N*-4), *N*-Boc was not found. IR (FT-IR, *ν*_max_, cm^−1^): 2947, 2852 (CH_aliph_), 1735 (C=O), 1689 (C=O), 1422, 1157, 1116 (C=C, C–N, C–O–C). HRMS (ESI^+^) C_18_H_33_N_2_O_5_ ([M + H]^+^) calcd. 357.2384, found 357.2384.

*Tert*-butyl 4-[3-(1,3-dihydro-2*H*-isoindol-2-yl)-1-methoxy-1-oxopropan-2-yl]piperidine-1-carboxylate (**7h**)

Compound **7h** was obtained from **5** (0.27 g, 1 mmol), isoindoline hydrochloride (0.17 g, 1.1 mmol), and DBU (0.17 mL, 1.15 mmol). The reaction was stirred at 60 °C for 12 h. The obtained residue was purified via flash column chromatography (eluent methylene chloride/methanol, *v*/*v*, 100:2) to give **7h** (0.256 g, 66%) as a dark brown liquid. ^1^H-NMR (400 MHz, CDCl_3_): *δ*_H_ ppm 1.20–1.33 (m, 2H, Pip), 1.45 (s, 9H, C(CH_3_)_3_), 1.54–1.60 (m, 1H, Pip), 1.75–1.78 (m, 2H, Pip), 2.52–2.58 (m, 1H, CHCOOCH_3_), 2.63–2.69 (m, 2H, Pip), 2.80–2.84 (m, 1H, *i*-Ind-CH_2_), 3.06–3.12 (m, 1H, *i*-Ind-CH_2_), 3.68 (s, 3H, OCH_3_), 3.86 (d, *J* = 11.5 Hz, 2H, *i*-Ind 1,3-H), 3.98 (d, *J* = 11.5 Hz, 2H, *i*-Ind 1,3-H), 4.11 (s, 2H, Pip), 7.17 (s, 4H, *i*-Ind CH). ^13^C-NMR (101 MHz, CDCl_3_): *δ*_C_ ppm 28.6 (COOC(CH_3_)_3_), 29.8 (Pip C-4), 30.4 (Pip CH_2_), 37.4 (Pip CH_2_), 43.9 (Pip 2 × CH_2_), 51.0 (CHCOOCH_3_), 51.6 (OCH_3_), 56.1 (*i*-Ind-CH_2_), 59.4 (*i*-Ind C-1,3), 79.5 (COOC(CH_3_)_3_), 122.3 (*i*-Ind 2 × CH), 126.8 (*i*-Ind 2 × CH), 140.1 (*i*-Ind C-3a,7a), 154.8 (COOC(CH_3_)_3_), 174.9 (COOCH_3_). ^15^N-NMR (71 MHz, CDCl_3_): *δ*_N_ ppm −339.5 (*N*-2), −292.8 (*N*-Boc). IR (FT-IR, *ν*_max_, cm^−1^): 2936, 2852, 2765 (CH_aliph_), 1734 (C=O), 1687 (C=O), 1422, 1159 (C=C, C–N, C–O–C). HRMS (ESI^+^) C_22_H_33_N_2_O_4_ ([M + H]^+^) calcd. 389.2435, found 389.2435.

*Tert*-butyl 4-[1-methoxy-1-oxo-3-(1*H*-pyrazol-1-yl)propan-2-yl]piperidine-1-carboxylate (**7i**)

Compound **7i** was obtained from **5** (0.27 g, 1 mmol), 1*H*-pyrazole (0.08 g, 1.1 mmol), and DBU (0.17 mL, 1.15 mmol). The reaction was stirred at 60 °C for 18 h. The obtained residue was purified via flash column chromatography (eluent methylene chloride/methanol, *v*/*v*, 100:4) to give **7i** (0.273 g, 81%) as a colorless liquid. ^1^H-NMR (400 MHz, CDCl_3_): *δ*_H_ ppm 1.24–1.35 (m, 2H, Pip), 1.44 (s, 9H, C(CH_3_)_3_), 1.57–1.61 (m, 1H, Pip), 1.70–1.76 (m, 2H, Pip), 2.62–2.67 (m, 2H, Pip), 2.95–3.00 (m, 1H, CHCOOCH_3_), 3.60 (s, 3H, OCH_3_), 4.12 (s, 2H, Pip), 4.28–4.33 (m, 1H, Pyr-CH_2_), 4.37–4.42 (m, 1H, Pyr-CH_2_), 6.18 (s, 1H, Pyr 4-H), 7.33 (s, 1H, Pyr 5-H), 7.48 (s, 1H, Pyr 3-H). ^13^C-NMR (101 MHz, CDCl_3_): *δ*_C_ ppm 28.5 (COOC(CH_3_)_3_), 29.4 (Pip C-4), 29.9 (Pip CH_2_), 37.2 (Pip CH_2_), 43.9 (Pip 2 × CH_2_), 51.4 (Pyr-CH_2_), 51.8 (CHCOOCH_3_), 51.9 (OCH_3_), 79.7 (COOC(CH_3_)_3_), 105.6 (Pyr C-4), 130.0 (Pyr C-5), 140.0 (Pyr C-3), 154.8 (COOC(CH_3_)_3_), 173.6 (COOCH_3_). ^15^N-NMR (71 MHz, CDCl_3_): *δ*_N_ ppm −174.6 (*N*-1), −78.8 (*N*-2), *N*-Boc was not found. IR (FT-IR, *ν*_max_, cm^−1^): 2936, 2866 (CH_aliph_), 1724 (C=O), 1674 (C=O), 1428, 1155, 1131 (C=C, C–N, C–O–C). HRMS (ESI^+^) C_17_H_27_N_3_NaO_4_ ([M + Na]^+^) calcd. 360.1894, found 360.1894.

*Tert*-butyl 4-[1-methoxy-3-(4-methyl-1*H*-pyrazol-1-yl)-1-oxopropan-2-yl]piperidine-1-carboxylate (**7j**)

Compound **7j** was obtained from **5** (0.27 g, 1 mmol), 4-methyl-1*H*-pyrazole (0.09 mL, 1.1 mmol), and DBU (0.17 mL, 1.15 mmol). The reaction was stirred at 60 °C for 14 h. The obtained residue was purified via flash column chromatography (eluent methylene chloride/methanol, *v*/*v*, 100:2) to give **7j** (0.211 g, 60%) as a colorless liquid. ^1^H-NMR (400 MHz, CDCl_3_): *δ*_H_ ppm 1.26–1.32 (m, 2H, Pip), 1.45 (s, 9H, C(CH_3_)_3_), 1.58–1.61 (m, 1H, Pip), 1.71–1.74 (m, 2H, Pip), 2.04 (s, 3H, Pyr-CH_3_), 2.63–2.68 (m, 2H, Pip), 2.94–2.98 (m, 1H, CHCOOCH_3_), 3.62 (s, 3H, OCH_3_), 4.13 (s, 2H, Pip), 4.21–4.35 (m, 2H, Pyr-CH_2_), 7.11 (s, 1H, Pyr 5-H), 7.29 (s, 1H, Pyr 3-H). ^13^C-NMR (101 MHz, CDCl_3_): *δ*_C_ ppm 8.9 (Pyr-CH_3_), 28.5 (COOC(CH_3_)_3_), 29.4 (Pip CH_2_), 29.8 (Pip CH_2_), 37.1 (Pip CH_2_), 43.9 (Pip 2 × CH_2_), 51.2 (Pyr-CH_2_), 51.7 (CHCOOCH_3_), 51.9 (OCH_3_), 79.6 (COOC(CH_3_)_3_), 116.0 (Pyr C-4), 128.7 (Pyr C-5), 140.3 (Pyr C-3), 154.8 (COOC(CH_3_)_3_), 173.6 (COOCH_3_). ^15^N-NMR (71 MHz, CDCl_3_): *δ*_N_ ppm −175.9 (*N*-1), −78.3 (*N*-2), *N*-Boc was not found. IR (FT-IR, *ν*_max_, cm^−1^): 2932, 2857 (CH_aliph_), 1732 (C=O), 1687 (C=O), 1423, 1160 (C=C, C–N, C–O–C). HRMS (ESI^+^) C_18_H_29_N_3_NaO_4_ ([M + Na]^+^) calcd. 374.2050, found 374.2050.

*Tert*-butyl 4-[3-(4-chloro-1*H*-pyrazol-1-yl)-1-methoxy-1-oxopropan-2-yl]piperidine-1-carboxylate (**7k**)

Compound **7k** was obtained from **5** (0.27 g, 1 mmol), 4-chloro-1*H*-pyrazole (0.11 g, 1.1 mmol), and DBU (0.17 mL, 1.15 mmol). The reaction was stirred at 60 °C for 16 h. The obtained residue was purified via flash column chromatography (eluent methylene chloride/methanol, *v*/*v*, 100:2) to give **7k** (0.212 g, 57%) as a colorless liquid. ^1^H-NMR (400 MHz, CDCl_3_): *δ*_H_ ppm 1.24–1.32 (m, 2H, Pip), 1.44 (s, 9H, C(CH_3_)_3_), 1.56–1.59 (m, 1H, Pip), 1.68–1.78 (m, 2H, Pip), 2.62–2.68 (m, 2H, Pip), 2.91–2.96 (m, 1H, CHCOOCH_3_), 3.63 (s, 3H, OCH_3_), 4.13 (s, 2H, Pip), 4.20–4.25 (m, 1H, Pyr-CH_2_), 4.32–4.37 (m, 1H, Pyr-CH_2_), 7.34 (s, 1H, Pyr 5-H), 7.40 (s, 1H, Pyr 3-H). ^13^C-NMR (101 MHz, CDCl_3_): *δ*_C_ ppm 28.5 (COOC(CH_3_)_3_), 29.4 (Pip CH_2_), 29.8 (Pip CH_2_), 37.2 (Pip CH_2_), 43.9 (Pip 2 × CH_2_), 51.5 (CHCOOCH_3_), 52.0 (Pyr-CH_2_), 52.1 (OCH_3_), 79.8 (COOC(CH_3_)_3_), 109.9 (Pyr C-4), 128.1 (Pyr C-5), 138.4 (Pyr C-3), 154.8 (COOC(CH_3_)_3_), 173.3 (COOCH_3_). ^15^N-NMR (71 MHz, CDCl_3_): *δ*_N_ ppm −177.1 (*N*-1), −78.5 (*N*-2), *N*-Boc was not found. IR (FT-IR, *ν*_max_, cm^−1^): 2945, 2873 (CH_aliph_), 1725 (C=O), 1688 (C=O), 1424, 1164 (C=C, C–N, C–O–C). HRMS (ESI^+^) C_17_H_26_ClN_3_NaO_4_ ([M + Na]^+^) calcd. 394.1504, found 394.1504.

*Tert*-butyl 4-[3-(4-bromo-1*H*-pyrazol-1-yl)-1-methoxy-1-oxopropan-2-yl]piperidine-1-carboxylate (**7l**)

Compound **7l** was obtained from **5** (0.27 g, 1 mmol), 4-bromopyrazole (0.16 g, 1.1 mmol), and DBU (0.17 mL, 1.15 mmol). The reaction was stirred at 60 °C for 12 h. The obtained residue was purified via flash column chromatography (eluent methylene chloride/methanol, *v*/*v*, 100:5) to give **7l** (0.354 g, 85%) as a colorless liquid. ^1^H-NMR (400 MHz, CDCl_3_): *δ*_H_ ppm 1.25–1.34 (m, 2H, Pip), 1.43 (s, 9H, C(CH_3_)_3_), 1.55–1.59 (m, 1H, Pip), 1.68–1.76 (m, 2H, Pip), 2.61–2.66 (m, 2H, Pip), 2.90–2.96 (m, 1H, CHCOOCH_3_), 3.62 (s, 3H, OCH_3_), 4.12 (s, 2H, Pip), 4.21–4.26 (m, 1H, Br-Pyr-CH_2_), 4.33–4.39 (m, 1H, Br-Pyr-CH_2_), 7.36 (s, 1H, Pyr 5-H), 7.42 (s, 1H, Pyr 3-H). ^13^C-NMR (101 MHz, CDCl_3_): *δ*_C_ ppm 28.5 (COOC(CH_3_)_3_), 29.4 (Pip C-4), 29.8 (Pip CH_2_), 37.1 (Pip CH_2_), 43.8 (Pip 2 × CH_2_), 51.4 (CHCOOCH_3_), 51.9 (Br-Pyr-CH_2_), 52.1 (OCH_3_), 79.7 (COOC(CH_3_)_3_), 93.0 (Pyr C-4), 130.2 (Pyr C-5), 140.5 (Pyr C-3), 154.7 (COOC(CH_3_)_3_), 173.2 (COOCH_3_). ^15^N-NMR (71 MHz, CDCl_3_): *δ*_N_ ppm −172.6 (*N*-1), −75.0 (*N*-2), *N*-Boc was not found. IR (FT-IR, *ν*_max_, cm^−1^): 2934, 2853 (CH_aliph_), 1731 (C=O), 1686 (C=O), 1422, 1156 (C=C, C–N, C–O–C). HRMS (ESI^+^) C_17_H_26_BrN_3_NaO_4_ ([M + Na]^+^) calcd. 438.0999, found 438.0999.

*Tert*-butyl 4-{1-methoxy-1-oxo-3-[3-(trifluoromethyl)-1*H*-pyrazol-1-yl]propan-2-yl}piperidine-1-carboxylate (**7m**)

Compound **7m** was obtained from **5** (0.27 g, 1 mmol), 3-trifluoromethylpyrazole (0.15 g, 1.1 mmol), and DBU (0.17 mL, 1.15 mmol). The reaction was stirred at 60 °C for 12 h. The obtained residue was purified via flash column chromatography (eluent hexane/ethyl acetate, *v*/*v*, 4:1) to give **7m** (0.32 g, 79%) as a colorless liquid. ^1^H-NMR (400 MHz, CDCl_3_): *δ*_H_ ppm 1.24–1.33 (m, 2H, Pip), 1.44 (s, 9H, C(CH_3_)_3_), 1.57–1.60 (m, 1H, Pip), 1.69–1.81 (m, 2H, Pip), 2.62–2.68 (m, 2H, Pip), 2.96–3.01 (m, 1H, CHCOOCH_3_), 3.61 (s, 3H, OCH_3_), 4.14 (s, 2H, Pip), 4.30–4.34 (m, 1H, CF_3_-Pyr-CH_2_), 4.41–4.47 (m, 1H, CF_3_-Pyr-CH_2_), 6.45 (s, 1H, Pyr 4-H), 7.40 (s, 1H, Pyr 5-H). ^13^C-NMR (101 MHz, CDCl_3_): *δ*_C_ ppm 28.5 (COOC(CH_3_)_3_), 29.4 (Pip CH_2_), 29.8 (Pip CH_2_), 37.3 (Pip CH_2_), 43.8 (Pip 2 × CH_2_), 51.5 (CHCOOCH_3_), 52.1 (OCH_3_), 52.1 (CF_3_-Pyr-CH_2_), 79.8 (COOC(CH_3_)_3_), 104.4 (Pyr C-4), 121.8 (q, *J* = 268.5 Hz, CF_3_), 131.7 (Pyr C-5), 143.0 (q, *J* = 38.3 Hz, Pyr C-3), 154.8 (COOC(CH_3_)_3_), 173.2 (COOCH_3_). ^15^N-NMR (71 MHz, CDCl_3_): *δ*_N_ ppm −174.3 (*N*-1), −74.7 (*N*-2), *N*-Boc was not found. ^19^F-NMR (376 MHz, CDCl_3_): *δ*_F_ ppm −62.0 (s, CF_3_). IR (FT-IR, *ν*_max_, cm^−1^): 2918, 2856 (CH_aliph_), 1733 (C=O), 1687 (C=O), 1407, 1121 (C=C, C–N, C–O–C). HRMS (ESI^+^) C_18_H_26_F_3_N_3_NaO_4_ ([M + Na]^+^) calcd. 428.1768, found 428.1768.

*Tert*-butyl 4-[3-(1*H*-indazol-1-yl)-1-methoxy-1-oxopropan-2-yl]piperidine-1-carboxylate (**7n**)

Compound **7n** was obtained from **5** (0.27 g, 1 mmol), 1*H*-indazole (0.13 g, 1.1 mmol), and DBU (0.17 mL, 1.15 mmol). The reaction was stirred at 60 °C for 18 h. The obtained residue was purified via flash column chromatography (eluent methylene chloride/methanol, *v*/*v*, 100:4) to give **7n** (0.252 g, 65%) as a yellowish liquid. ^1^H-NMR (400 MHz, CDCl_3_): *δ*_H_ ppm 1.31–1.39 (m, 2H, Pip), 1.45 (s, 9H, C(CH_3_)_3_), 1.63–1.66 (m, 1H, Pip), 1.73–1.81 (m, 2H, Pip), 2.63–2.69 (m, 2H, Pip), 3.17–3.22 (m, 1H, CHCOOCH_3_), 3.57 (s, 3H, OCH_3_), 4.15 (s, 2H, Pip), 4.54–4.59 (m, 1H, Ind-CH_2_), 4.67–4.72 (m, 1H, Ind-CH_2_), 7.06 (t, *J* = 7.5 Hz, 1H, Ind), 7.26 (t, *J* = 7.5 Hz, 1H, Ind), 7.61 (d, *J* = 8.4 Hz, 1H, Ind), 7.67 (d, *J* = 8.4 Hz, 1H, Ind), 7.89 (s, 1H, Ind 3-H). ^13^C-NMR (101 MHz, CDCl_3_): *δ*_C_ ppm 28.5 (COOC(CH_3_)_3_), 29.4 (Pip C-4), 29.9 (Pip CH_2_), 37.5 (Pip CH_2_), 43.9 (Pip 2 × CH_2_), 51.8 (CHCOOCH_3_), 52.0 (OCH_3_), 52.9 (Ind-CH_2_), 79.7 (COOC(CH_3_)_3_), 117.5 (Ind CH), 120.3 (Ind CH), 121.7 (Ind C-3a), 121.8 (Ind CH), 123.9 (Ind CH), 126.2 (Ind CH), 149.3 (Ind C-7a), 154.8 (COOC(CH_3_)_3_), 173.3 (COOCH_3_). ^15^N-NMR (71 MHz, CDCl_3_): *δ*_N_ ppm −160.1 (*N*-1), −99.9 (*N*-2), *N*-Boc was not found. IR (FT-IR, *ν*_max_, cm^−1^): 2932, 2885 (CH_aliph_), 1732 (C=O), 1686 (C=O), 1422, 1161 (C=C, C–N, C–O–C). HRMS (ESI^+^) C_21_H_30_N_3_O_4_ ([M + H]^+^) calcd. 388.2231, found 388.2231.

*Tert*-butyl 4-[3-(1*H*-imidazol-1-yl)-1-methoxy-1-oxopropan-2-yl]piperidine-1-carboxylate (**7o**)

Compound **7o** was obtained from **5** (0.27 g, 1 mmol), imidazole (0.08 g, 1.1 mmol), and DBU (0.17 mL, 1.15 mmol). The reaction was stirred at 60 °C for 14 h. The obtained residue was purified via flash column chromatography (eluent methylene chloride/methanol, *v*/*v*, 100:4) to give **7o** (0.219 g, 65%) as a yellowish liquid. ^1^H-NMR (400 MHz, CDCl_3_): *δ*_H_ ppm 1.21–1.32 (m, 2H, Pip), 1.41 (s, 9H, C(CH_3_)_3_), 1.52–1.55 (m, 1H, Pip), 1.66–1.76 (m, 2H, Pip), 2.56–2.69 (m, 3H, Pip CH_2_, CHCOOCH_3_), 3.57 (s, 3H, OCH_3_), 4.02–4.26 (m, 4H, Pip CH_2_, Imid-CH_2_), 6.81 (s, 1H, Imid 2-H), 6.98 (s, 1H, Imid 4-H), 7.38 (s, 1H, Imid 5-H). ^13^C-NMR (101 MHz, CDCl_3_): *δ*_C_ ppm 28.4 (COOC(CH_3_)_3_), 29.4 (Pip C-4), 29.8 (Pip CH_2_), 37.2 (Pip CH_2_), 43.7 (Pip 2 × CH_2_), 46.3 (Imid-CH_2_), 52.1 (OCH_3_), 52.9 (CHCOOCH_3_), 79.7 (COOC(CH_3_)_3_), 119.0 (Imid C-2), 129.7 (Imid C-4), 137.5 (Imid C-5), 154.6 (COOC(CH_3_)_3_), 173.0 (COOCH_3_). ^15^N-NMR (71 MHz, CDCl_3_): *δ*_N_ ppm −293.8 (*N*-Boc), −213.2 (*N*-1), −124.3 (*N*-3). IR (FT-IR, *ν*_max_, cm^−1^): 2974, 2857 (CH_aliph_), 1731 (C=O), 1683 (C=O), 1423, 1162 (C=C, C–N, C–O–C). HRMS (ESI^+^) C_17_H_28_N_3_O_4_ ([M + H]^+^) calcd. 338.2074, found 338.2074.

*Tert*-butyl 4-[1-methoxy-1-oxo-3-(2-propyl-1*H*-imidazol-1-yl)propan-2-yl]piperidine-1-carboxylate (**7p**)

Compound **7p** was obtained from **5** (0.27 g, 1 mmol), 2-propylimidazole (0.12 g, 1.1 mmol), and DBU (0.17 mL, 1.15 mmol). The reaction was stirred at 60 °C for 18 h. The obtained residue was purified via flash column chromatography (eluent methylene chloride/methanol, *v*/*v*, 100:2) to give **7p** (0.133 g, 35%) as a yellowish liquid. ^1^H-NMR (400 MHz, CDCl_3_): *δ*_H_ ppm 0.99 (t, *J* = 7.4 Hz, 3H, CH_2_CH_2_CH_3_), 1.24–1.34 (m, 2H, Pip), 1.44 (s, 9H, C(CH_3_)_3_), 1.55–1.58 (m, 1H, Pip), 1.72–1.82 (m, 4H, Pip CH_2_, CH_2_CH_2_CH_3_), 2.57–2.69 (m, 5H, Pip CH_2_, CH_2_CH_2_CH_3_, CHCOOCH_3_), 3.59 (s, 3H, OCH_3_), 3.96–4.01 (m, 1H, Pim-CH_2_), 4.13–4.19 (m, 3H, Pip CH_2_, Pim-CH_2_), 6.74 (s, 1H, Pim 5-H), 6.91 (s, 1H, Pim 4-H). ^13^C-NMR (101 MHz, CDCl_3_): *δ*_C_ ppm 14.1 (CH_2_CH_2_CH_3_), 21.3 (CH_2_CH_2_CH_3_), 28.5 (COOC(CH_3_)_3_), 28.6 (CH_2_CH_2_CH_3_), 29.6 (Pip CH_2_), 29.8 (Pip CH_2_), 37.4 (Pip CH_2_), 43.8 (Pip 2 × CH_2_), 45.2 (Pim-CH_2_), 52.1 (OCH_3_), 52.5 (CHCOOCH_3_), 79.8 (COOC(CH_3_)_3_), 119.1 (Pim C-5), 127.3 (Pim C-4), 148.2 (Pim C-2), 154.7 (COOC(CH_3_)_3_), 173.2 (COOCH_3_). ^15^N-NMR (71 MHz, CDCl_3_): *δ*_N_ ppm −217.2 (*N*-1), −131.5 (*N*-3), *N*-Boc was not found. IR (FT-IR, *ν*_max_, cm^−1^): 2960, 2871 (CH_aliph_), 1733 (C=O), 1686 (C=O), 1423, 1163 (C=C, C–N, C–O–C). HRMS (ESI^+^) C_20_H_34_N_3_O_4_ ([M + H]^+^) calcd. 380.2544, found 380.2544.

*Tert*-butyl 4-[3-(1*H*-benzimidazol-1-yl)-1-methoxy-1-oxopropan-2-yl]piperidine-1-carboxylate (**7r**)

Compound **7r** was obtained from **5** (0.27 g, 1 mmol), benzimidazole (0.13 g, 1.1 mmol), and DBU (0.17 mL, 1.15 mmol). The reaction was stirred at 60 °C for 8 h. The obtained residue was purified via flash column chromatography (eluent methylene chloride/methanol, *v*/*v*, 100:2) to give **7r** (0.344 g, 89%) as a colorless liquid. ^1^H-NMR (400 MHz, CDCl_3_): *δ*_H_ ppm 1.25–1.41 (m, 2H, Pip), 1.46 (s, 9H, C(CH_3_)_3_), 1.61–1.67 (m, 1H, Pip), 1.78–1.87 (m, 2H, Pip), 2.67–2.73 (m, 2H, Pip), 2.84–2.90 (m, 1H, CHCOOCH_3_), 3.54 (s, 3H, OCH_3_), 4.19 (s, 2H, Pip), 4.27–4.32 (m, 1H, Bim-CH_2_), 4.50–4.57 (m, 1H, Bim-CH_2_), 7.28–7.35 (m, 3H, Bim CH), 7.78–7.80 (m, 1H, Bim CH), 7.85 (s, 1H, Bim CH). ^13^C-NMR (101 MHz, CDCl_3_): *δ*_C_ ppm 28.5 (COOC(CH_3_)_3_), 29.6 (Pip CH_2_), 29.8 (Pip CH_2_), 37.5 (Pip CH_2_), 43.8 (Pip 2 × CH_2_), 44.4 (Bim-CH_2_), 51.3 (CHCOOCH_3_), 52.2 (OCH_3_), 79.9 (COOC(CH_3_)_3_), 109.3 (Bim CH), 120.7 (Bim CH), 122.5 (Bim CH), 123.3 (Bim CH), 133.5 (Bim C-7a), 143.4 (Bim C-2), 143.9 (Bim C-3a), 154.7 (COOC(CH_3_)_3_), 173.2 (COOCH_3_). ^15^N-NMR (71 MHz, CDCl_3_): *δ*_N_ ppm −228.9 (*N*-1), −137.4 (*N*-3), *N*-Boc was not found. IR (FT-IR, *ν*_max_, cm^−1^): 2934, 2860 (CH_aliph_), 1732 (C=O), 1680 (C=O), 1429, 1135 (C=C, C–N, C–O–C). HRMS (ESI^+^) C_21_H_30_N_3_O_4_ ([M + H]^+^) calcd. 388.2231, found 388.2230.

#### 3.2.4. General Procedure for Compound **9**

Corresponding ketone (1 mmol), 2,2-dimethyl-1,3-dioxane-4,6-dione **2** (1 mmol) and diethyl 1,4-dihydro-2,6-dimethyl-3,5-pyridinedicarboxylate (1 mmol) were dissolved in DMSO (1 mL). After 10 min, *L*-proline (0.2 mmol) was added, and the resulting mixture was stirred at room temperature for 18 h. The residue was dissolved in ethyl acetate and washed with brine. The organic layer was dried with anhydrous sodium sulfate, filtered, and then concentrated under reduced pressure. The resulting mixture was purified via flash column chromatography to give the corresponding product **9**.

*Tert*-butyl 3-(2,2-dimethyl-4,6-dioxo-1,3-dioxan-5-yl)azetidine-1-carboxylate (**9**)

*Tert*-butyl 3-oxoazetidine-1-carboxylate (**8**) (0.172 g, 1 mmol), 2,2-dimethyl-1,3-dioxane-4,6-dione **2** (0.14 g, 1 mmol) and diethyl 1,4-dihydro-2,6-dimethyl-3,5-pyridinedicarboxylate (0.25 g, 1 mmol) were dissolved in DMSO (1 mL). After 10 min, *L*-proline (0.02 g, 0.2 mmol) was added, and the resulting mixture was stirred at room temperature for 18 h. The obtained residue was diluted with ethyl acetate and washed with brine (2 × 15 mL). The organic layer was dried with anhydrous sodium sulfate, filtered, and then concentrated under reduced pressure. The resulting mixture was purified via flash column chromatography (eluent *n*-hexane/ethyl acetate, *v*/*v*, 4:1) to give the product **9** (0.224 g, 75%) as a white amorphous solid, mp 140–141 °C. ^1^H-NMR (400 MHz, CDCl_3_): *δ*_H_ ppm 1.43 (s, 9H, C(CH_3_)_3_), 1.77 (s, 3H, CH_3_), 1.87 (s, 3H, CH_3_), 3.07 (h, *J* = 7.5 Hz, 1H, Az 3-H), 3.82–3.85 (m, 1H, CH), 3.89–3.93 (m, 2H, Az 2,4-H), 4.25–4.29 (m, 2H, Az 2,4-H). ^13^C-NMR (101 MHz, CDCl_3_): *δ*_C_ ppm 26.5 (CH_3_), 27.4 (Az C-3), 28.5 (C(CH_3_)_3_), 28.7 (COOC(CH_3_)_3_), 49.8 (CH), 54.0 (Az 2 × CH_2_), 79.8 (COOC(CH_3_)_3_), 105.7 (C) 156.1 (COOC(CH_3_)_3_), 164.0 (2 × C=O). ^15^N-NMR (71 MHz, CDCl_3_): *δ*_N_ ppm −308.0 (*N*-Boc). IR (FT-IR, *ν*_max_, cm^−1^): 2980, 2887 (CH_aliph_), 1791 (C=O), 1743 (C=O), 1696 (C=O), 1386, 1133 (C=C, C–N, C–O–C). HRMS (ESI^+^) C_14_H_21_NNaO_6_ ([M + Na]^+^) calcd. 322.1261, found 322.1261.

#### 3.2.5. General Procedure for Compound **10**

A 50-mL round-bottomed flask was charged under nitrogen atmosphere with compound **9** (1 mmol) and *N*,*N*-dimethylmethyleneiminium iodide (2 mmol). The solids were dissolved in THF and MeOH (1:1). The reaction mixture was stirred at 70 °C for 18 h. Upon cooling of the mixture to room temperature, the solvents were removed in vacuo, and the residue was taken up in ethyl acetate, washed with sat. NaHCO_3_ soln, 10% aq. KHSO_4_ and brine. The organic layer was dried with anhydrous sodium sulfate, filtered, and then concentrated under reduced pressure. The resulting mixture was purified via flash column chromatography to give the corresponding product **10**.

*Tert*-butyl 3-(3-methoxy-3-oxoprop-1-en-2-yl)azetidine-1-carboxylate (**10**)

A 50-mL round-bottomed flask was charged under nitrogen atmosphere with compound **9** (0.299 g, 1 mmol) and *N*,*N*-dimethylmethyleneiminium iodide (0.37 g, 2 mmol). The solids were dissolved in THF (6 mL) and MeOH (6 mL). The reaction mixture was stirred at 70 °C for 18 h. Upon cooling of the mixture to room temperature, the solvents were removed in vacuo, and the yellow residue was taken up in ethyl acetate (15 mL), washed with sat. NaHCO_3_ soln (15 mL), 10% aq. KHSO_4_ (15 mL) and brine (15 mL). The organic layer was dried with anhydrous sodium sulfate, filtered, and then concentrated under reduced pressure. The resulting mixture was purified via flash column chromatography (eluent *n*-hexane/ethyl acetate, *v*/*v*, 4:1) to give the product **10** (0.222 g, 92%) as a colorless liquid. ^1^H-NMR (400 MHz, CDCl_3_): *δ*_H_ ppm 1.42 (s, 9H, C(CH_3_)_3_), 3.55 (p, *J* = 7.3 Hz, 1H, Az 3-H), 3.75 (s, 3H, OCH_3_), 3.82 (t, *J* = 7.6 Hz, 2H, Az 2,4-H), 4.13 (t, *J* = 8.7 Hz, 2H, Az 2,4-H), 5.68 (s, 1H, C=CH_2_), 6.35 (s, 1H, C=CH_2_). ^13^C-NMR (101 MHz, CDCl_3_): *δ*_C_ ppm 28.5 (COOC(CH_3_)_3_), 30.2 (Az C-3), 52.2 (OCH_3_), 53.9 (Az 2 × CH_2_), 79.6 (COOC(CH_3_)_3_, 124.7 (C=CH_2_), 140.1 (C=CH_2_), 156.5 (COOC(CH_3_)_3_), 166.7 (COOCH_3_). ^15^N-NMR (71 MHz, CDCl_3_): *δ*_N_ ppm −310.72 (*N*-Boc). IR (FT-IR, *ν*_max_, cm^−1^): 2979, 2888 (CH_aliph_), 1700 (C=O), 1640 (C=O), 1268, 1145 (C=C, C–N, C–O–C). HRMS (ESI^+^) C_12_H_19_NNaO_4_ ([M + Na]^+^) calcd. 264.1206, found 264.1206.

#### 3.2.6. General Procedure for Compounds **11a**–**f**

An appropriate *N*-heterocyclic compound **6** (1.1 mmol), DBU (1.15 mmol), and *tert*-butyl 3-(3-methoxy-3-oxoprop-1-en-2-yl)azetidine-1-carboxylate **10** (1 mmol) were dissolved in acetonitrile (1 mL) and stirred at 60 °C for 4–18 h. The reaction mixture was diluted with water (10 mL) and extracted with ethyl acetate (2 × 15 mL). The organic layer was dried with anhydrous sodium sulfate, filtered, and then concentrated under reduced pressure. The resulting mixtures were purified via flash column chromatography to provide products **11a**–**f**.

*Tert*-butyl 3-[1-methoxy-3-(morpholin-4-yl)-1-oxopropan-2-yl]azetidine-1-carboxylate (**11a**)

Compound **11a** was obtained from **10** (0.241 g, 1 mmol), morpholine (0.09 mL, 1.1 mmol), and DBU (0.17 mL, 1.15 mmol). The reaction was stirred at 60 °C for 4 h. The obtained residue was purified via flash column chromatography (eluent methylene chloride/methanol, *v*/*v*, 100:2) to give **11a** (0.266 g, 81%) as a colorless liquid. ^1^H-NMR (400 MHz, CDCl_3_): *δ*_H_ ppm 1.42 (s, 9H, C(CH_3_)_3_), 2.31–2.36 (m, 1H, Mor-CH_2_), 2.39–2.41 (m, 2H, Mor CH_2_), 2.46–2.53 (m, 2H, Mor CH_2_), 2.63–2.76 (m, 2H, Mor-CH_2_, Az 3-H), 2.84–2.90 (m, 1H, CHCOOCH_3_), 3.62–3.70 (m, 9H, OCH_3_, Az CH_2_, Mor 2 × CH_2_), 3.94–4.04 (m, 2H, Az CH_2_). ^13^C-NMR (101 MHz, CDCl_3_): *δ*_C_ ppm 28.5 (COOC(CH_3_)_3_), 30.1 (Az C-3), 47.1 (CHCOOCH_3_), 52.0 (OCH_3_), 53.1 (Az 2 × CH_2_), 53.8 (Mor 2 × CH_2_), 58.8 (Mor-CH_2_), 66.9 (Mor 2 × CH_2_), 79.7 (COOC(CH_3_)_3_), 156.3 (COOC(CH_3_)_3_), 173.9 (COOCH_3_). ^15^N-NMR (71 MHz, CDCl_3_): *δ*_N_ ppm −339.7 (*N*-4), −309.3 (*N*-Boc). IR (FT-IR, *ν*_max_, cm^−1^): 2980, 2887 (CH_aliph_), 1733 (C=O), 1697 (C=O), 1393, 1366, 1141, 1115 (C=C, C–N, C–O–C). HRMS (ESI^+^) C_16_H_29_N_2_O_5_ ([M + H]^+^) calcd. 329.2071, found 329.2071.

*Tert*-butyl 3-[3-(1,3-dihydro-2*H*-isoindol-2-yl)-1-methoxy-1-oxopropan-2-yl]azetidine-1-carboxylate (**11b**)

Compound **11b** was obtained from **10** (0.241 g, 1 mmol), isoindoline hydrochloride (0.17 g, 1.1 mmol), and DBU (0.17 mL, 1.15 mmol). The reaction was stirred at 60 °C for 12 h. The obtained residue was purified via flash column chromatography (eluent methylene chloride/methanol, *v*/*v*, 100:2) to give **11b** (0.22 g, 61%) as a dark brown liquid. ^1^H-NMR (400 MHz, CDCl_3_): *δ*_H_ ppm 1.43 (s, 9H, C(CH_3_)_3_), 2.71–2.84 (m, 2H, *i*-Ind-CH_2_, Az 3-H), 2.95–3.00 (m, 1H, CHCOOCH_3_), 3.15–3.21 (m, 1H, *i*-Ind-CH_2_), 3.68–3.77 (m, 5H, OCH_3_, Az CH_2_), 3.97–4.12 (m, 6H, *i*-Ind 1,3-CH_2_, Az CH_2_), 7.20 (s, 4H, *i*-Ind CH). ^13^C-NMR (101 MHz, CDCl_3_): *δ*_C_ ppm 28.5 (COOC(CH_3_)_3_), 30.0 (Az C-3), 48.6 (CHCOOCH_3_), 52.3 (OCH_3_), 53.2 (Az 2 × CH_2_), 55.6 (*i*-Ind-CH_2_), 59.3 (*i*-Ind C-1,3), 79.8 (COOC(CH_3_)_3_), 122.5 (*i*-Ind 2 × CH), 127.4 (*i*-Ind 2 × CH), 138.6 (*i*-Ind C-3a,7a), 156.4 (COOC(CH_3_)_3_), 173.7 (COOCH_3_). ^15^N-NMR (71 MHz, CDCl_3_): *δ*_N_ ppm −335.8 (*N*-2), −310.3 (*N*-Boc). IR (FT-IR, *ν*_max_, cm^−1^): 2980, 2886 (CH_aliph_), 1732 (C=O), 1695 (C=O), 1392, 1365, 1146 (C=C, C–N, C–O–C). HRMS (ESI^+^) C_20_H_29_N_2_O_4_ ([M + H]^+^) calcd. 361.2122, found 361.2122.

*Tert*-butyl 3-[1-methoxy-1-oxo-3-(1*H*-pyrazol-1-yl)propan-2-yl]azetidine-1-carboxylate (**11c**)

Compound **11c** was obtained from **10** (0.241 g, 1 mmol), 1*H*-pyrazole (0.08 g, 1.1 mmol), and DBU (0.17 mL, 1.15 mmol). The reaction was stirred at 60 °C for 14 h. The obtained residue was purified via flash column chromatography (eluent methylene chloride/methanol, *v*/*v*, 100:2) to give **11c** (0.232 g, 75%) as a colorless liquid. ^1^H-NMR (400 MHz, CDCl_3_): *δ*_H_ ppm 1.41 (s, 9H, C(CH_3_)_3_), 2.65–2.71 (m, 1H, Az 3-H), 3.18–3.24 (m, 1H, CHCOOCH_3_), 3.48–3.54 (m, 1H, Az 2,4-H), 3.67 (s, 3H, OCH_3_), 3.69–3.73 (m, 1H, Az 2,4-H), 3.91–4.01 (m, 2H, Az 2,4-H), 4.24–4.29 (m, 1H, Pyr-CH_2_), 4.35–4.41 (m, 1H, Pyr-CH_2_), 6.21 (s, 1H, Pyr 4-H), 7.32 (s, 1H, Pyr 5-H), 7.49 (s, 1H, Pyr 3-H). ^13^C-NMR (101 MHz, CDCl_3_): *δ*_C_ ppm 28.5 (COOC(CH_3_)_3_), 28.9 (Az C-3), 49.9 (CHCOOCH_3_), 51.3 (Pyr-CH_2_), 52.4 (OCH_3_), 53.1 (Az 2 × CH_2_), 79.7 (COOC(CH_3_)_3_), 106.0 (Pyr C-4), 129.9 (Pyr C-5), 140.1 (Pyr C-3), 156.2 (COOC(CH_3_)_3_), 172.4 (COOCH_3_). ^15^N-NMR (71 MHz, CDCl_3_): *δ*_N_ ppm −310.5 (*N*-Boc), −175.9 (*N*-1), −77.6 (*N*-2). IR (FT-IR, *ν*_max_, cm^−1^): 2980, 2887 (CH_aliph_), 1733 (C=O), 1693 (C=O), 1394, 1366, 1142 (C=C, C–N, C–O–C). HRMS (ESI^+^) C_15_H_23_N_3_NaO_4_ ([M + Na]^+^) calcd. 332.1581, found 332.1582.

*Tert*-butyl 3-[3-(4-bromo-1*H*-pyrazol-1-yl)-1-methoxy-1-oxopropan-2-yl]azetidine-1-carboxylate (**11d**)

Compound **11d** was obtained from **10** (0.241 g, 1 mmol), 4-bromopyrazole (0.16 g, 1.1 mmol), and DBU (0.17 mL, 1.15 mmol). The reaction was stirred at 60 °C for 18 h. The obtained residue was purified via flash column chromatography (eluent methylene chloride/methanol, *v*/*v*, 100:2) to give **11d** (0.357 g, 92%) as a colorless liquid. ^1^H-NMR (400 MHz, CDCl_3_): *δ*_H_ ppm 1.41 (s, 9H, C(CH_3_)_3_), 2.61–2.72 (m, 1H, Az 3-H), 3.13–3.22 (m, 1H, CHCOOCH_3_), 3.55–3.62 (m, 1H, Az 2,4-H), 3.69–3.75 (m, 4H, OCH_3_, Az 2,4-H), 3.95–4.02 (m, 2H, Az 2,4-H), 4.22–4.24 (m, 1H, Pyr-CH_2_), 4.30–4.35 (m, 1H, Pyr-CH_2_), 7.36 (s, 1H, Pyr 5-H), 7.43 (s, 1H, Pyr 3-H). ^13^C-NMR (101 MHz, CDCl_3_): *δ*_C_ ppm 28.5 (COOC(CH_3_)_3_), 28.8 (Az C-3), 49.5 (CHCOOCH_3_), 51.7 (Pyr-CH_2_), 52.5 (OCH_3_), 53.1 (Az 2 × CH_2_), 79.8 (COOC(CH_3_)_3_), 93.4 (Pyr C-4), 130.2 (Pyr C-5), 140.6 (Pyr C-3), 156.2 (COOC(CH_3_)_3_), 172.1 (COOCH_3_). ^15^N-NMR (71 MHz, CDCl_3_): *δ*_N_ ppm −311.2 (*N*-Boc), −174.8 (*N*-1), −73.9 (*N*-2). IR (FT-IR, *ν*_max_, cm^−1^): 2980, 2887 (CH_aliph_), 1733 (C=O), 1691 (C=O), 1394, 1366, 1143 (C=C, C–N, C–O–C). HRMS (ESI^+^) C_15_H_23_BrN_3_O_4_ ([M + H]^+^) calcd. 388.0866, found 388.0865.

*Tert*-butyl 3-{1-methoxy-1-oxo-3-[3-(trifluoromethyl)-1*H*-pyrazol-1-yl]propan-2-yl}azetidine-1-carboxylate (**11e**)

Compound **11e** was prepared from **10** (0.241 g, 1 mmol), 3-trifluoromethylpyrazole (0.15 g, 1.1 mmol), and DBU (0.17 mL, 1.15 mmol). The reaction was stirred at 60 °C for 18 h. The obtained residue was purified via flash column chromatography (eluent methylene chloride/methanol, *v*/*v*, 100:2) to give **11e** (0.23 g, 61%) as a white amorphous solid. ^1^H-NMR (400 MHz, CDCl_3_): *δ*_H_ ppm 1.41 (s, 9H, C(CH_3_)_3_), 2.64–2.73 (m, 1H, Az 3-H), 3.20–3.25 (m, 1H, CHCOOCH_3_), 3.59–3.63 (m, 1H, Az 2,4-H), 3.67 (s, 3H, OCH_3_), 3.73–3.75 (m, 1H, Az 2,4-H), 3.96–4.02 (m, 2H, Az 2,4-H), 4.28–4.32 (m, 1H, Pyr-CH_2_), 4.37–4.43 (m, 1H, Pyr-CH_2_), 6.47 (s, 1H, Pyr 4-H), 7.41 (s, 1H, Pyr 5-H). ^13^C-NMR (101 MHz, CDCl_3_): *δ*_C_ ppm 28.4 (COOC(CH_3_)_3_), 28.9 (Az C-3), 49.5 (CHCOOCH_3_), 51.8 (Pyr-CH_2_), 52.5 (OCH_3_, Az CH_2_), 53.0 (Az CH_2_), 79.8 (COOC(CH_3_)_3_), 104.8 (Pyr C-4), 121.2 (q, *J* = 268.6 Hz, CF_3_), 131.7 (Pyr C-5), 143.2 (q, *J* = 38.2 Hz, Pyr C-3), 156.2 (COOC(CH_3_)_3_), 172.0 (COOCH_3_). ^15^N-NMR (71 MHz, CDCl_3_): *δ*_N_ ppm −310.8 (*N*-Boc), −172.2 (*N*-1), −76.6 (*N*-2). ^19^F-NMR (376 MHz, CDCl_3_): *δ*_F_ ppm −62.0 (s, CF_3_). IR (FT-IR, *ν*_max_, cm^−1^): 2980, 2888 (CH_aliph_), 1732 (C=O), 1675 (C=O), 1386, 1245, 1147 (C=C, C–N, C–O–C). HRMS (ESI^+^) C_16_H_22_F_3_N_3_NaO_4_ ([M + Na]^+^) calcd. 400.1455, found 400.1455.

*Tert*-butyl 3-[3-(1*H*-benzimidazol-1-yl)-1-methoxy-1-oxopropan-2-yl]azetidine-1-carboxylate (**11f**)

Compound **11f** was obtained from **10** (0.241 g, 1 mmol), benzimidazole (0.13 g, 1.1 mmol), and DBU (0.17 mL, 1.15 mmol). The reaction was stirred at 60 °C for 8 h. The obtained residue was purified via flash column chromatography (eluent methylene chloride/methanol, *v*/*v*, 100:2) to give **11f** (0.316 g, 88%) as a colorless liquid. ^1^H-NMR (400 MHz, CDCl_3_): *δ*_H_ ppm 1.42 (s, 9H, C(CH_3_)_3_), 2.72–2.81 (m, 1H, Az 3-H), 3.17–3.23 (m, 1H, CHCOOCH_3_), 3.62 (s, 3H, OCH_3_), 3.65–3.68 (m, 1H, Az 2,4-H), 3.75–3.79 (m, 1H, Az 2,4-H), 4.01–4.05 (m, 2H, Az 2,4-H), 4.23–4.28 (m, 1H, Bim-CH_2_), 4.45–4.51 (m, 1H, Bim-CH_2_), 7.29–7.35 (m, 3H, Bim), 7.80–7.82 (m, 1H, Bim), 7.93 (s, 1H, Bim 2-H). ^13^C-NMR (101 MHz, CDCl_3_): *δ*_C_ ppm 28.5 (COOC(CH_3_)_3_), 29.1 (Az C-3), 44.5 (Bim-CH_2_), 49.4 (CHCOOCH_3_), 52.7 (OCH_3_, Az CH_2_), 53.3 (Az CH_2_), 80.1 (COOC(CH_3_)_3_), 109.3 (Bim CH), 120.5 (Bim CH), 123.0 (Bim CH), 123.8 (Bim CH), 133.4 (Bim C), 143.1 (Bim C-2), 156.1 (COOC(CH_3_)_3_), 163.9 (Bim C), 172.0 (COOCH_3_). ^15^N-NMR (71 MHz, CDCl_3_): *δ*_N_ ppm −311.6 (*N*-Boc), −232.6 (*N*-1), −143.6 (*N*-3). IR (FT-IR, *ν*_max_, cm^−1^): 2979, 2885 (CH_aliph_), 1732 (C=O), 1686 (C=O), 1365, 1253, 1143 (C=C, C–N, C–O–C). HRMS (ESI^+^) C_19_H_26_N_3_O_4_ ([M + H]^+^) calcd. 360.1918, found 360.1918.

#### 3.2.7. General Procedure for Compound **15**

A 50 mL round-bottomed flask was charged under nitrogen atmosphere with compound **14** (1 mmol) and *N*,*N*-dimethylmethyleneiminium iodide (2 mmol). The solids were dissolved in THF and MeOH (1:1). The reaction mixture was stirred at 70 °C for 18 h. Upon cooling of the mixture to room temperature, the solvents were removed in vacuo, and the residue was taken up in ethyl acetate, washed with sat. NaHCO_3_ soln, 10% aq. KHSO_4_ and brine. The organic layer was dried with anhydrous sodium sulfate, filtered, and then concentrated under reduced pressure. The resulting mixture was purified via flash column chromatography to give the corresponding product **15**.

*Tert*-butyl 4-[2-(methoxycarbonyl)prop-2-en-1-yl]piperidine-1-carboxylate (**15**)

A 50 mL round-bottomed flask was charged under nitrogen atmosphere with compound **14** (0.341 g, 1 mmol) and *N*,*N*-dimethylmethyleneiminium iodide (0.37 g, 2 mmol). The solids were dissolved in THF (6 mL) and MeOH (6 mL). The reaction mixture was stirred at 70 °C for 18 h. Upon cooling of the mixture to room temperature, the solvents were removed in vacuo, and the yellow residue was taken up in ethyl acetate (15 mL), washed with sat. NaHCO_3_ soln (15 mL), 10% aq. KHSO_4_ (15 mL) and brine (15 mL). The organic layer was dried with anhydrous sodium sulfate, filtered, and then concentrated under reduced pressure. The resulting mixture was purified via flash column chromatography (eluent *n*-hexane/ethyl acetate, *v*/*v*, 4:1) to give the corresponding product **15** (0.26 g, 92%) as a white amorphous solid. ^1^H-NMR (400 MHz, CDCl_3_): *δ*_H_ ppm 1.00–1.10 (m, 2H, Pip CH_2_), 1.43 (s, 9H, C(CH_3_)_3_), 1.61–1.64 (m, 3H, Pip 4-H, Pip CH_2_), 2.22–2.23 (m, 2H, Pip-CH_2_), 2.64 (t, *J* = 13.0 Hz, 2H, Pip CH_2_), 3.74 (s, 3H, OCH_3_), 4.05 (s, 2H, Pip CH_2_), 5.50 (s, 1H, C=CH_2_), 6.17 (s, 1H, C=CH_2_). ^13^C-NMR (101 MHz, CDCl_3_): *δ*_C_ ppm 28.6 (COOC(CH_3_)_3_), 32.0 (Pip 2 × CH_2_), 35.0 (Pip C-4), 39.3 (Pip-CH_2_), 44.0 (Pip 2 × CH_2_), 52.0 (OCH_3_), 79.3 (COOC(CH_3_)_3_), 126.7 (C=CH_2_), 138.3 (C=CH_2_), 155.0 (COOC(CH_3_)_3_), 167.7 (COOCH_3_). ^15^N-NMR (71 MHz, CDCl_3_): *δ*_N_ ppm −292.1 (*N*-Boc). IR (FT-IR, *ν*_max_, cm^−1^): 2980, 2903 (CH_aliph_), 1711 (C=O), 1673 (C=O), 1432, 1159 (C=C, C–N, C–O–C). HRMS (ESI^+^) C_15_H_25_NNaO_4_ ([M + Na]^+^) calcd. 306.1676, found 306.1675.

#### 3.2.8. General Procedure for Compounds **16a**–**f**

An appropriate *N*-heterocyclic compound **6** (1.1 mmol), DBU (1.15 mmol), and *tert*-butyl 4-[2-(methoxycarbonyl)prop-2-en-1-yl]piperidine-1-carboxylate **15** (1 mmol) were dissolved in acetonitrile (1 mL) and stirred at 60 °C for 4–18 h. The reaction was diluted with water (10 mL) and extracted with ethyl acetate (2 × 15 mL). The organic layer was dried with anhydrous sodium sulfate, filtered, and then concentrated under reduced pressure. The resulting mixtures were purified via flash column chromatography to provide products **16a**–**f**.

*Tert*-butyl 4-{3-methoxy-2-[(morpholin-4-yl)methyl]-3-oxopropyl}piperidine-1-carboxylate (**16a**)

Compound **16a** was obtained from **15** (0.283 g, 1 mmol), morpholine (0.09 mL, 1.1 mmol), and DBU (0.17 mL, 1.15 mmol). The reaction was stirred at 60 °C for 4 h. The obtained residue was purified via flash column chromatography (eluent methylene chloride/methanol, *v*/*v*, 100:2) to give **16a** (0.289 g, 78%) as a colorless liquid. ^1^H-NMR (400 MHz, CDCl_3_): *δ*_H_ ppm 0.98–1.15 (m, 2H, Pip 3,5-H), 1.32–1.34 (m, 2H, Pip 4-H, Pip-CH_2_), 1.44 (s, 9H, C(CH_3_)_3_), 1.56–1.61 (m, 2H, Pip 5-H, Pip-CH_2_), 1.71–1.74 (m, 1H, Pip 3-H), 2.28–2.37 (m, 3H, Mor 3,5-H, Mor-CH_2_), 2.46–2.79 (m, 6H, Mor CH_2_, CHCOOCH_3_, Mor-CH_2_,), 3.61–3.74 (m, 7H, OCH_3_, Mor 2,6-H), 4.06 (s, 2H, Pip 2,6-H). ^13^C-NMR (101 MHz, CDCl_3_): *δ*_C_ ppm 28.6 (COOC(CH_3_)_3_), 31.7 (Pip CH_2_), 32.6 (Pip CH_2_), 34.4 (Pip C-4), 37.5 (Pip-CH_2_), 41.0 (CHCOOCH_3_), 44.0 (Pip 2 × CH_2_), 51.7 (OCH_3_), 53.8 (Mor 2 × CH_2_), 61.5 (Mor-CH_2_), 67.1 (Mor 2 × CH_2_), 79.4 (COOC(CH_3_)_3_), 154.9 (COOC(CH_3_)_3_), 176.1 (COOCH_3_). ^15^N-NMR (71 MHz, CDCl_3_): *δ*_N_ ppm −336.6 (*N*-4), *N*-Boc was not found. IR (FT-IR, *ν*_max_, cm^−1^): 2980, 2889 (CH_aliph_), 1736 (C=O), 1689 (C=O), 1421, 1159 (C=C, C–N, C–O–C). HRMS (ESI^+^) C_19_H_35_N_2_O_5_ ([M + H]^+^) calcd. 371.2540, found 371.2540.

*Tert*-butyl 4-{2-[(1,3-dihydro-2*H*-isoindol-2-yl)methyl]-3-methoxy-3-oxopropyl}piperidine-1-carboxylate (**16b**)

Compound **16b** was obtained from **15** (0.283 g, 1 mmol), isoindoline hydrochloride (0.17 g, 1.1 mmol), and DBU (0.17 mL, 1.15 mmol). The reaction was stirred at 60 °C for 12 h. The obtained residue was purified via flash column chromatography (eluent methylene chloride/methanol, *v*/*v*, 100:2) to give **16b** (0.306 g, 76%) as a dark brown liquid. ^1^H-NMR (400 MHz, CDCl_3_): *δ*_H_ ppm 1.02–1.15 (m, 2H, Pip 3,5-H), 1.39–1.45 (m, 11H, C(CH_3_)_3_, Pip-CH_2_, Pip 4-H), 1.59–1.67 (m, 2H, Pip 3,5-H), 1.75–1.79 (m, 1H, Pip-CH_2_), 2.58–2.74 (m, 2H, *i*-Ind-CH_2_), 2.80–2.90 (m, 2H, Pip 2,6-H), 3.11–3.17 (m, 1H, CHCOOCH_3_), 3.71 (OCH_3_), 3.99–4.11 (m, 6H, Pip 2,6-H, *i*-Ind 2 × CH_2_), 7.17 (s, 4H, *i*-Ind CH). ^13^C-NMR (101 MHz, CDCl_3_): *δ*_C_ ppm 28.6 (COOC(CH_3_)_3_), 31.7 (Pip CH_2_), 32.6 (Pip CH_2_), 34.2 (Pip C-4), 37.8 (Pip-CH_2_), 42.5 (CHCOOCH_3_), 43.8 (Pip 2 × CH_2_), 52.1 (OCH_3_), 58.4 (*i*-Ind-CH_2_), 59.1 (*i*-Ind 2 × CH_2_), 79.5 (COOC(CH_3_)_3_), 122.5 (*i*-Ind 2 × CH), 127.3 (*i*-Ind 2 × CH), 138.8 (*i*-Ind C-3a,7a), 155.0 (COOC(CH_3_)_3_), 175.8 (COOCH_3_). ^15^N-NMR (71 MHz, CDCl_3_): *δ*_N_ ppm −331.3 (*N*-2), *N*-Boc was not found. IR (FT-IR, *ν*_max_, cm^−1^): 2980, 2887 (CH_aliph_), 1733 (C=O), 1686 (C=O), 1422, 1161 (C=C, C–N, C–O–C). HRMS (ESI^+^) C_23_H_35_N_2_O_4_ ([M + H]^+^) calcd. 403.2591, found 403.2591.

*Tert*-butyl 4-{3-methoxy-3-oxo-2-[(1*H*-pyrazol-1-yl)methyl]propyl}piperidine-1-carboxylate (**16c**)

Compound **16c** was obtained from **15** (0.283 g, 1 mmol), 1*H*-pyrazole (0.08 g, 1.1 mmol), and DBU (0.17 mL, 1.15 mmol). The reaction was stirred at 60 °C for 14 h. The obtained residue was purified via flash column chromatography (eluent methylene chloride/methanol, *v*/*v*, 100:2) to give **16c** (0.207 g, 59%) as a colorless liquid. ^1^H-NMR (400 MHz, CDCl_3_): *δ*_H_ ppm 0.98–1.10 (m, 2H, Pip 3,5-H), 1.24–1.34 (m, 2H, Pip 4-H, Pip-CH_2_), 1.43 (s, 9H, C(CH_3_)_3_), 1.58–1.72 (m, 3H, Pip 3,5-H, Pip-CH_2_), 2.58–2.67 (m, 2H, Pip 2,6-H), 3.09–3.19 (m, 1H, CHCOOCH_3_), 3.63 (s, 3H, OCH_3_), 4.04 (m, 2H, Pip 2,6-H), 4.17–4.22 (m, 1H, Pyr-CH_2_), 4.33–4.38 (m, 1H, Pyr-CH_2_), 6.20 (s, Pyr 4-H), 7.33 (s, 1H, Pyr 5-H), 7.50 (s, 1H, Pyr 3-H). ^13^C-NMR (101 MHz, CDCl_3_): *δ*_C_ ppm 28.6 (COOC(CH_3_)_3_), 31.6 (Pip C-5), 32.4 (Pip C-3), 34.0 (Pip C-1), 36.8 (Pip-CH_2_), 43.7 (CHCOOCH_3_), 44.1 (Pip C-2,6), 52.1 (OCH_3_), 53.8 (Pyr-CH_2_), 79.5 (COOC(CH_3_)_3_), 105.6 (Pyr C-4), 129.9 (Pyr C-5), 139.9 (Pyr C-3), 154.9 (COOC(CH_3_)_3_), 174.6 (COOCH_3_). ^15^N-NMR (71 MHz, CDCl_3_): *δ*_N_ ppm −173.8 (*N*-1), −79.3 (*N*-2), *N*-Boc was not found. IR (FT-IR, *ν*_max_, cm^−1^): 2980, 2888 (CH_aliph_), 1734 (C=O), 1685 (C=O), 1422, 1159 (C=C, C–N, C–O–C). HRMS (ESI^+^) C_18_H_29_N_3_NaO_4_ ([M + Na]^+^) calcd. 374.2050, found 374.2053.

*Tert*-butyl 4-{2-[(4-bromo-1*H*-pyrazol-1-yl)methyl]-3-methoxy-3-oxopropyl}piperidine-1-carboxylate (**16d**)

Compound **16d** was obtained from **15** (0.283 g, 1 mmol), 4-bromopyrazole (0.16 g, 1.1 mmol), and DBU (0.17 mL, 1.15 mmol). The reaction was stirred at 60 °C for 18 h. The obtained residue was purified via flash column chromatography (eluent methylene chloride/methanol, *v*/*v*, 100:2) to give **16d** (0.353 g, 82%) as a colorless liquid. ^1^H-NMR (400 MHz, CDCl_3_): *δ*_H_ ppm 0.99–1.14 (m, 2H, Pip 3,5-H), 1.27–1.34 (m, 2H, Pip 4-H, Pip-CH_2_), 1.44 (s, 9H, C(CH_3_)_3_), 1.59–1.72 (m, 3H, Pip 3,5-H, Pip-CH_2_), 2.59–2.68 (m, 2H, Pip 2,6-H), 3.07–3.14 (m, 1H, CHCOOCH_3_), 3.65 (s, 3H, OCH_3_), 4.04–4.07 (m, 2H, Pip 2,6-H), 4.12–4.17 (m, 1H, Pyr-CH_2_), 4.29–4.34 (m, 1H, Pyr-CH_2_), 7.37 (s, 1H, Pyr 5-H), 7.44 (s, 1H, Pyr 3-H). ^13^C-NMR (101 MHz, CDCl_3_): *δ*C ppm 28.6 (COOC(CH_3_)_3_), 31.7 (Pip C-5), 32.4 (Pip C-3), 33.9 (Pip C-1), 36.7 (Pip-CH_2_), 43.8 (Pip C-2,6), 44.0 (CHCOOCH_3_), 52.3 (OCH_3_), 54.2 (Pyr-CH_2_), 79.5 (COOC(CH_3_)_3_), 93.1 (Pyr C-4), 130.1 (Pyr C-5), 140.5 (Pyr C-3), 154.9 (COOC(CH_3_)_3_), 174.3 (COOCH_3_). ^15^N-NMR (71 MHz, CDCl_3_): *δ*_N_ ppm −172.6 (*N*-1), −75.0 (*N*-2), *N*-Boc was not found. IR (FT-IR, *ν*_max_, cm^−1^): 2980, 2888 (CH_aliph_), 1735 (C=O), 1680 (C=O), 1425, 1160 (C=C, C–N, C–O–C). HRMS (ESI^+^) C_18_H_29_BrN_3_O_4_ ([M + H]^+^) calcd. 430.1336, found 430.1329.

*Tert*-butyl 4-(3-methoxy-3-oxo-2-{[3-(trifluoromethyl)-1*H*-pyrazol-1-yl]methyl}propyl)piperidine-1-carboxylate (**16e**)

Compound **16e** was obtained from **15** (0.283 g, 1 mmol), 3-trifluoromethylpyrazole (0.15 g, 1.1 mmol), and DBU (0.17 mL, 1.15 mmol). The reaction was stirred at 60 °C for 18 h. The obtained residue was purified via flash column chromatography (eluent methylene chloride/methanol, *v*/*v*, 100:2) to give **16e** (0.331 g, 79%) as a colorless liquid. ^1^H-NMR (400 MHz, CDCl_3_): *δ*_H_ ppm 1.00–1.14 (m, 2H, Pip 3,5-H), 1.28–1.35 (m, 2H, Pip 4-H, Pip-CH_2_), 1.44 (s, 9H, C(CH_3_)_3_), 1.60–1.73 (m, 3H, Pip 3,5-H, Pip-CH_2_), 2.60–2.68 (m, 2H, Pip 2,6-H), 3.12–3.18 (m, 1H, CHCOOCH_3_), 3.64 (s, 3H, OCH_3_), 4.05–4.08 (m, 2H, Pip 2,6-H), 4.21–4.25 (m, 1H, Pyr-CH_2_), 4.37–4.43 (m, 1H, Pyr-CH_2_), 6.47 (s, 1H, Pyr 4-H), 7.41 (s, 1H, Pyr 5-H). ^13^C-NMR (101 MHz, CDCl_3_): *δ*_C_ ppm 28.6 (COOC(CH_3_)_3_), 31.6 (Pip C-5), 32.4 (Pip C-3), 33.9 (Pip C-1), 36.8 (Pip-CH_2_), 43.8 (Pip C-2,6), 44.1 (CHCOOCH_3_), 52.2 (OCH_3_), 54.3 (Pyr-CH_2_), 79.5 (COOC(CH_3_)_3_), 104.4 (Pyr C-4), 121.3 (q, *J* = 268.6 Hz, CF_3_), 131.5 (Pyr C-5), 143.0 (q, *J* = 38.2 Hz, Pyr C-3), 154.9 (COOC(CH_3_)_3_), 174.3 (COOCH_3_). ^15^N-NMR (71 MHz, CDCl_3_): *δ*_N_ ppm −170.3 (*N*-1), −77.2 (*N*-2), *N*-Boc was not found. ^19^F-NMR (376 MHz, CDCl_3_): *δ*_F_ ppm −62.0 (s, CF_3_). IR (FT-IR, *ν*_max_, cm^−1^): 2980, 2886 (CH_aliph_), 1736 (C=O), 1684 (C=O), 1424, 1159, 1127 (C=C, C–N, C–O–C). HRMS (ESI^+^) C_19_H_29_F_3_N_3_O_4_ ([M + H]^+^) calcd. 420.2105, found 420.2106.

*Tert*-butyl 4-{2-[(1*H*-benzimidazol-1-yl)methyl]-3-methoxy-3-oxopropyl}piperidine-1-carboxylate (**16f**)

Compound **16f** was obtained from **15** (0.283 g, 1 mmol), benzimidazole (0.13 g, 1.1 mmol), and DBU (0.17 mL, 1.15 mmol). The reaction was stirred at 60 °C for 8 h. The obtained residue was purified via flash column chromatography (eluent methylene chloride/methanol, *v*/*v*, 100:2) to give **16f** (0.341 g, 85%) as a colorless liquid. ^1^H-NMR (400 MHz, CDCl_3_): *δ*_H_ ppm 0.99–1.13 (m, 2H, Pip 3,5-H), 1.37–1.44 (m, 11H, C(CH_3_)_3_, Pip 4-H, Pip-CH_2_), 1.59–1.79 (m, 3H, Pip 3,5-H, Pip-CH_2_), 2.58–2.65 (m, 2H, Pip 2,6-H), 3.05–3.12 (m, 1H, CHCOOCH_3_), 3.57 (s, 3H, OCH_3_), 3.99–4.14 (m, 2H, Pip 2,6-H), 4.17–4.22 (m, 1H, Pyr-CH_2_), 4.44–4.50 (m, 1H, Pyr-CH_2_), 7.29–7.36 (m, 3H, Bim), 7.79–7.81 (m, 1H, Bim), 7.90 (s, 1H, Bim 2-H). ^13^C-NMR (101 MHz, CDCl_3_): *δ*_C_ ppm 28.6 (COOC(CH_3_)_3_), 31.6 (Pip C-5), 32.5 (Pip C-3), 34.1 (Pip C-1), 37.2 (Pip-CH_2_), 43.5 (Pip C-2,6), 44.2 (CHCOOCH_3_), 47.1 (OCH_3_), 52.4 (Bim-CH_2_), 79.6 (COOC(CH_3_)_3_), 109.4 (Bim CH), 120.6 (Bim CH), 122.6 (Bim CH), 123.5 (Bim CH), 133.5 (Bim C), 143.2 (Bim C-2), 154.9 (COOC(CH_3_)_3_), 163.9 (Bim C), 174.1 (COOCH_3_). ^15^N-NMR (71 MHz, CDCl_3_): *δ*_N_ ppm −231.0 (*N*-1), −141.9 (*N*-3), *N*-Boc was not found. IR (FT-IR, *ν*_max_, cm^−1^): 2980, 2887 (CH_aliph_), 1733 (C=O), 1682 (C=O), 1424, 1161 (C=C, C–N, C–O–C). HRMS (ESI^+^) C_22_H_32_N_3_O_4_ ([M + H]^+^) calcd. 402.2387, found 402.2387.

#### 3.2.9. General Procedure for Compound **19**

Compound **18** (1 mmol) was dissolved in methylene chloride and cooled to −10 °C. Maintaining the temperature at −10 °C, acetic acid was added dropwise (3.5 mmol) and sodium borohydride was added in portions over 1 h. The reaction was stirred at −10 °C for 6 h. The reaction mixture was diluted with DCM and washed with water and brine. The organic layer was dried with anhydrous sodium sulfate, filtered, and then concentrated under reduced pressure. The obtained residue was purified via flash column chromatography to provide compound **19**.

*Tert*-butyl 3-[(2,2-dimethyl-4,6-dioxo-1,3-dioxan-5-yl)methyl]azetidine-1-carboxylate (**19**)

Compound **18** (0.327 g, 1 mmol) was dissolved in methylene chloride and cooled to −10 °C. Maintaining the temperature at −10 °C, acetic acid was added dropwise (0.2 mL, 3.5 mmol) and sodium borohydride was added in portions over 1 h. The reaction was stirred at −10 °C for 6 h. The reaction mixture was diluted with DCM (10 mL) and washed with water (2 × 15 mL) and brine (15 mL). The organic layer was dried with anhydrous sodium sulfate, filtered, and then concentrated under reduced pressure. The obtained residue was purified via flash column chromatography (eluent *n*-hexane/ethyl acetate, *v*/*v*, 3:1) to provide compound **19** (0.213 g, 68%) as a white solid, mp 114–115 °C. ^1^H-NMR (400 MHz, CDCl_3_): *δ*_H_ ppm 1.42 (s, 9H, C(CH_3_)_3_), 1.77 (d, *J* = 13.4 Hz, 6H, 2 × CH_3_), 2.38 (t, *J* = 6.8 Hz, 2H, Az-CH_2_), 2.83 (hept, *J* = 7.2 Hz, 1H, Az 3-H), 3.39 (t, *J* = 5.7 Hz, 1H, 5-H), 3.61–3.65 (m, 2H, Az 2,4-H), 3.99–4.04 (m, 2H, Az 2,4-H). ^13^C-NMR (101 MHz, CDCl_3_): *δ*_C_ ppm 26.9 (CH_3_), 27.2 (Az C-3), 28.5 (COOC(CH_3_)_3_), 28.6 (CH_3_), 31.1 (Az-CH_2_), 44.7 (C-5), 54.3 (Az 2 × CH_2_), 79.6 (COOC(CH_3_)_3_), 105.4 (C-2), 156.4 (COOC(CH_3_)_3_), 165.3 (2 × C=O). ^15^N-NMR (71 MHz, CDCl_3_): *δ*_N_ ppm −310.6 (*N*-Boc). IR (FT-IR, *ν*_max_, cm^−1^): 2980, 2887 (CH_aliph_), 1738 (C=O), 1693 (C=O), 1394, 1159 (C=C, C–N, C–O–C). HRMS (ESI^+^) C_15_H_24_NO_6_ ([M + H]^+^) calcd. 314.1598, found 314.1598.

#### 3.2.10. General Procedure for Compound **20**

A 50 mL round-bottomed flask was charged under nitrogen atmosphere with compound **19** (1 mmol) and *N*,*N*-dimethylmethyleneiminium iodide (2 mmol). The solids were dissolved in THF and MeOH (1:1). The reaction mixture was stirred at 70 °C for 18 h. Upon cooling of the mixture to room temperature, the solvents were removed in vacuo, and the residue was taken up in ethyl acetate, washed with sat. NaHCO_3_ soln, 10% aq. KHSO_4_ and brine (15 mL). The organic layer was dried with anhydrous sodium sulfate, filtered, and then concentrated under reduced pressure. The resulting mixture was purified via flash chromatography to give the product **20**.

*Tert*-butyl 3-[2-(methoxycarbonyl)prop-2-en-1-yl]azetidine-1-carboxylate (**20**)

A 50 mL round-bottomed flask was charged under nitrogen atmosphere with compound **19** (0.313 g, 1 mmol) and *N*,*N*-dimethylmethyleneiminium iodide (0.37 g, 2 mmol). The solids were dissolved in THF (6 mL) and MeOH (6 mL). The reaction mixture was stirred at 70 °C for 18 h. Upon cooling of the mixture to room temperature, the solvents were removed in vacuo, and the yellow residue was taken up in ethyl acetate (15 mL), washed with sat. NaHCO_3_ soln (15 mL), 10% aq. KHSO_4_ (15 mL) and brine (15 mL). The organic layer was dried with anhydrous sodium sulfate, filtered, and then concentrated under reduced pressure. The resulting mixture was purified via flash column chromatography (eluent *n*-hexane/ethyl acetate, *v*/*v*, 4:1) to give the corresponding product **20** (0.24 g, 94%) as a colorless liquid. ^1^H-NMR (400 MHz, CDCl_3_): *δ*_H_ ppm 1.39 (s, 9H, C(CH_3_)_3_), 2.54–2.55 (m, 2H, Az-CH_2_), 2.64–2.73 (m, 1H, Az 3-H), 3.49–3.53 (m, 2H, Az CH_2_), 3.71 (s, 3H, OCH_3_), 3.94–3.98 (m, 2H, Az CH_2_), 5.46 (s, 1H, C=CH_2_), 6.14 (s, 1H, C=CH_2_). ^13^C-NMR (101 MHz, CDCl_3_): *δ*C ppm 27.3 (Az C-3), 28.4 (COOC(CH_3_)_3_), 36.6 (Az-CH_2_), 52.0 (OCH_3_), 54.1 (Az 2 × CH_2_), 79.3 (COOC(CH_3_)_3_), 125.9 (C=CH_2_), 137.8 (C=CH_2_), 156.4 (COOC(CH_3_)_3_), 167.2 (COOCH_3_). ^15^N-NMR (71 MHz, CDCl_3_): *δ*_N_ ppm −309.9 (*N*-Boc). IR (FT-IR, *ν*_max_, cm^−1^): 2980, 2884 (CH_aliph_), 1697 (C=O), 1645 (C=O), 1394, 1132 (C=C, C–N, C–O–C). HRMS (ESI^+^) C_13_H_21_NNaO_4_ ([M + Na]^+^) calcd. 278.1363, found 278.1363.

#### 3.2.11. General Procedure for Compounds **21a**–**f**

An appropriate *N*-heterocyclic compound **6** (1.1 mmol), DBU (1.15 mmol), and *tert*-butyl 3-[2-(methoxycarbonyl)prop-2-en-1-yl]azetidine-1-carboxylate **20** (1 mmol) were dissolved in acetonitrile (1 mL) and stirred at 60 °C for 4–18 h. The reaction was diluted with water (10 mL) and extracted with ethyl acetate (2 × 15 mL). The organic layer was dried with anhydrous sodium sulfate, filtered, and then concentrated under reduced pressure. The resulting mixtures were purified via flash column chromatography to provide products **21a**–**f**.

*Tert*-butyl 3-{3-methoxy-2-[(morpholin-4-yl)methyl]-3-oxopropyl}azetidine-1-carboxylate (**21a**)

Compound **21a** was obtained from **20** (0.255 g, 1 mmol), morpholine (0.09 mL, 1.1 mmol), and DBU (0.17 mL, 1.15 mmol). The reaction was stirred at 60 °C for 4 h. The obtained residue was purified via flash column chromatography (eluent methylene chloride/methanol, *v*/*v*, 100:2) to give **21a** (0.294 g, 86%) as a yellowish liquid. ^1^H-NMR (400 MHz, CDCl_3_): *δ*_H_ ppm 1.41 (s, 9H, C(CH_3_)_3_), 1.76–1.90 (m, 2H, Az-CH_2_), 2.32–2.40 (m, 3H, Mor CH_2_, Mor-CH_2_), 2.47–2.53 (m, 3H, Az 3-H, Mor CH_2_), 2.55–2.60 (m, 1H, CHCOOCH_3_), 2.63–2.68 (m, 1H, Mor-CH_2_), 3.46–3.52 (m, 2H, Az CH_2_), 3.61–3.71 (m, 7H, OCH_3_, Mor 2 × CH_2_), 3.93–4.00 (m, 2H, Az CH_2_). ^13^C-NMR (101 MHz, CDCl_3_): *δ*_C_ ppm 27.3 (Az C-3), 28.5 (COOC(CH_3_)_3_), 35.3 (Az-CH_2_), 41.7 (CHCOOCH_3_), 51.9 (OCH_3_), 53.7 (Mor 2 × CH_2_), 54.5 (Az 2 × CH_2_), 60.7 (Mor-CH_2_), 66.9 (Mor 2 × CH_2_), 79.5 (COOC(CH_3_)_3_), 156.4 (COOC(CH_3_)_3_), 175.2 (COOCH_3_). ^15^N-NMR (71 MHz, CDCl_3_): *δ*_N_ ppm −338.3 (*N*-4), −309.7 (*N*-Boc). IR (FT-IR, *ν*_max_, cm^−1^): 2980, 2886 (CH_aliph_), 1735 (C=O), 1697 (C=O), 1395, 1116 (C=C, C–N, C–O–C). HRMS (ESI^+^) C_17_H_31_N_2_O_5_ ([M + H]^+^) calcd. 343.2227, found 343.2226.

*Tert*-butyl 3-{2-[(1,3-dihydro-2*H*-isoindol-2-yl)methyl]-3-methoxy-3-oxopropyl}azetidine-1-carboxylate (**21b**)

Compound **21b** was obtained from **20** (0.255 g, 1 mmol), isoindoline hydrochloride (0.17 g, 1.1 mmol), and DBU (0.17 mL, 1.15 mmol). The reaction was stirred at 60 °C for 12 h. The obtained residue was purified via flash column chromatography (eluent methylene chloride/methanol, *v*/*v*, 100:2) to give **21b** (0.243 g, 65%) as a dark brown liquid. ^1^H-NMR (400 MHz, CDCl_3_): *δ*_H_ ppm 1.42 (s, 9H, C(CH_3_)_3_), 1.90–1.94 (m, 2H, Az-CH_2_), 2.51–2.58 (m, 1H, Az 3-H), 2.65–2.70 (m, 1H, CHCOOCH_3_), 2.80–2.85 (m, 1H, *i*-Ind-CH_2_), 3.10–3.15 (m, 1H, *i*-Ind-CH_2_), 3.50–3.56 (m, 2H, Az CH_2_), 3.70 (s, 3H, OCH_3_), 3.96–4.06 (m, 6H, *i*-Ind 1,3-CH_2_, Az CH_2_), 7.20 (s, 4H, *i*-Ind CH). ^13^C-NMR (101 MHz, CDCl_3_): *δ*_C_ ppm 27.3 (Az C-3), 28.5 (COOC(CH_3_)_3_), 35.4 (Az-CH_2_), 43.5 (CHCOOCH_3_), 52.1 (OCH_3_), 54.5 (Az 2 × CH_2_), 57.8 (*i*-Ind-CH_2_), 59.1 (*i*-Ind 2 × CH_2_), 79.6 (COOC(CH_3_)_3_), 122.5 (*i*-Ind 2 × CH), 127.2 (*i*-Ind 2 × CH), 139.0 (*i*-Ind 2 × CH_2_), 156.4 (COOC(CH_3_)_3_), 175.1 (COOCH_3_). ^15^N-NMR (71 MHz, CDCl_3_): *δ*_N_ ppm −335.2 (*N*-2), −310.1 (*N*-Boc). IR (FT-IR, *ν*_max_, cm^−1^): 2980, 2884 (CH_aliph_), 1698 (C=O), 1635 (C=O), 1395, 1133 (C=C, C–N, C–O–C). HRMS (ESI^+^) C_21_H_31_N_2_O_4_ ([M + H]^+^) calcd. 375.2278, found 375.2278.

*Tert*-butyl 3-{3-methoxy-3-oxo-2-[(1*H*-pyrazol-1-yl)methyl]propyl}azetidine-1-carboxylate (**21c**)

Compound **21c** was obtained from **20** (0.255 g, 1 mmol), 1*H*-pyrazole (0.08 g, 1.1 mmol), and DBU (0.17 mL, 1.15 mmol). The reaction was stirred at 60 °C for 14 h. The obtained residue was purified via flash column chromatography (eluent methylene chloride/methanol, *v*/*v*, 100:2) to give **21c** (0.236 g, 73%) as a colorless liquid. ^1^H-NMR (400 MHz, CDCl_3_): *δ*_H_ ppm 1.41 (s, 9H, C(CH_3_)_3_), 1.70–1.77 (m, 1H, Az-CH_2_), 1.89–1.96 (m, 1H, Az-CH_2_), 2.46–2.55 (m, 1H, Az 3-H), 2.95–3.02 (m, 1H, CHCOOCH_3_), 3.46–3.50 (m, 2H, Az CH_2_), 3.63 (s, 3H, OCH_3_), 3.94–4.00 (m, 2H, Az CH_2_), 4.17–4.22 (m, 1H, Pyr-CH_2_), 4.35–4.41 (m, 1H, Pyr-CH_2_), 6.21 (s, 1H, Pyr 4-H), 7.35 (s, 1H, Pyr 5-H), 7.50 (s, 1H, Pyr 3-H). ^13^C-NMR (101 MHz, CDCl_3_): *δ*_C_ ppm 27.0 (Az C-3), 28.5 (COOC(CH_3_)_3_), 34.6 (Az-CH_2_), 44.8 (CHCOOCH_3_), 52.2 (OCH_3_)_,_ 53.1 (Pyr-CH_2_), 54.4 (Az 2 × CH_2_), 79.6 (COOC(CH_3_)_3_), 105.7 (Pyr C-4), 130.1 (Pyr C-5), 140.0 (Pyr C-3), 156.4 (COOC(CH_3_)_3_), 173.8 (COOCH_3_). ^15^N-NMR (71 MHz, CDCl_3_): *δ*_N_ ppm −310.2 (*N*-Boc), −174.5 (*N*-1), −80.1 (*N*-2). IR (FT-IR, *ν*_max_, cm^−1^): 2980, 2885 (CH_aliph_), 1734 (C=O), 1693 (C=O), 1395, 1157 (C=C, C–N, C–O–C). HRMS (ESI^+^) C_16_H_26_N_3_O_4_ ([M + H]^+^) calcd. 324.1918, found 324.1918.

*Tert*-butyl 3-{2-[(4-bromo-1*H*-pyrazol-1-yl)methyl]-3-methoxy-3-oxopropyl}azetidine-1-carboxylate (**21d**)

Compound **21d** was obtained from **20** (0.255 g, 1 mmol), 4-bromopyrazole (0.16 g, 1.1 mmol), and DBU (0.17 mL, 1.15 mmol). The reaction was stirred at 60 °C for 18 h. The obtained residue was purified via flash column chromatography (eluent methylene chloride/methanol, *v*/*v*, 100:2) to give **21d** (0.334 g, 83%) as a colorless liquid. ^1^H-NMR (400 MHz, CDCl_3_): *δ*_H_ ppm 1.42 (s, 9H, C(CH_3_)_3_), 1.72–1.78 (m, 1H, Az-CH_2_), 1.89–1.96 (m, 1H, Az-CH_2_), 2.50–2.57 (m, 1H, Az 3-H), 2.92–2.99 (m, 1H, CHCOOCH_3_), 3.47–3.51 (m, 2H, Az CH_2_), 3.66 (s, 3H, OCH_3_), 3.95–4.01 (m, 2H, Az CH_2_), 4.12–4.17 (m, 1H, Pyr-CH_2_), 4.32–4.37 (m, 1H, Pyr-CH_2_), 7.38 (s, 1H, Pyr 5-H), 7.45 (s, 1H, Pyr 3-H). ^13^C-NMR (101 MHz, CDCl_3_): *δ*_C_ ppm 27.0 (Az C-3), 28.5 (COOC(CH_3_)_3_), 34.6 (Az-CH_2_), 44.5 (CHCOOCH_3_), 52.4 (OCH_3_), 53.7 (Pyr-CH_2_), 54.4 (Az 2 × CH_2_), 79.6 (COOC(CH_3_)_3_), 93.2 (Pyr C-4), 130.2 (Pyr C-5), 140.6 (Pyr C-3), 156.3 (COOC(CH_3_)_3_), 173.6 (COOCH_3_). ^15^N-NMR (71 MHz, CDCl_3_): *δ*_N_ ppm −310.6 (*N*-Boc), −173.4 (*N*-1), −75.2 (*N*-2). IR (FT-IR, *ν*_max_, cm^−1^): 2980, 2884 (CH_aliph_), 1734 (C=O), 1690 (C=O), 1399, 1140 (C=C, C–N, C–O–C). HRMS (ESI^+^) C_16_H_24_BrN_3_NaO_4_ ([M + Na]^+^) calcd. 424.0842, found 424.0842.

*Tert*-butyl 3-(3-methoxy-3-oxo-2-{[3-(trifluoromethyl)-1*H*-pyrazol-1-yl]methyl}propyl)azetidine-1-carboxylate (**21e**)

Compound **21e** was prepared from **20** (0.255 g, 1 mmol), 3-trifluoromethylpyrazole (0.15 g, 1.1 mmol), and DBU (0.17 mL, 1.15 mmol). The reaction was stirred at 60 °C for 18 h. The obtained residue was purified via flash column chromatography (eluent methylene chloride/methanol, *v*/*v*, 100:2) to give **21e** (0.239 g, 61%) as a colorless liquid. ^1^H-NMR (400 MHz, CDCl_3_): *δ*_H_ ppm 1.42 (s, 9H, C(CH_3_)_3_), 1.75–1.81 (m, 1H, Az-CH_2_), 1.91–1.99 (m, 1H, Az-CH_2_), 2.51–2.58 (m, 1H, Az 3-H), 2.99–3.05 (m, 1H, CHCOOCH_3_), 3.48–3.52 (m, 2H, Az CH_2_), 3.65 (s, 3H, OCH_3_), 3.96–4.03 (m, 2H, Az CH_2_), 4.20–4.25 (m, 1H, Pyr-CH_2_), 4.40–4.45 (m, 1H, Pyr-CH_2_), 6.47 (s, 1H, Pyr 4-H), 7.41 (s, 1H, Pyr 5-H). ^13^C-NMR (101 MHz, CDCl_3_): *δ*_C_ ppm 27.0 (Az C-3), 28.5 (COOC(CH_3_)_3_), 34.7 (Az-CH_2_), 44.6 (CHCOOCH_3_), 52.4 (OCH_3_), 53.8 (Pyr-CH_2_), 54.3 (Az 2 × CH_2_), 79.7 (COOC(CH_3_)_3_), 104.5 (Pyr C-4), 122.1 (q, *J* = 268.5 Hz, CF_3_), 131.7 (Pyr C-5), 142.1 (q, *J* = 38.3 Hz, Pyr C-3), 156.4 (COOC(CH_3_)_3_), 173.5 (COOCH_3_). ^15^N-NMR (71 MHz, CDCl_3_): *δ*_N_ ppm −311.1 (*N*-Boc), −171.1 (*N*-1), −77.6 (*N*-2). ^19^F-NMR (376 MHz, CDCl_3_): *δ*_F_ ppm −62.0 (s, CF_3_). IR (FT-IR, *ν*_max_, cm^−1^): 2980, 2885 (CH_aliph_), 1736 (C=O), 1691 (C=O), 1392, 1127 (C=C, C–N, C–O–C). HRMS (ESI^+^) C_17_H_24_F_3_N_3_NaO_4_ ([M + Na]^+^) calcd. 414.1611, found 414.1610.

*Tert*-butyl 3-{2-[(1*H*-benzimidazol-1-yl)methyl]-3-methoxy-3-oxopropyl}azetidine-1-carboxylate (**21f**)

Compound **21f** was obtained from **20** (0.255 g, 1 mmol), benzimidazole (0.13 g, 1.1 mmol), and DBU (0.17 mL, 1.15 mmol). The reaction was stirred at 60 °C for 8 h. The obtained residue was purified via flash column chromatography (eluent methylene chloride/methanol, *v*/*v*, 100:2) to give **21f** (0.332 g, 89%) as a colorless liquid. ^1^H-NMR (400 MHz, CDCl_3_): *δ*_H_ ppm 1.42 (s, 9H, C(CH_3_)_3_), 1.82–1.86 (m, 1H, Az-CH_2_), 2.00–2.07 (m, 1H, Az-CH_2_), 2.54–2.57 (m, 1H, Az 3-H), 2.90–2.96 (m, 1H, CHCOOCH_3_), 3.49–3.51 (m, 2H, az CH_2_), 3.59 (s, 3H, OCH_3_), 3.97–4.02 (m, 2H, az CH_2_), 4.19–4.24 (m, 1H, Bim-CH_2_), 4.49–4.55 (m, 1H, Bim-CH_2_), 7.29–7.35 (m, 3H, Bim), 7.81–7.83 (m, 1H, Bim), 7.95 (s, 1H, Bim 2-H). ^13^C-NMR (101 MHz, CDCl_3_): *δ*_C_ ppm 27.0 (Az C-3), 28.5 (COOC(CH_3_)_3_), 35.0 (Az-CH_2_), 44.3 (CHCOOCH_3_), 46.6 (Bim-CH_2_), 52.5 (OCH_3_), 54.3 (Az 2 × CH_2_), 79.8 (COOC(CH_3_)_3_), 109.4 (Bim CH), 120.5 (Bim CH), 122.9 (Bim CH), 123.7 (Bim CH), 133.4 (Bim C), 143.2 (Bim C-2), 156.3 (COOC(CH_3_)_3_), 163.8 (Bim C), 173.4 (COOCH_3_). ^15^N-NMR (71 MHz, CDCl_3_): *δ*_N_ ppm −311.0 (*N*-Boc), −231.5 (*N*-1), −143.7 (*N*-3). IR (FT-IR, *ν*_max_, cm^−1^): 2980, 2885 (CH_aliph_), 1733 (C=O), 1689 (C=O), 1393, 1140 (C=C, C–N, C–O–C). HRMS (ESI^+^) C_20_H_28_N_3_O_4_ ([M + H]^+^) calcd. 374.2074, found 374.2074.

## 4. Conclusions

In conclusion, two complementary synthetic approaches for the preparation of novel piperidine- and azetidine-containing 2,3-disubstituted propanoic acid derivatives were developed. Ketone-derived Meldrum’s acid intermediates were converted into the corresponding *α*,*β*-unsaturated methyl esters and subsequently subjected to aza-Michael addition with a range of saturated and aromatic nitrogen heterocycles. This route afforded piperidine-containing derivatives in 35–89% yields and the corresponding azetidine derivatives in 61–92% yields. A complementary approach starting from *N*-Boc-piperidine-4-carboxylic acid and *N*-Boc-azetidine-3-carboxylic acid provided regioisomeric propanoate derivatives in 59–85% and 61–89% yields, respectively. Thus, the two synthetic pathways enabled access to structurally diverse compounds bearing piperidine or azetidine fragments at complementary positions of the propanoate backbone. The structures of the synthesized compounds were unambiguously confirmed via HRMS and multinuclear NMR spectroscopy, including detailed ^1^H-^1^H COSY, ^1^H-^1^H NOESY, ^1^H-^13^C HMBC, and ^1^H-^15^N HMBC analyses of representative compounds. The developed methodology provides efficient access to structurally diverse heterocyclic propanoic acid derivatives that may serve as versatile amino acid building blocks for peptide chemistry and DNA-encoded library synthesis.

## Figures and Tables

**Table 1:** Optimization of reaction solvent for compound **3**.

Entry	Solvent *	Yield (%), 18 h **
1	DCM	18
2	Toluene	16
3	THF	59
**4**	**DMSO**	**71**
5	EtOH	28
6	CH_3_CN	24

* All reaction mixtures were stirred at room temperature. ** After filtration through a Buchner funnel using methanol. Bold indicates the optimized reaction conditions giving the highest isolated yield.

**Table 2:** Optimization of reaction conditions for compounds **7a** and **7i**: effect of base, solvent, temperature and reaction time.

Entry	Base	Solvent	7a, Yield (%), 4 h **	7a, Yield (%), 18 h **	7i, Yield (%), 4 h **	7i, Yield (%), 18 h **
1	K_2_CO_3_	CH_3_CN ^a^	15	12	25	35
2	Cs_2_CO_3_	CH_3_CN ^a^	8	7	46	65
3	NaH	CH_3_CN ^a^	n.d.	n.d.	n.d.	n.d.
4	TEA	CH_3_CN ^a^	31	26	n.d.	n.d.
**5**	**DBU**	**CH_3_CN** ^a^	**70**	**65**	**32**	**81**
6	K_3_PO_4_	CH_3_CN ^a^	54	45	22	48
7	NaOCH_3_	CH_3_CN ^a^	35	21	35	37
8	*t*-BuOK	CH_3_CN ^a^	39	19	45	38
9	DBU	CH_3_CN ^b^	34	50	12	40
10	DBU	CH_3_CN ^c^	10	42	8	19
11	DBU	CH_3_CN ^d^	38	32	42	23
12	DBU	EtOH ^e^	45	32	n.d.	n.d.
13	DBU	THF ^e^	n.d.	10	n.d.	22
14	DBU	Dioxane ^a^	8	25	n.d.	n.d.
15	-	CH_3_CN ^a^	n.d.	n.d.	n.d.	n.d.

^a^ Reaction mixtures were stirred at ^a^ 60 °C; ^b^ room temperature; ^c^ 40 °C; ^d^ 80 °C; ^e^ reflux. ** After purification via column chromatography. n.d. = not detected. Bold indicates the optimized reaction conditions giving the highest isolated yield.

## Data Availability

The data presented in this study are available on request from the corresponding authors.

## Supplementary Materials

The following supporting information can be downloaded at: https://www.mdpi.com/article/10.3390/molecules31162909/s1, Figure S1: ^1^H NMR (400 MHz, CDCl_3_) spectrum of compound **3**, Figure S2: ^13^C NMR (101 MHz, CDCl_3_) spectrum of compound **3**, Figure S3: HRMS (ESI-TOF) spectrum of compound **3**, Figure S4: ^1^H NMR (400 MHz, CDCl_3_) spectrum of compound **5**, Figure S5: ^13^C NMR (101 MHz, CDCl_3_) spectrum of compound **5**, Figure S6: HRMS (ESI-TOF) spectrum of compound **5**, Figure S7: ^1^H NMR (400 MHz, CDCl_3_) spectrum of compound **7a**, Figure S8: ^13^C NMR (101 MHz, CDCl_3_) spectrum of compound **7a**, Figure S9: HRMS (ESI-TOF) spectrum of compound **7a**, Figure S10: ^1^H NMR (400 MHz, CDCl_3_) spectrum of compound **7b**, Figure S11: ^13^C NMR (101 MHz, CDCl_3_) spectrum of compound **7b**, Figure S12: HRMS (ESI-TOF) spectrum of compound **7b**, Figure S13: ^1^H NMR (400 MHz, CDCl_3_) spectrum of compound **7c**, Figure S14: ^13^C NMR (101 MHz, CDCl_3_) spectrum of compound **7c**, Figure S15: HRMS (ESI-TOF) spectrum of compound **7c**, Figure S16: ^1^H NMR (400 MHz, CDCl_3_) spectrum of compound **7d**, Figure S17: ^13^C NMR (101 MHz, CDCl_3_) spectrum of compound **7d**, Figure S18: HRMS (ESI-TOF) spectrum of compound **7d**, Figure S19: ^1^H NMR (400 MHz, CDCl_3_) spectrum of compound **7e**, Figure S20: ^13^C NMR (101 MHz, CDCl_3_) spectrum of compound **7e**, Figure S21: HRMS (ESI-TOF) spectrum of compound **7e**, Figure S22: ^1^H NMR (400 MHz, CDCl_3_) spectrum of compound **7f**, Figure S23: ^13^C NMR (101 MHz, CDCl_3_) spectrum of compound **7f**, Figure S24: HRMS (ESI-TOF) spectrum of compound **7f**, Figure S25: ^1^H NMR (400 MHz, CDCl_3_) spectrum of compound **7g**, Figure S26: ^13^C NMR (101 MHz, CDCl_3_) spectrum of compound **7g**, Figure S27: HRMS (ESI-TOF) spectrum of compound **7g**, Figure S28: ^1^H NMR (400 MHz, CDCl_3_) spectrum of compound **7h**, Figure S29: ^13^C NMR (101 MHz, CDCl_3_) spectrum of compound **7h**, Figure S30: HRMS (ESI-TOF) spectrum of compound **7h**, Figure S31: ^1^H NMR (400 MHz, CDCl_3_) spectrum of compound **7i**, Figure S32: ^13^C NMR (101 MHz, CDCl_3_) spectrum of compound **7i**, Figure S33: HRMS (ESI-TOF) spectrum of compound **7i**, Figure S34: ^1^H NMR (400 MHz, CDCl_3_) spectrum of compound **7j**, Figure S35: ^13^C NMR (101 MHz, CDCl_3_) spectrum of compound **7j**, Figure S36: HRMS (ESI-TOF) spectrum of compound **7j**, Figure S37: ^1^H NMR (400 MHz, CDCl_3_) spectrum of compound **7k**, Figure S38: ^13^C NMR (101 MHz, CDCl_3_) spectrum of compound **7k**, Figure S39: HRMS (ESI-TOF) spectrum of compound **7k**, Figure S40: ^1^H NMR (400 MHz, CDCl_3_) spectrum of compound **7l**, Figure S41: ^13^C NMR (101 MHz, CDCl_3_) spectrum of compound **7l**, Figure S42: HRMS (ESI-TOF) spectrum of compound **7l**, Figure S43: ^1^H NMR (400 MHz, CDCl_3_) spectrum of compound **7m**, Figure S44: ^13^C NMR (101 MHz, CDCl_3_) spectrum of compound **7m**, Figure S45: ^19^F NMR (376 MHz, CDCl_3_) spectrum of compound **7m**, Figure S46: HRMS (ESI-TOF) spectrum of compound **7m**, Figure S47: ^1^H NMR (400 MHz, CDCl_3_) spectrum of compound **7n**, Figure S48: ^13^C NMR (101 MHz, CDCl_3_) spectrum of compound **7n**, Figure S49: HRMS (ESI-TOF) spectrum of compound **7n**, Figure S50: ^1^H NMR (400 MHz, CDCl_3_) spectrum of compound **7o**, Figure S51: ^13^C NMR (101 MHz, CDCl_3_) spectrum of compound **7o**, Figure S52: HRMS (ESI-TOF) spectrum of compound **7o**, Figure S53: ^1^H NMR (400 MHz, CDCl_3_) spectrum of compound **7p**, Figure S54: ^13^C NMR (101 MHz, CDCl_3_) spectrum of compound **7p**, Figure S55: HRMS (ESI-TOF) spectrum of compound **7p**, Figure S56: ^1^H NMR (400 MHz, CDCl_3_) spectrum of compound **7r**, Figure S57: ^13^C NMR (101 MHz, CDCl_3_) spectrum of compound **7r**, Figure S58: HRMS (ESI-TOF) spectrum of compound **7r**, Figure S59: ^1^H NMR (400 MHz, CDCl_3_) spectrum of compound **9**, Figure S60: ^13^C NMR (101 MHz, CDCl_3_) spectrum of compound **9**, Figure S61: HRMS (ESI-TOF) spectrum of compound **9**, Figure S62: ^1^H NMR (400 MHz, CDCl_3_) spectrum of compound **10**, Figure S63: ^13^C NMR (101 MHz, CDCl_3_) spectrum of compound **10**, Figure S64: HRMS (ESI-TOF) spectrum of compound **10**, Figure S65: ^1^H NMR (400 MHz, CDCl_3_) spectrum of compound **11a**, Figure S66: ^13^C NMR (101 MHz, CDCl_3_) spectrum of compound **11a**, Figure S67: HRMS (ESI-TOF) spectrum of compound **11a**, Figure S68: ^1^H NMR (400 MHz, CDCl_3_) spectrum of compound **11b**, Figure S69: ^13^C NMR (101 MHz, CDCl_3_) spectrum of compound **11b**, Figure S70: HRMS (ESI-TOF) spectrum of compound **11b**, Figure S71: ^1^H NMR (400 MHz, CDCl_3_) spectrum of compound **11c**, Figure S72: ^13^C NMR (101 MHz, CDCl_3_) spectrum of compound **11c**, Figure S73: HRMS (ESI-TOF) spectrum of compound **11c**, Figure S74: ^1^H NMR (400 MHz, CDCl_3_) spectrum of compound **11d**, Figure S75: ^13^C NMR (101 MHz, CDCl_3_) spectrum of compound **11d**, Figure S76: HRMS (ESI-TOF) spectrum of compound **11d**, Figure S77: ^1^H NMR (400 MHz, CDCl_3_) spectrum of compound **11e**, Figure S78: ^13^C NMR (101 MHz, CDCl_3_) spectrum of compound **11e**, Figure S79: ^1^H–^1^H COSY NMR (400 MHz, CDCl_3_) spectrum of compound **11e**, Figure S80: ^1^H–^13^C HSQC NMR (400/101 MHz, CDCl_3_) spectrum of compound **11e**, Figure S81: ^1^H–^13^C HMBC NMR (400/101 MHz, CDCl_3_) spectrum of compound **11e**, Figure S82: ^1^H–^15^N HMBC NMR (400/71 MHz, CDCl_3_) spectrum of compound **11e**, Figure S83: ^1^H–^1^H NOESY NMR (400 MHz, CDCl_3_) spectrum of compound **11e**, Figure S84: ^19^F NMR (376 MHz, CDCl_3_) spectrum of compound **11e**, Figure S85: HRMS (ESI-TOF) spectrum of compound **11e**, Figure S86: ^1^H NMR (400 MHz, CDCl_3_) spectrum of compound **11f**, Figure S87: ^13^C NMR (101 MHz, CDCl_3_) spectrum of compound **11f**, Figure S88: HRMS (ESI-TOF) spectrum of compound **11f**, Figure S89: ^1^H NMR (400 MHz, CDCl_3_) spectrum of compound **15**, Figure S90: ^13^C NMR (101 MHz, CDCl_3_) spectrum of compound **15**, Figure S91: HRMS (ESI-TOF) spectrum of compound **15**, Figure S92: ^1^H NMR (400 MHz, CDCl_3_) spectrum of compound **16a**, Figure S93: ^13^C NMR (101 MHz, CDCl_3_) spectrum of compound **16a**, Figure S94: HRMS (ESI-TOF) spectrum of compound **16a**, Figure S95: ^1^H NMR (400 MHz, CDCl_3_) spectrum of compound **16b**, Figure S96: ^13^C NMR (101 MHz, CDCl_3_) spectrum of compound **16b**, Figure S97: HRMS (ESI-TOF) spectrum of compound **16b**, Figure S98: ^1^H NMR (400 MHz, CDCl_3_) spectrum of compound **16c**, Figure S99: ^13^C NMR (101 MHz, CDCl_3_) spectrum of compound **16c**, Figure S100: HRMS (ESI-TOF) spectrum of compound **16c**, Figure S101: ^1^H NMR (400 MHz, CDCl_3_) spectrum of compound **16d**, Figure S102: ^13^C NMR (101 MHz, CDCl_3_) spectrum of compound **16d**, Figure S103: HRMS (ESI-TOF) spectrum of compound **16d**, Figure S104: ^1^H NMR (400 MHz, CDCl_3_) spectrum of compound **16e**, Figure S105: ^13^C NMR (101 MHz, CDCl_3_) spectrum of compound **16e**, Figure S106: HRMS (ESI-TOF) spectrum of compound **16e**, Figure S107: ^1^H NMR (400 MHz, CDCl_3_) spectrum of compound **16f**, Figure S108: ^13^C NMR (101 MHz, CDCl_3_) spectrum of compound **16f**, Figure S109: HRMS (ESI-TOF) spectrum of compound **16f**, Figure S110: ^1^H NMR (400 MHz, CDCl_3_) spectrum of compound **19**, Figure S111: ^13^C NMR (101 MHz, CDCl_3_) spectrum of compound **19**, Figure S112: HRMS (ESI-TOF) spectrum of compound **19**, Figure S113: ^1^H NMR (400 MHz, CDCl_3_) spectrum of compound **20**, Figure S114: ^13^C NMR (101 MHz, CDCl_3_) spectrum of compound **20**, Figure S115: HRMS (ESI-TOF) spectrum of compound **20**, Figure S116: ^1^H NMR (400 MHz, CDCl_3_) spectrum of compound **21a**, Figure S117: ^13^C NMR (101 MHz, CDCl_3_) spectrum of compound **21a**, Figure S118: HRMS (ESI-TOF) spectrum of compound **21a**, Figure S119: ^1^H NMR (400 MHz, CDCl_3_) spectrum of compound **21b**, Figure S120: ^13^C NMR (101 MHz, CDCl_3_) spectrum of compound **21b**, Figure S121: HRMS (ESI-TOF) spectrum of compound **21b**, Figure S122: ^1^H NMR (400 MHz, CDCl_3_) spectrum of compound **21c**, Figure S123: ^13^C NMR (101 MHz, CDCl_3_) spectrum of compound **21c**, Figure S124: HRMS (ESI-TOF) spectrum of compound **21c**, Figure S125: ^1^H NMR (400 MHz, CDCl_3_) spectrum of compound **21d**, Figure S126: ^13^C NMR (101 MHz, CDCl_3_) spectrum of compound **21d**, Figure S127: HRMS (ESI-TOF) spectrum of compound **21d**, Figure S128: ^1^H NMR (400 MHz, CDCl_3_) spectrum of compound **21e**, Figure S129: ^13^C NMR (101 MHz, CDCl_3_) spectrum of compound **21e**, Figure S130: ^1^H–^1^H COSY NMR (400 MHz, CDCl_3_) spectrum of compound **21e**, Figure S131: ^1^H–^13^C HSQC NMR (400/101 MHz, CDCl_3_) spectrum of compound **21e**, Figure S132: ^1^H–^13^C HMBC NMR (400/101 MHz, CDCl_3_) spectrum of compound **21e**, Figure S133: ^1^H–^15^N HMBC NMR (400/71 MHz, CDCl_3_) spectrum of compound **21e**, Figure S134: ^19^F NMR (376 MHz, CDCl_3_) spectrum of compound **21e**, Figure S135: HRMS (ESI-TOF) spectrum of compound **21e**, Figure S136: ^1^H NMR (400 MHz, CDCl_3_) spectrum of compound **21f**, Figure S137: ^13^C NMR (101 MHz, CDCl_3_) spectrum of compound **21f**, Figure S138: HRMS (ESI-TOF) spectrum of compound **21f**, Figure S139: Chiral HPLC analysis of compound **7r**, Figure S140: Chiral HPLC analysis of compound **11f**, Figure S141: Chiral HPLC analysis of compound **16f**, Figure S142: Chiral HPLC analysis of compound **21f**.
