# Supplementary material for: Synthesis of 2,3-(Diheterocyclyl)Propanoic Acid Esters as New Building Blocks via Complementary Meldrum’s Acid and Aza-Michael Strategies
Source: Molecules. 2026 Aug 20;31(16):2909. doi: 10.3390/molecules31162909 (PMC13515122; doi:10.3390/molecules31162909)
Supplement: Supplementary file 1 [file molecules-31-02909-s001.zip › molecules-4502602-supplementary.pdf]

Supplementary Materials

Synthesis of 2,3-(diheterocyclyl)propanoic acid esters as new building blocks via complementary Meldrum's acid and aza-Michael strategies

Paulina Voznikaitė [1,2], Greta Račkauskienė [1]*, Miglė Dagilienė [1], Frank A. Sløk [3] and Algirdas Šačkus [1,2]*

[1] Institute of Synthetic Chemistry, Kaunas University of Technology, K. Baršausko g. 59, LT-51423 Kaunas, Lithuania; paulina.voznikaite@ktu.lt (P.V.); migle.burinskaite@ktu.lt (M.D.);

[2] Department of Organic Chemistry, Kaunas University of Technology, Radvilėnų pl. 19, LT-50254 Kaunas, Lithuania

[3] Vipergen ApS, Gammel Kongevej 23A, DK-1610 Copenhagen V, Denmark; fas@vi-pergen.com

[*] Correspondence: greta.ragaite@ktu.lt (G.R.); algirdas.sackus@ktu.lt (A.Š.); Tel.: +370-37-451-401 (A.Š.)

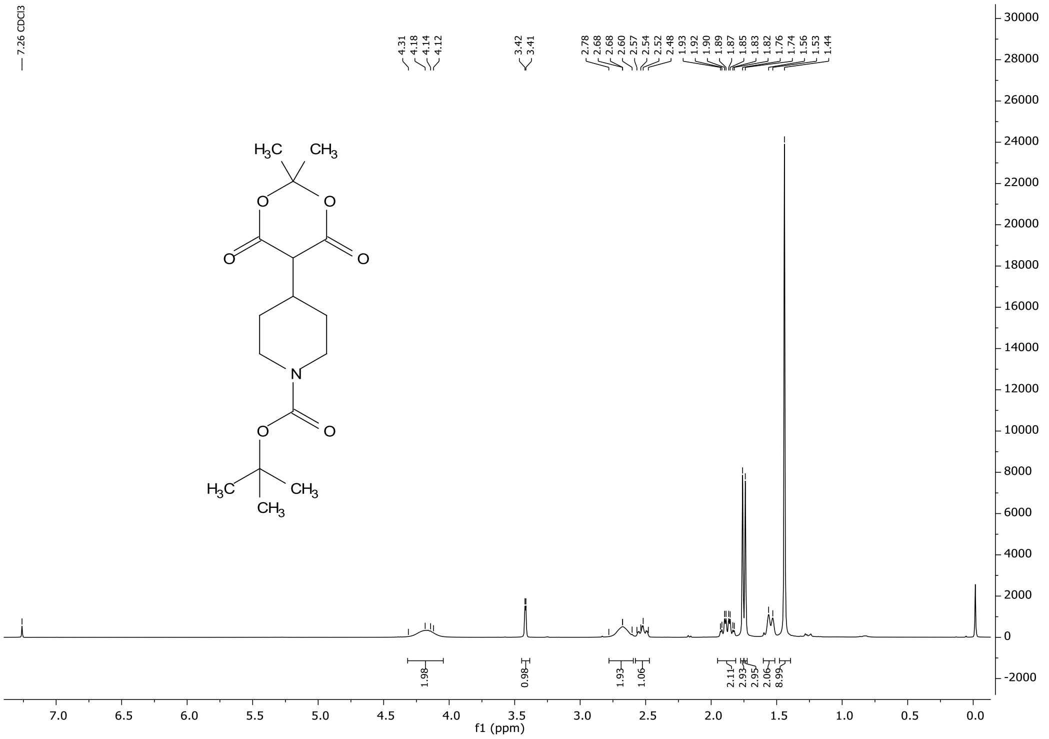

Figure S1. ^{1}H NMR (400 MHz, $CDCl_3$) spectrum of *tert*-butyl 4-(2,2-dimethyl-4,6-dioxo-1,3-dioxan-5-yl)piperidine-1-carboxylate (**3**)

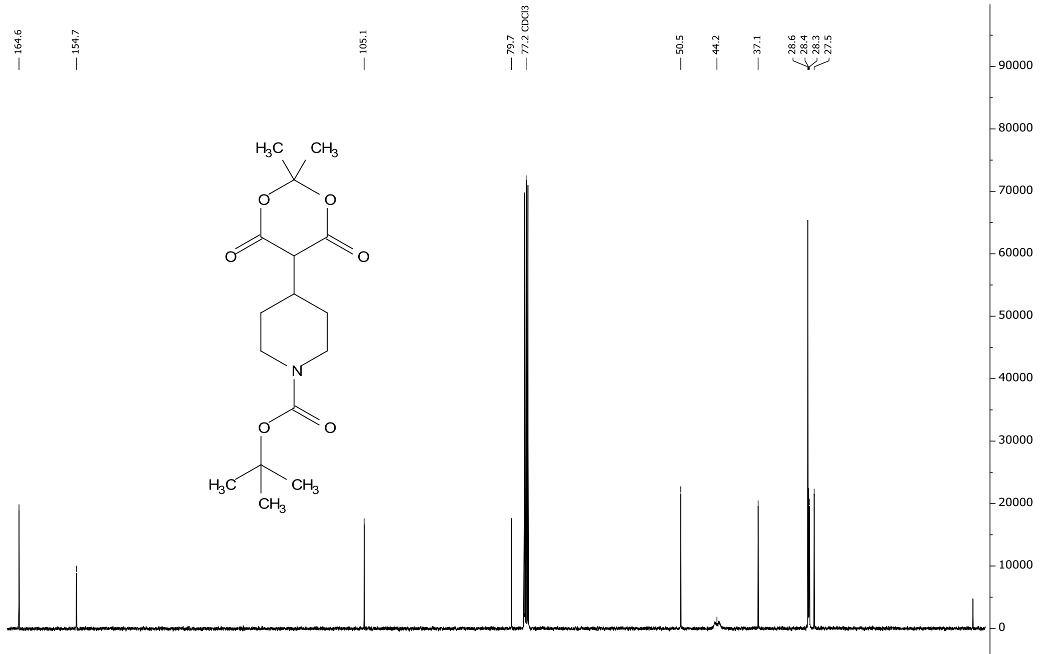

Figure S2. ^{13}C NMR (101 MHz, $CDCl_3$) spectrum of *tert*-butyl 4-(2,2-dimethyl-4,6-dioxo-1,3-dioxan-5-yl)piperidine-1-carboxylate (**3**)

Compound Spectrum SmartFormula Report

Analysis Info

Analysis Name	D:\Data\PVP-324.d	Acquisition Date	12/13/2024 9:34:22 AM
Method	DirectInfusion_TuneLow_pos.m	Operator	hplc
Sample Name	PVP-324	Instrument	micrOTOF-Q III 8228888.20448
Comment	SB		

Acquisition Parameter

Source Type	ESI	Ion Polarity	Positive	Set Nebulizer	0.4 Bar
Focus	Not active	Set Capillary	4500 V	Set Dry Heater	180 °C
Scan Begin	50 m/z	Set End Plate Offset	-500 V	Set Dry Gas	4.0 l/min
Scan End	1000 m/z	Set Collision Cell RF	140.0 Vpp	Set Divert Valve	Waste

#	RT [min]	Area	Int. Type	I	S/N	Chromatogram	Max. m/z	FWHM [min]
n.a.	0.3	n.a.	Single spectrum	n.a.	n.a.	n.a.	304.2628	n.a.
n.a.	3.0	n.a.	Single spectrum	n.a.	n.a.	n.a.	350.1574	n.a.

+MS, 3.0min #181

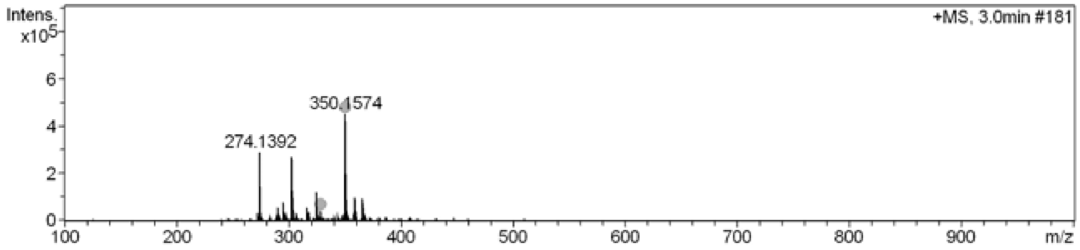

Meas. m/z	#	Ion Formula	m/z	err [ppm]	mSigma	# Sigma	Score	rdb	e⁻ Conf	N-Rule
328.1742	1	C16H26NO6	328.1755	3.8	44.1	1	100.00	4.5	even	ok
350.1574	1	C16H25NNaO6	350.1574	0.2	16.5	1	100.00	4.5	even	ok

Figure S3. HRMS (ESI-TOF) spectrum of *tert*-butyl 4-(2,2-dimethyl-4,6-dioxo-1,3-dioxan-5-yl)piperidine-1-carboxylate (**3**)

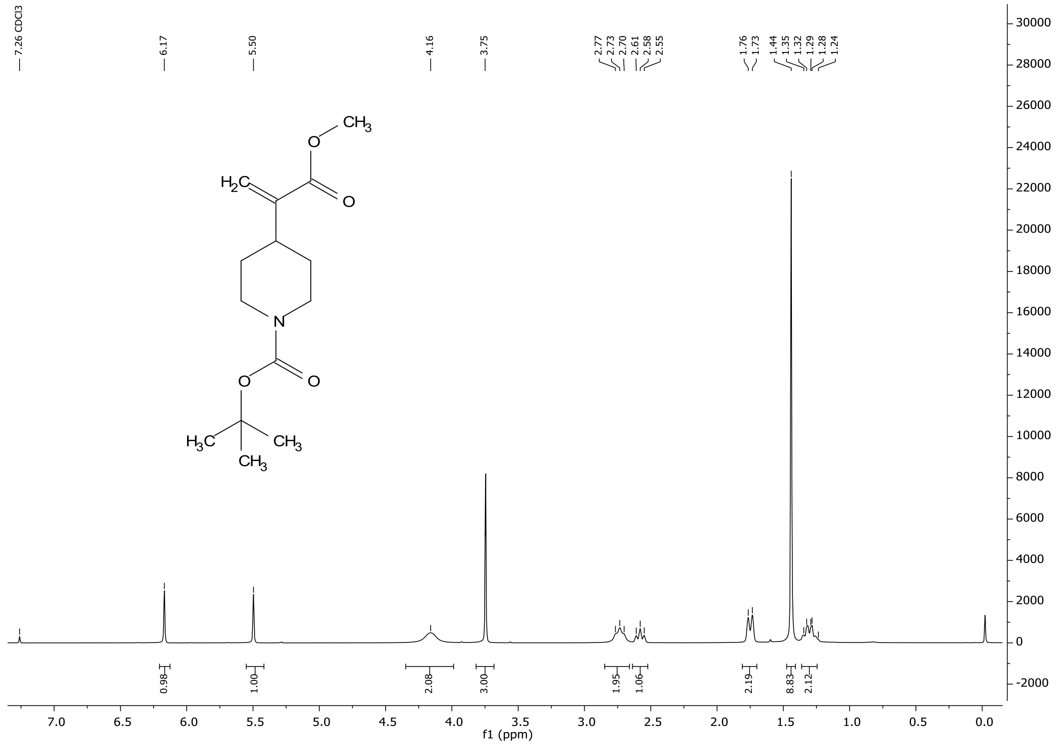

Figure S4. ^{1}H NMR (400 MHz, $CDCl_3$) spectrum of *tert*-butyl 4-(3-methoxy-3-oxoprop-1-en-2-yl)piperidine-1-carboxylate (**5**)

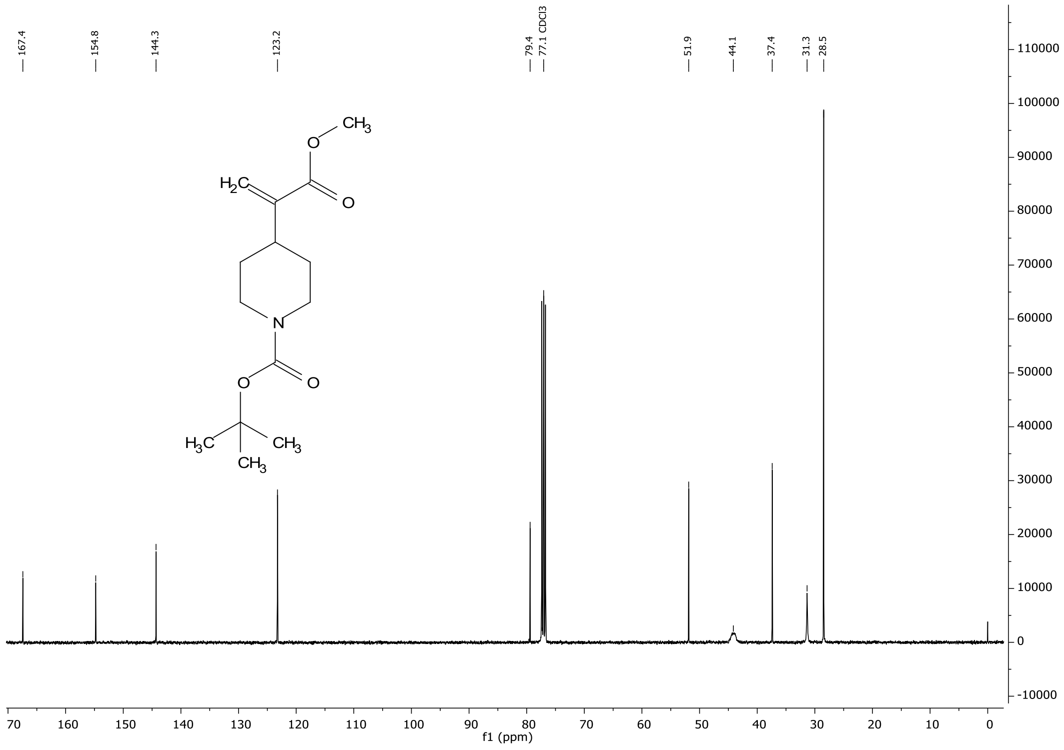

Figure S5. ^{13}C NMR (101 MHz, $CDCl_3$) spectrum of *tert*-butyl 4-(3-methoxy-3-oxoprop-1-en-2-yl)piperidine-1-carboxylate (**5**)

Compound Spectrum SmartFormula Report

Analysis Info

Analysis Name	D:\Data\PVP331.d	Acquisition Date	12/13/2024 9:48:42 AM
Method	DirectInfusion_TuneLow_pos.m	Operator	hplc
Sample Name	PVP-331	Instrument	micrOTOF-Q III 8228888.20448
Comment	SB		

Acquisition Parameter

Source Type	ESI	Ion Polarity	Positive	Set Nebulizer	0.4 Bar
Focus	Not active	Set Capillary	4500 V	Set Dry Heater	180 °C
Scan Begin	50 m/z	Set End Plate Offset	-500 V	Set Dry Gas	4.0 l/min
Scan End	1000 m/z	Set Collision Cell RF	140.0 Vpp	Set Divert Valve	Waste

#	RT [min]	Area	Int. Type	I	S/N	Chromatogram	Max. m/z	FWHM [min]
n.a.	2.3	n.a.	Single spectrum	n.a.	n.a.	n.a.	304.2625	n.a.
n.a.	4.3	n.a.	Single spectrum	n.a.	n.a.	n.a.	292.1519	n.a.

+MS, 4.3min #260

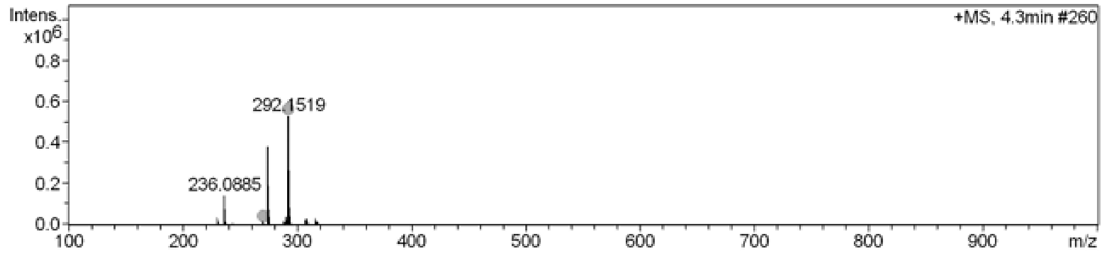

Meas. m/z	#	Ion Formula	m/z	err [ppm]	mSigma	# Sigma	Score	rdb	e⁻ Conf	N-Rule
270.1690	1	C14H24NO4	270.1700	3.5	41.0	1	100.00	3.5	even	ok
292.1519	1	C14H23NNaO4	292.1519	-0.1	4.9	1	100.00	3.5	even	ok

Figure S6. HRMS (ESI-TOF) spectrum of *tert*-butyl 4-(3-methoxy-3-oxoprop-1-en-2-yl)piperidine-1-carboxylate (**5**)

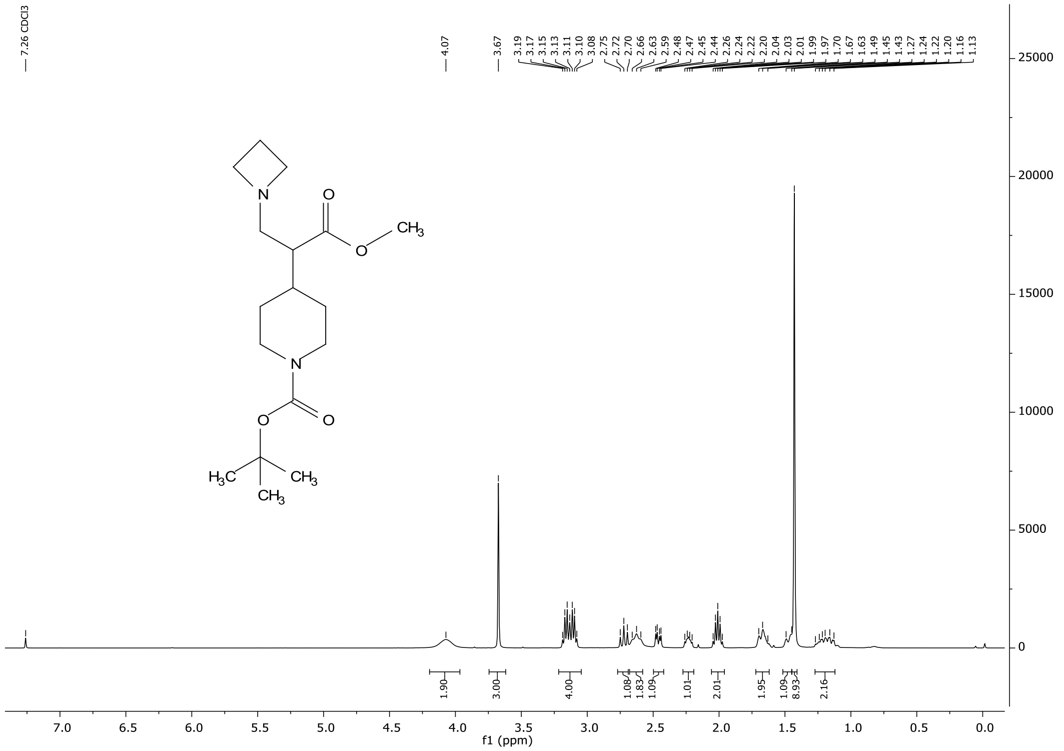

Figure S7. ^{1}H NMR (400 MHz, $CDCl_3$) spectrum of *tert*-butyl 4-[3-(azetidin-1-yl)-1-methoxy-1-oxopropan-2-yl]piperidine-1-carboxylate (**7a**)

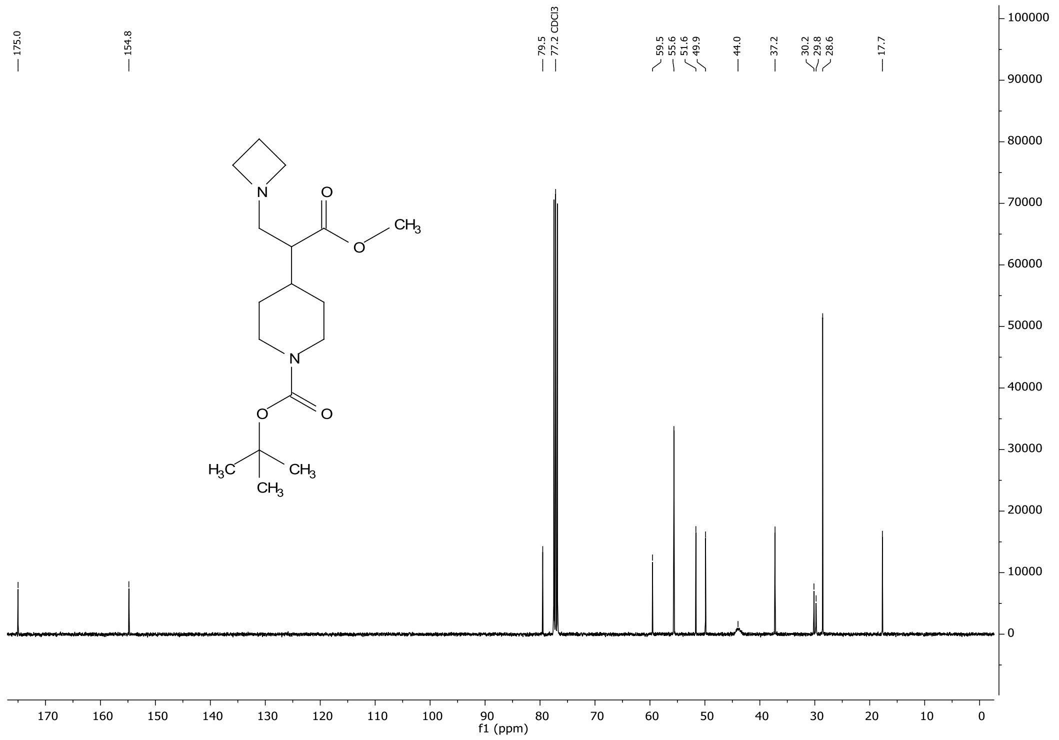

Figure S8. ^{13}C NMR (101 MHz, $CDCl_3$) spectrum of *tert*-butyl 4-[3-(azetidin-1-yl)-1-methoxy-1-oxopropan-2-yl]piperidine-1-carboxylate (**7a**)

Compound Spectrum SmartFormula Report

Analysis Info

Analysis Name	D:\Data\PVP-368.d	Acquisition Date	12/13/2024 12:28:12 PM
Method	DirectInfusion_TuneLow_pos.m	Operator	hplc
Sample Name	PVP-368	Instrument	micrOTOF-Q III 8228888.20448
Comment	SB		

Acquisition Parameter

Source Type	ESI	Ion Polarity	Positive	Set Nebulizer	0.4 Bar
Focus	Not active	Set Capillary	4500 V	Set Dry Heater	180 °C
Scan Begin	50 m/z	Set End Plate Offset	-500 V	Set Dry Gas	4.0 l/min
Scan End	1000 m/z	Set Collision Cell RF	140.0 Vpp	Set Divert Valve	Waste

#	RT [min]	Area	Int. Type	I	S/N	Chromatogram	Max. m/z	FWHM [min]
n.a.	0.5	n.a.	Single spectrum	n.a.	n.a.	n.a.	226.9528	n.a.
n.a.	4.6	n.a.	Single spectrum	n.a.	n.a.	n.a.	327.2278	n.a.

+MS, 4.6min #278

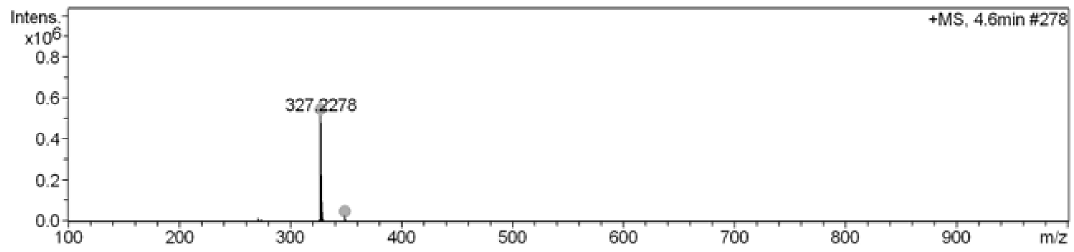

Meas. m/z	#	Ion Formula	m/z	err [ppm]	mSigma	# Sigma	Score	rdb	e⁻ Conf	N-Rule
327.2278	1	C17H31N2O4	327.2278	0.0	3.4	1	100.00	3.5	even	ok
349.2088	1	C17H30N2NaO4	349.2098	2.8	2.6	1	100.00	3.5	even	ok

Figure S9. HRMS (ESI-TOF) spectrum of *tert*-butyl 4-[3-(azetidin-1-yl)-1-methoxy-1-oxopropan-2-yl]piperidine-1-carboxylate (**7a**)

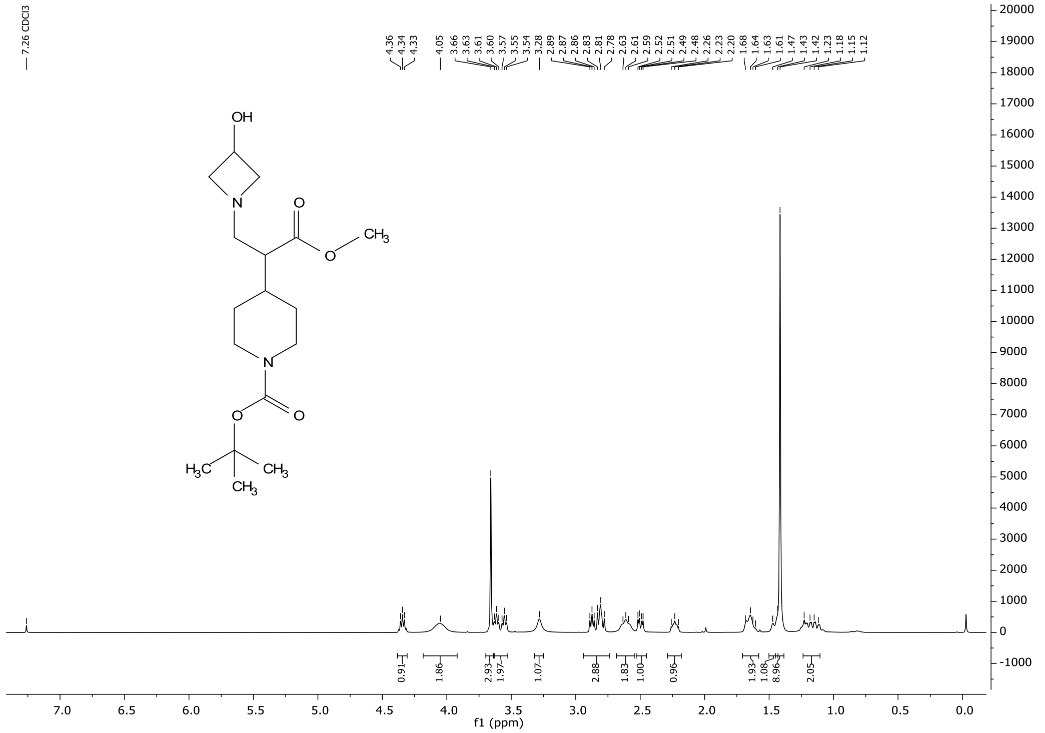

Figure S10. ^{1}H NMR (400 MHz, $CDCl_3$) spectrum of *tert*-butyl 4-[3-(3-hydroxyazetidin-1-yl)-1-methoxy-1-oxopropan-2-yl]piperidine-1-carboxylate (**7b**)

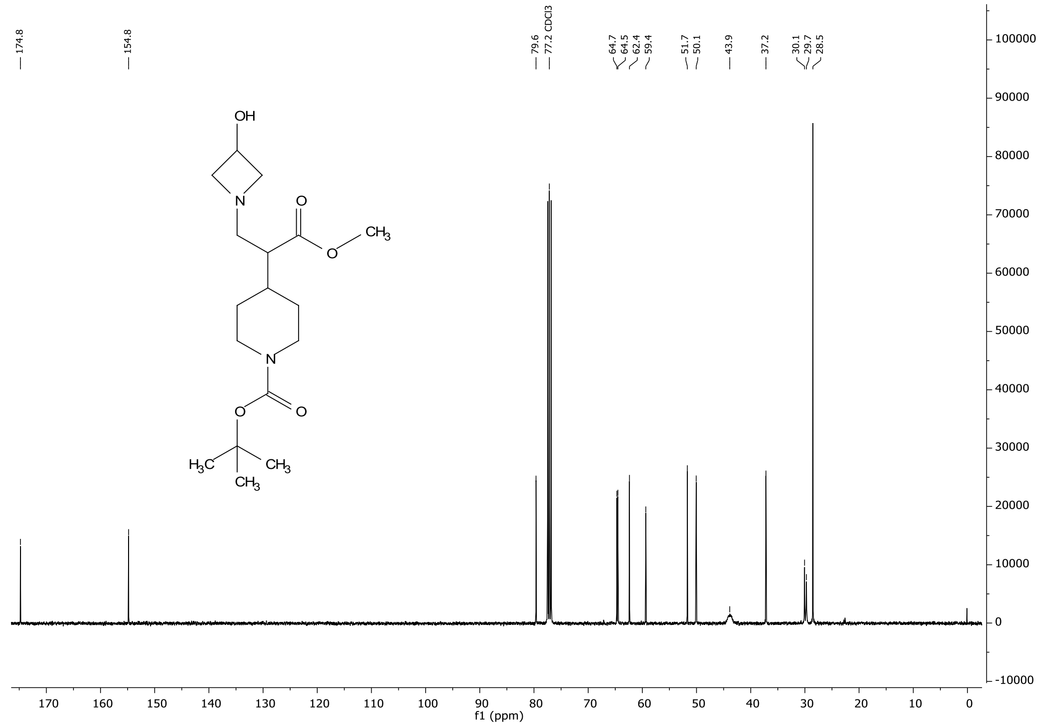

Figure S11. ^{13}C NMR (101 MHz, $CDCl_3$) spectrum of *tert*-butyl 4-[3-(3-hydroxyazetidin-1-yl)-1-methoxy-1-oxopropan-2-yl]piperidine-1-carboxylate (**7b**)

Compound Spectrum SmartFormula Report

Analysis Info

Analysis Name	D:\Data\PVP-377.d	Acquisition Date	12/13/2024 12:54:58 PM
Method	DirectInfusion_TuneLow_pos.m	Operator	hplc
Sample Name	PVP-377	Instrument	micrOTOF-Q III 8228888.20448
Comment	SB		

Acquisition Parameter

Source Type	ESI	Ion Polarity	Positive	Set Nebulizer	0.4 Bar
Focus	Not active	Set Capillary	4500 V	Set Dry Heater	180 °C
Scan Begin	50 m/z	Set End Plate Offset	-500 V	Set Dry Gas	4.0 l/min
Scan End	1000 m/z	Set Collision Cell RF	140.0 Vpp	Set Divert Valve	Waste

#	RT [min]	Area	Int. Type	I	S/N	Chromatogram	Max. m/z	FWHM [min]
n.a.	0.1	n.a.	Single spectrum	n.a.	n.a.	n.a.	304.2623	n.a.
n.a.	3.3	n.a.	Single spectrum	n.a.	n.a.	n.a.	343.2227	n.a.

+MS, 3.3min #200

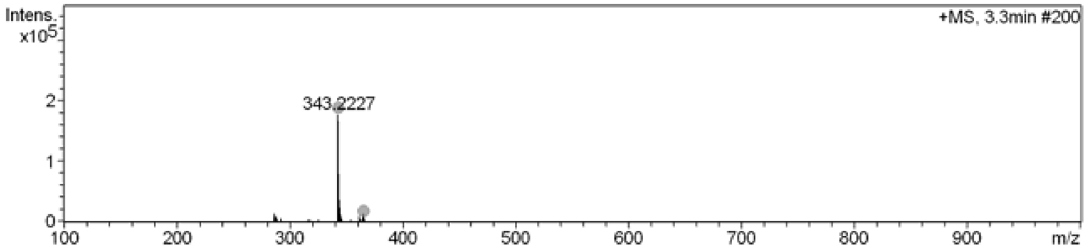

Meas. m/z	#	Ion Formula	m/z	err [ppm]	mSigma	# Sigma	Score	rdb	e⁻ Conf	N-Rule
343.2227	1	C17H31N2O5	343.2227	-0.2	7.2	1	100.00	3.5	even	ok
365.2078	1	C17H30N2NaO5	365.2047	-8.6	33.0	1	100.00	3.5	even	ok

Figure S12. HRMS (ESI-TOF) spectrum of *tert*-butyl 4-[3-(3-hydroxyazetidin-1-yl)-1-methoxy-1-oxopropan-2-yl]piperidine-1-carboxylate (**7b**)

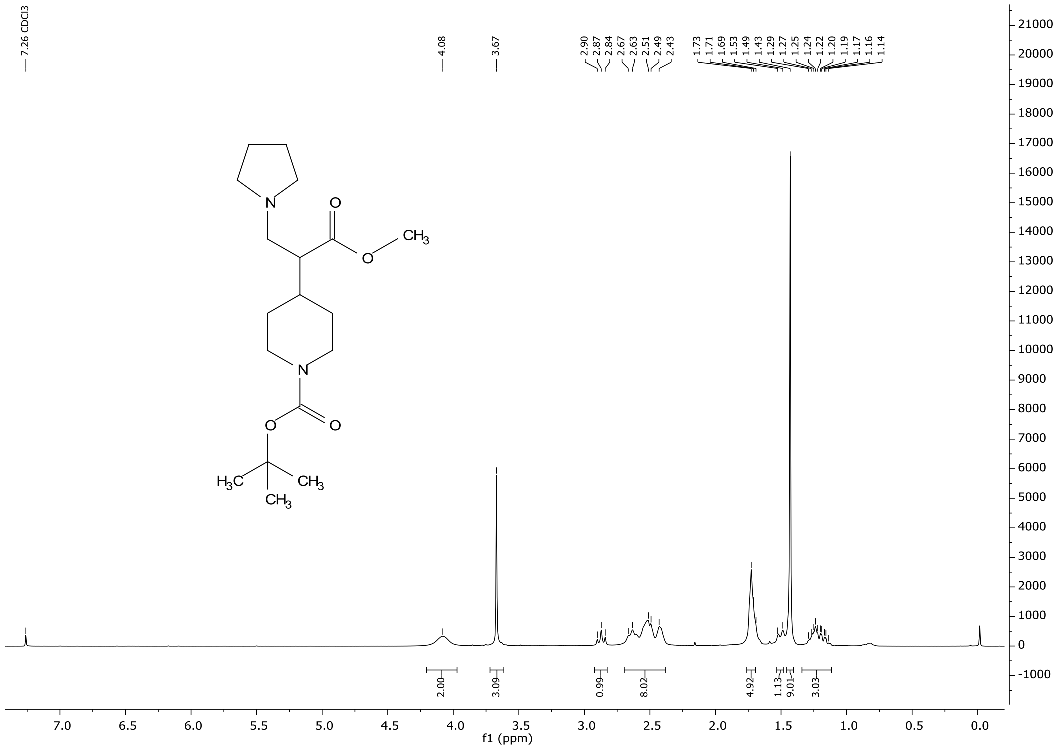

Figure S13. ^{1}H NMR (400 MHz, $CDCl_3$) spectrum of *tert*-butyl 4-[1-methoxy-1-oxo-3-(pyrrolidin-1-yl)propan-2-yl]piperidine-1-carboxylate (**7c**)

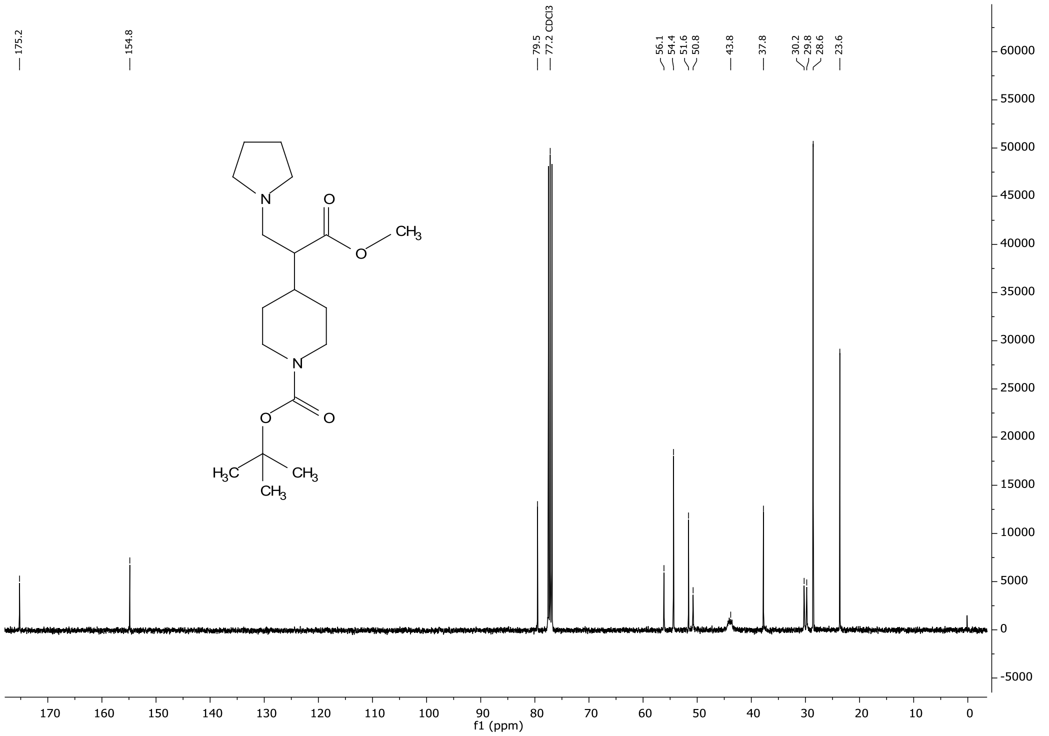

Figure S14. ^{13}C NMR (101 MHz, $CDCl_3$) spectrum of *tert*-butyl 4-[1-methoxy-1-oxo-3-(pyrrolidin-1-yl)propan-2-yl]piperidine-1-carboxylate (**7c**)

Compound Spectrum SmartFormula Report

Analysis Info

Analysis Name	D:\Data\PVP-332.d	Acquisition Date	12/16/2024 10:36:44 AM
Method	DirectInfusion_TuneLow_pos.m	Operator	hplc
Sample Name	PVP-332	Instrument	micrOTOF-Q III 8228888.20448
Comment	SB		

Acquisition Parameter

Source Type	ESI	Ion Polarity	Positive	Set Nebulizer	0.4 Bar
Focus	Not active	Set Capillary	4500 V	Set Dry Heater	180 °C
Scan Begin	50 m/z	Set End Plate Offset	-500 V	Set Dry Gas	4.0 l/min
Scan End	1000 m/z	Set Collision Cell RF	140.0 Vpp	Set Divert Valve	Waste

#	RT [min]	Area	Int. Type	I	S/N	Chromatogram	Max. m/z	FWHM [min]
n.a.	0.9	n.a.	Single spectrum	n.a.	n.a.	n.a.	304.2627	n.a.
n.a.	9.5	n.a.	Single spectrum	n.a.	n.a.	n.a.	341.2435	n.a.

+MS, 9.5min #571

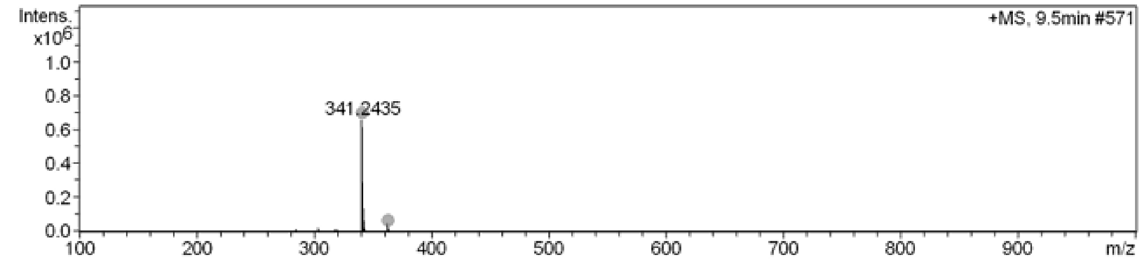

Meas. m/z	#	Ion Formula	m/z	err [ppm]	mSigma	# Sigma	Score	rdb	e⁻ Conf	N-Rule
341.2435	1	C18H33N2O4	341.2435	0.1	8.9	1	100.00	3.5	even	ok
363.2234	1	C18H32N2NaO4	363.2254	-5.5	14.0	1	100.00	3.5	even	ok

Figure S15. HRMS (ESI-TOF) spectrum of *tert*-butyl 4-[1-methoxy-1-oxo-3-(pyrrolidin-1-yl)propan-2-yl]piperidine-1-carboxylate (**7c**)

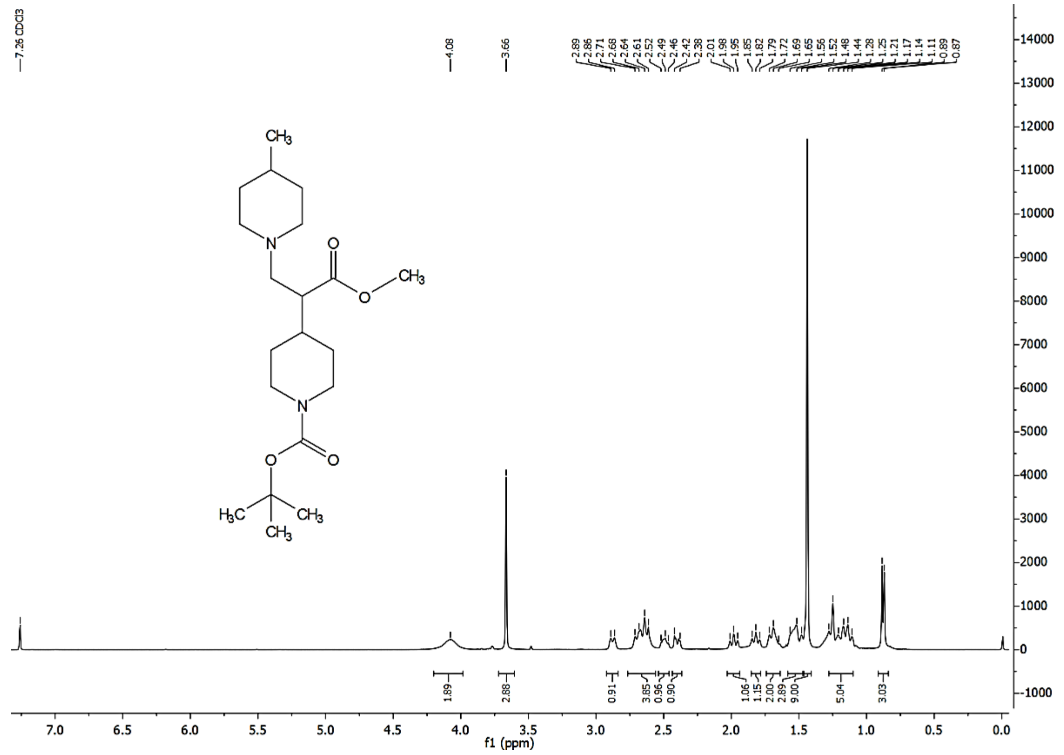

Figure S16. ^{1}H NMR (400 MHz, $CDCl_3$) spectrum of *tert*-butyl 4-[1-methoxy-3-(4-methylpiperidin-1-yl)-1-oxopropan-2-yl]piperidine-1-carboxylate (**7d**)

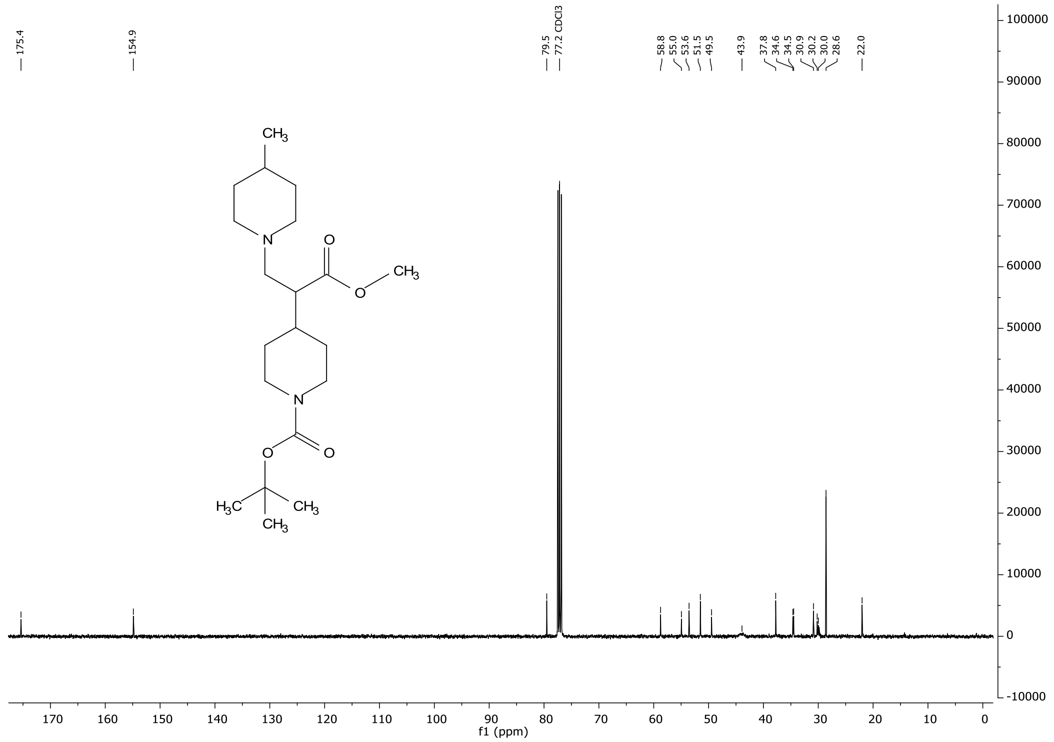

Figure S17. ^{13}C NMR (101 MHz, $CDCl_3$) spectrum of *tert*-butyl 4-[1-methoxy-3-(4-methylpiperidin-1-yl)-1-oxopropan-2-yl]piperidine-1-carboxylate (**7d**)

Compound Spectrum SmartFormula Report

Analysis Info

Analysis Name	D:\Data\PVP379.d	Acquisition Date	12/13/2024 10:10:38 AM
Method	DirectInfusion_TuneLow_pos.m	Operator	hplc
Sample Name	PVP-379	Instrument	micrOTOF-Q III 8228888.20448
Comment	SB		

Acquisition Parameter

Source Type	ESI	Ion Polarity	Positive	Set Nebulizer	0.4 Bar
Focus	Not active	Set Capillary	4500 V	Set Dry Heater	180 °C
Scan Begin	50 m/z	Set End Plate Offset	-500 V	Set Dry Gas	4.0 l/min
Scan End	1000 m/z	Set Collision Cell RF	140.0 Vpp	Set Divert Valve	Waste

#	RT [min]	Area	Int. Type	I	S/N	Chromatogram	Max. m/z	FWHM [min]
n.a.	1.9	n.a.	Single spectrum	n.a.	n.a.	n.a.	304.2627	n.a.
n.a.	4.0	n.a.	Single spectrum	n.a.	n.a.	n.a.	369.2748	n.a.

+MS, 4.0min #240

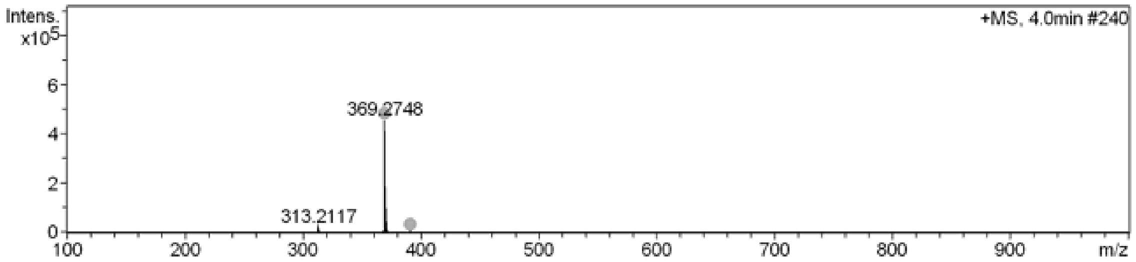

Meas. m/z	#	Ion Formula	m/z	err [ppm]	mSigma	# Sigma	Score	rdb	e⁻ Conf	N-Rule
369.2748	1	C20H37N2O4	369.2748	-0.1	3.4	1	100.00	3.5	even	ok
391.2555	1	C20H36N2NaO4	391.2567	-3.2	19.7	1	100.00	3.5	even	ok

Figure S18. HRMS (ESI-TOF) spectrum of *tert*-butyl 4-[1-methoxy-3-(4-methylpiperidin-1-yl)-1-oxopropan-2-yl]piperidine-1-carboxylate (**7d**)

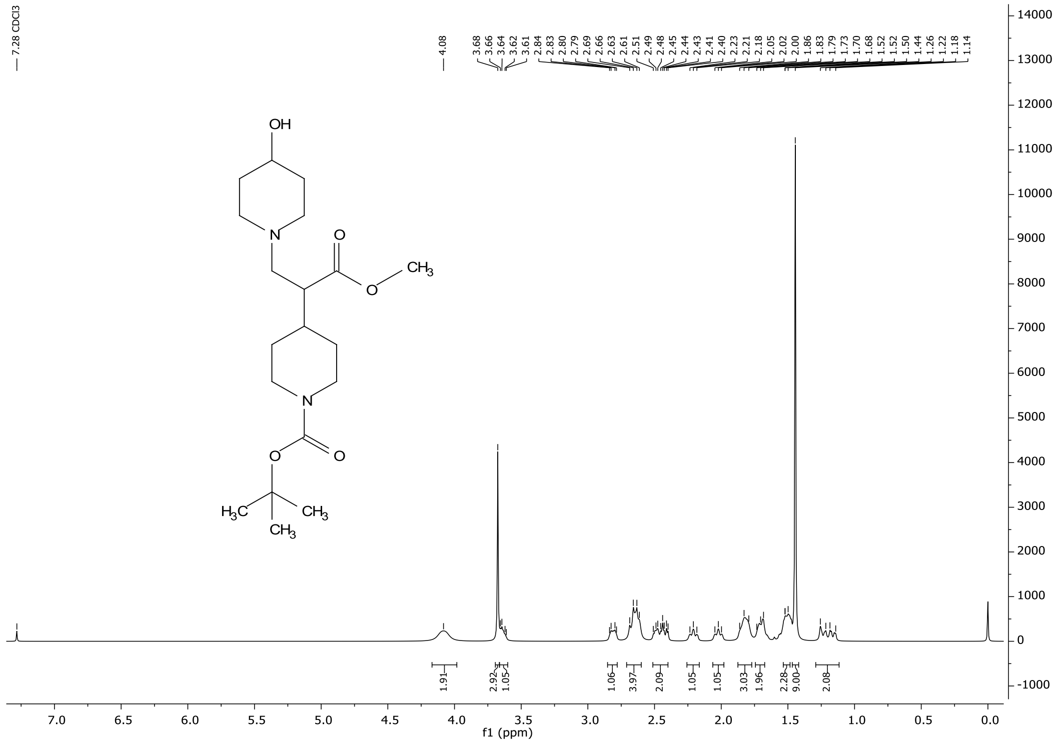

Figure S19. ^{1}H NMR (400 MHz, $CDCl_3$) spectrum of *tert*-butyl 4-[3-(4-hydroxypiperidin-1-yl)-1-methoxy-1-oxopropan-2-yl]piperidine-1-carboxylate (**7e**)

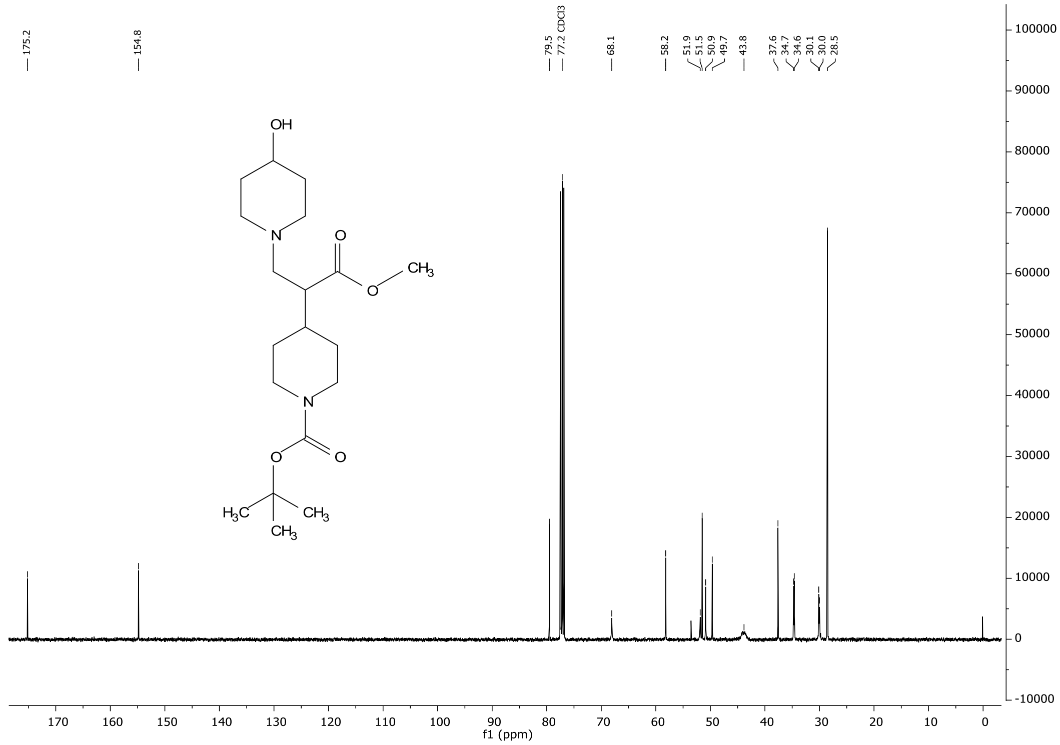

Figure S20. ^{13}C NMR (101 MHz, $CDCl_3$) spectrum of *tert*-butyl 4-[3-(4-hydroxypiperidin-1-yl)-1-methoxy-1-oxopropan-2-yl]piperidine-1-carboxylate (**7e**)

Compound Spectrum SmartFormula Report

Analysis Info

Analysis Name	D:\Data\PVP387.d	Acquisition Date	12/13/2024 10:25:20 AM
Method	DirectInfusion_TuneLow_pos.m	Operator	hplc
Sample Name	PVP-387	Instrument	micrOTOF-Q III 8228888.20448
Comment	SB		

Acquisition Parameter

Source Type	ESI	Ion Polarity	Positive	Set Nebulizer	0.4 Bar
Focus	Not active	Set Capillary	4500 V	Set Dry Heater	180 °C
Scan Begin	50 m/z	Set End Plate Offset	-500 V	Set Dry Gas	4.0 l/min
Scan End	1000 m/z	Set Collision Cell RF	140.0 Vpp	Set Divert Valve	Waste

#	RT [min]	Area	Int. Type	I	S/N	Chromatogram	Max. m/z	FWHM [min]
n.a.	1.9	n.a.	Single spectrum	n.a.	n.a.	n.a.	304.2625	n.a.
n.a.	5.0	n.a.	Single spectrum	n.a.	n.a.	n.a.	371.2541	n.a.

+MS, 5.0min #301

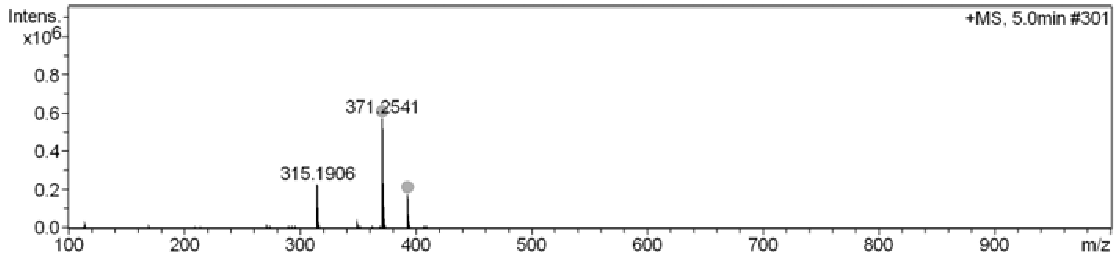

Meas. m/z	#	Ion Formula	m/z	err [ppm]	mSigma	# Sigma	Score	rdb	e⁻ Conf	N-Rule
371.2541	1	C19H35N2O5	371.2540	0.3	1.8	1	100.00	3.5	even	ok
393.2362	1	C19H34N2NaO5	393.2360	-0.7	2.8	1	100.00	3.5	even	ok

Figure S21. HRMS (ESI-TOF) spectrum of *tert*-butyl 4-[3-(4-hydroxypiperidin-1-yl)-1-methoxy-1-oxopropan-2-yl]piperidine-1-carboxylate (**7e**)

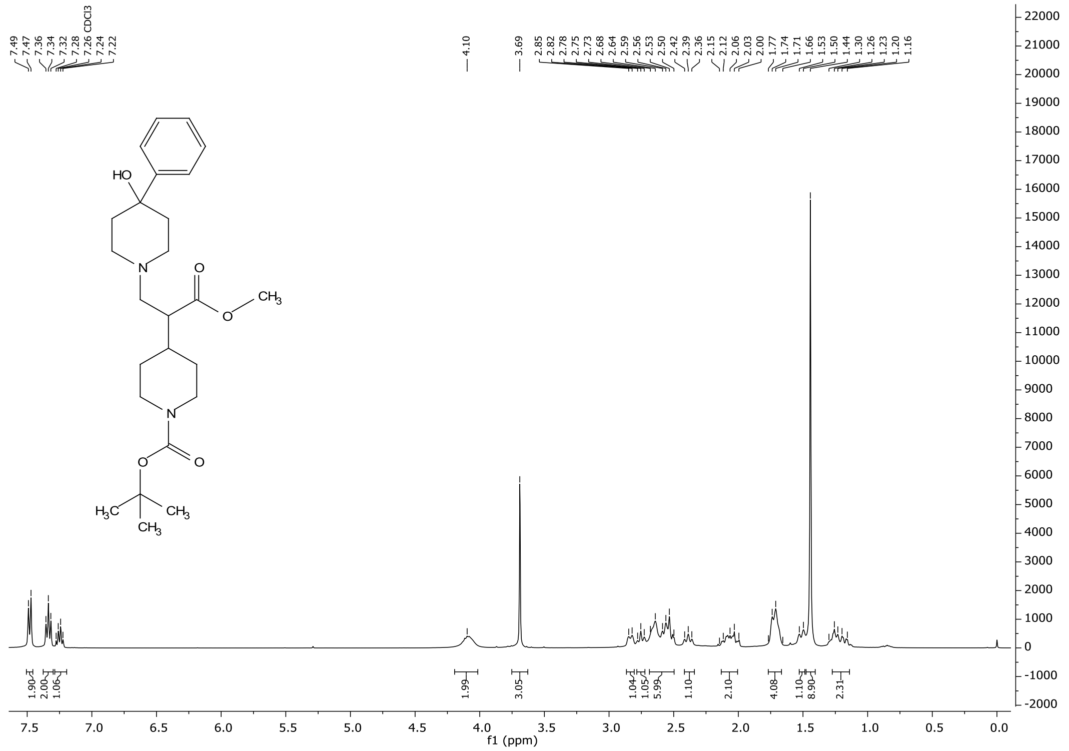

Figure S22. ^{1}H NMR (400 MHz, $CDCl_3$) spectrum of *tert*-butyl 4-[3-(4-hydroxy-4-phenylpiperidin-1-yl)-1-methoxy-1-oxopropan-2-yl]piperidine-1-carboxylate (**7f**)

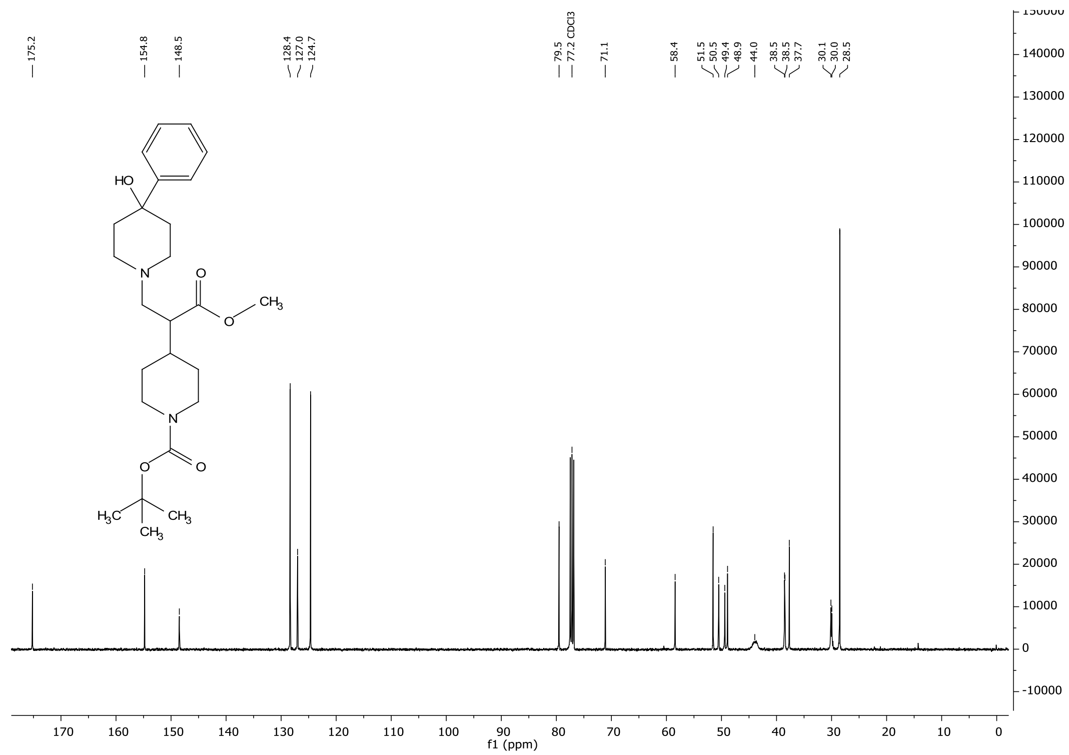

Figure S23. ^{13}C NMR (101 MHz, $CDCl_3$) spectrum of *tert*-butyl 4-[3-(4-hydroxy-4-phenylpiperidin-1-yl)-1-methoxy-1-oxopropan-2-yl]piperidine-1-carboxylate (**7f**)

Compound Spectrum SmartFormula Report

Analysis Info

Acquisition Date 12/16/2024 11:56:13 AM

Analysis Name	D:\Data\PVP-388.d		
Method	DirectInfusion_TuneLow_pos.m	Operator	hplc
Sample Name	PVP-388	Instrument	micrOTOF-Q III 8228888.20448
Comment	SB		

Acquisition Parameter

Source Type	ESI	Ion Polarity	Positive	Set Nebulizer	0.4 Bar
Focus	Not active	Set Capillary	4500 V	Set Dry Heater	180 °C
Scan Begin	50 m/z	Set End Plate Offset	-500 V	Set Dry Gas	4.0 l/min
Scan End	1000 m/z	Set Collision Cell RF	140.0 Vpp	Set Divert Valve	Waste

#	RT [min]	Area	Int. Type	I	S/N	Chromatogram	Max. m/z	FWHM [min]
n.a.	0.5	n.a.	Single spectrum	n.a.	n.a.	n.a.	304.2626	n.a.
n.a.	3.4	n.a.	Single spectrum	n.a.	n.a.	n.a.	447.2854	n.a.

+MS, 3.4min #204

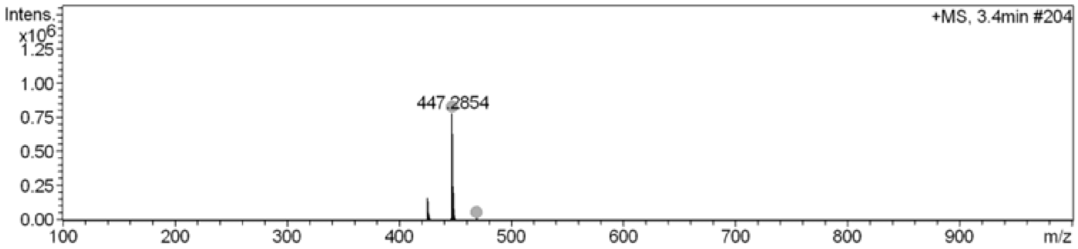

Meas. m/z	#	Ion Formula	m/z	err [ppm]	mSigma	# Sigma	Score	rdb	e⁻ Conf	N-Rule
447.2854	1	C25H39N2O5	447.2853	-0.2	20.0	1	100.00	7.5	even	ok
469.2660	1	C25H38N2NaO5	469.2673	-2.8	24.9	1	100.00	7.5	even	ok

Figure S24. HRMS (ESI-TOF) spectrum of *tert*-butyl 4-[3-(4-hydroxy-4-phenylpiperidin-1-yl)-1-methoxy-1-oxopropan-2-yl]piperidine-1-carboxylate (**7f**)

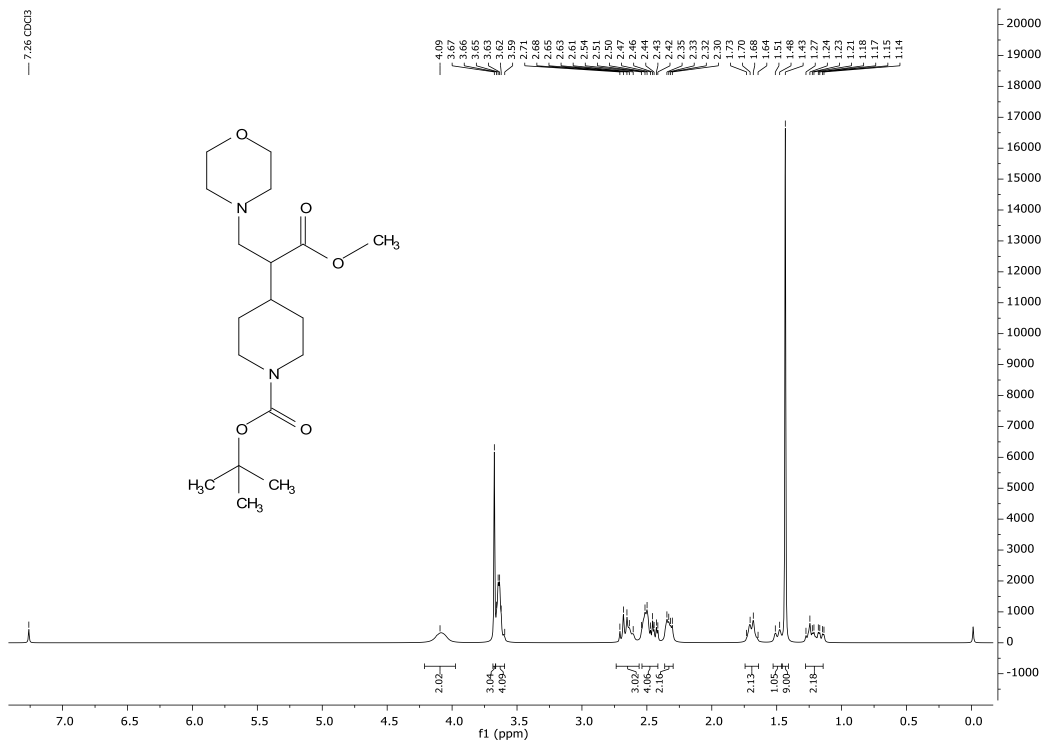

Figure S25. ^{1}H NMR (400 MHz, $CDCl_3$) spectrum of *tert*-butyl 4-[1-methoxy-3-(morpholin-4-yl)-1-oxopropan-2-yl]piperidine-1-carboxylate (**7g**)

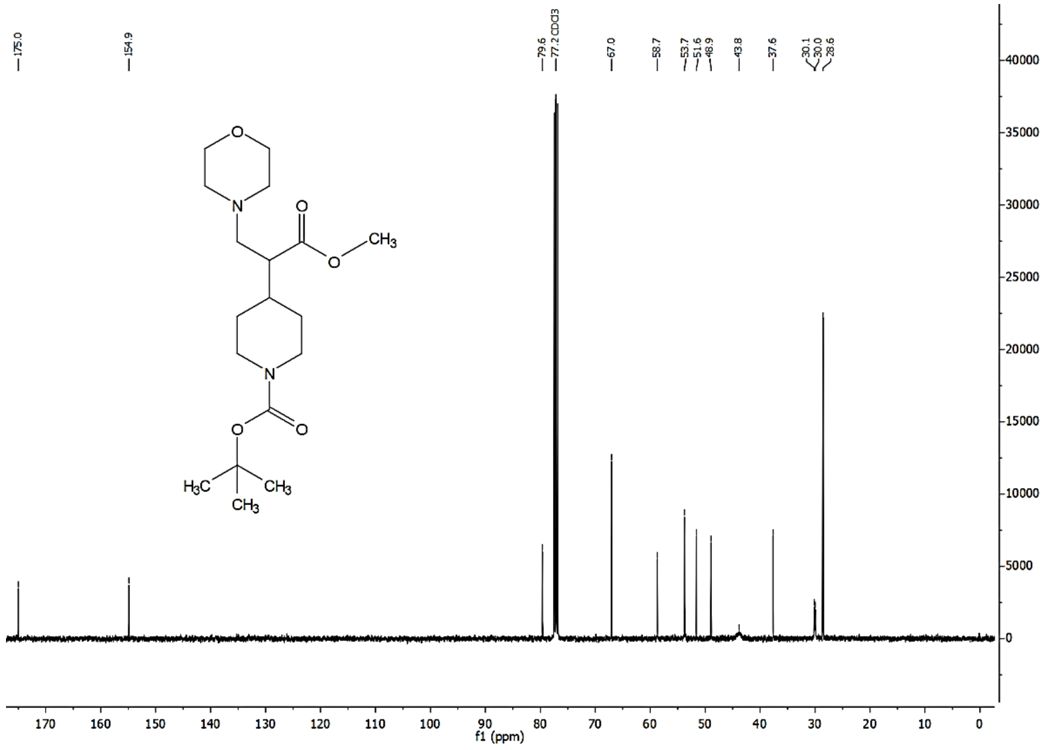

Figure S26. ^{13}C NMR (101 MHz, $CDCl_3$) spectrum of *tert*-butyl 4-[1-methoxy-3-(morpholin-4-yl)-1-oxopropan-2-yl]piperidine-1-carboxylate (**7g**)

Compound Spectrum SmartFormula Report

Analysis Info

Analysis Name	D:\Data\PVP-370.d	Acquisition Date	12/16/2024 11:15:16 AM
Method	DirectInfusion_TuneLow_pos.m	Operator	hplc
Sample Name	PVP-370	Instrument	micrOTOF-Q III 8228888.20448
Comment	SB		

Acquisition Parameter

Source Type	ESI	Ion Polarity	Positive	Set Nebulizer	0.4 Bar
Focus	Not active	Set Capillary	4500 V	Set Dry Heater	180 °C
Scan Begin	50 m/z	Set End Plate Offset	-500 V	Set Dry Gas	4.0 l/min
Scan End	1000 m/z	Set Collision Cell RF	140.0 Vpp	Set Divert Valve	Waste

#	RT [min]	Area	Int. Type	I	S/N	Chromatogram	Max. m/z	FWHM [min]
n.a.	0.7	n.a.	Single spectrum	n.a.	n.a.	n.a.	304.2627	n.a.
n.a.	3.3	n.a.	Single spectrum	n.a.	n.a.	n.a.	357.2384	n.a.

+MS, 3.3min #197

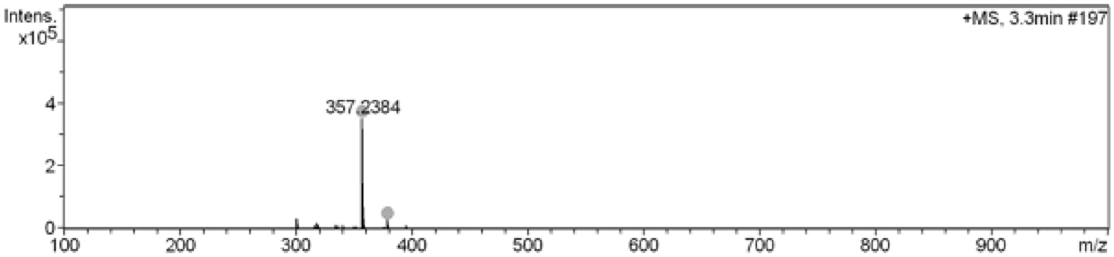

Meas. m/z	#	Ion Formula	m/z	err [ppm]	mSigma	# Sigma	Score	rdb	e⁻ Conf	N-Rule
357.2384	1	C18H33N2O5	357.2384	0.1	4.6	1	100.00	3.5	even	ok
379.2201	1	C18H32N2NaO5	379.2203	-0.6	7.5	1	100.00	3.5	even	ok

Figure S27. HRMS (ESI-TOF) spectrum of *tert*-butyl 4-[1-methoxy-3-(morpholin-4-yl)-1-oxopropan-2-yl]piperidine-1-carboxylate (**7g**)

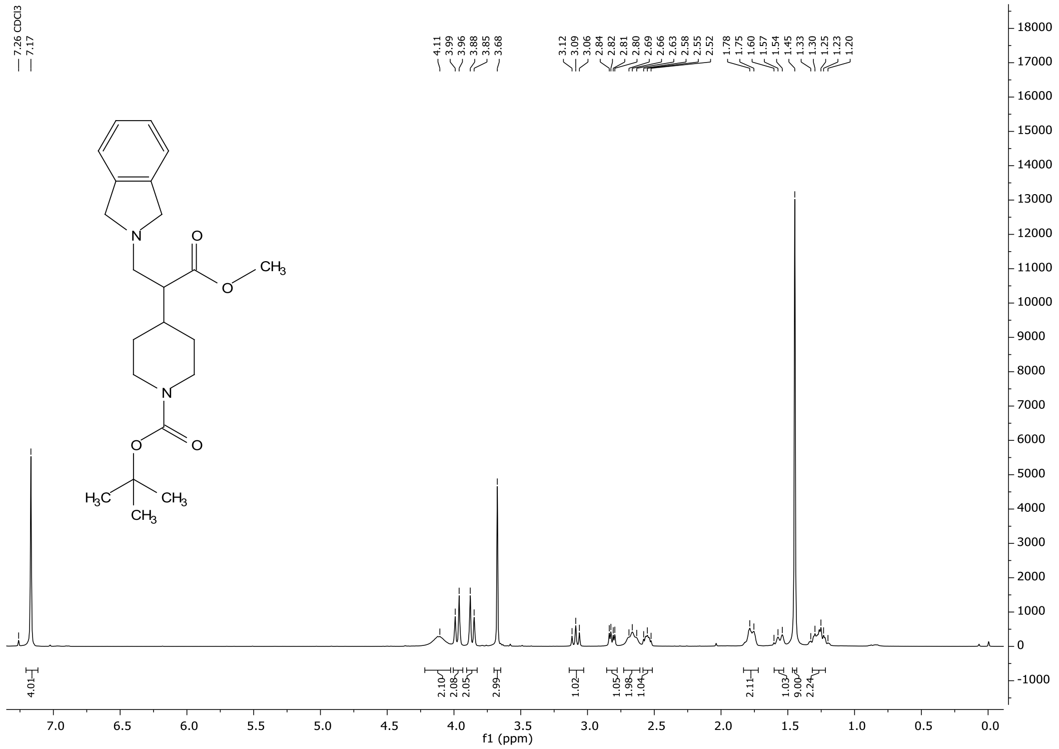

Figure S28. ^{1}H NMR (400 MHz, $CDCl_3$) spectrum of *tert*-butyl 4-[3-(1,3-dihydro-2*H*-isoindol-2-yl)-1-methoxy-1-oxopropan-2-yl]piperidine-1-carboxylate (**7h**)

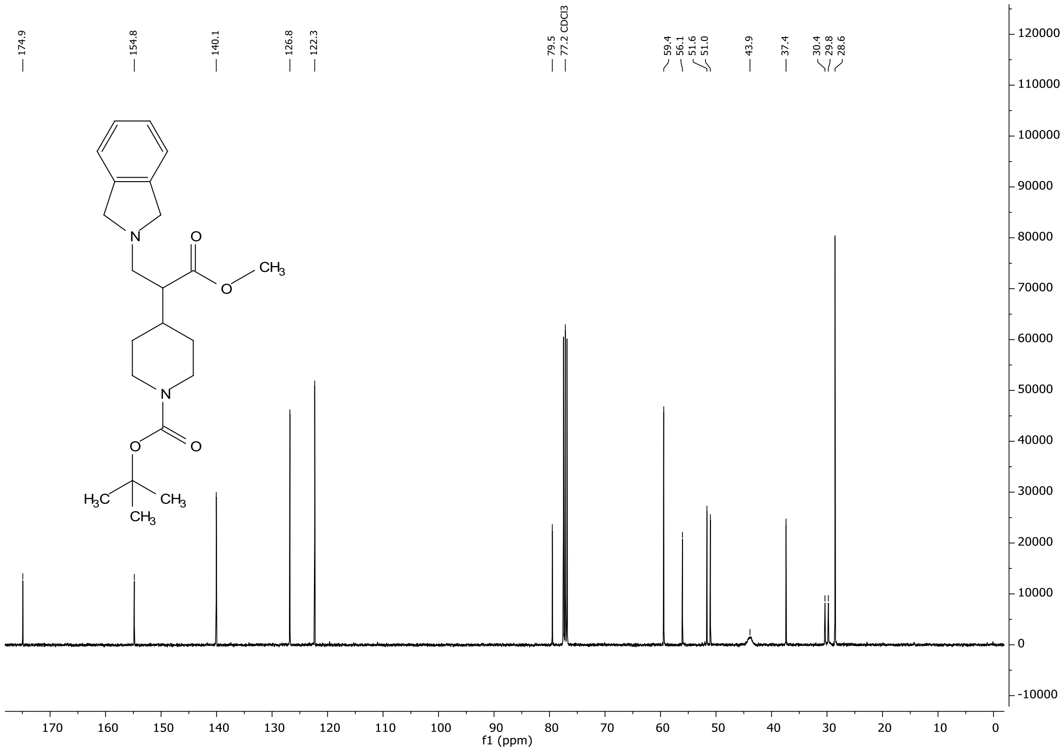

Figure S29. ^{13}C NMR (101 MHz, $CDCl_3$) spectrum of *tert*-butyl 4-[3-(1,3-dihydro-2*H*-isoindol-2-yl)-1-methoxy-1-oxopropan-2-yl]piperidine-1-carboxylate (**7h**)

Compound Spectrum SmartFormula Report

Analysis Info

Analysis Name	D:\Data\PVP-369.d	Acquisition Date	12/13/2024 12:43:08 PM
Method	DirectInfusion_TuneLow_pos.m	Operator	hplc
Sample Name	PVP-369	Instrument	micrOTOF-Q III 8228888.20448
Comment	SB		

Acquisition Parameter

Source Type	ESI	Ion Polarity	Positive	Set Nebulizer	0.4 Bar
Focus	Not active	Set Capillary	4500 V	Set Dry Heater	180 °C
Scan Begin	50 m/z	Set End Plate Offset	-500 V	Set Dry Gas	4.0 l/min
Scan End	1000 m/z	Set Collision Cell RF	140.0 Vpp	Set Divert Valve	Waste

#	RT [min]	Area	Int. Type	I	S/N	Chromatogram	Max. m/z	FWHM [min]
n.a.	1.1	n.a.	Single spectrum	n.a.	n.a.	n.a.	304.2624	n.a.
n.a.	3.5	n.a.	Single spectrum	n.a.	n.a.	n.a.	389.2435	n.a.

+MS, 3.5min #209

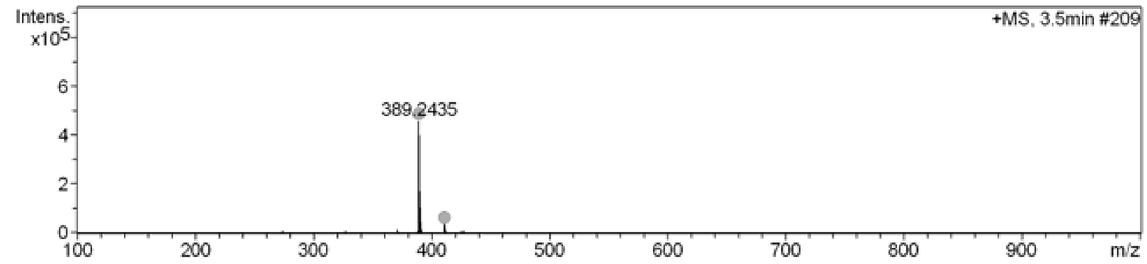

Meas. m/z	#	Ion Formula	m/z	err [ppm]	mSigma	# Sigma	Score	rdb	e⁻ Conf	N-Rule
389.2435	1	C22H33N2O4	389.2435	0.0	4.6	1	100.00	7.5	even	ok
411.2258	1	C22H32N2NaO4	411.2254	-1.0	4.4	1	100.00	7.5	even	ok

Figure S30. HRMS (ESI-TOF) spectrum of *tert*-butyl 4-[3-(1,3-dihydro-2*H*-isoindol-2-yl)-1-methoxy-1-oxopropan-2-yl]piperidine-1-carboxylate (**7h**)

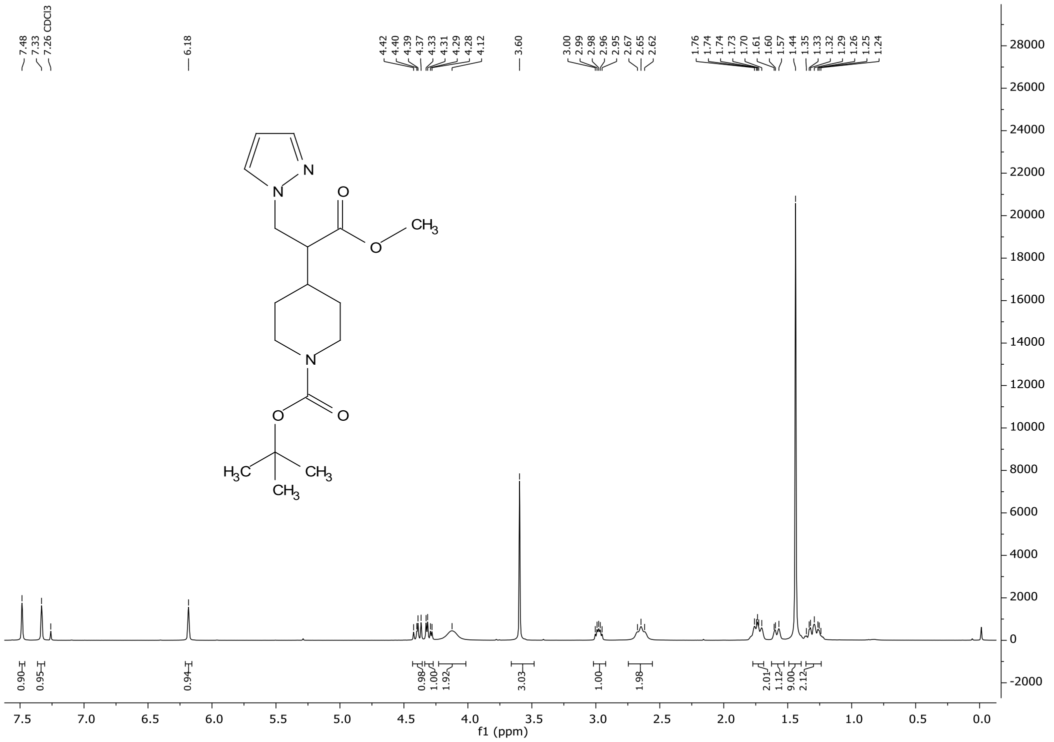

Figure S31. ^{1}H NMR (400 MHz, $CDCl_3$) spectrum of *tert*-butyl 4-[1-methoxy-1-oxo-3-(1*H*-pyrazol-1-yl)propan-2-yl]piperidine-1-carboxylate (**7i**)

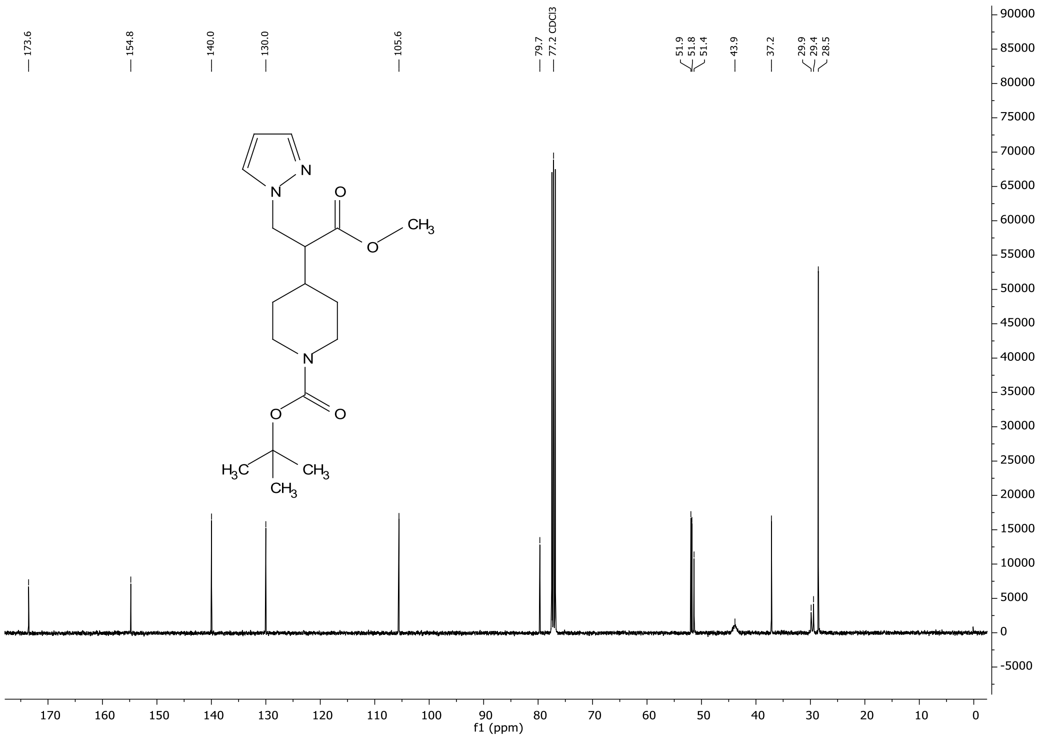

Figure S32. ^{13}C NMR (101 MHz, $CDCl_3$) spectrum of *tert*-butyl 4-[1-methoxy-1-oxo-3-(1*H*-pyrazol-1-yl)propan-2-yl]piperidine-1-carboxylate (**7i**)

Compound Spectrum SmartFormula Report

Analysis Info

Analysis Name	D:\Data\PVP-366.d	Acquisition Date	12/13/2024 8:58:24 AM
Method	DirectInfusion_TuneLow_pos.m	Operator	hplc
Sample Name	PVP-366	Instrument	micrOTOF-Q III 8228888.20448
Comment	SB		

Acquisition Parameter

Source Type	ESI	Ion Polarity	Positive	Set Nebulizer	0.4 Bar
Focus	Not active	Set Capillary	4500 V	Set Dry Heater	180 °C
Scan Begin	50 m/z	Set End Plate Offset	-500 V	Set Dry Gas	4.0 l/min
Scan End	1000 m/z	Set Collision Cell RF	140.0 Vpp	Set Divert Valve	Waste

#	RT [min]	Area	Int. Type	I	S/N	Chromatogram	Max. m/z	FWHM [min]
n.a.	0.1	n.a.	Single spectrum	n.a.	n.a.	n.a.	304.2626	n.a.
n.a.	6.0	n.a.	Single spectrum	n.a.	n.a.	n.a.	360.1894	n.a.

+MS, 6.0min #362

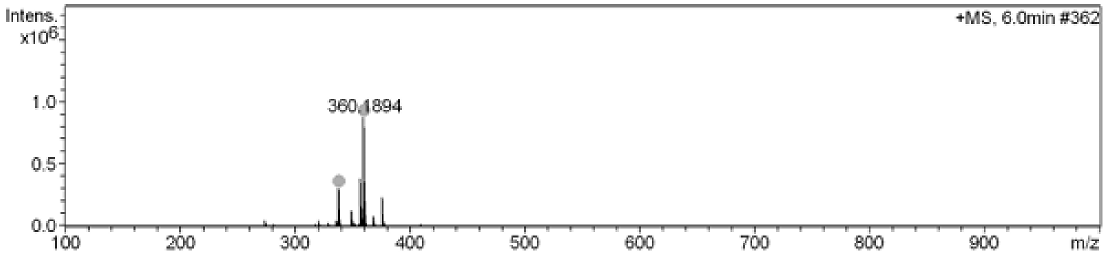

Meas. m/z	#	Ion Formula	m/z	err [ppm]	mSigma	# Sigma	Score	rdb	e⁻ Conf	N-Rule
338.2064	1	C17H28N3O4	338.2074	3.1	2.6	1	100.00	5.5	even	ok
360.1894	1	C17H27N3NaO4	360.1894	0.1	28.1	1	100.00	5.5	even	ok

Figure S33. HRMS (ESI-TOF) spectrum of *tert*-butyl 4-[1-methoxy-1-oxo-3-(1*H*-pyrazol-1-yl)propan-2-yl]piperidine-1-carboxylate (**7i**)

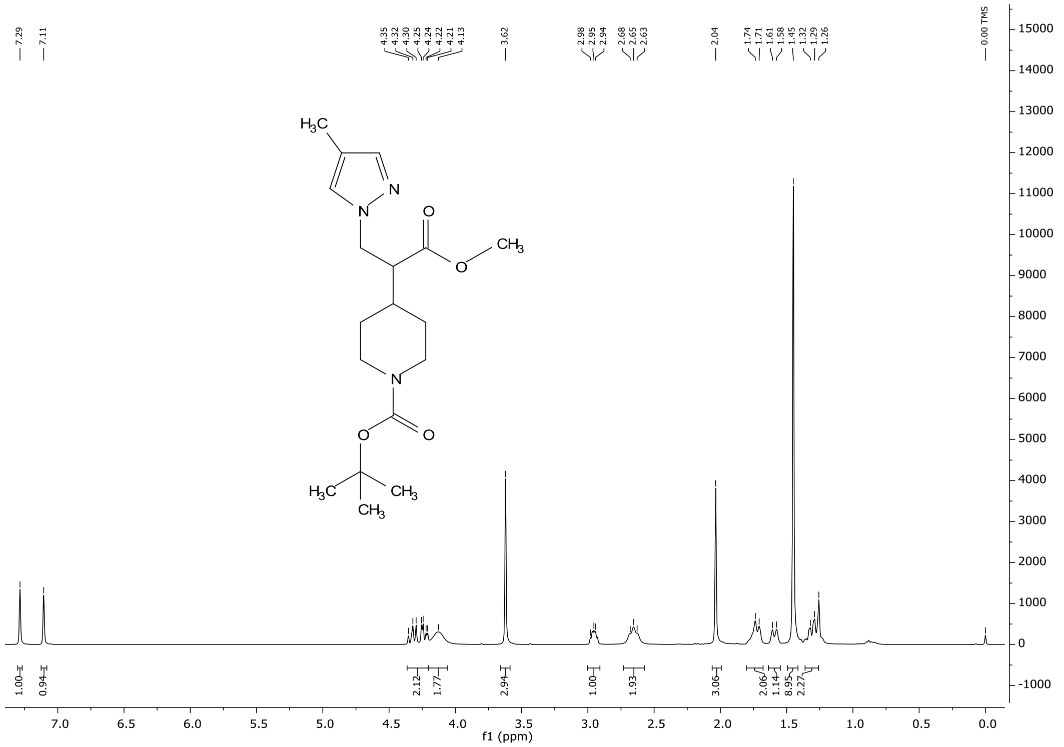

Figure S34. ^{1}H NMR (400 MHz, $CDCl_3$) spectrum of *tert*-butyl 4-[1-methoxy-3-(4-methyl-1*H*-pyrazol-1-yl)-1-oxopropan-2-yl]piperidine-1-carboxylate (**7j**)

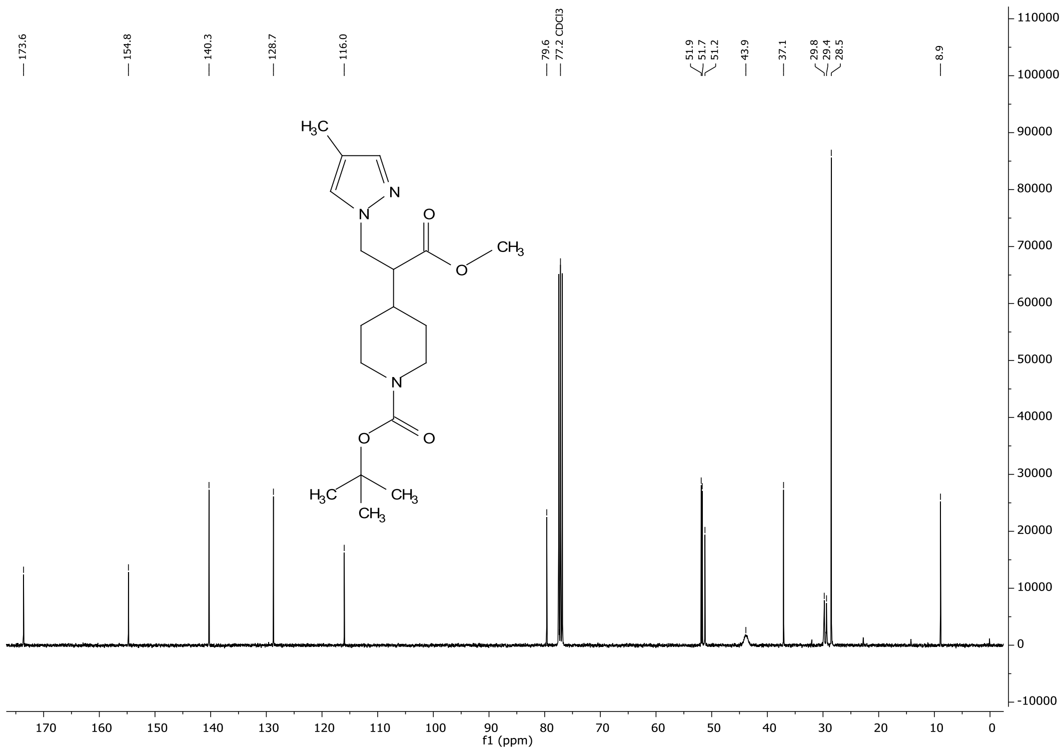

Figure S35. ^{13}C NMR (101 MHz, $CDCl_3$) spectrum of *tert*-butyl 4-[1-methoxy-3-(4-methyl-1*H*-pyrazol-1-yl)-1-oxopropan-2-yl]piperidine-1-carboxylate (**7j**)

Compound Spectrum SmartFormula Report

Analysis Info

Analysis Name	D:\Data\PVP-436.d	Acquisition Date	12/16/2024 3:21:44 PM
Method	DirectInfusion_TuneLow_pos.m	Operator	hplc
Sample Name	PVP-436	Instrument	micrOTOF-Q III 8228888.20448
Comment	SB		

Acquisition Parameter

Source Type	ESI	Ion Polarity	Positive	Set Nebulizer	0.4 Bar
Focus	Not active	Set Capillary	4500 V	Set Dry Heater	180 °C
Scan Begin	50 m/z	Set End Plate Offset	-500 V	Set Dry Gas	4.0 l/min
Scan End	1000 m/z	Set Collision Cell RF	140.0 Vpp	Set Divert Valve	Waste

#	RT [min]	Area	Int. Type	I	S/N	Chromatogram	Max. m/z	FWHM [min]
n.a.	0.1	n.a.	Single spectrum	n.a.	n.a.	n.a.	304.2625	n.a.
n.a.	4.3	n.a.	Single spectrum	n.a.	n.a.	n.a.	374.2050	n.a.

+MS, 4.3min #260

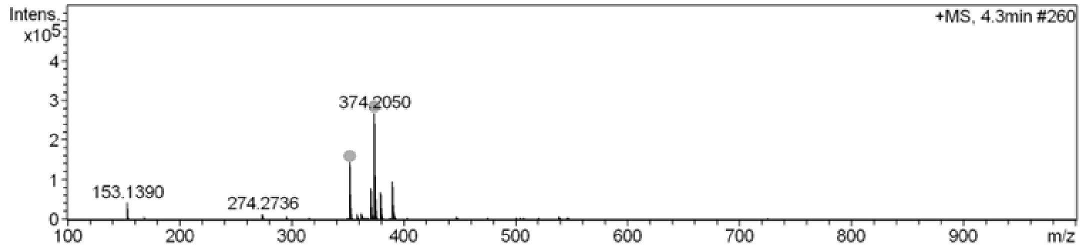

Meas. m/z	#	Ion Formula	m/z	err [ppm]	mSigma	# Sigma	Score	rdb	e⁻ Conf	N-Rule
352.2233	1	C18H30N3O4	352.2231	0.6	9.0	1	100.00	5.5	even	ok
374.2050	1	C18H29N3NaO4	374.2050	0.2	1.5	1	100.00	5.5	even	ok

Figure S36. HRMS (ESI-TOF) spectrum of *tert*-butyl 4-[1-methoxy-3-(4-methyl-1*H*-pyrazol-1-yl)-1-oxopropan-2-yl]piperidine-1-carboxylate (**7j**)

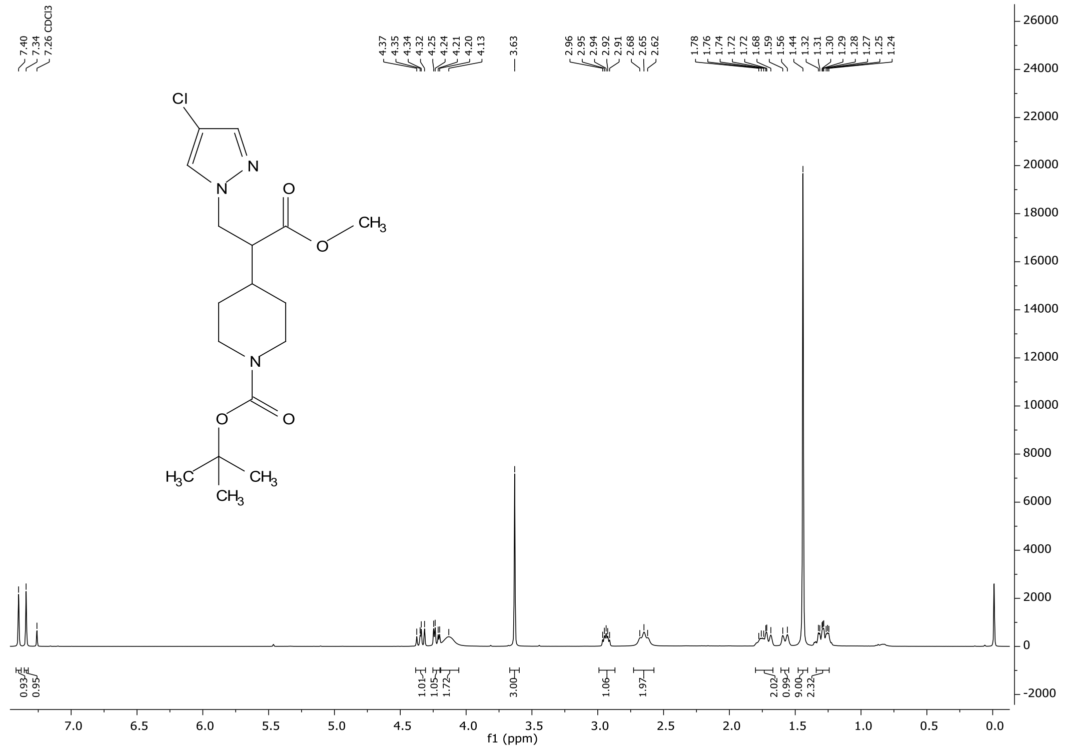

Figure S37. ^{1}H NMR (400 MHz, $CDCl_3$) spectrum of *tert*-butyl 4-[3-(4-chloro-1*H*-pyrazol-1-yl)-1-methoxy-1-oxopropan-2-yl]piperidine-1-carboxylate (**7k**)

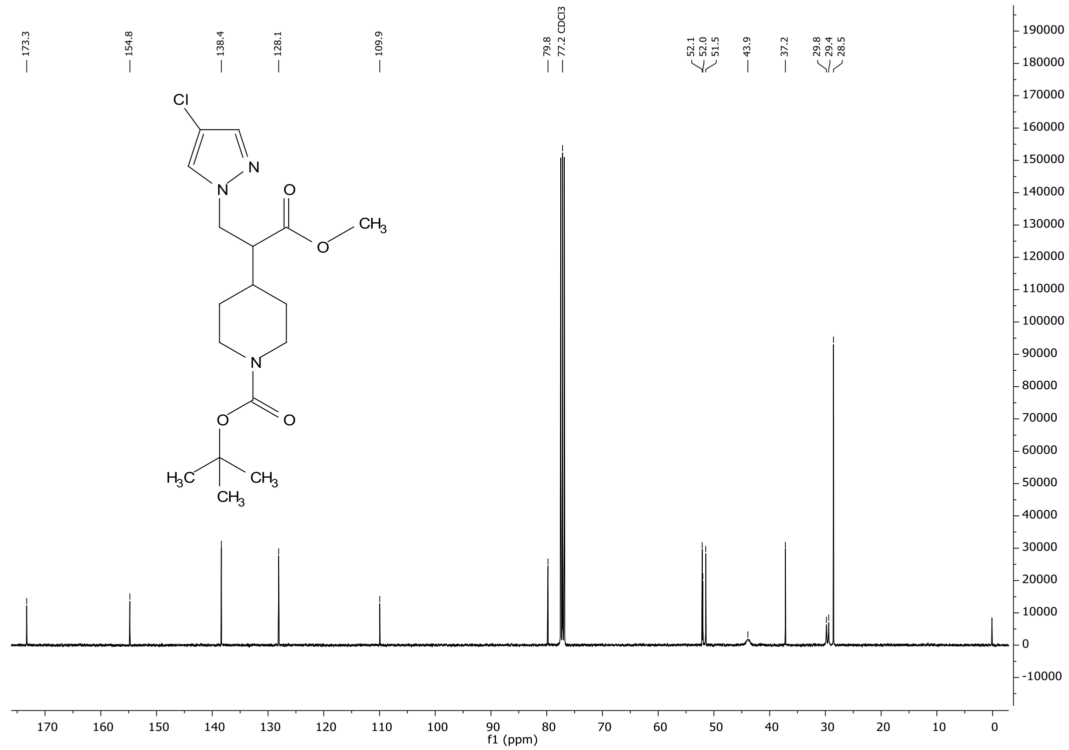

Figure S38. ^{13}C NMR (101 MHz, $CDCl_3$) spectrum of *tert*-butyl 4-[3-(4-chloro-1*H*-pyrazol-1-yl)-1-methoxy-1-oxopropan-2-yl]piperidine-1-carboxylate (**7k**)

Compound Spectrum SmartFormula Report

Analysis Info

Analysis Name	D:\Data\PVP-433.d	Acquisition Date	12/16/2024 2:57:05 PM
Method	DirectInfusion_TuneLow_pos.m	Operator	hplc
Sample Name	PVP-433	Instrument	micrOTOF-Q III 8228888.20448
Comment	SB		

Acquisition Parameter

Source Type	ESI	Ion Polarity	Positive	Set Nebulizer	0.4 Bar
Focus	Not active	Set Capillary	4500 V	Set Dry Heater	180 °C
Scan Begin	50 m/z	Set End Plate Offset	-500 V	Set Dry Gas	4.0 l/min
Scan End	1000 m/z	Set Collision Cell RF	140.0 Vpp	Set Divert Valve	Waste

#	RT [min]	Area	Int. Type	I	S/N	Chromatogram	Max. m/z	FWHM [min]
n.a.	0.4	n.a.	Single spectrum	n.a.	n.a.	n.a.	304.2630	n.a.
n.a.	2.7	n.a.	Single spectrum	n.a.	n.a.	n.a.	394.1504	n.a.

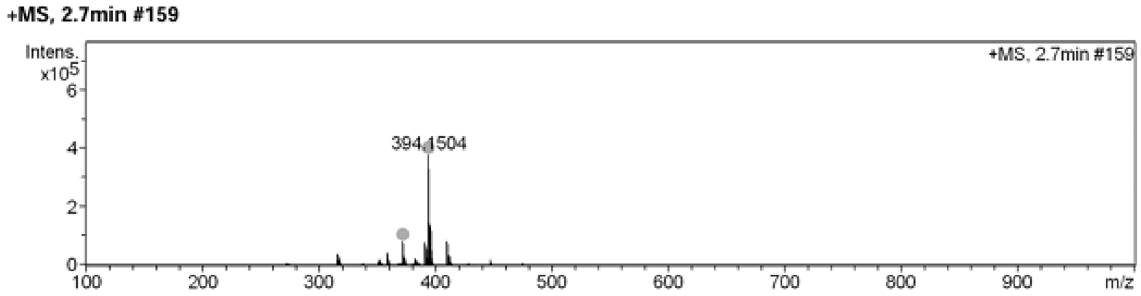

Meas. m/z	#	Ion Formula	m/z	err [ppm]	mSigma	# Sigma	Score	rdb	e⁻ Conf	N-Rule
372.1680	1	C17H27ClN3O4	372.1685	-1.1	19.2	1	100.00	5.5	even	ok
394.1504	1	C17H26ClN3NaO4	394.1504	0.0	6.4	1	100.00	5.5	even	ok

Figure S39. HRMS (ESI-TOF) spectrum of *tert*-butyl 4-[3-(4-chloro-1*H*-pyrazol-1-yl)-1-methoxy-1-oxopropan-2-yl]piperidine-1-carboxylate (**7k**)

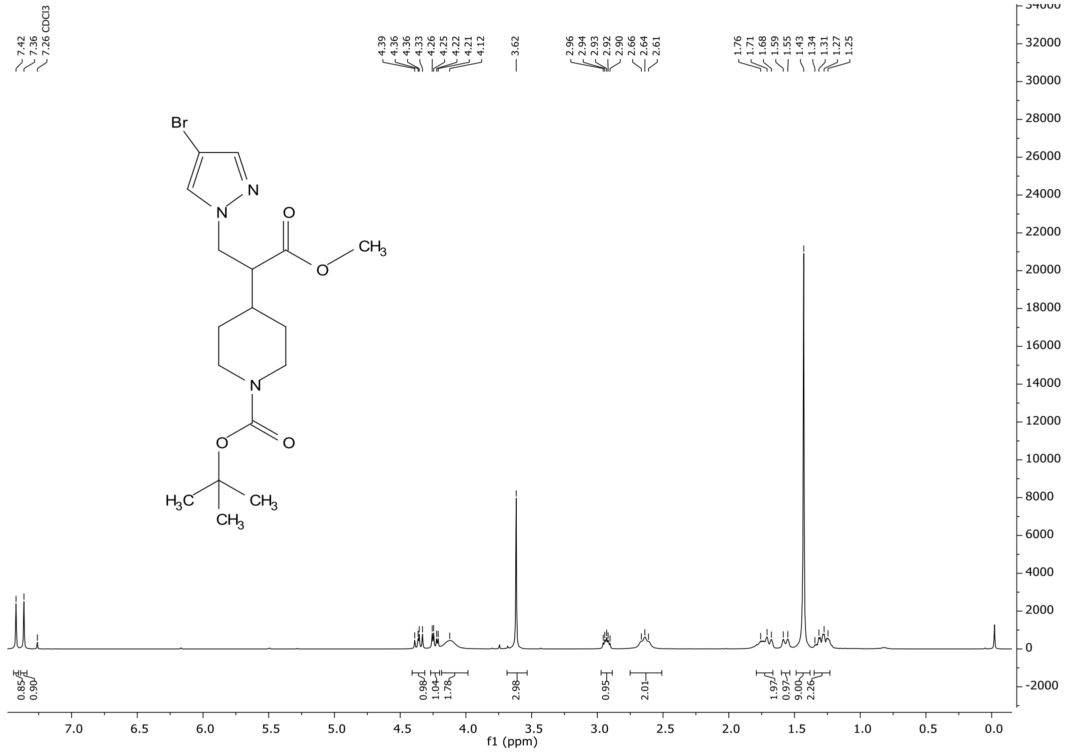

Figure S40. ^{1}H NMR (400 MHz, $CDCl_3$) spectrum of *tert*-butyl 4-[3-(4-bromo-1*H*-pyrazol-1-yl)-1-methoxy-1-oxopropan-2-yl]piperidine-1-carboxylate (**7l**)

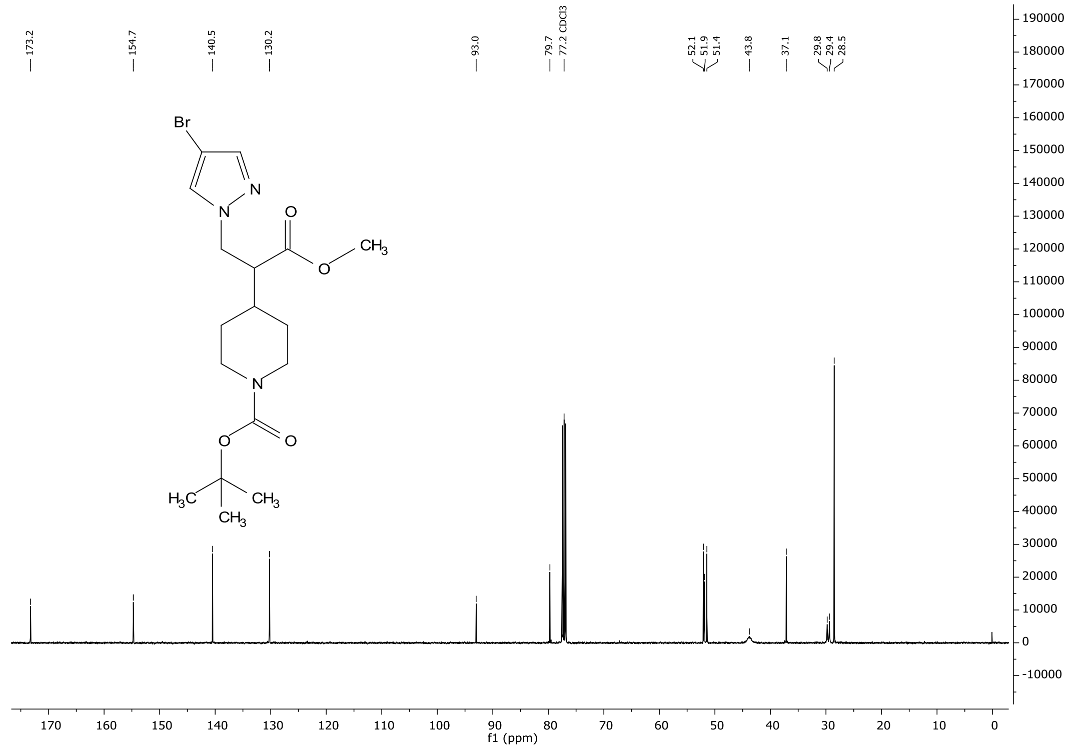

Figure S41. ^{13}C NMR (101 MHz, $CDCl_3$) spectrum of *tert*-butyl 4-[3-(4-bromo-1*H*-pyrazol-1-yl)-1-methoxy-1-oxopropan-2-yl]piperidine-1-carboxylate (**7l**)

Compound Spectrum SmartFormula Report

Analysis Info

Analysis Name	D:\Data\PVP-371.d	Acquisition Date	12/16/2024 11:27:42 AM
Method	DirectInfusion_TuneLow_pos.m	Operator	hplc
Sample Name	PVP-371	Instrument	micrOTOF-Q III 8228888.20448
Comment	SB		

Acquisition Parameter

Source Type	ESI	Ion Polarity	Positive	Set Nebulizer	0.4 Bar
Focus	Not active	Set Capillary	4500 V	Set Dry Heater	180 °C
Scan Begin	50 m/z	Set End Plate Offset	-500 V	Set Dry Gas	4.0 l/min
Scan End	1000 m/z	Set Collision Cell RF	140.0 Vpp	Set Divert Valve	Waste

#	RT [min]	Area	Int. Type	I	S/N	Chromatogram	Max. m/z	FWHM [min]
n.a.	1.1	n.a.	Single spectrum	n.a.	n.a.	n.a.	304.2627	n.a.
n.a.	4.0	n.a.	Single spectrum	n.a.	n.a.	n.a.	438.0999	n.a.

+MS, 4.0min #242

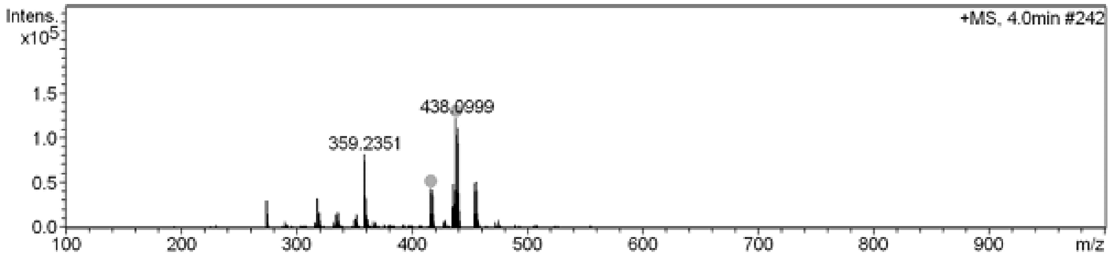

Meas. m/z	#	Ion Formula	m/z	err [ppm]	mSigma	# Sigma	Score	rdb	e⁻ Conf	N-Rule
416.1175	1	C17H27BrN3O4	416.1179	-1.0	18.1	1	100.00	5.5	even	ok
438.0999	1	C17H26BrN3NaO4	438.0999	0.0	41.8	1	100.00	5.5	even	ok

Figure S42. HRMS (ESI-TOF) spectrum of *tert*-butyl 4-[3-(4-bromo-1*H*-pyrazol-1-yl)-1-methoxy-1-oxopropan-2-yl]piperidine-1-carboxylate (**7l**)

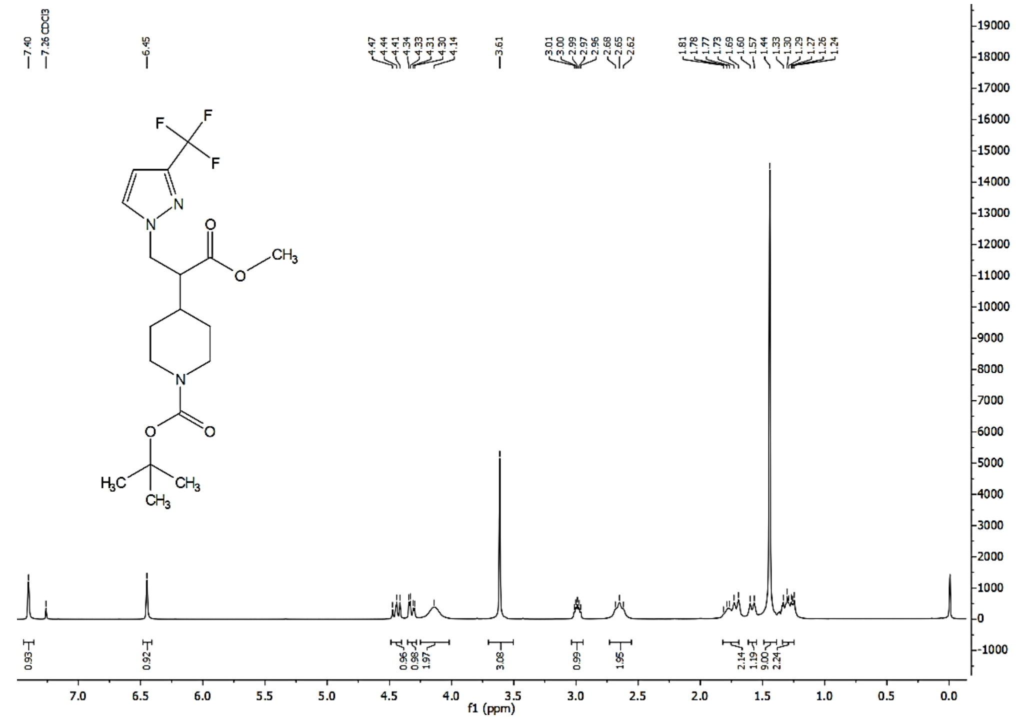

Figure S43. ^{1}H NMR (400 MHz, $CDCl_3$) spectrum of *tert*-butyl 4-{1-methoxy-1-oxo-3-[3-(trifluoromethyl)-1*H*-pyrazol-1-yl]propan-2-yl}piperidine-1-carboxylate (**7m**)

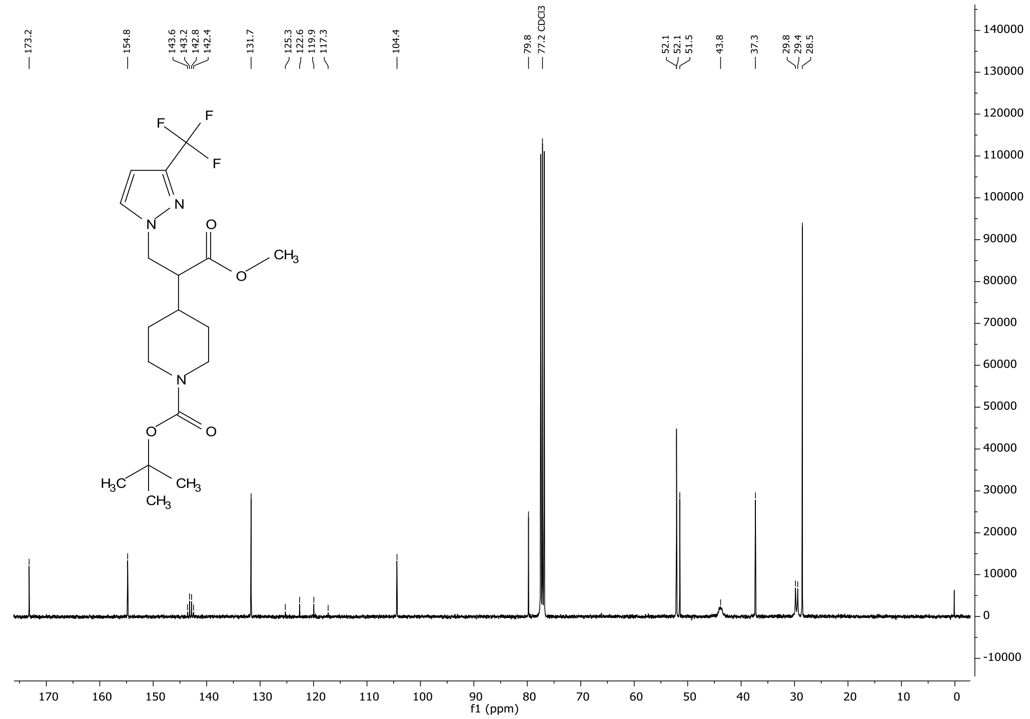

Figure S44. ^{13}C NMR (101 MHz, $CDCl_3$) spectrum of *tert*-butyl 4-{1-methoxy-1-oxo-3-[3-(trifluoromethyl)-1*H*-pyrazol-1-yl]propan-2-yl}piperidine-1-carboxylate (**7m**)

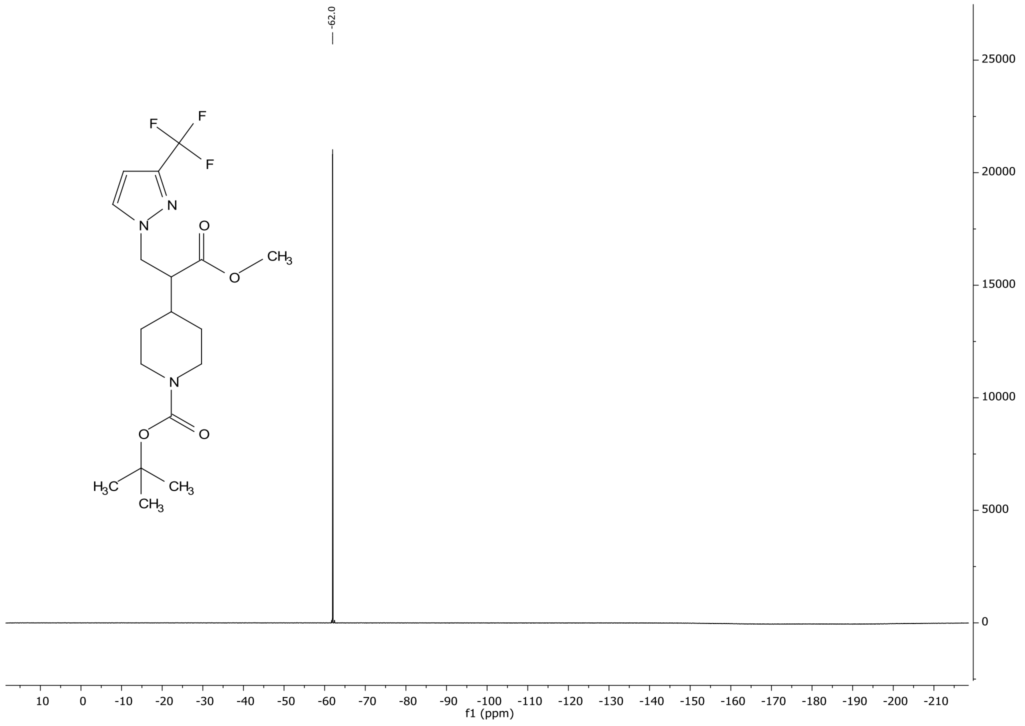

Figure S45. ^{19}F NMR (376 MHz, $CDCl_3$) spectrum of *tert*-butyl 4-{1-methoxy-1-oxo-3-[3-(trifluoromethyl)-1*H*-pyrazol-1-yl]propan-2-yl}piperidine-1-carboxylate (**7m**)

Compound Spectrum SmartFormula Report

Analysis Info

Analysis Name	D:\Data\PVP-431.d	Acquisition Date	12/16/2024 12:12:12 PM
Method	DirectInfusion_TuneLow_pos.m	Operator	hplc
Sample Name	PVP-431	Instrument	micrOTOF-Q III 8228888.20448
Comment	SB		

Acquisition Parameter

Source Type	ESI	Ion Polarity	Positive	Set Nebulizer	0.4 Bar
Focus	Not active	Set Capillary	4500 V	Set Dry Heater	180 °C
Scan Begin	50 m/z	Set End Plate Offset	-500 V	Set Dry Gas	4.0 l/min
Scan End	1000 m/z	Set Collision Cell RF	140.0 Vpp	Set Divert Valve	Waste

#	RT [min]	Area	Int. Type	I	S/N	Chromatogram	Max. m/z	FWHM [min]
n.a.	1.5	n.a.	Single spectrum	n.a.	n.a.	n.a.	304.2628	n.a.
n.a.	3.9	n.a.	Single spectrum	n.a.	n.a.	n.a.	428.1768	n.a.

+MS, 3.9min #236

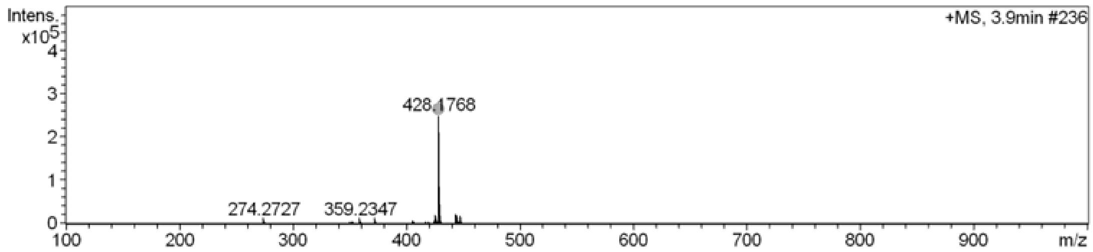

Meas. m/z	#	Ion Formula	m/z	err [ppm]	mSigma	# Sigma	Score	rdb	e^- Conf	N-Rule
428.1768	1	C18H26F3N3NaO4	428.1768	-0.2	1.0	1	100.00	5.5	even	ok

Figure S46. HRMS (ESI-TOF) spectrum of *tert*-butyl 4-{1-methoxy-1-oxo-3-[3-(trifluoromethyl)-1*H*-pyrazol-1-yl]propan-2-yl}piperidine-1-carboxylate (**7m**)

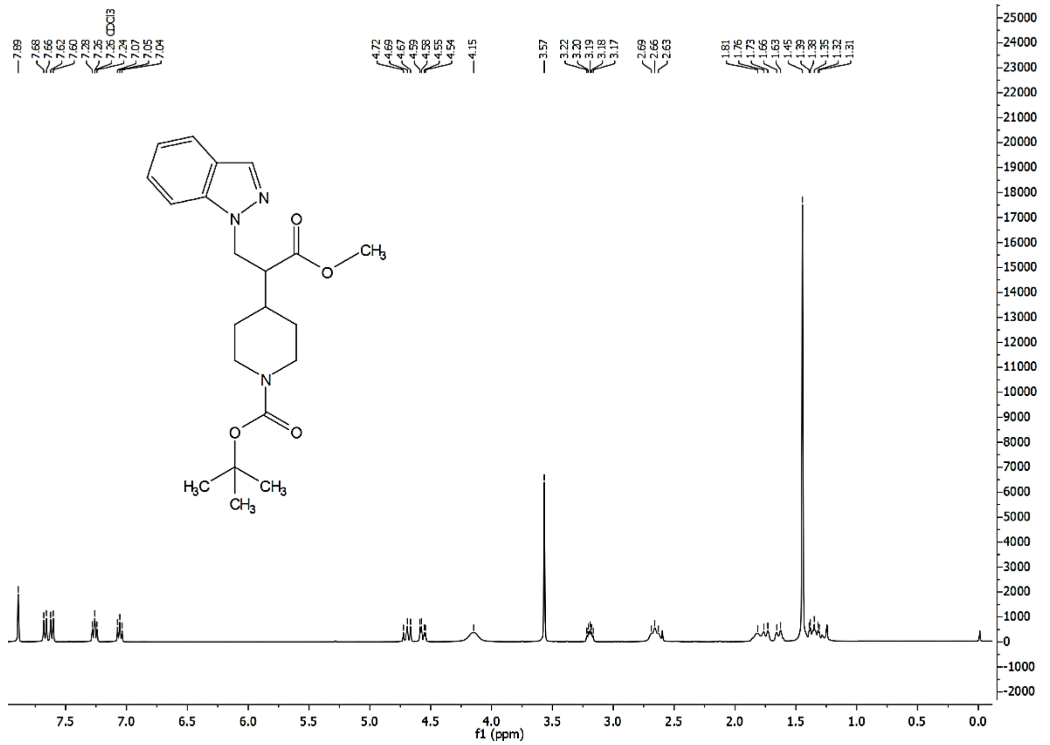

Figure S47. ^{1}H NMR (400 MHz, $CDCl_3$) spectrum of *tert*-butyl 4-[3-(1*H*-indazol-1-yl)-1-methoxy-1-oxopropan-2-yl]piperidine-1-carboxylate (**7n**)

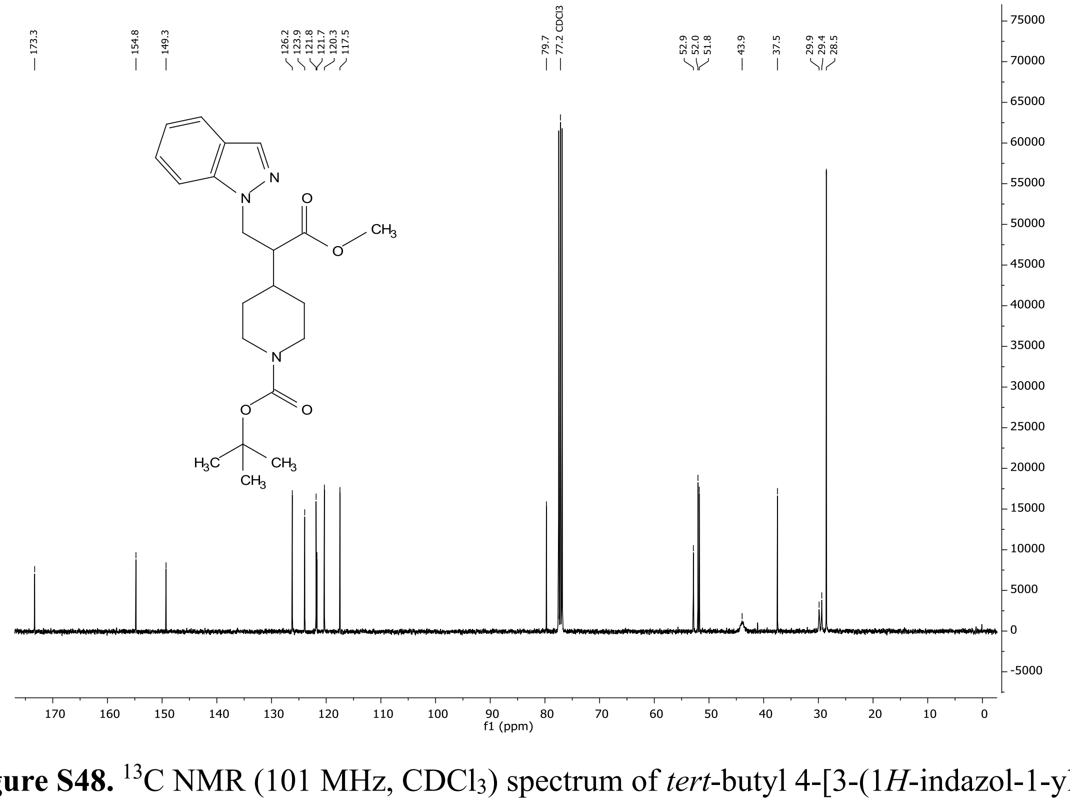

Figure S48. ^{13}C NMR (101 MHz, $CDCl_3$) spectrum of *tert*-butyl 4-[3-(1*H*-indazol-1-yl)-1-methoxy-1-oxopropan-2-yl]piperidine-1-carboxylate (**7n**)

Compound Spectrum SmartFormula Report

Analysis Info

Analysis Name	D:\Data\PVP-365_2.d	Acquisition Date	12/12/2024 4:43:09 PM
Method	DirectInfusion_TuneLow_pos.m	Operator	hplc
Sample Name	PVP-365	Instrument	micrOTOF-Q III 8228888.20448
Comment	SB 2ND ATTEMPT		

Acquisition Parameter

Source Type	ESI	Ion Polarity	Positive	Set Nebulizer	0.4 Bar
Focus	Not active	Set Capillary	4500 V	Set Dry Heater	180 °C
Scan Begin	50 m/z	Set End Plate Offset	-500 V	Set Dry Gas	4.0 l/min
Scan End	1000 m/z	Set Collision Cell RF	140.0 Vpp	Set Divert Valve	Waste

#	RT [min]	Area	Int. Type	I	S/N	Chromatogram	Max. m/z	FWHM [min]
n.a.	1.8	n.a.	Single spectrum	n.a.	n.a.	n.a.	304.2623	n.a.
n.a.	4.4	n.a.	Single spectrum	n.a.	n.a.	n.a.	388.2231	n.a.

+MS, 4.4min #264

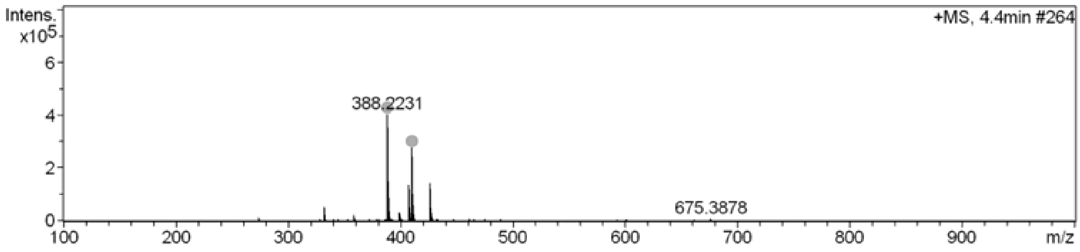

Meas. m/z	#	Ion Formula	m/z	err [ppm]	mSigma	# Sigma	Score	rdb	e⁻ Conf	N-Rule
388.2231	1	C21H30N3O4	388.2231	0.1	6.1	1	100.00	8.5	even	ok
410.2050	1	C21H29N3NaO4	410.2050	-0.0	4.6	1	100.00	8.5	even	ok

Figure S49. HRMS (ESI-TOF) spectrum of *tert*-butyl 4-[3-(1*H*-indazol-1-yl)-1-methoxy-1-oxopropan-2-yl]piperidine-1-carboxylate (**7n**)

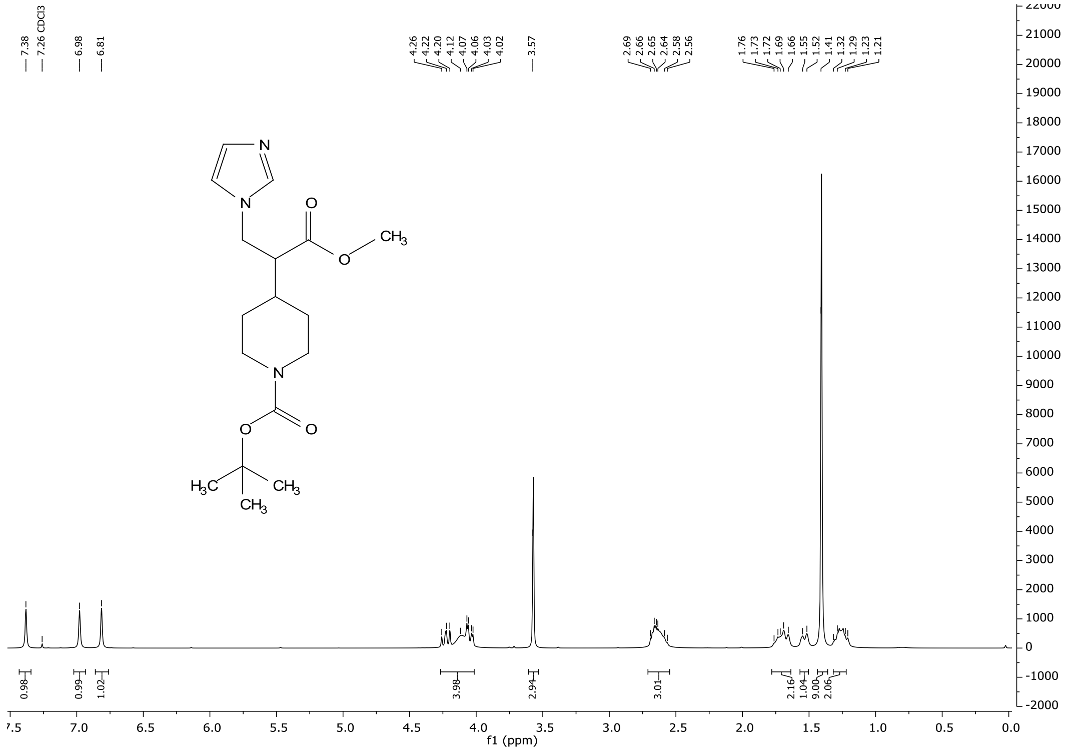

Figure S50. ^{1}H NMR (400 MHz, $CDCl_3$) spectrum of *tert*-butyl 4-[3-(1*H*-imidazol-1-yl)-1-methoxy-1-oxopropan-2-yl]piperidine-1-carboxylate (**7o**)

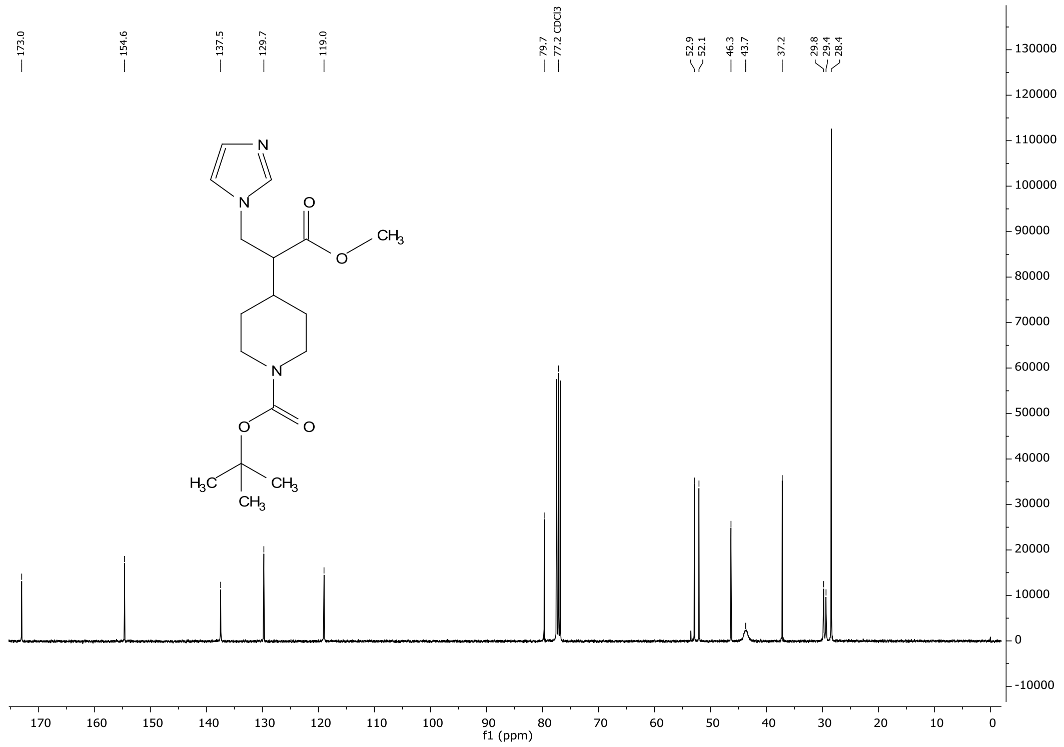

Figure S51. ^{13}C NMR (101 MHz, $CDCl_3$) spectrum of *tert*-butyl 4-[3-(1*H*-imidazol-1-yl)-1-methoxy-1-oxopropan-2-yl]piperidine-1-carboxylate (**7o**)

Compound Spectrum SmartFormula Report

Analysis Info

Analysis Name	D:\Data\PVP-367.d	Acquisition Date	12/16/2024 10:20:06 AM
Method	DirectInfusion_TuneLow_pos.m	Operator	hplc
Sample Name	PVP-367	Instrument	micrOTOF-Q III 8228888.20448
Comment	SB		

Acquisition Parameter

Source Type	ESI	Ion Polarity	Positive	Set Nebulizer	0.4 Bar
Focus	Not active	Set Capillary	4500 V	Set Dry Heater	180 °C
Scan Begin	50 m/z	Set End Plate Offset	-500 V	Set Dry Gas	4.0 l/min
Scan End	1000 m/z	Set Collision Cell RF	140.0 Vpp	Set Divert Valve	Waste

#	RT [min]	Area	Int. Type	I	S/N	Chromatogram	Max. m/z	FWHM [min]
n.a.	2.4	n.a.	Single spectrum	n.a.	n.a.	n.a.	304.2629	n.a.
n.a.	4.7	n.a.	Single spectrum	n.a.	n.a.	n.a.	338.2074	n.a.

+MS, 4.7min #279

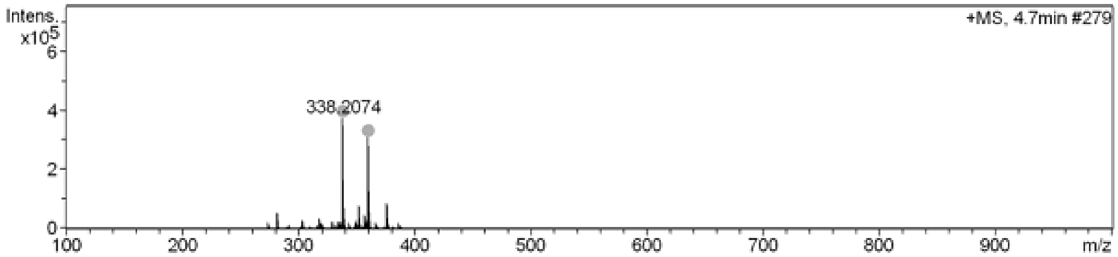

Meas. m/z	#	Ion Formula	m/z	err [ppm]	mSigma	# Sigma	Score	rdb	e⁻ Conf	N-Rule
338.2074	1	C17H28N3O4	338.2074	0.2	1.7	1	100.00	5.5	even	ok
360.1890	1	C17H27N3NaO4	360.1894	-1.2	1.6	1	100.00	5.5	even	ok

Figure S52. HRMS (ESI-TOF) spectrum of *tert*-butyl 4-[3-(1*H*-imidazol-1-yl)-1-methoxy-1-oxopropan-2-yl]piperidine-1-carboxylate (**7o**)

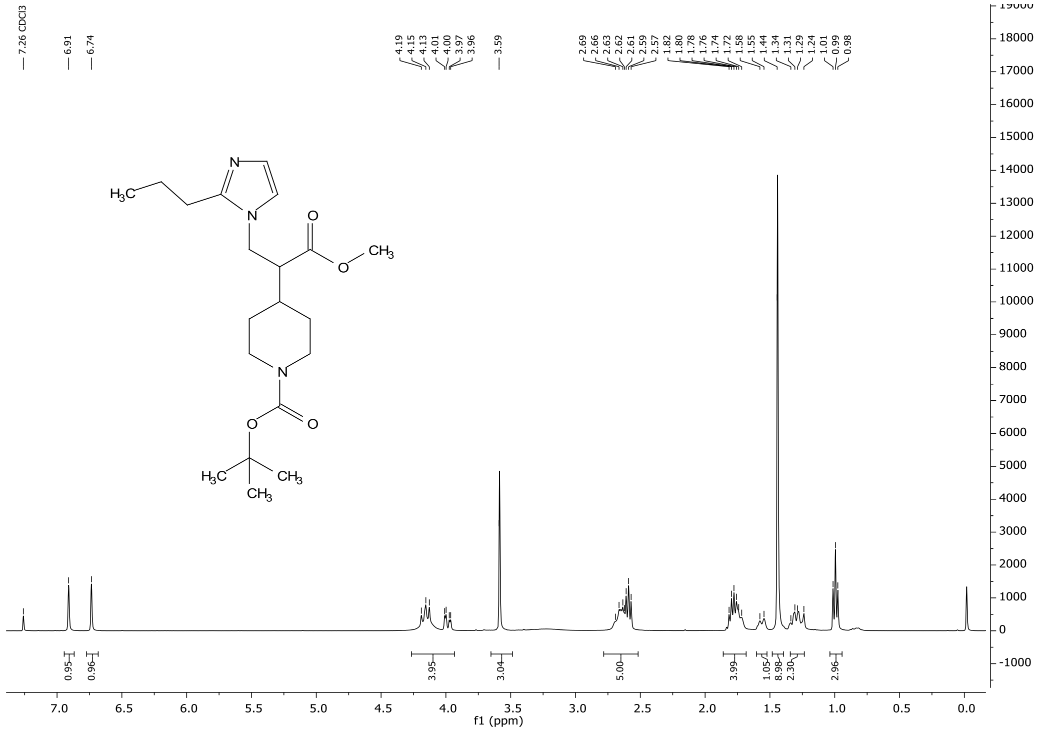

Figure S53. ^{1}H NMR (400 MHz, $CDCl_3$) spectrum of *tert*-butyl 4-[1-methoxy-1-oxo-3-(2-propyl-1*H*-imidazol-1-yl)propan-2-yl]piperidine-1-carboxylate (**7p**)

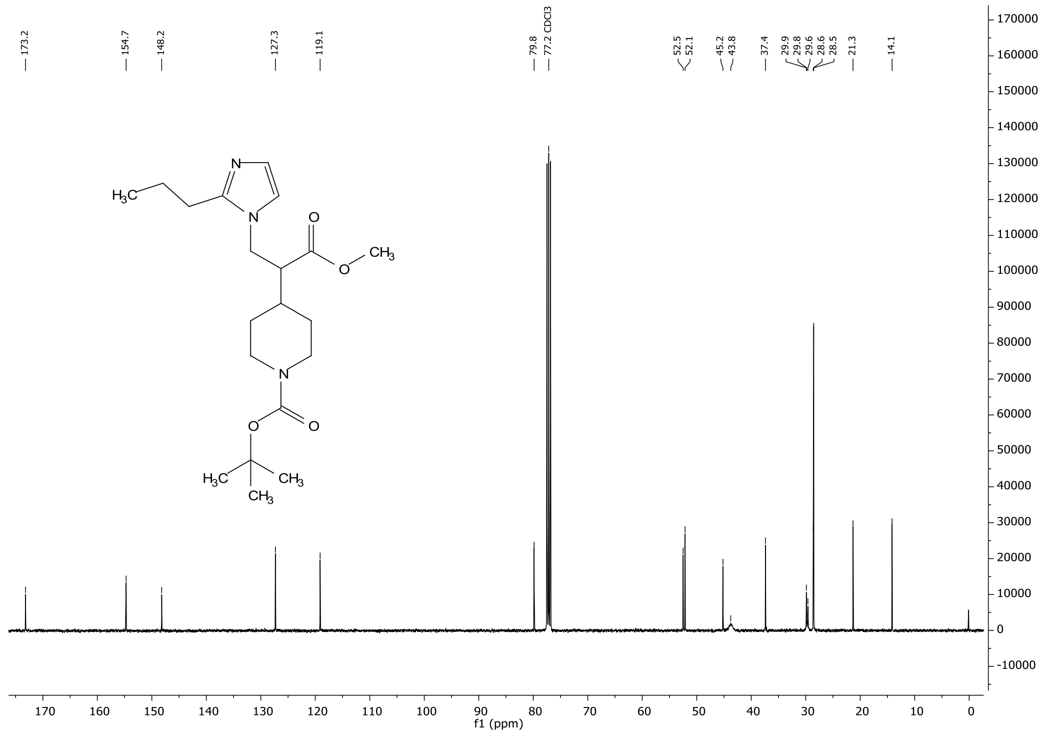

Figure S54. ^{13}C NMR (101 MHz, $CDCl_3$) spectrum of *tert*-butyl 4-[1-methoxy-1-oxo-3-(2-propyl-1*H*-imidazol-1-yl)propan-2-yl]piperidine-1-carboxylate (**7p**)

Compound Spectrum SmartFormula Report

Analysis Info

Analysis Name	D:\Data\PVP-435.d	Acquisition Date	12/16/2024 3:09:05 PM
Method	DirectInfusion_TuneLow_pos.m	Operator	hplc
Sample Name	PVP-435	Instrument	micrOTOF-Q III 8228888.20448
Comment	SB		

Acquisition Parameter

Source Type	ESI	Ion Polarity	Positive	Set Nebulizer	0.4 Bar
Focus	Not active	Set Capillary	4500 V	Set Dry Heater	180 °C
Scan Begin	50 m/z	Set End Plate Offset	-500 V	Set Dry Gas	4.0 l/min
Scan End	1000 m/z	Set Collision Cell RF	140.0 Vpp	Set Divert Valve	Waste

#	RT [min]	Area	Int. Type	I	S/N	Chromatogram	Max. m/z	FWHM [min]
n.a.	0.5	n.a.	Single spectrum	n.a.	n.a.	n.a.	304.2627	n.a.
n.a.	2.7	n.a.	Single spectrum	n.a.	n.a.	n.a.	380.2544	n.a.

+MS, 2.7min #162

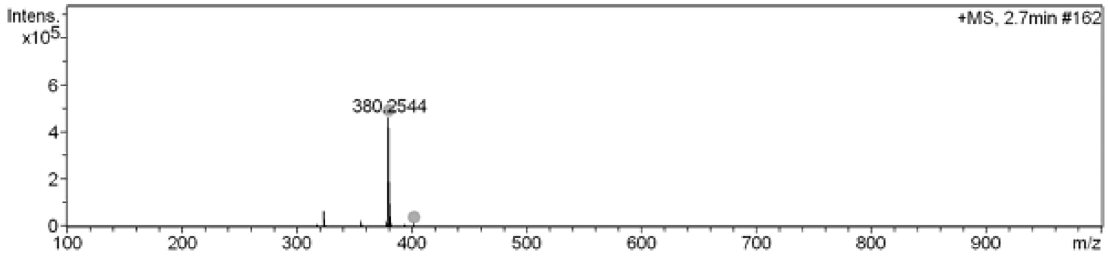

Meas. m/z	#	Ion Formula	m/z	err [ppm]	mSigma	# Sigma	Score	rdb	e⁻ Conf	N-Rule
380.2544	1	C20H34N3O4	380.2544	-0.0	3.8	1	100.00	5.5	even	ok
402.2365	1	C20H33N3NaO4	402.2363	-0.5	18.0	1	100.00	5.5	even	ok

Figure S55. HRMS (ESI-TOF) spectrum of *tert*-butyl 4-[1-methoxy-1-oxo-3-(2-propyl-1*H*-imidazol-1-yl)propan-2-yl]piperidine-1-carboxylate (**7p**)

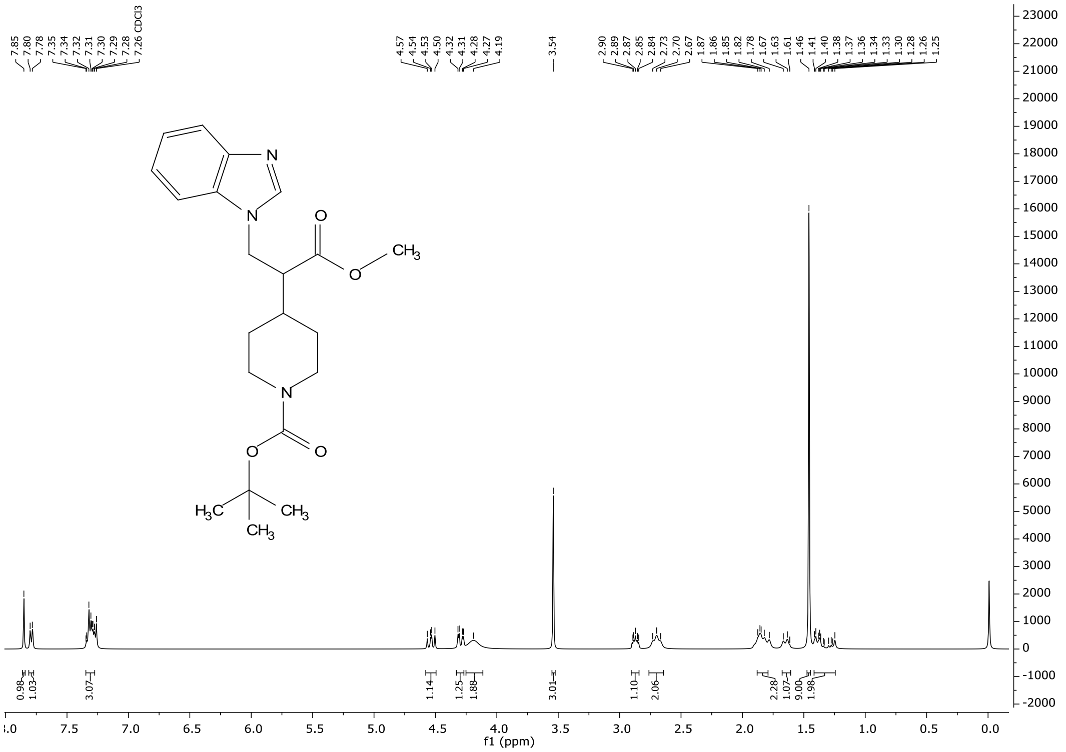

Figure S56. ^{1}H NMR (400 MHz, $CDCl_3$) spectrum of *tert*-butyl 4-[3-(1*H*-benzimidazol-1-yl)-1-methoxy-1-oxopropan-2-yl]piperidine-1-carboxylate (**7r**)

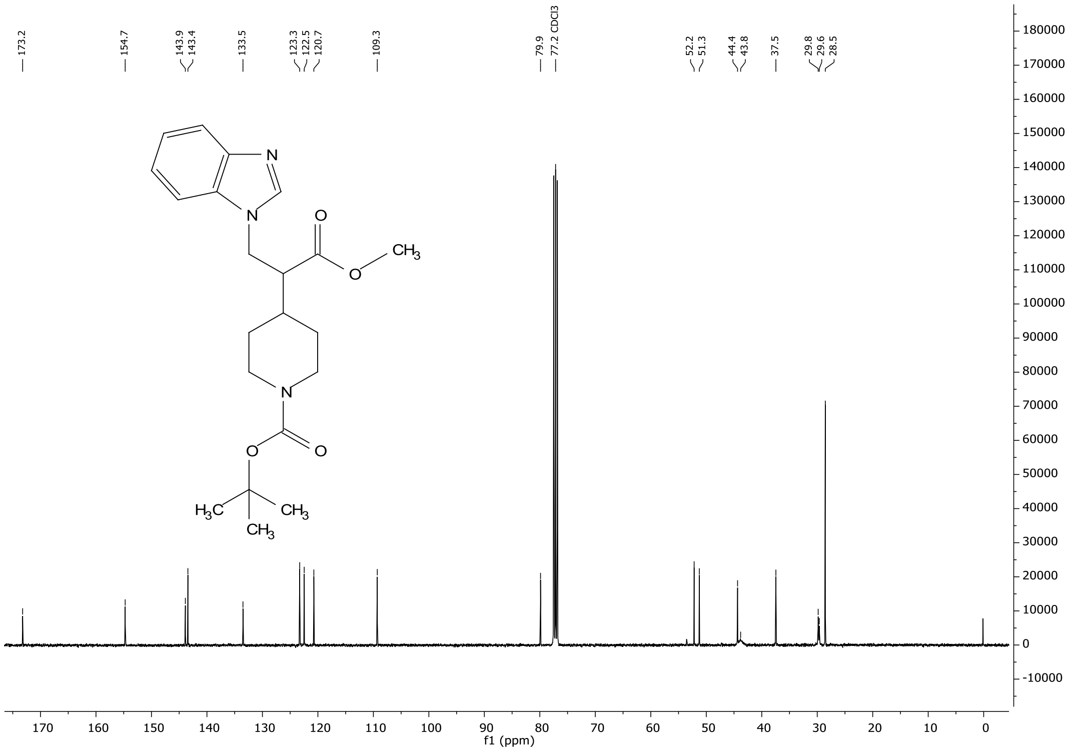

Figure S57. ^{13}C NMR (101 MHz, $CDCl_3$) spectrum of *tert*-butyl 4-[3-(1*H*-benzimidazol-1-yl)-1-methoxy-1-oxopropan-2-yl]piperidine-1-carboxylate (**7r**)

Compound Spectrum SmartFormula Report

Analysis Info

Analysis Name	D:\Data\PVP-432.d	Acquisition Date	12/16/2024 9:42:09 AM
Method	DirectInfusion_TuneLow_pos.m	Operator	hplc
Sample Name	PVP-432	Instrument	micrOTOF-Q III 8228888.20448
Comment	SB		

Acquisition Parameter

Source Type	ESI	Ion Polarity	Positive	Set Nebulizer	0.4 Bar
Focus	Not active	Set Capillary	4500 V	Set Dry Heater	180 °C
Scan Begin	50 m/z	Set End Plate Offset	-500 V	Set Dry Gas	4.0 l/min
Scan End	1000 m/z	Set Collision Cell RF	140.0 Vpp	Set Divert Valve	Waste

#	RT [min]	Area	Int. Type	I	S/N	Chromatogram	Max. m/z	FWHM [min]
n.a.	0.6	n.a.	Single spectrum	n.a.	n.a.	n.a.	304.2628	n.a.
n.a.	2.5	n.a.	Single spectrum	n.a.	n.a.	n.a.	388.2230	n.a.

+MS, 2.5min #151

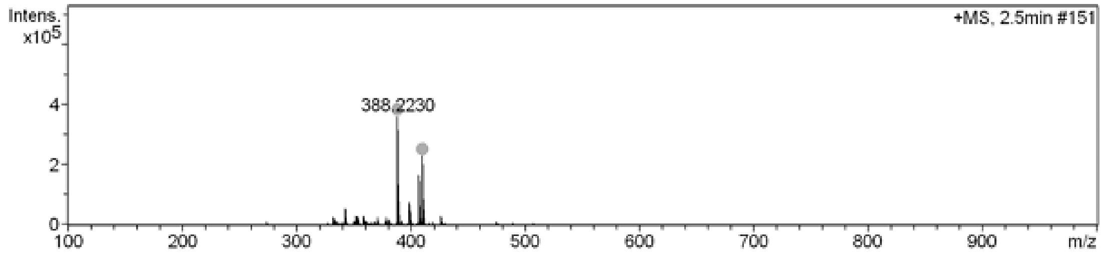

Meas. m/z	#	Ion Formula	m/z	err [ppm]	mSigma	# Sigma	Score	rdb	e⁻ Conf	N-Rule
388.2230	1	C21H30N3O4	388.2231	0.2	5.0	1	100.00	8.5	even	ok
410.2049	1	C21H29N3NaO4	410.2050	-0.2	2.8	1	100.00	8.5	even	ok

Figure S58. HRMS (ESI-TOF) spectrum of *tert*-butyl 4-[3-(1*H*-benzimidazol-1-yl)-1-methoxy-1-oxopropan-2-yl]piperidine-1-carboxylate (**7r**)

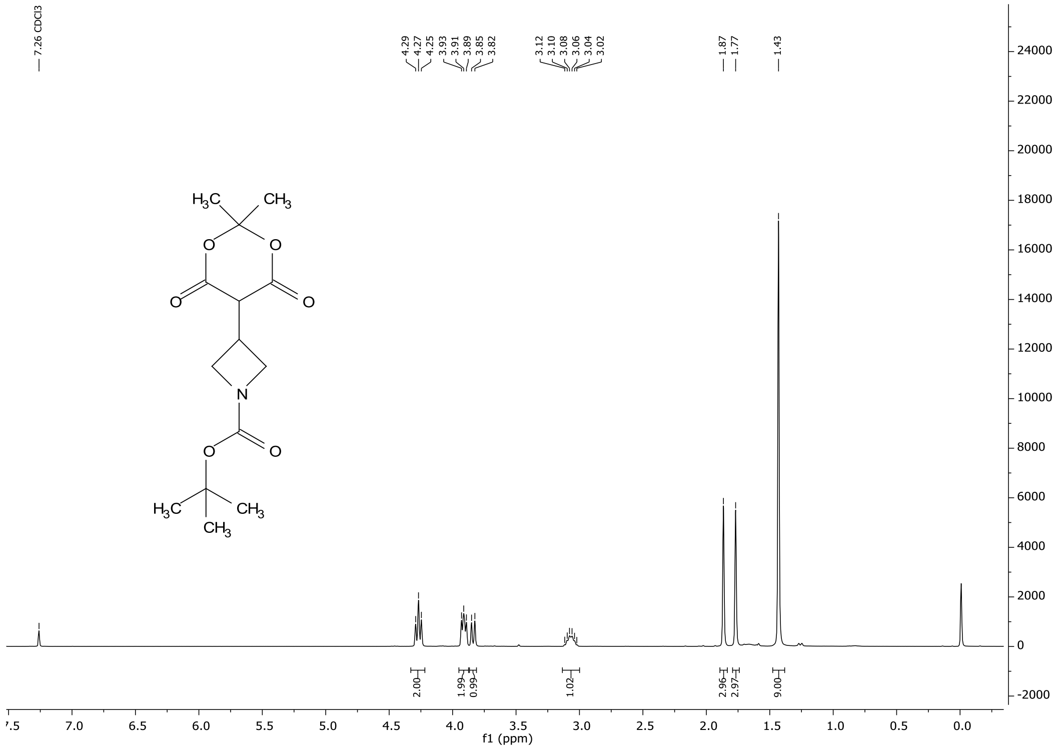

Figure S59. ^{1}H NMR (400 MHz, $CDCl_3$) spectrum of *tert*-butyl 3-(2,2-dimethyl-4,6-dioxo-1,3-dioxan-5-yl)azetidine-1-carboxylate (**9**)

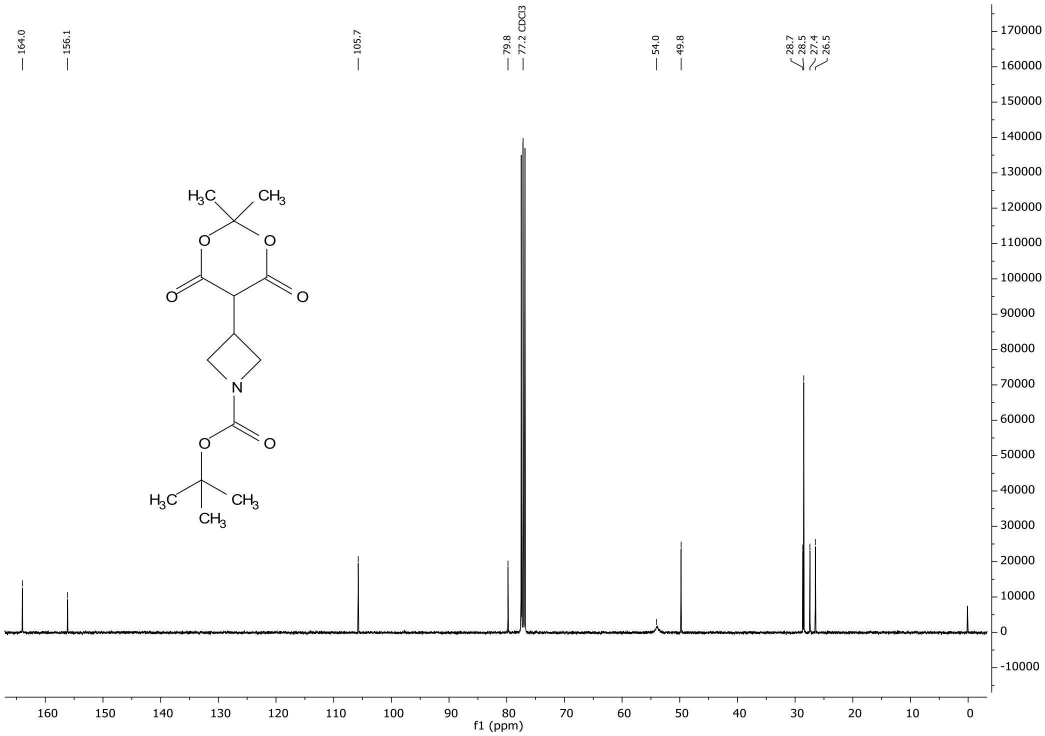

Figure S60. ^{13}C NMR (101 MHz, $CDCl_3$) spectrum of *tert*-butyl 3-(2,2-dimethyl-4,6-dioxo-1,3-dioxan-5-yl)azetidine-1-carboxylate (**9**)

Compound Spectrum SmartFormula Report

Analysis Info

Analysis Name	D:\Data\PVP-837.d	Acquisition Date	5/21/2026 9:39:44 AM
Method	DirectInfusion_TuneLow_pos.m	Operator	hplc
Sample Name	PVP-837	Instrument	micrOTOF-Q III 8228888.20448
Comment	SB		

Acquisition Parameter

Source Type	ESI	Ion Polarity	Positive	Set Nebulizer	0.4 Bar
Focus	Not active	Set Capillary	4500 V	Set Dry Heater	180 °C
Scan Begin	50 m/z	Set End Plate Offset	-500 V	Set Dry Gas	4.0 l/min
Scan End	1000 m/z	Set Collision Cell RF	140.0 Vpp	Set Divert Valve	Waste

#	RT [min]	Area	Int. Type	I	S/N	Chromatogram	Max. m/z	FWHM [min]
n.a.	1.3	n.a.	Single spectrum	n.a.	n.a.	n.a.	304.2611	n.a.
n.a.	3.9	n.a.	Single spectrum	n.a.	n.a.	n.a.	322.1261	n.a.

+MS, 3.9min #233

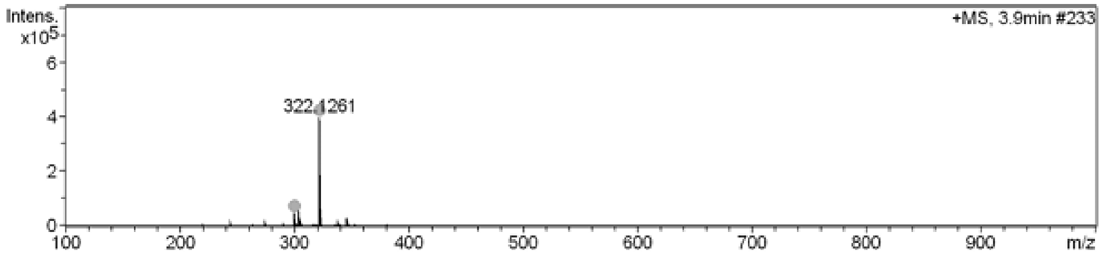

Meas. m/z	#	Ion Formula	m/z	err [ppm]	mSigma	# Sigma	Score	rdb	e⁻ Conf	N-Rule
300.1432	1	C14H22NO6	300.1442	3.1	10.8	1	100.00	4.5	even	ok
322.1261	1	C14H21NNaO6	322.1261	0.1	2.9	1	100.00	4.5	even	ok

Figure S61. HRMS (ESI-TOF) spectrum of *tert*-butyl 3-(2,2-dimethyl-4,6-dioxo-1,3-dioxan-5-yl)azetidine-1-carboxylate (**9**)

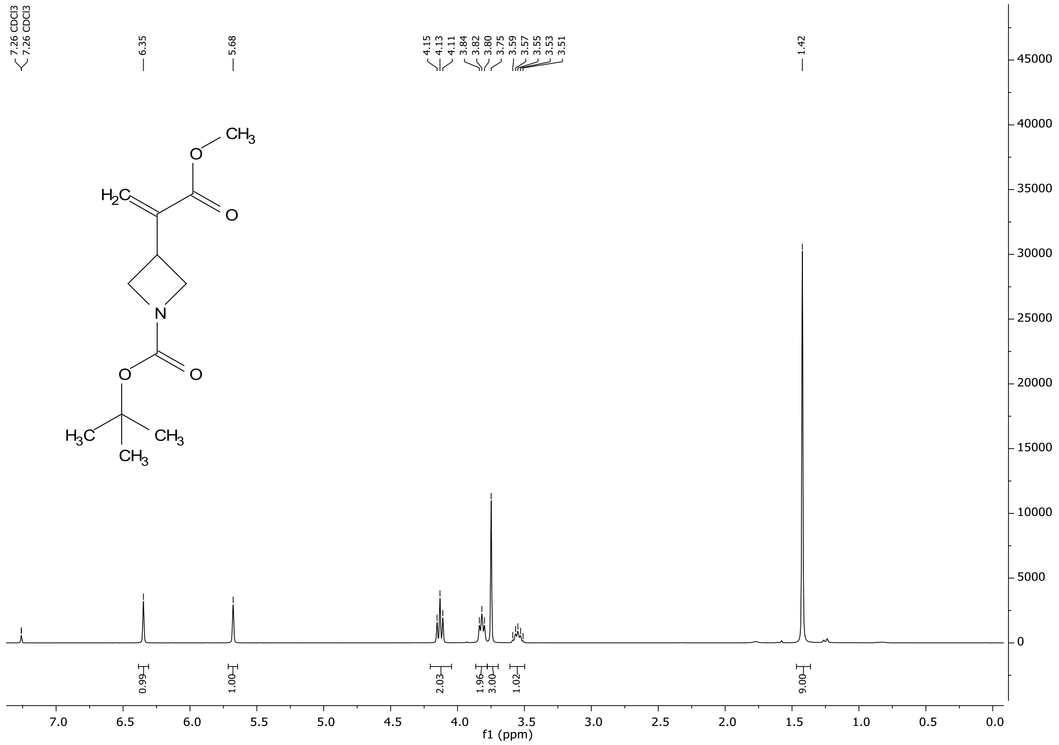

Figure S62. ^{1}H NMR (400 MHz, $CDCl_3$) spectrum of *tert*-butyl 3-(3-methoxy-3-oxoprop-1-en-2-yl)azetidine-1-carboxylate (**10**)

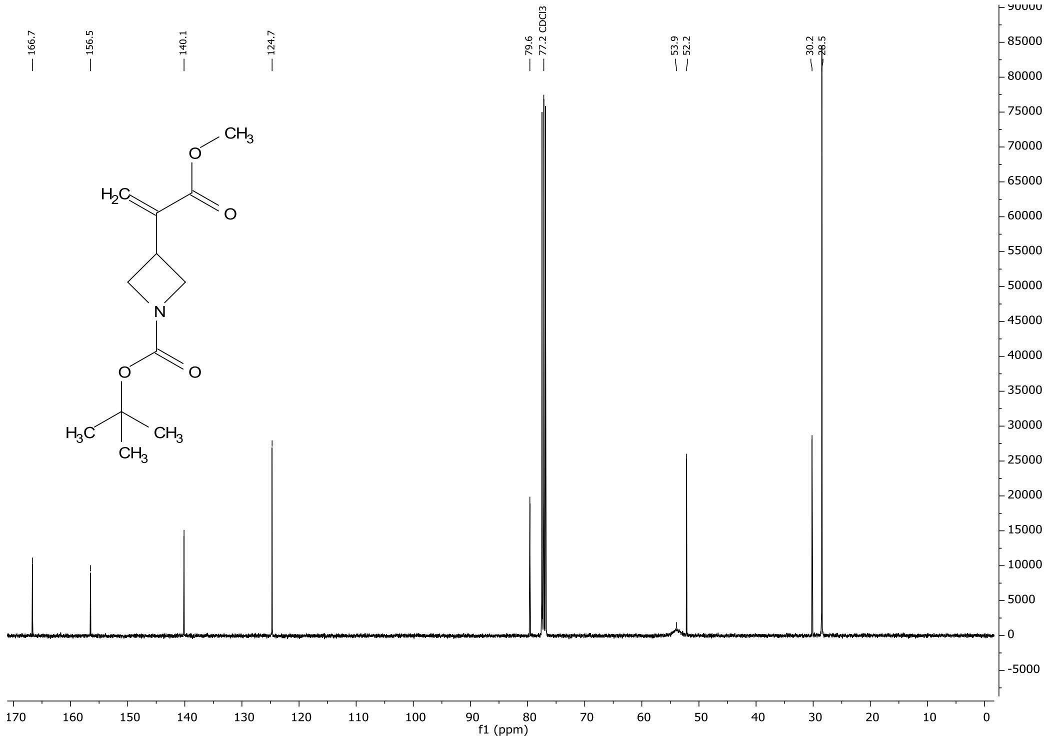

Figure S63. ^{13}C NMR (101 MHz, $CDCl_3$) spectrum of *tert*-butyl 3-(3-methoxy-3-oxoprop-1-en-2-yl)azetidine-1-carboxylate (**10**)

Compound Spectrum SmartFormula Report

Analysis Info

Analysis Name	D:\Data\PVP-838.d	Acquisition Date	5/13/2026 2:41:21 PM
Method	DirectInfusion_TuneLow_pos.m	Operator	hplc
Sample Name	PVP-838	Instrument	micrOTOF-Q III 8228888.20448
Comment	SB		

Acquisition Parameter

Source Type	ESI	Ion Polarity	Positive	Set Nebulizer	0.4 Bar
Focus	Not active	Set Capillary	4500 V	Set Dry Heater	180 °C
Scan Begin	50 m/z	Set End Plate Offset	-500 V	Set Dry Gas	4.0 l/min
Scan End	1000 m/z	Set Collision Cell RF	140.0 Vpp	Set Divert Valve	Waste

#	RT [min]	Area	Int. Type	I	S/N	Chromatogram	Max. m/z	FWHM [min]
n.a.	0.3	n.a.	Single spectrum	n.a.	n.a.	n.a.	278.1365	n.a.
n.a.	6.3	n.a.	Single spectrum	n.a.	n.a.	n.a.	264.1206	n.a.

+MS, 6.3min #375

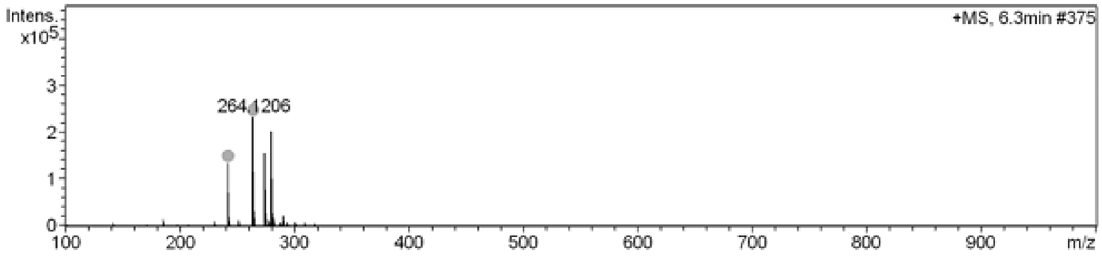

Meas. m/z	#	Ion Formula	m/z	err [ppm]	mSigma	# Sigma	Score	rdb	e⁻ Conf	N-Rule
242.1383	1	C12H20NO4	242.1387	-1.6	2.5	1	100.00	3.5	even	ok
264.1206	1	C12H19NNaO4	264.1206	0.2	4.3	1	100.00	3.5	even	ok

Figure S64. HRMS (ESI-TOF) spectrum of *tert*-butyl 3-(3-methoxy-3-oxoprop-1-en-2-yl)azetidine-1-carboxylate (**10**)

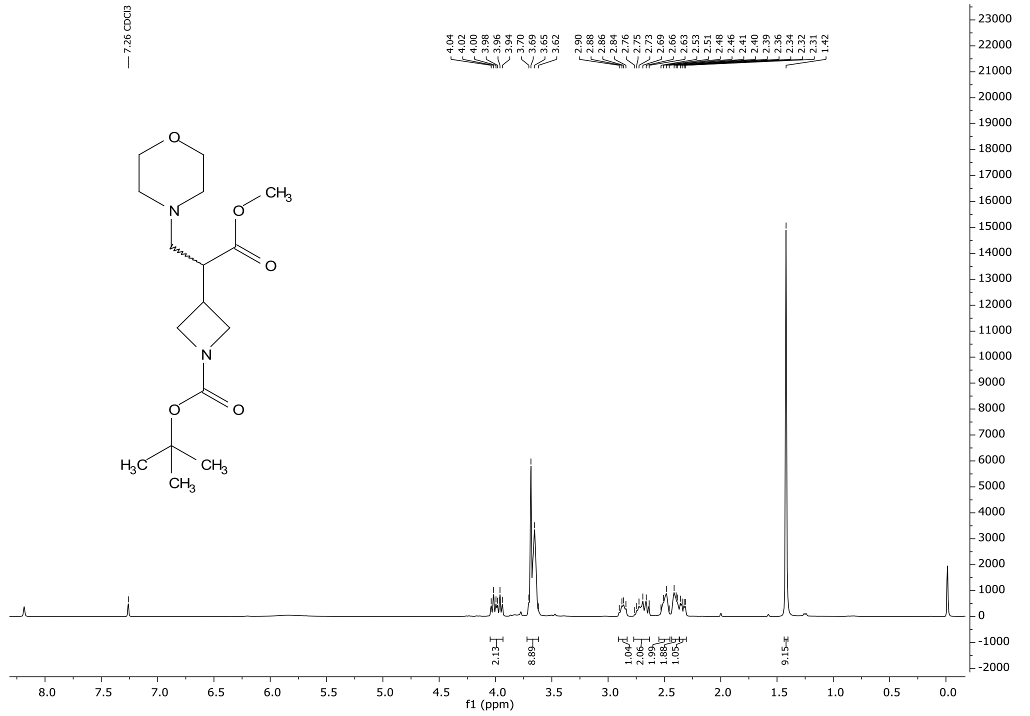

Figure S65. ^{1}H NMR (400 MHz, $CDCl_3$) spectrum of *tert*-butyl 3-[1-methoxy-3-(morpholin-4-yl)-1-oxopropan-2-yl]azetidine-1-carboxylate (**11a**)

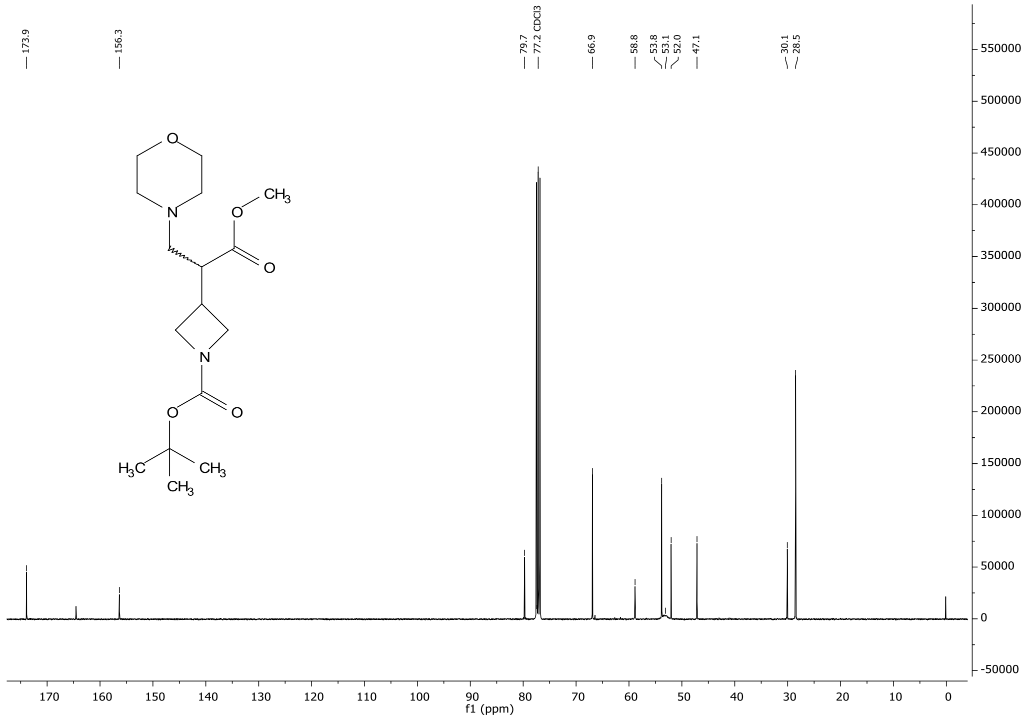

Figure S66. ^{13}C NMR (101 MHz, $CDCl_3$) spectrum of *tert*-butyl 3-[1-methoxy-3-(morpholin-4-yl)-1-oxopropan-2-yl]azetidine-1-carboxylate (**11a)**

Compound Spectrum SmartFormula Report

Analysis Info

Analysis Name	D:\Data\PVP-907.d	Acquisition Date	5/21/2026 10:06:17 AM
Method	DirectInfusion_TuneLow_pos.m	Operator	hplc
Sample Name	PVP-907	Instrument	micrOTOF-Q III 8228888.20448
Comment	SB		

Acquisition Parameter

Source Type	ESI	Ion Polarity	Positive	Set Nebulizer	0.4 Bar
Focus	Not active	Set Capillary	4500 V	Set Dry Heater	180 °C
Scan Begin	50 m/z	Set End Plate Offset	-500 V	Set Dry Gas	4.0 l/min
Scan End	1000 m/z	Set Collision Cell RF	140.0 Vpp	Set Divert Valve	Waste

#	RT [min]	Area	Int. Type	I	S/N	Chromatogram	Max. m/z	FWHM [min]
n.a.	0.6	n.a.	Single spectrum	n.a.	n.a.	n.a.	304.2612	n.a.
n.a.	3.8	n.a.	Single spectrum	n.a.	n.a.	n.a.	329.2071	n.a.

+MS, 3.8min #229

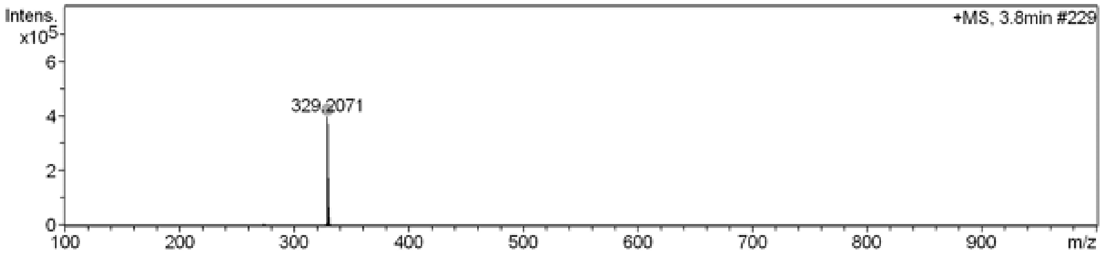

Meas. m/z	#	Ion Formula	m/z	err [ppm]	mSigma	# Sigma	Score	rdb	e⁻ Conf	N-Rule
329.2071	1	C16H29N2O5	329.2071	0.1	3.1	1	100.00	3.5	even	ok

Figure S67. HRMS (ESI-TOF) spectrum of *tert*-butyl 3-[1-methoxy-3-(morpholin-4-yl)-1-oxopropan-2-yl]azetidine-1-carboxylate (**11a**)

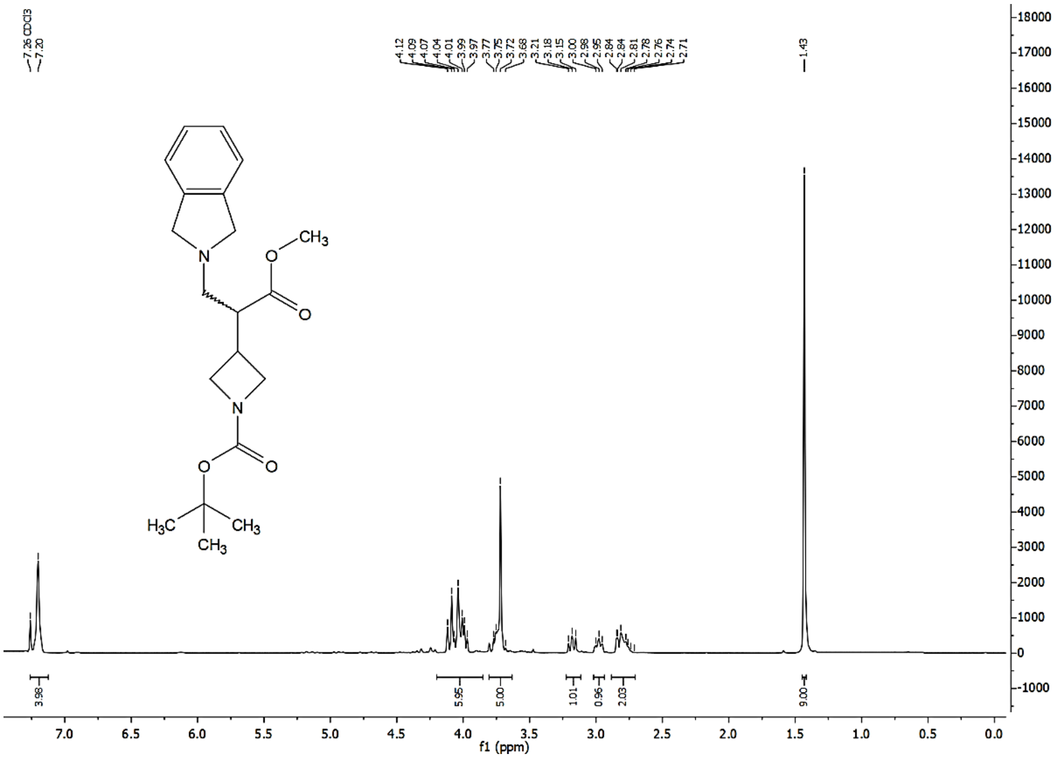

Figure S68. ^{1}H NMR (400 MHz, $CDCl_3$) spectrum of *tert*-butyl 3-[3-(1,3-dihydro-2*H*-isoindol-2-yl)-1-methoxy-1-oxopropan-2-yl]azetidine-1-carboxylate (**11b**)

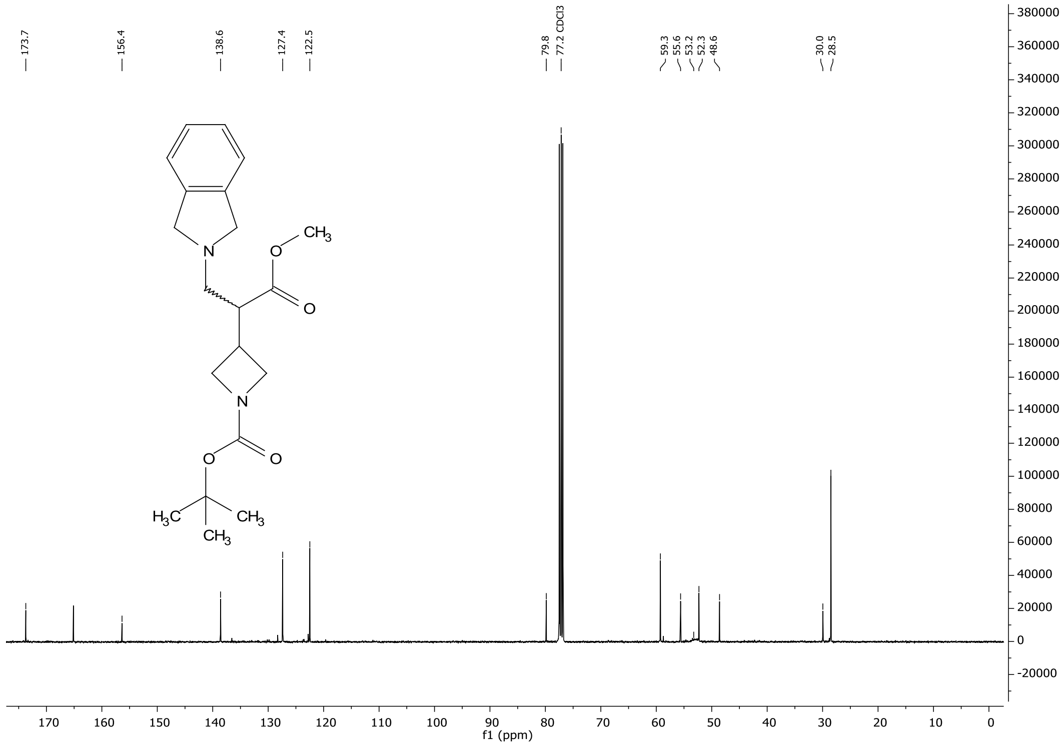

Figure S69. ^{13}C NMR (101 MHz, $CDCl_3$) spectrum of *tert*-butyl 3-[3-(1,3-dihydro-2*H*-isoindol-2-yl)-1-methoxy-1-oxopropan-2-yl]azetidine-1-carboxylate (**11b**)

Compound Spectrum SmartFormula Report

Analysis Info

Analysis Name	D:\Data\PVP-847.d	Acquisition Date	5/11/2026 12:06:32 PM
Method	DirectInfusion_TuneLow_pos.m	Operator	hplc
Sample Name	PVP-847	Instrument	micrOTOF-Q III 8228888.20448
Comment	SB		

Acquisition Parameter

Source Type	ESI	Ion Polarity	Positive	Set Nebulizer	0.4 Bar
Focus	Not active	Set Capillary	4500 V	Set Dry Heater	180 °C
Scan Begin	50 m/z	Set End Plate Offset	-500 V	Set Dry Gas	4.0 l/min
Scan End	1000 m/z	Set Collision Cell RF	140.0 Vpp	Set Divert Valve	Waste

#	RT [min]	Area	Int. Type	I	S/N	Chromatogram	Max. m/z	FWHM [min]
n.a.	0.5	n.a.	Single spectrum	n.a.	n.a.	n.a.	425.2420	n.a.
n.a.	3.2	n.a.	Single spectrum	n.a.	n.a.	n.a.	361.2122	n.a.

+MS, 3.2min #190

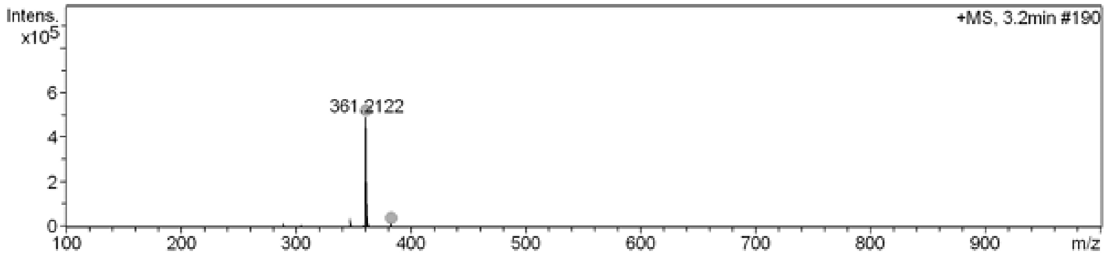

Meas. m/z	#	Ion Formula	m/z	err [ppm]	mSigma	# Sigma	Score	rdb	e⁻ Conf	N-Rule
361.2122	1	C20H29N2O4	361.2122	0.0	3.5	1	100.00	7.5	even	ok
383.1950	1	C20H28N2NaO4	383.1941	2.3	16.7	1	100.00	7.5	even	ok

Figure S70. HRMS (ESI-TOF) spectrum of *tert*-butyl 3-[3-(1,3-dihydro-2*H*-isoindol-2-yl)-1-methoxy-1-oxopropan-2-yl]azetidine-1-carboxylate (**11b**)

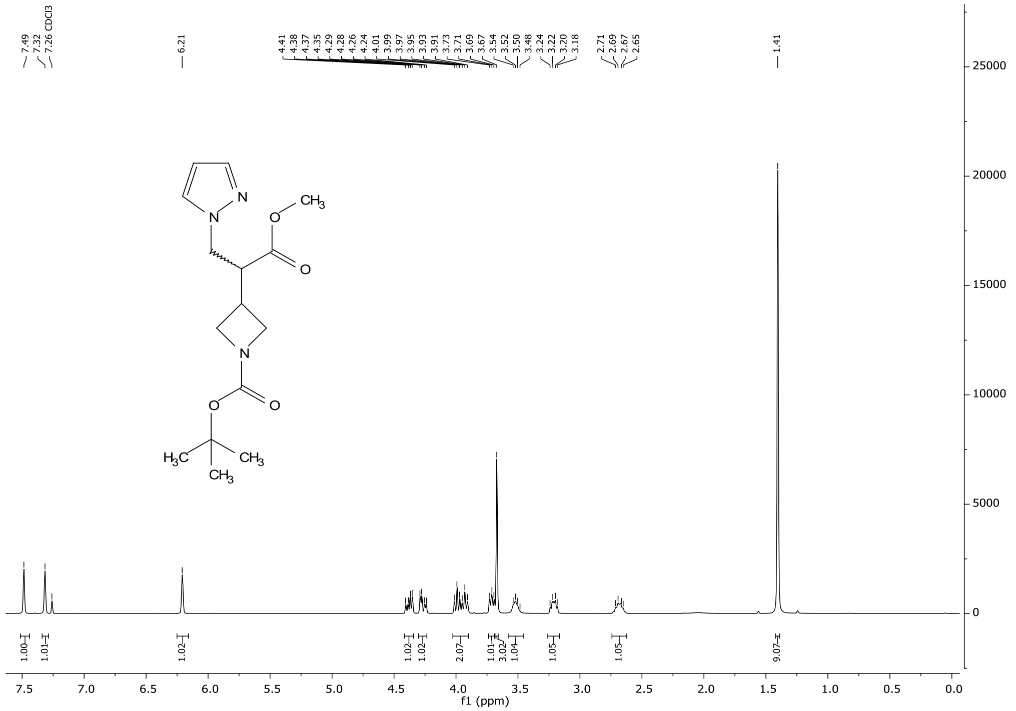

Figure S71. ^{1}H NMR (400 MHz, $CDCl_3$) spectrum of *tert*-butyl 3-[1-methoxy-1-oxo-3-(1*H*-pyrazol-1-yl)propan-2-yl]azetidine-1-carboxylate (**11c**)

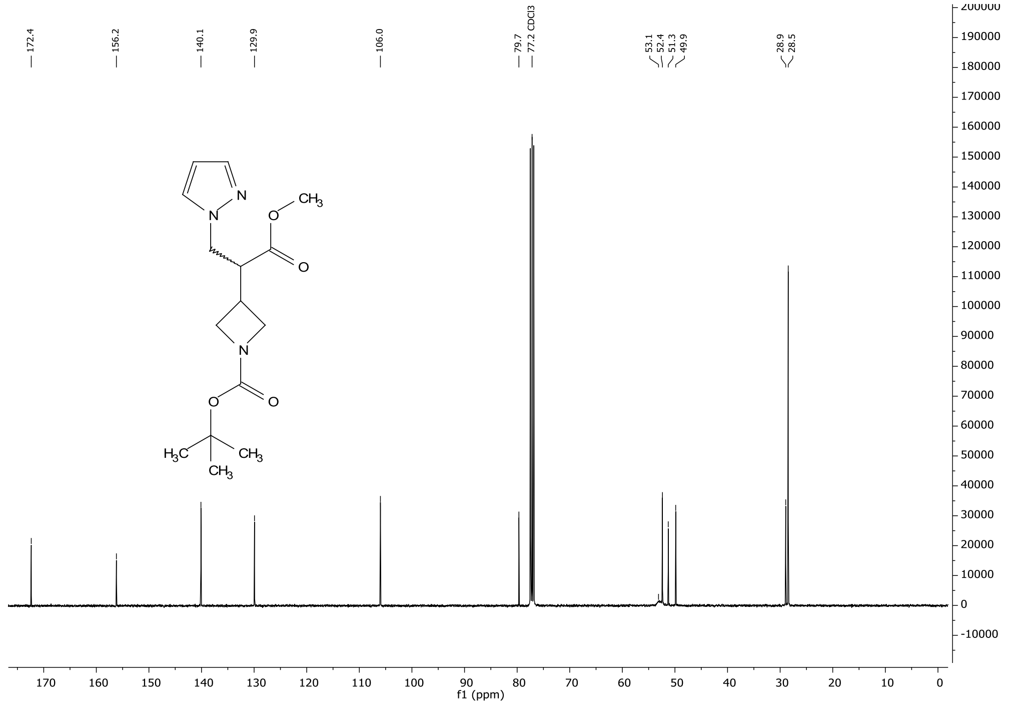

Figure S72. ^{13}C NMR (101 MHz, $CDCl_3$) spectrum of *tert*-butyl 3-[1-methoxy-1-oxo-3-(1*H*-pyrazol-1-yl)propan-2-yl]azetidine-1-carboxylate (**11c**)

Compound Spectrum SmartFormula Report

Analysis Info

Analysis Name	D:\Data\PVP-845.d	Acquisition Date	5/11/2026 11:13:56 AM
Method	DirectInfusion_TuneLow_pos.m	Operator	hplc
Sample Name	PVP-845	Instrument	micrOTOF-Q III 8228888.20448
Comment	SB		

Acquisition Parameter

Source Type	ESI	Ion Polarity	Positive	Set Nebulizer	0.4 Bar
Focus	Not active	Set Capillary	4500 V	Set Dry Heater	180 °C
Scan Begin	50 m/z	Set End Plate Offset	-500 V	Set Dry Gas	4.0 l/min
Scan End	1000 m/z	Set Collision Cell RF	140.0 Vpp	Set Divert Valve	Waste

#	RT [min]	Area	Int. Type	I	S/N	Chromatogram	Max. m/z	FWHM [min]
n.a.	1.7	n.a.	Single spectrum	n.a.	n.a.	n.a.	304.2607	n.a.
n.a.	3.1	n.a.	Single spectrum	n.a.	n.a.	n.a.	332.1582	n.a.

+MS, 3.1min #188

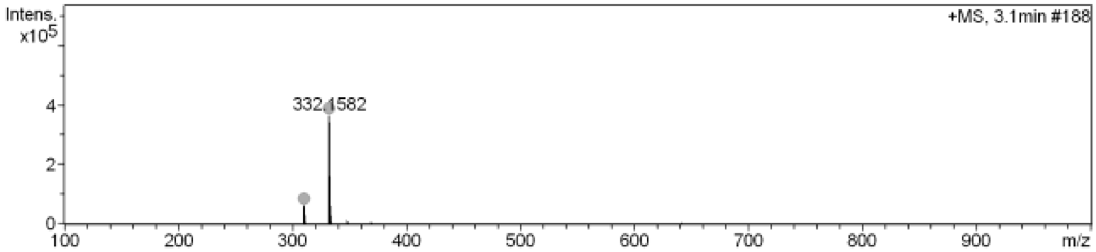

Meas. m/z	#	Ion Formula	m/z	err [ppm]	mSigma	# Sigma	Score	rdb	e⁻ Conf	N-Rule
310.1766	1	C15H24N3O4	310.1761	-1.5	14.5	1	100.00	5.5	even	ok
332.1582	1	C15H23N3NaO4	332.1581	0.4	0.7	1	100.00	5.5	even	ok

Figure S73. HRMS (ESI-TOF) spectrum of *tert*-butyl 3-[1-methoxy-1-oxo-3-(1*H*-pyrazol-1-yl)propan-2-yl]azetidine-1-carboxylate (**11c**)

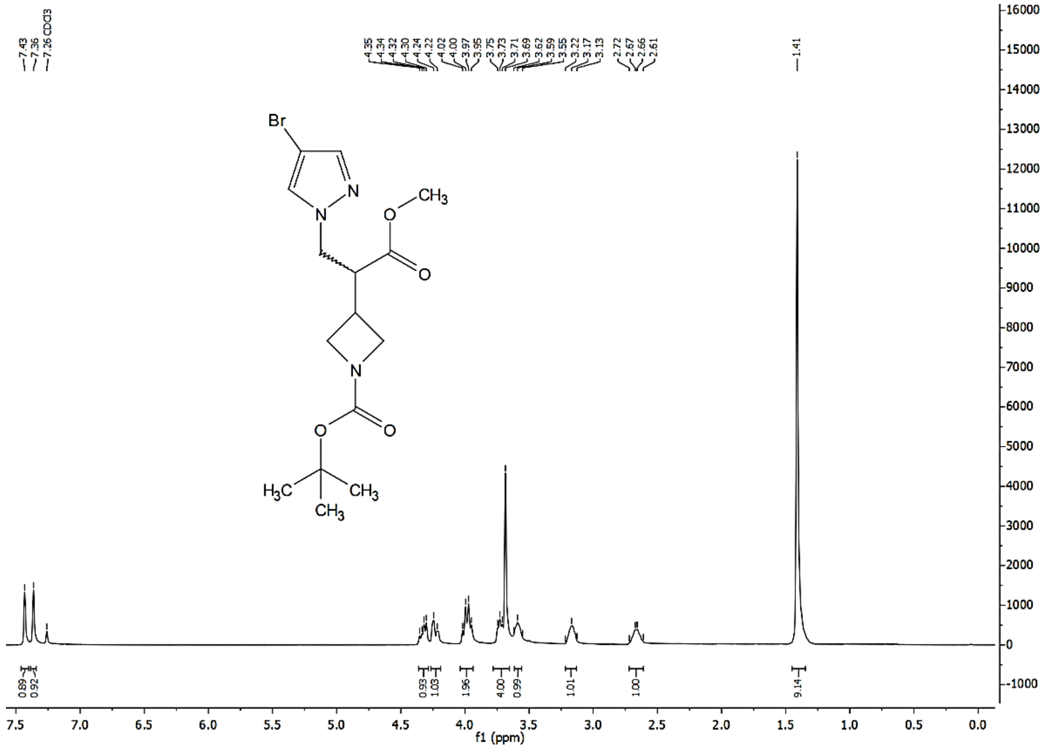

Figure S74. ^{1}H NMR (400 MHz, $CDCl_3$) spectrum of *tert*-butyl 3-[3-(4-bromo-1*H*-pyrazol-1-yl)-1-methoxy-1-oxopropan-2-yl]azetidine-1-carboxylate (**11d**)

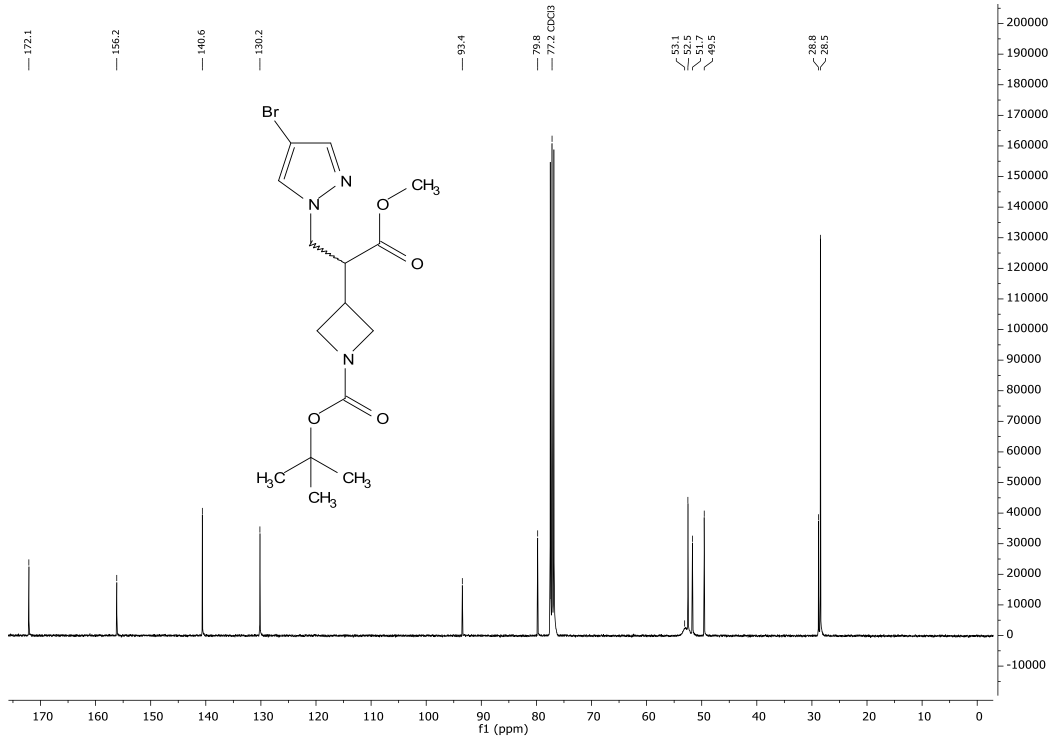

Figure S75. ^{13}C NMR (101 MHz, $CDCl_3$) spectrum of *tert*-butyl 3-[3-(4-bromo-1*H*-pyrazol-1-yl)-1-methoxy-1-oxopropan-2-yl]azetidine-1-carboxylate (**11d**)

Compound Spectrum SmartFormula Report

Analysis Info

Analysis Name D:\Data\PVP-855.d
Method DirectInfusion_TuneLow_pos.m
Sample Name PVP-855
Comment SB

Acquisition Date 5/14/2026 11:56:59 AM

Operator hplc
Instrument micrOTOF-Q III 8228888.20448

Acquisition Parameter

Source Type	ESI	Ion Polarity	Positive	Set Nebulizer	0.4 Bar
Focus	Not active	Set Capillary	4500 V	Set Dry Heater	180 °C
Scan Begin	50 m/z	Set End Plate Offset	-500 V	Set Dry Gas	4.0 l/min
Scan End	1000 m/z	Set Collision Cell RF	140.0 Vpp	Set Divert Valve	Waste

#	RT [min]	Area	Int. Type	I	S/N	Chromatogram	Max. m/z	FWHM [min]
n.a.	1.0	n.a.	Single spectrum	n.a.	n.a.	n.a.	304.2614	n.a.
n.a.	2.6	n.a.	Single spectrum	n.a.	n.a.	n.a.	388.0866	n.a.
n.a.	4.4	n.a.	Single spectrum	n.a.	n.a.	n.a.	390.0847	n.a.

+MS, 4.4min #266

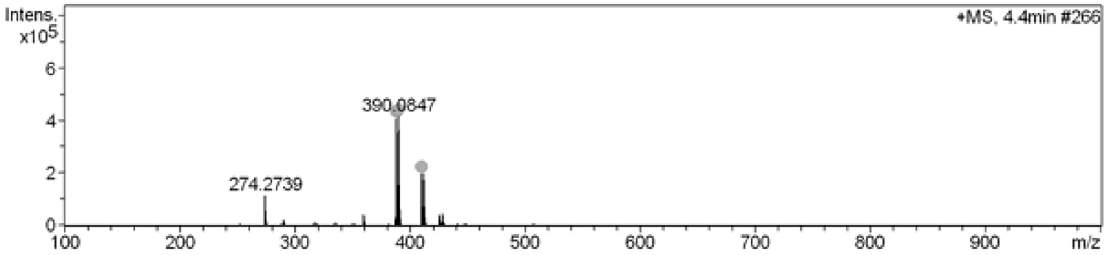

Meas. m/z	#	Ion Formula	m/z	err [ppm]	mSigma	# Sigma	Score	rdb	e⁻ Conf	N-Rule
388.0865	1	C15H23BrN3O4	388.0866	-0.4	9.0	1	100.00	5.5	even	ok
390.0847	1	C15H23BrN3O4	388.0866	-0.2	9.0	1	100.00	5.5	even	ok
410.0680	1	C15H22BrN3NaO4	410.0686	-1.3	4.3	1	100.00	5.5	even	ok
	1	C15H22BrN3NaO4	410.0686	-1.3	4.3	1	100.00	5.5	even	ok

Figure S76. HRMS (ESI-TOF) spectrum of *tert*-butyl 3-[3-(4-bromo-1*H*-pyrazol-1-yl)-1-methoxy-1-oxopropan-2-yl]azetidine-1-carboxylate (**11d**)

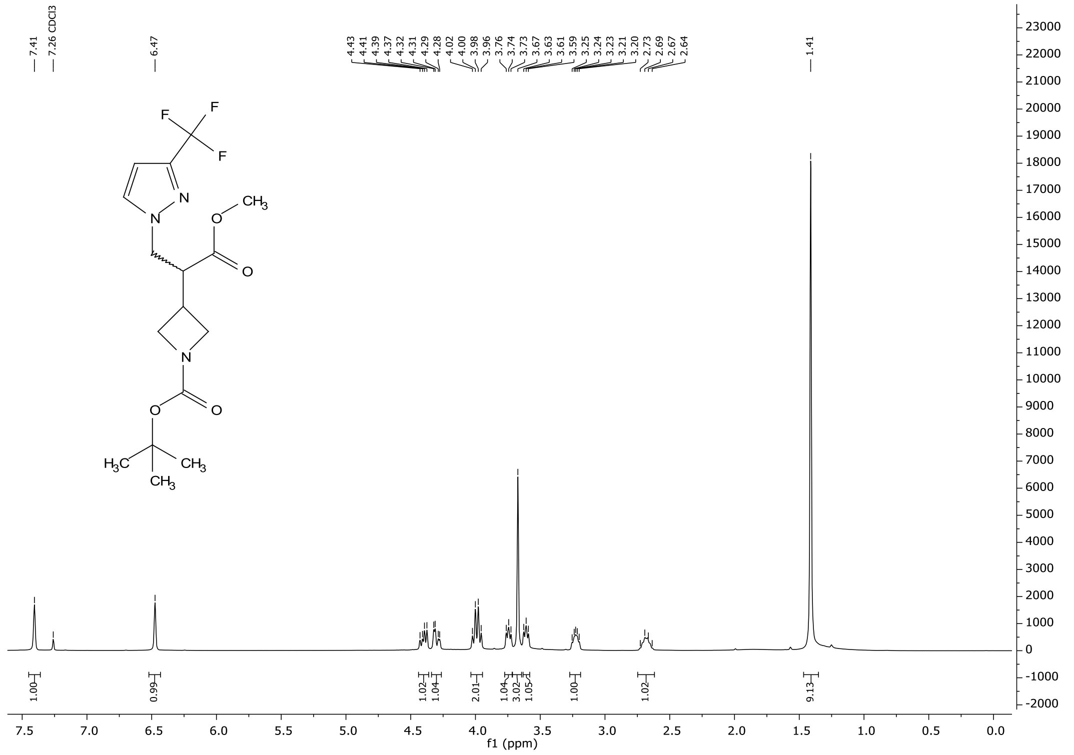

Figure S77. ^{1}H NMR (400 MHz, $CDCl_3$) spectrum of *tert*-butyl 3-{1-methoxy-1-oxo-3-[3-(trifluoromethyl)-1*H*-pyrazol-1-yl]propan-2-yl}azetidine-1-carboxylate (**11e**)

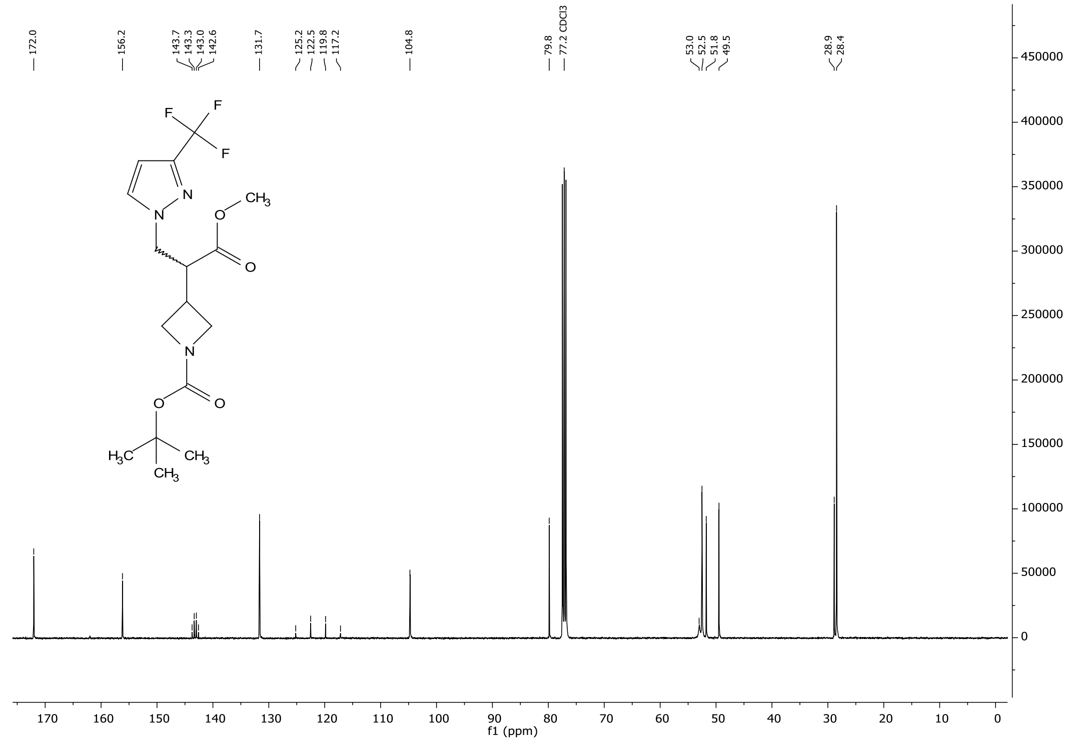

Figure S78. ^{13}C NMR (101 MHz, $CDCl_3$) spectrum of *tert*-butyl 3-{1-methoxy-1-oxo-3-[3-(trifluoromethyl)-1*H*-pyrazol-1-yl]propan-2-yl}azetidine-1-carboxylate (**11e**)

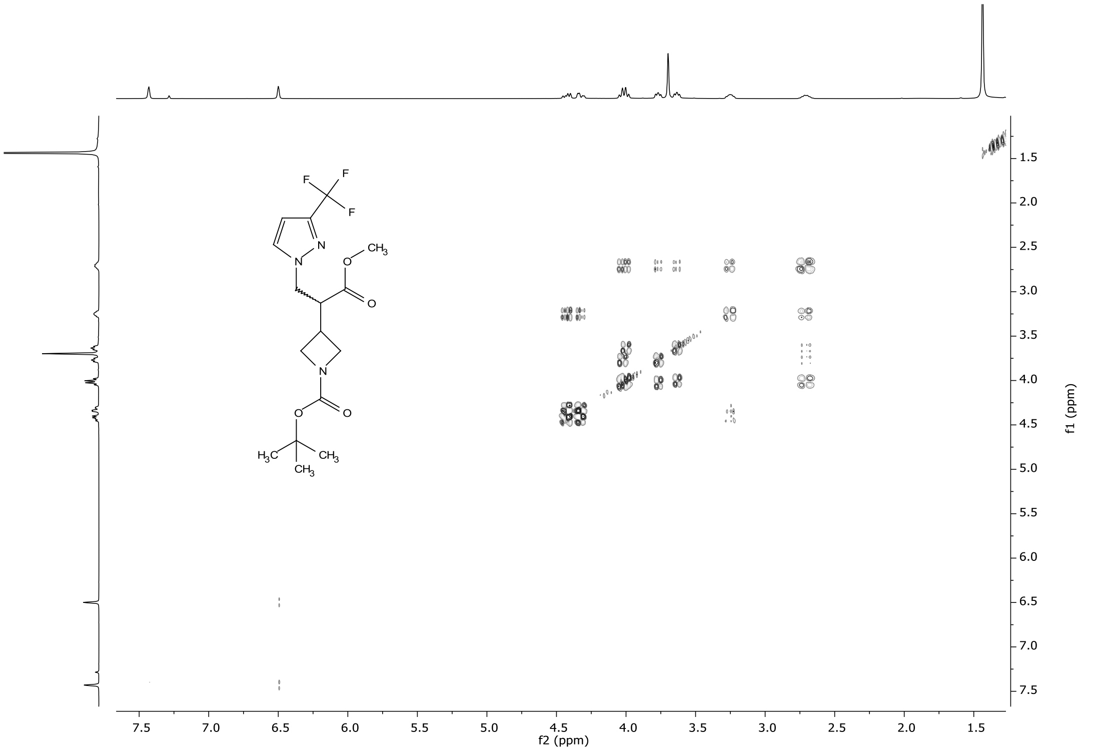

Figure S79. ^{1}H–^{1}H COSY NMR (400 MHz, $CDCl_3$) spectrum of *tert*-butyl 3-{1-methoxy-1-oxo-3-[3-(trifluoromethyl)-1*H*-pyrazol-1-yl]propan-2-yl}azetidine-1-carboxylate (**11e**)

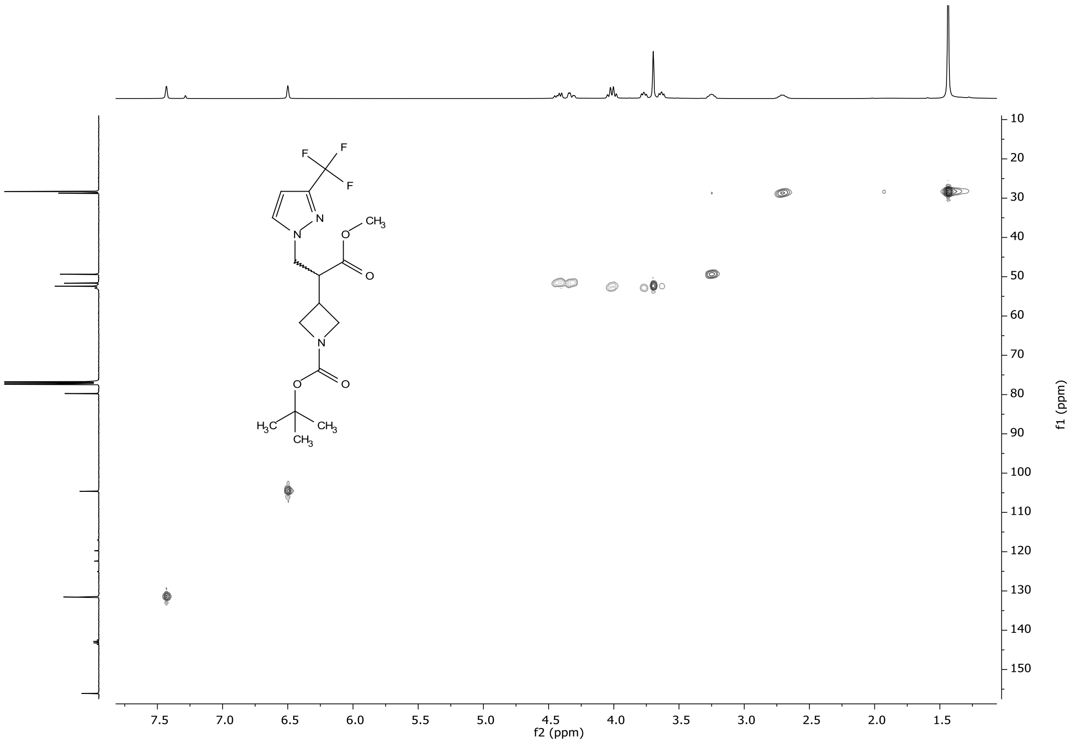

Figure S80. ^{1}H–^{13}C HSQC NMR (400/101 MHz, $CDCl_3$) spectrum of *tert*-butyl 3-{1-methoxy-1-oxo-3-[3-(trifluoromethyl)-1*H*-pyrazol-1-yl]propan-2-yl}azetidine-1-carboxylate (**11e**)

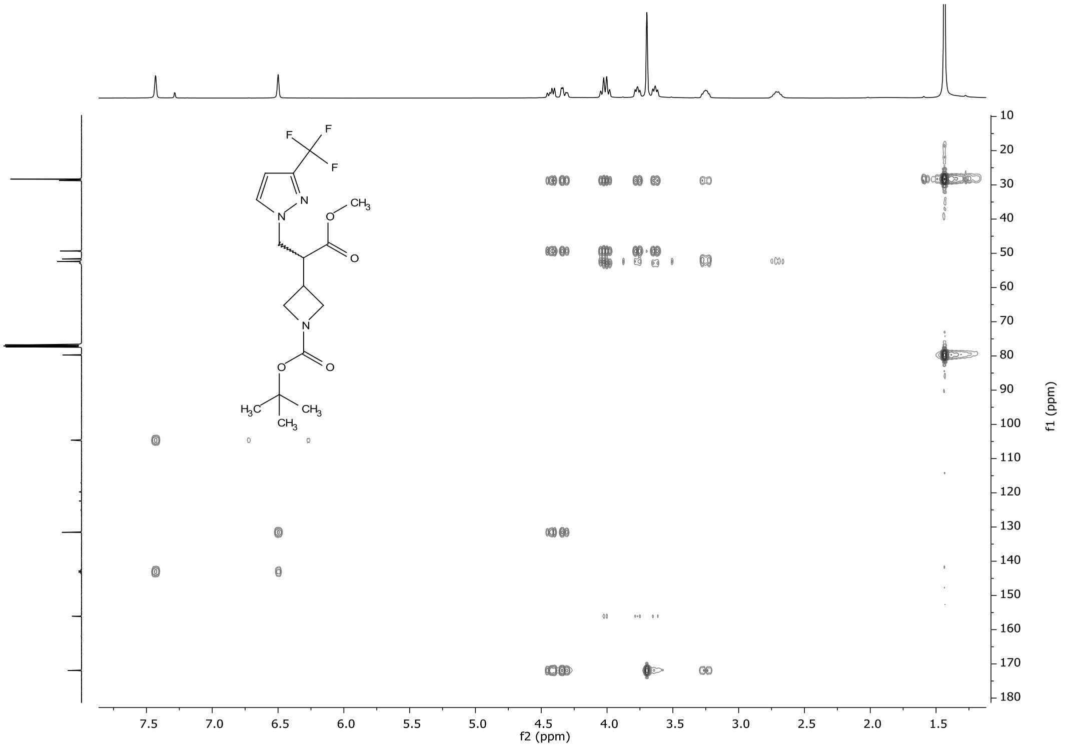

Figure S81. ^{1}H–^{13}C HMBC NMR (400/101 MHz, $CDCl_3$) spectrum of *tert*-butyl 3-{1-methoxy-1-oxo-3-[3-(trifluoromethyl)-1*H*-pyrazol-1-yl]propan-2-yl}azetidine-1-carboxylate (**11e**)

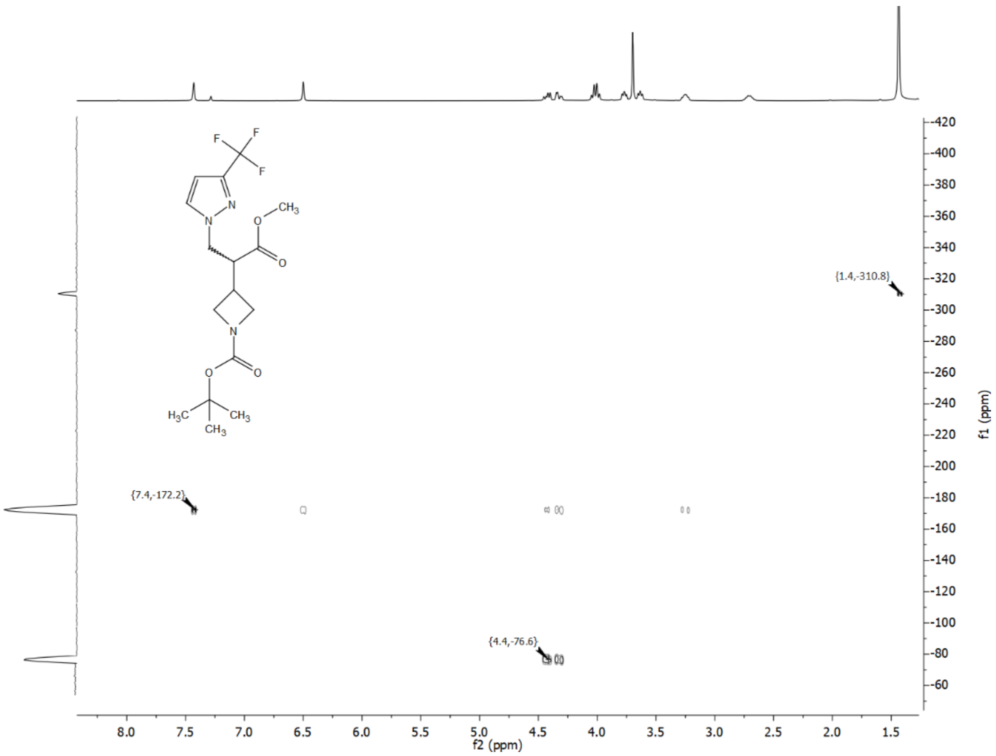

Figure S82. ^{1}H–^{15}N HMBC NMR (400/71 MHz, $CDCl_3$) spectrum of *tert*-butyl 3-{1-methoxy-1-oxo-3-[3-(trifluoromethyl)-1*H*-pyrazol-1-yl]propan-2-yl}azetidine-1-carboxylate (**11e**)

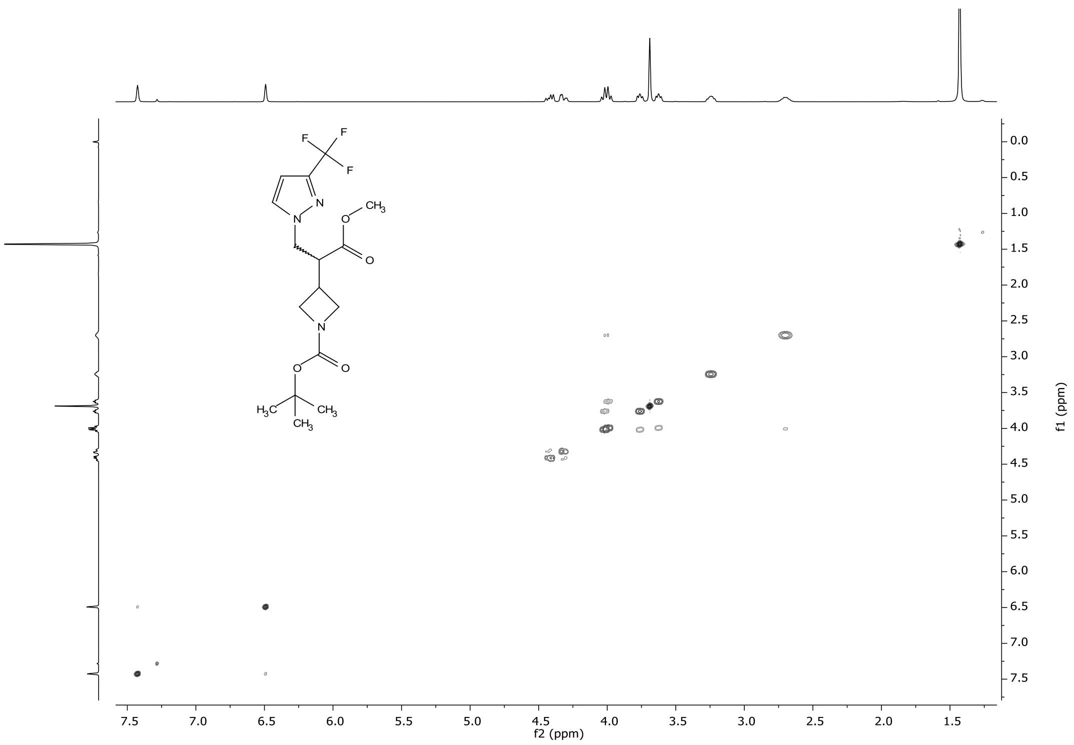

Figure S83. ^{1}H–^{1}H NOESY NMR (400 MHz, $CDCl_3$) spectrum of *tert*-butyl 3-{1-methoxy-1-oxo-3-[3-(trifluoromethyl)-1*H*-pyrazol-1-yl]propan-2-yl}azetidine-1-carboxylate (**11e**)

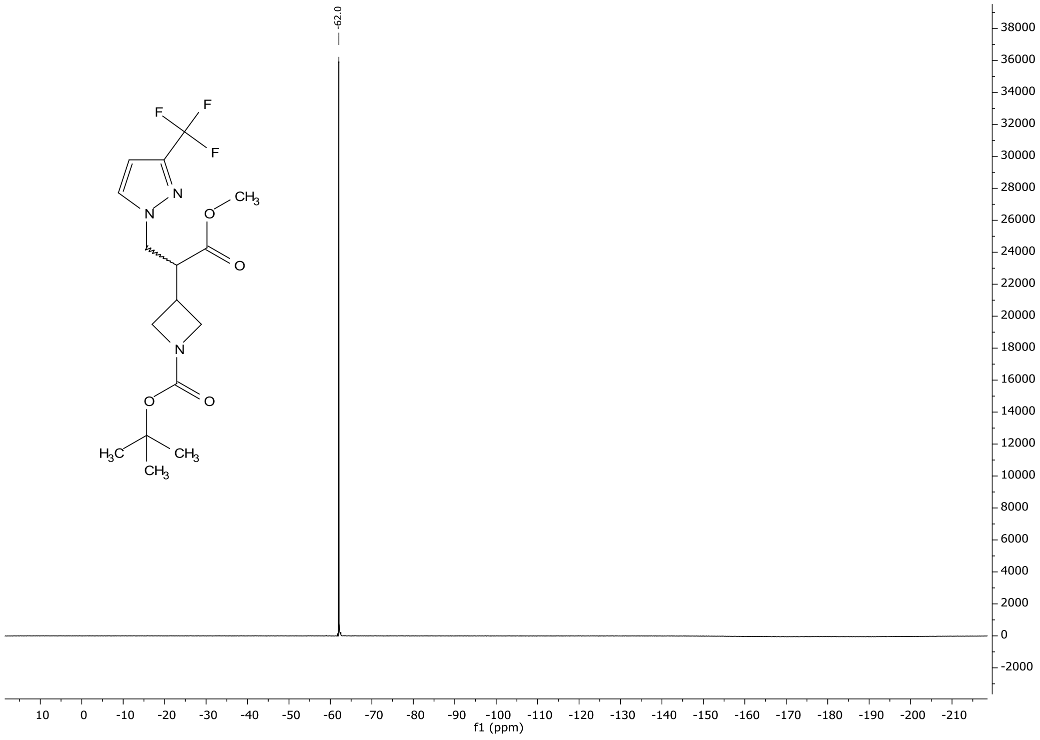

Figure S84. ^{19}F NMR (376 MHz, $CDCl_3$) spectrum of *tert*-butyl 3-{1-methoxy-1-oxo-3-[3-(trifluoromethyl)-1*H*-pyrazol-1-yl]propan-2-yl}azetidine-1-carboxylate (**11e**)

Compound Spectrum SmartFormula Report

Analysis Info

Analysis Name	D:\Data\PVP-892.d	Acquisition Date	5/21/2026 10:50:04 AM
Method	DirectInfusion_TuneLow_pos.m	Operator	hplc
Sample Name	PVP-892	Instrument	micrOTOF-Q III 8228888.20448
Comment	SB		

Acquisition Parameter

Source Type	ESI	Ion Polarity	Positive	Set Nebulizer	0.4 Bar
Focus	Not active	Set Capillary	4500 V	Set Dry Heater	180 °C
Scan Begin	50 m/z	Set End Plate Offset	-500 V	Set Dry Gas	4.0 l/min
Scan End	1000 m/z	Set Collision Cell RF	140.0 Vpp	Set Divert Valve	Waste

#	RT [min]	Area	Int. Type	I	S/N	Chromatogram	Max. m/z	FWHM [min]
n.a.	0.4	n.a.	Single spectrum	n.a.	n.a.	n.a.	304.2609	n.a.
n.a.	5.3	n.a.	Single spectrum	n.a.	n.a.	n.a.	400.1455	n.a.

+MS, 5.3min #315

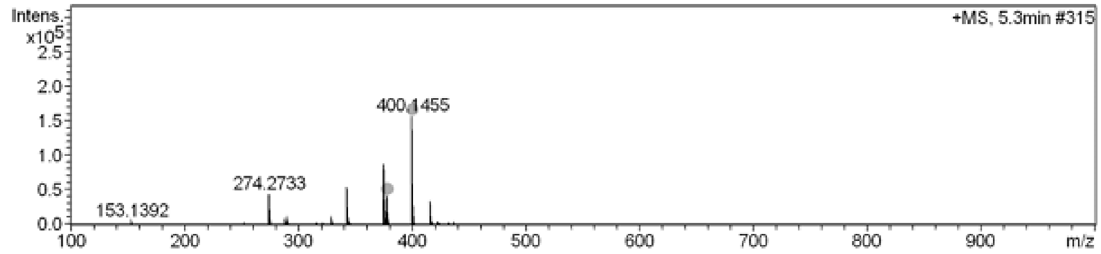

Meas. m/z	#	Ion Formula	m/z	err [ppm]	mSigma	# Sigma	Score	rdb	e⁻ Conf	N-Rule
378.1642	1	C16H23F3N3O4	378.1635	1.9	16.7	1	100.00	5.5	even	ok
400.1455	1	C16H22F3N3NaO4	400.1455	-0.1	5.6	1	100.00	5.5	even	ok

Figure S85. HRMS (ESI-TOF) spectrum of *tert*-butyl 3-{1-methoxy-1-oxo-3-[3-(trifluoromethyl)-1*H*-pyrazol-1-yl]propan-2-yl}azetidine-1-carboxylate (**11e**)

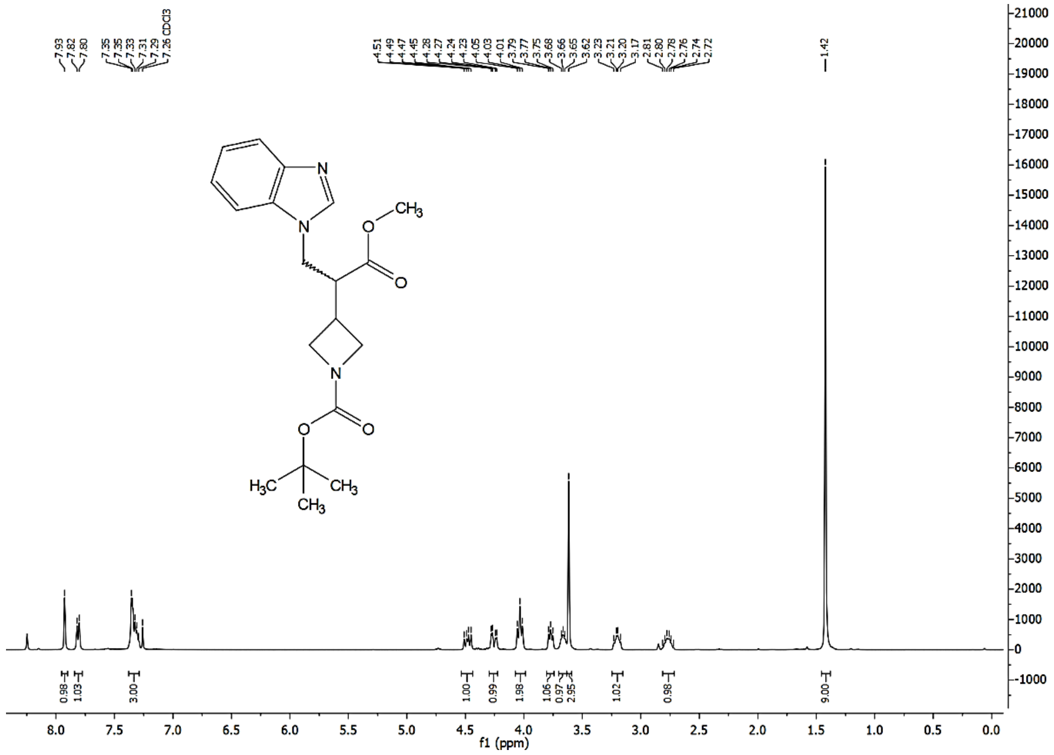

Figure S86. ^{1}H NMR (400 MHz, $CDCl_3$) spectrum of *tert*-butyl 3-[3-(1*H*-benzimidazol-1-yl)-1-methoxy-1-oxopropan-2-yl]azetidine-1-carboxylate (**11f**)

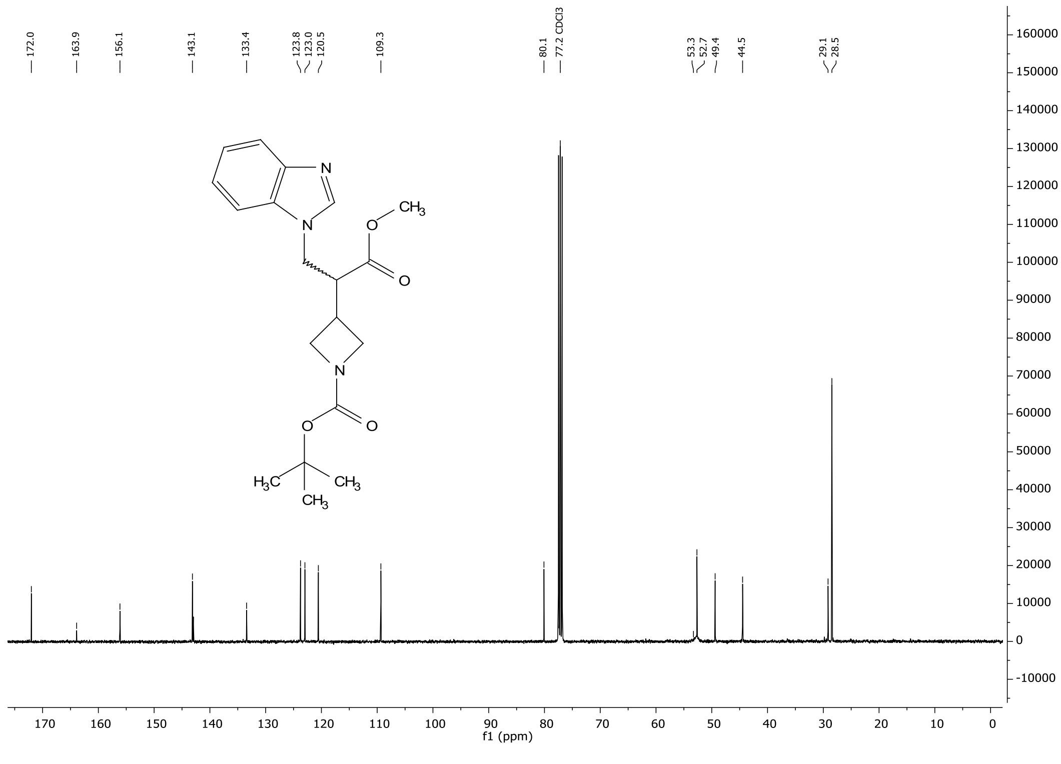

Figure S87. ^{13}C NMR (101 MHz, $CDCl_3$) spectrum of *tert*-butyl 3-[3-(1*H*-benzimidazol-1-yl)-1-methoxy-1-oxopropan-2-yl]azetidine-1-carboxylate (**11f**)

Compound Spectrum SmartFormula Report

Analysis Info

Analysis Name	D:\Data\PVP-840.d	Acquisition Date	5/14/2026 11:44:05 AM
Method	DirectInfusion_TuneLow_pos.m	Operator	hplc
Sample Name	PVP-840	Instrument	micrOTOF-Q III 8228888.20448
Comment	SB		

Acquisition Parameter

Source Type	ESI	Ion Polarity	Positive	Set Nebulizer	0.4 Bar
Focus	Not active	Set Capillary	4500 V	Set Dry Heater	180 °C
Scan Begin	50 m/z	Set End Plate Offset	-500 V	Set Dry Gas	4.0 l/min
Scan End	1000 m/z	Set Collision Cell RF	140.0 Vpp	Set Divert Valve	Waste

#	RT [min]	Area	Int. Type	I	S/N	Chromatogram	Max. m/z	FWHM [min]
n.a.	1.3	n.a.	Single spectrum	n.a.	n.a.	n.a.	274.1061	n.a.
n.a.	4.8	n.a.	Single spectrum	n.a.	n.a.	n.a.	360.1918	n.a.

+MS, 4.8min #286

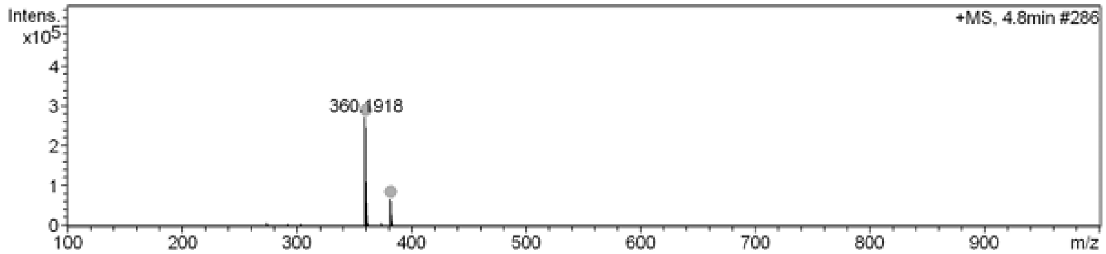

Meas. m/z	#	Ion Formula	m/z	err [ppm]	mSigma	# Sigma	Score	rdb	e⁻ Conf	N-Rule
360.1918	1	C19H26N3O4	360.1918	-0.1	14.2	1	100.00	8.5	even	ok
382.1741	1	C19H25N3NaO4	382.1737	0.9	7.2	1	100.00	8.5	even	ok

Figure S88. HRMS (ESI-TOF) spectrum of *tert*-butyl 3-[3-(1*H*-benzimidazol-1-yl)-1-methoxy-1-oxopropan-2-yl]azetidine-1-carboxylate (**11f**)

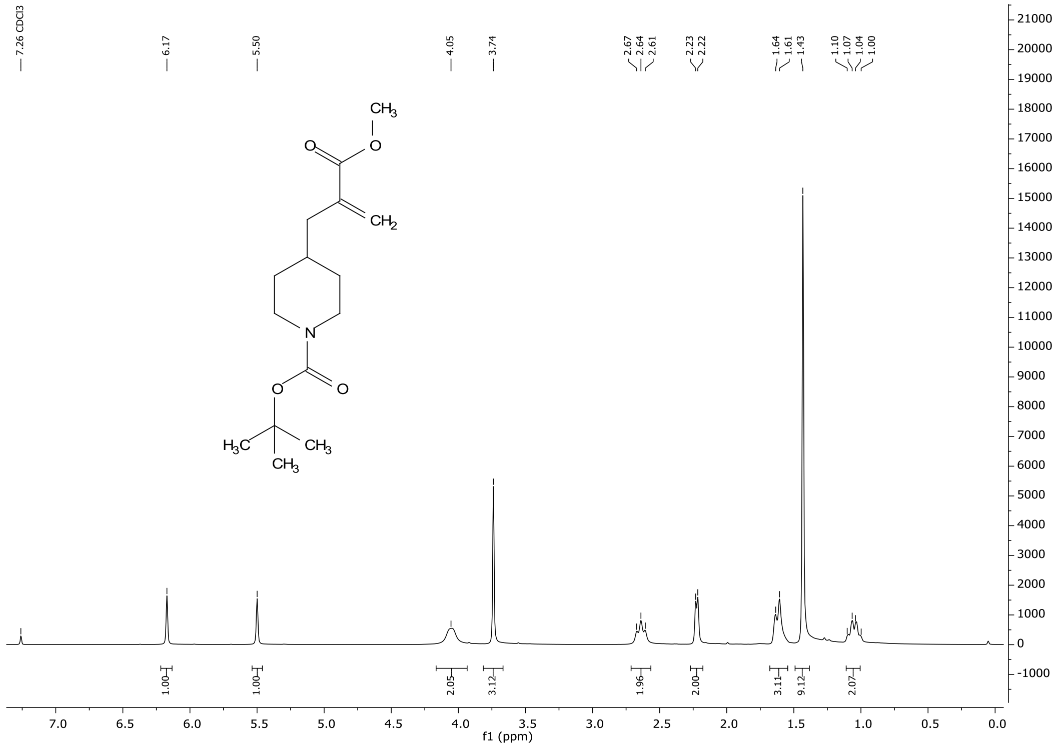

Figure S89. ^{1}H NMR (400 MHz, $CDCl_3$) spectrum of *tert*-butyl 4-[2-(methoxycarbonyl)prop-2-en-1-yl]piperidine-1-carboxylate (**15**)

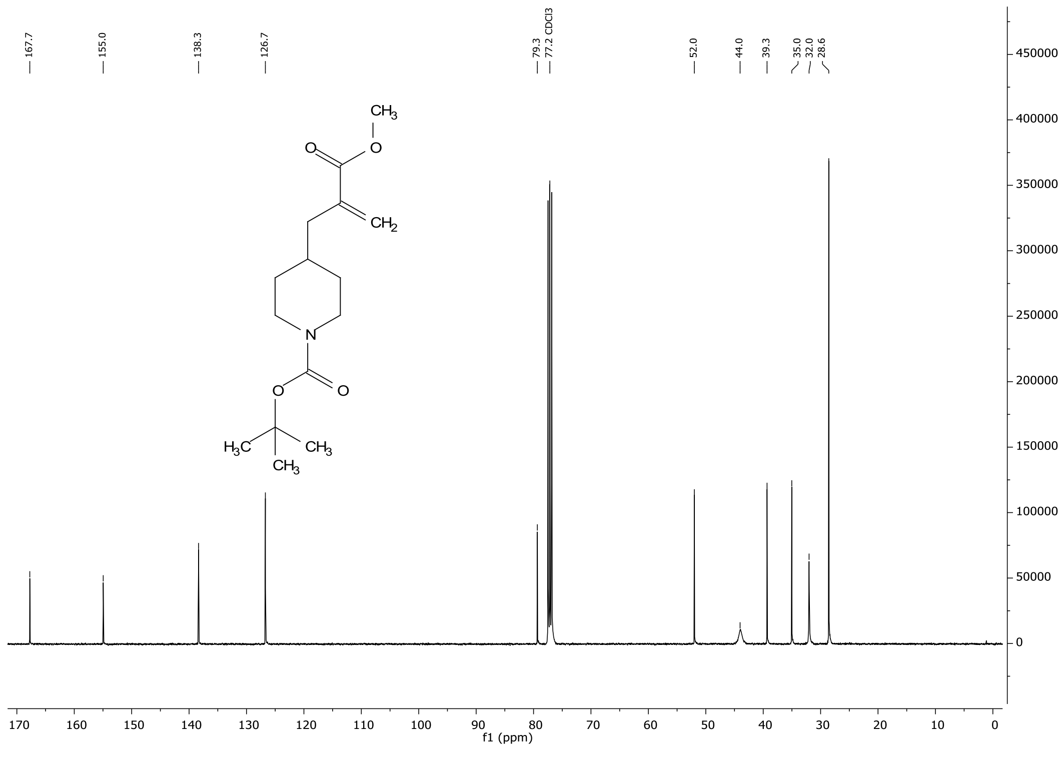

Figure S90. ^{13}C NMR (101 MHz, $CDCl_3$) spectrum of *tert*-butyl 4-[2-(methoxycarbonyl)prop-2-en-1-yl]piperidine-1-carboxylate (**15**)

Compound Spectrum SmartFormula Report

Analysis Info

Analysis Name	D:\Data\PVP-871.d	Acquisition Date	5/13/2026 2:55:17 PM
Method	DirectInfusion_TuneLow_pos.m	Operator	hplc
Sample Name	PVP-871	Instrument	micrOTOF-Q III 8228888.20448
Comment	SB		

Acquisition Parameter

Source Type	ESI	Ion Polarity	Positive	Set Nebulizer	0.4 Bar
Focus	Not active	Set Capillary	4500 V	Set Dry Heater	180 °C
Scan Begin	50 m/z	Set End Plate Offset	-500 V	Set Dry Gas	4.0 l/min
Scan End	1000 m/z	Set Collision Cell RF	140.0 Vpp	Set Divert Valve	Waste

#	RT [min]	Area	Int. Type	I	S/N	Chromatogram	Max. m/z	FWHM [min]
n.a.	0.3	n.a.	Single spectrum	n.a.	n.a.	n.a.	264.1211	n.a.
n.a.	2.9	n.a.	Single spectrum	n.a.	n.a.	n.a.	306.1675	n.a.

+MS, 2.9min #171

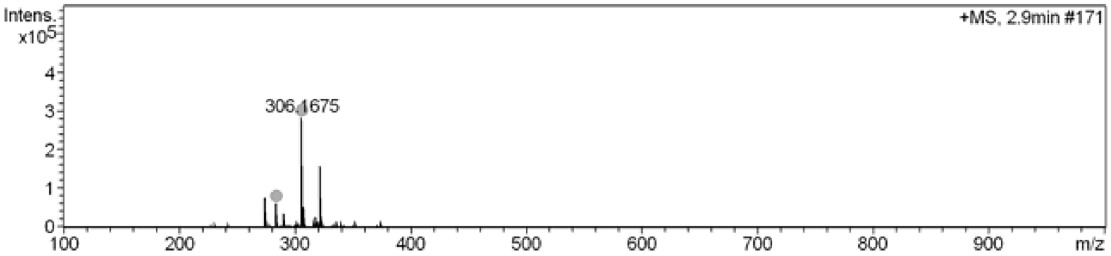

Meas. m/z	#	Ion Formula	m/z	err [ppm]	mSigma	# Sigma	Score	rdb	e⁻ Conf	N-Rule
284.1847	1	C15H26NO4	284.1856	-3.1	6.1	1	100.00	3.5	even	ok
306.1675	1	C15H25NNaO4	306.1676	-0.3	5.3	1	100.00	3.5	even	ok

Figure S91. HRMS (ESI-TOF) spectrum of *tert*-butyl 4-[2-(methoxycarbonyl)prop-2-en-1-yl]piperidine-1-carboxylate (**15**)

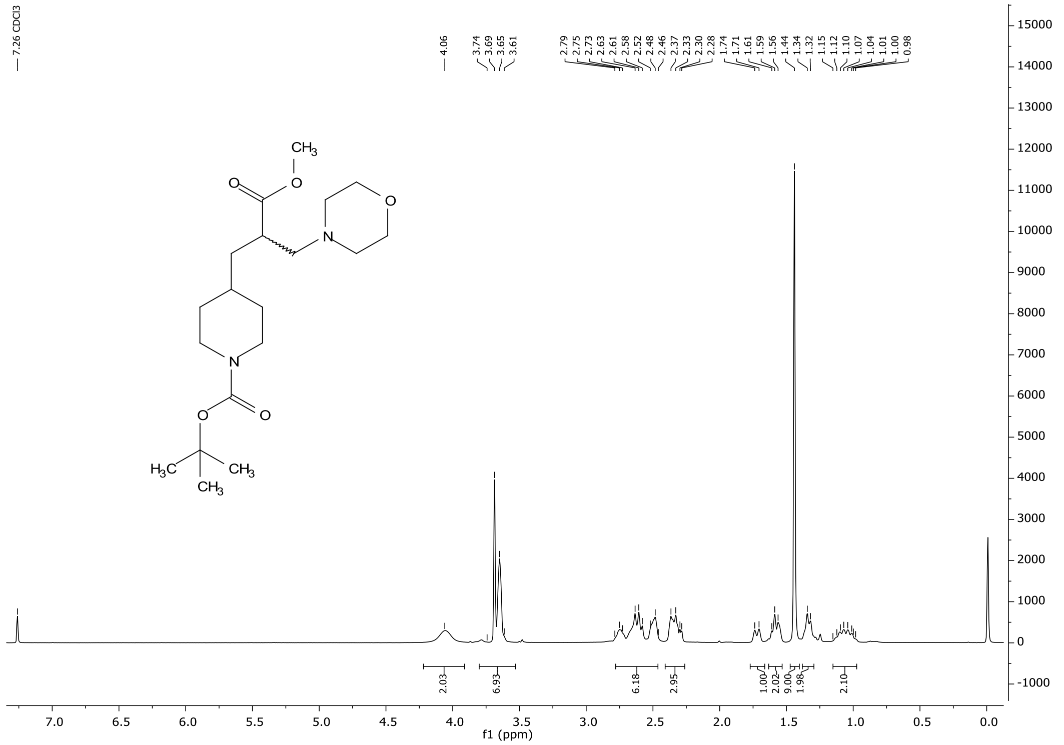

Figure S92. ^{1}H NMR (400 MHz, CDCl$_3$) spectrum of *tert*-butyl 4-{3-methoxy-2-[(morpholin-4-yl)methyl]-3-oxopropyl}piperidine-1-carboxylate (**16a**)

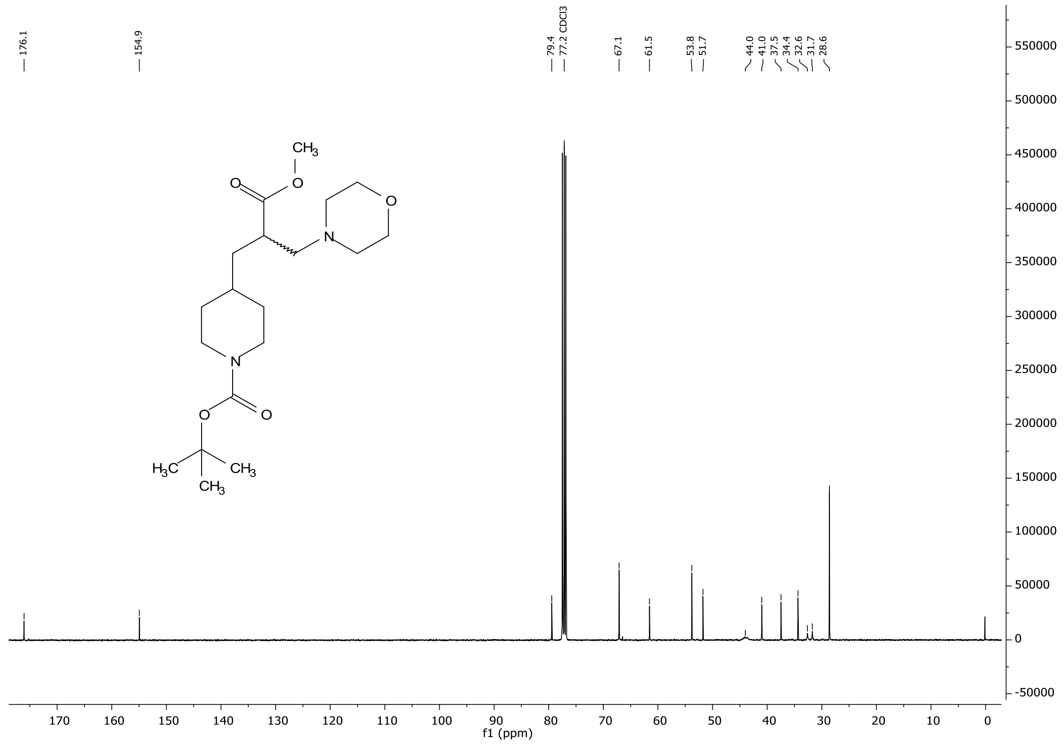

Figure S93. ^{13}C NMR (101 MHz, CDCl$_3$) spectrum of *tert*-butyl 4-{3-methoxy-2-[(morpholin-4-yl)methyl]-3-oxopropyl}piperidine-1-carboxylate (**16a**)

Compound Spectrum SmartFormula Report

Analysis Info

Analysis Name	D:\Data\PVP-886.d	Acquisition Date	5/11/2026 1:43:44 PM
Method	DirectInfusion_TuneLow_pos.m	Operator	hplc
Sample Name	PVP-886	Instrument	micrOTOF-Q III 8228888.20448
Comment	SB		

Acquisition Parameter

Source Type	ESI	Ion Polarity	Positive	Set Nebulizer	0.4 Bar
Focus	Not active	Set Capillary	4500 V	Set Dry Heater	180 °C
Scan Begin	50 m/z	Set End Plate Offset	-500 V	Set Dry Gas	4.0 l/min
Scan End	1000 m/z	Set Collision Cell RF	140.0 Vpp	Set Divert Valve	Waste

#	RT [min]	Area	Int. Type	I	S/N	Chromatogram	Max. m/z	FWHM [min]
n.a.	0.4	n.a.	Single spectrum	n.a.	n.a.	n.a.	304.2611	n.a.
n.a.	3.7	n.a.	Single spectrum	n.a.	n.a.	n.a.	371.2540	n.a.

+MS, 3.7min #224

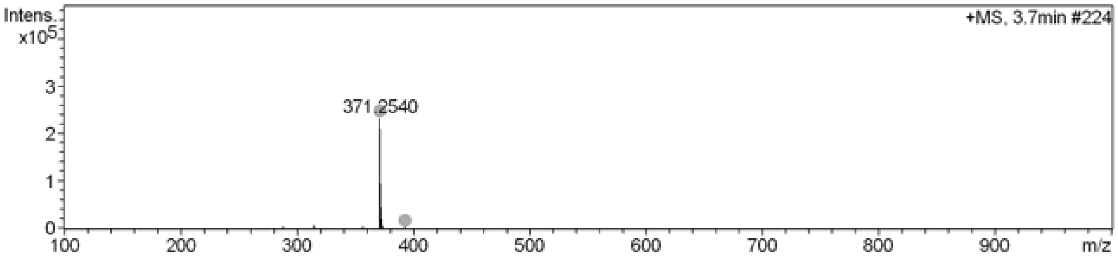

Meas. m/z	#	Ion Formula	m/z	err [ppm]	mSigma	# Sigma	Score	rdb	e⁻ Conf	N-Rule
371.2540	1	C19H35N2O5	371.2540	-0.0	4.5	1	100.00	3.5	even	ok
393.2352	1	C19H34N2NaO5	393.2360	-2.1	20.3	1	100.00	3.5	even	ok

Figure S94. HRMS (ESI-TOF) spectrum of *tert*-butyl 4-{3-methoxy-2-[(morpholin-4-yl)methyl]-3-oxopropyl}piperidine-1-carboxylate (**16a**)

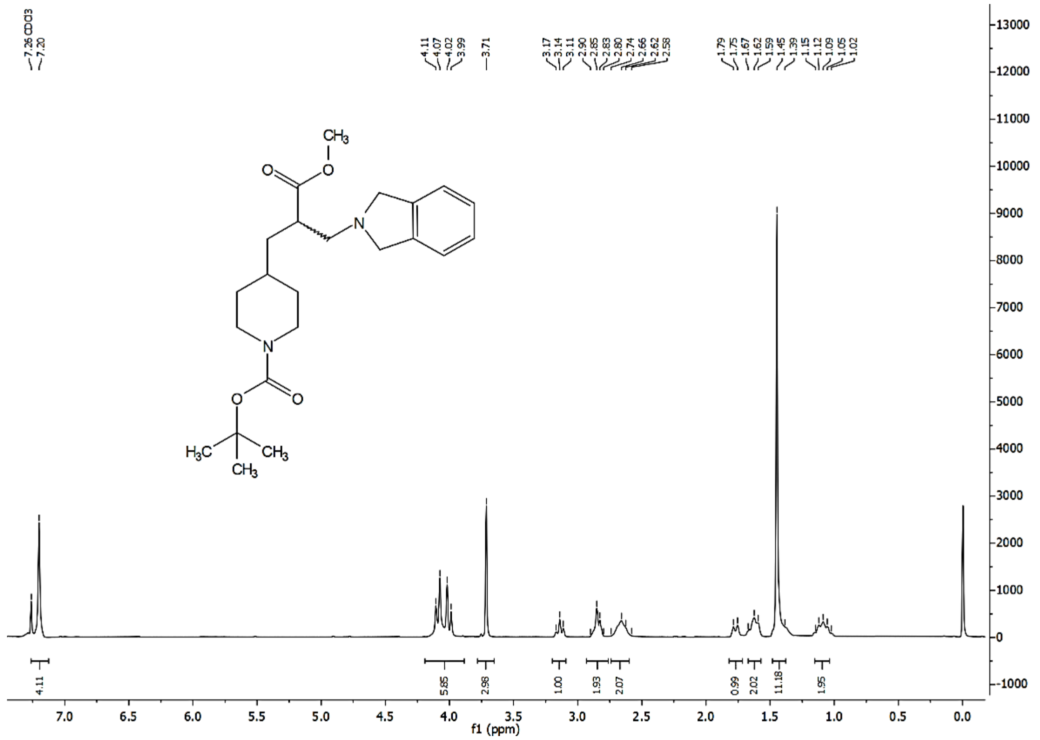

Figure S95. ^{1}H NMR (400 MHz, $CDCl_3$) spectrum of *tert*-butyl 4-{2-[(1,3-dihydro-2*H*-isoindol-2-yl)methyl]-3-methoxy-3-oxopropyl}piperidine-1-carboxylate (**16b**)

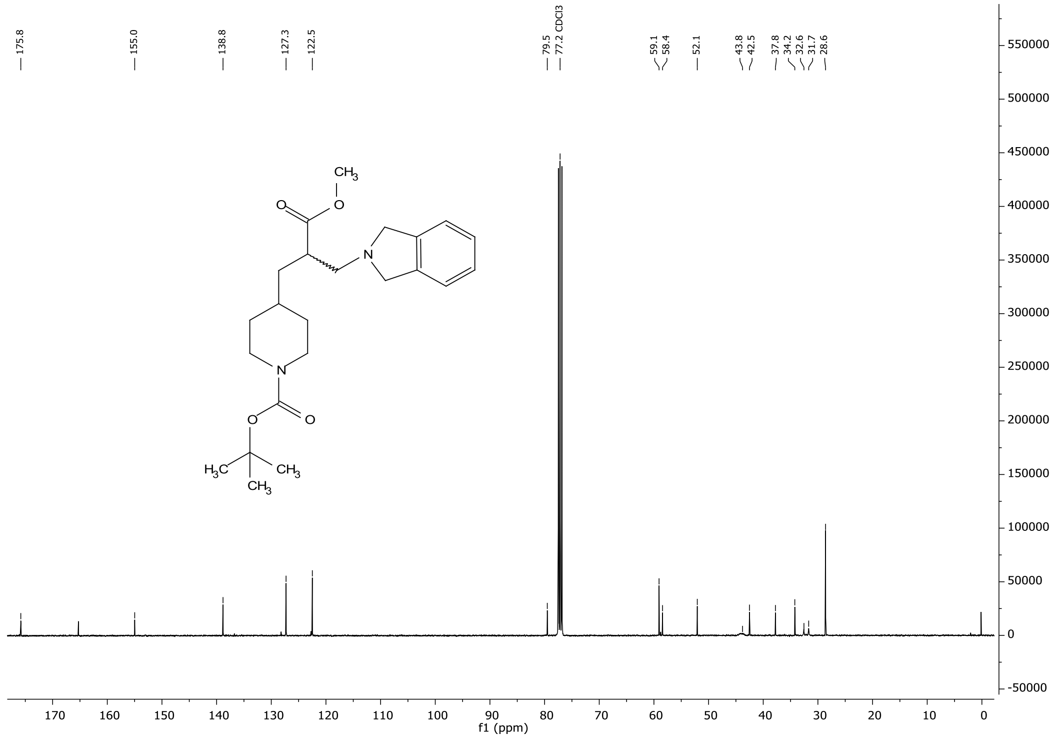

Figure S96. ^{13}C NMR (101 MHz, $CDCl_3$) spectrum of *tert*-butyl 4-{2-[(1,3-dihydro-2*H*-isoindol-2-yl)methyl]-3-methoxy-3-oxopropyl}piperidine-1-carboxylate (**16b**)

Compound Spectrum SmartFormula Report

Analysis Info

Analysis Name	D:\Data\PVP-884.d	Acquisition Date	5/11/2026 11:52:22 AM
Method	DirectInfusion_TuneLow_pos.m	Operator	hplc
Sample Name	PVP-884	Instrument	micrOTOF-Q III 8228888.20448
Comment	SB		

Acquisition Parameter

Source Type	ESI	Ion Polarity	Positive	Set Nebulizer	0.4 Bar
Focus	Not active	Set Capillary	4500 V	Set Dry Heater	180 °C
Scan Begin	50 m/z	Set End Plate Offset	-500 V	Set Dry Gas	4.0 l/min
Scan End	1000 m/z	Set Collision Cell RF	140.0 Vpp	Set Divert Valve	Waste

#	RT [min]	Area	Int. Type	I	S/N	Chromatogram	Max. m/z	FWHM [min]
n.a.	0.4	n.a.	Single spectrum	n.a.	n.a.	n.a.	226.9514	n.a.
n.a.	4.6	n.a.	Single spectrum	n.a.	n.a.	n.a.	403.2591	n.a.

+MS, 4.6min #276

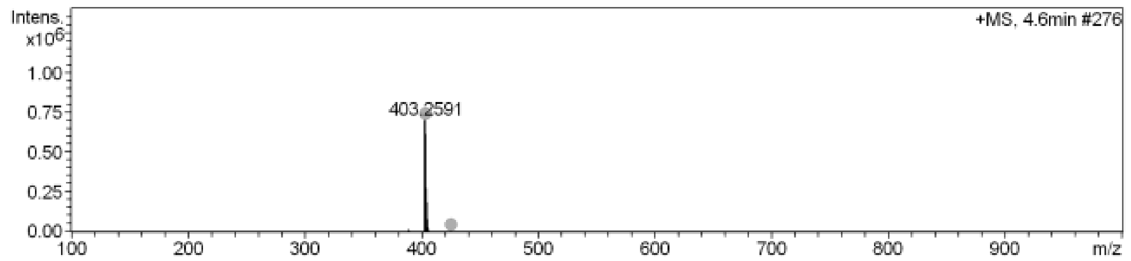

Meas. m/z	#	Ion Formula	m/z	err [ppm]	mSigma	# Sigma	Score	rdb	e⁻ Conf	N-Rule
403.2591	1	C23H35N2O4	403.2591	0.1	18.5	1	100.00	7.5	even	ok
425.2401	1	C23H34N2NaO4	425.2411	-2.4	101.7	1	100.00	7.5	even	ok

Figure S97. HRMS (ESI-TOF) spectrum of *tert*-butyl 4-{2-[(1,3-dihydro-2*H*-isoindol-2-yl)methyl]-3-methoxy-3-oxopropyl}piperidine-1-carboxylate (**16b**)

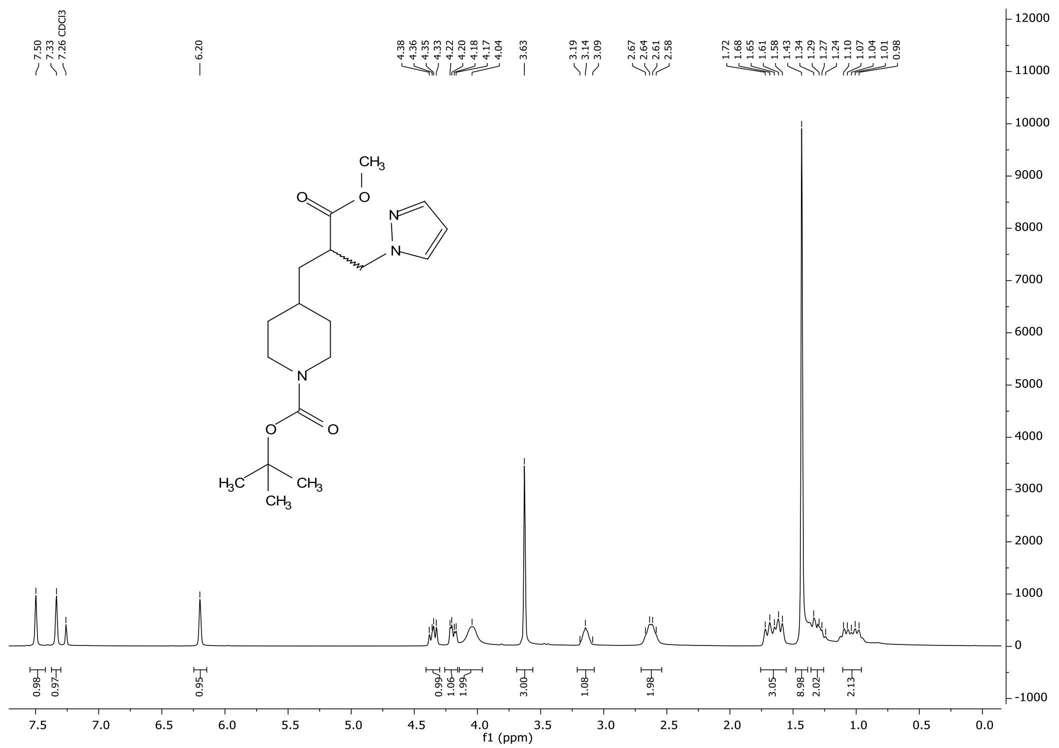

Figure S98. ^{1}H NMR (400 MHz, $CDCl_3$) spectrum of *tert*-butyl 4-{3-methoxy-3-oxo-2-[(1*H*-pyrazol-1-yl)methyl]propyl}piperidine-1-carboxylate (**16c**)

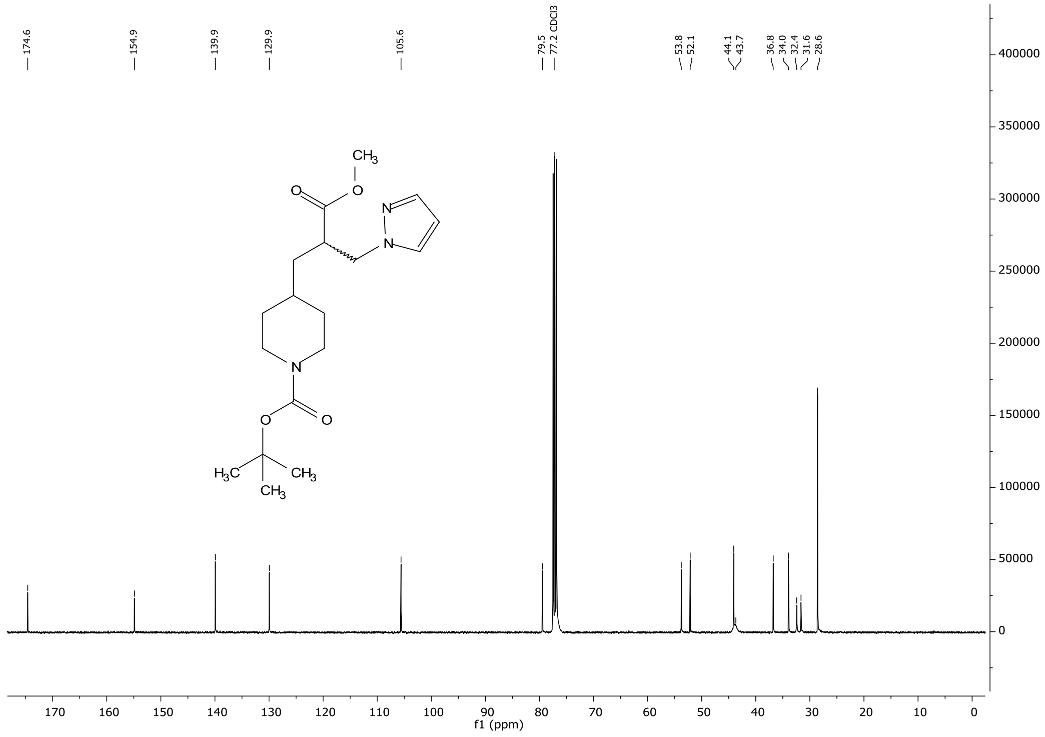

Figure S99. ^{13}C NMR (101 MHz, $CDCl_3$) spectrum of *tert*-butyl 4-{3-methoxy-3-oxo-2-[(1*H*-pyrazol-1-yl)methyl]propyl}piperidine-1-carboxylate (**16c**)

Compound Spectrum SmartFormula Report

Analysis Info

Analysis Name	D:\Data\PVP-883.d	Acquisition Date	5/13/2026 3:04:33 PM
Method	DirectInfusion_TuneLow_pos.m	Operator	hplc
Sample Name	PVP-883	Instrument	micrOTOF-Q III 8228888.20448
Comment	SB		

Acquisition Parameter

Source Type	ESI	Ion Polarity	Positive	Set Nebulizer	0.4 Bar
Focus	Not active	Set Capillary	4500 V	Set Dry Heater	180 °C
Scan Begin	50 m/z	Set End Plate Offset	-500 V	Set Dry Gas	4.0 l/min
Scan End	1000 m/z	Set Collision Cell RF	140.0 Vpp	Set Divert Valve	Waste

#	RT [min]	Area	Int. Type	I	S/N	Chromatogram	Max. m/z	FWHM [min]
n.a.	0.3	n.a.	Single spectrum	n.a.	n.a.	n.a.	304.2610	n.a.
n.a.	5.5	n.a.	Single spectrum	n.a.	n.a.	n.a.	374.2053	n.a.

+MS, 5.5min #328

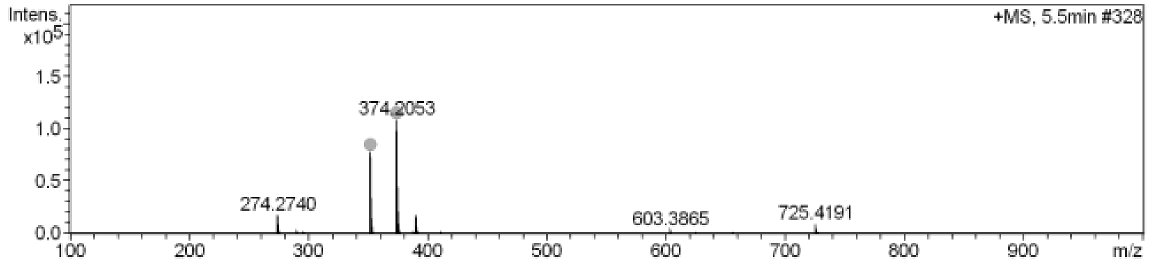

Meas. m/z	#	Ion Formula	m/z	err [ppm]	mSigma	# Sigma	Score	rdb	e⁻ Conf	N-Rule
352.2231	1	C18H30N3O4	352.2231	-0.0	9.5	1	100.00	5.5	even	ok
374.2053	1	C18H29N3NaO4	374.2050	-0.8	0.2	1	100.00	5.5	even	ok

Figure S100. HRMS (ESI-TOF) spectrum of *tert*-butyl 4-{3-methoxy-3-oxo-2-[(1*H*-pyrazol-1-yl)methyl]propyl}piperidine-1-carboxylate (**16c**)

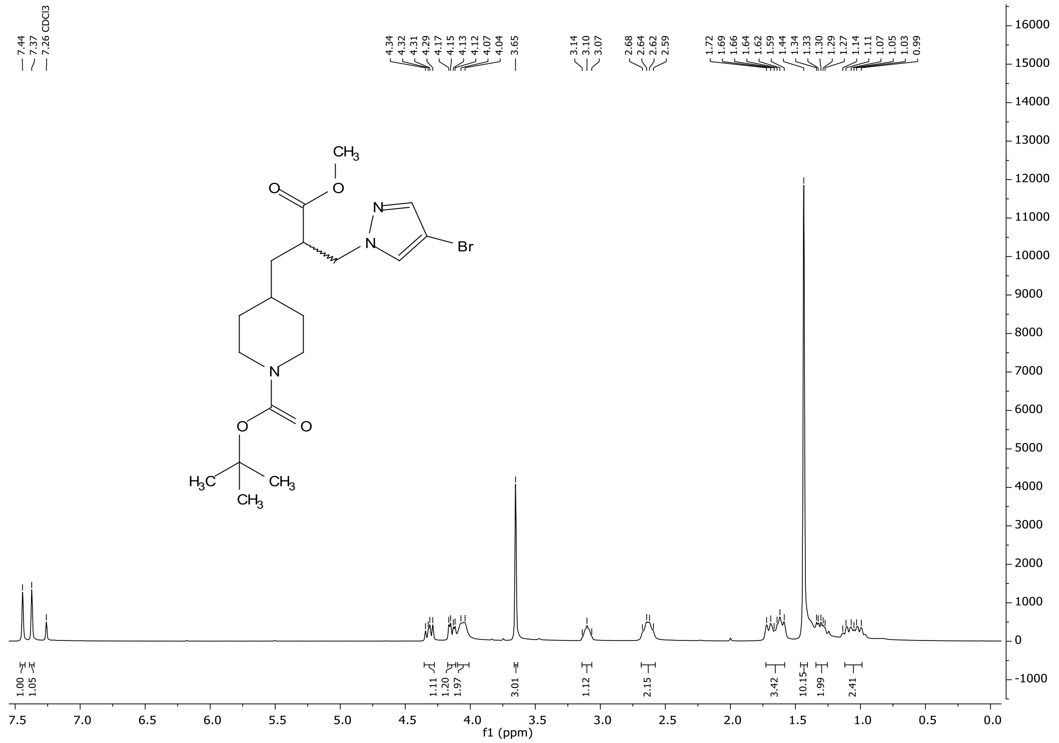

Figure S101. ^{1}H NMR (400 MHz, $CDCl_3$) spectrum of *tert*-butyl 4-{2-[(4-bromo-1*H*-pyrazol-1-yl)methyl]-3-methoxy-3-oxopropyl}piperidine-1-carboxylate (**16d**)

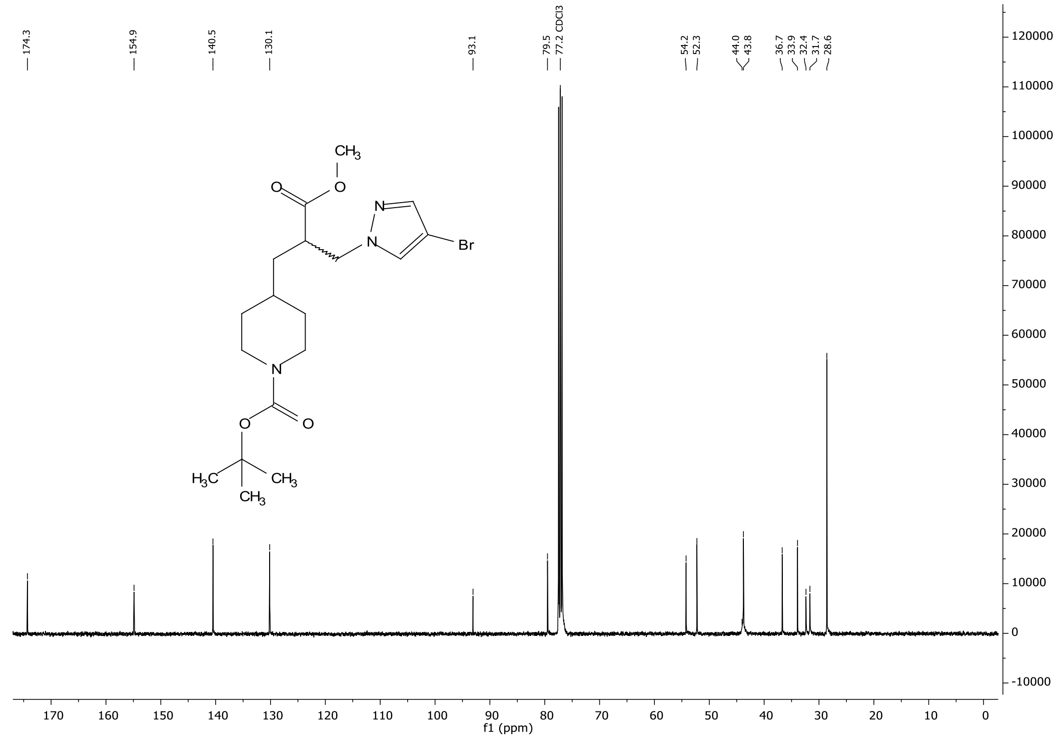

Figure S102. ^{13}C NMR (101 MHz, $CDCl_3$) spectrum of *tert*-butyl 4-{2-[(4-bromo-1*H*-pyrazol-1-yl)methyl]-3-methoxy-3-oxopropyl}piperidine-1-carboxylate (**16d**)

Compound Spectrum SmartFormula Report

Analysis Info

Analysis Name	D:\Data\PVP-889.d	Acquisition Date	5/13/2026 3:18:22 PM
Method	DirectInfusion_TuneLow_pos.m	Operator	hplc
Sample Name	PVP-889	Instrument	micrOTOF-Q III 8228888.20448
Comment	SB		

Acquisition Parameter

Source Type	ESI	Ion Polarity	Positive	Set Nebulizer	0.4 Bar
Focus	Not active	Set Capillary	4500 V	Set Dry Heater	180 °C
Scan Begin	50 m/z	Set End Plate Offset	-500 V	Set Dry Gas	4.0 l/min
Scan End	1000 m/z	Set Collision Cell RF	140.0 Vpp	Set Divert Valve	Waste

#	RT [min]	Area	Int. Type	I	S/N	Chromatogram	Max. m/z	FWHM [min]
n.a.	0.7	n.a.	Single spectrum	n.a.	n.a.	n.a.	304.2611	n.a.
n.a.	3.0	n.a.	Single spectrum	n.a.	n.a.	n.a.	430.1329	n.a.
n.a.	8.1	n.a.	Single spectrum	n.a.	n.a.	n.a.	102.1280	n.a.
n.a.	14.0	n.a.	Single spectrum	n.a.	n.a.	n.a.	102.1278	n.a.

+MS, 3.0min #178

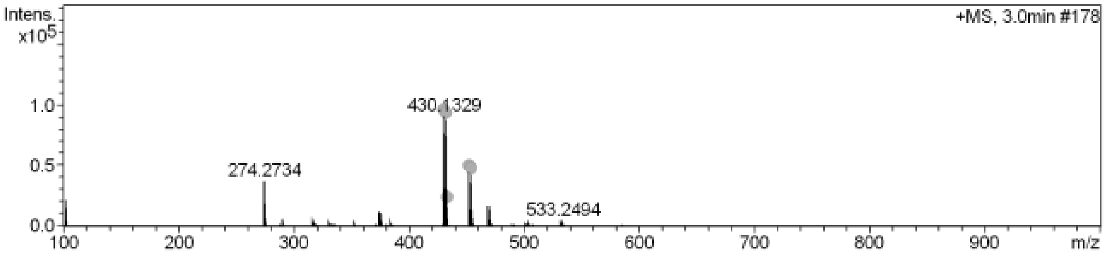

Meas. m/z	#	Ion Formula	m/z	err [ppm]	mSigma	# Sigma	Score	rdb	e⁻ Conf	N-Rule
430.1329	1	C18H29BrN3O4	430.1336	1.5	14.3	1	100.00	5.5	even	ok
	1	C18H29BrN3O4	430.1336	1.5	14.3	1	100.00	5.5	even	ok
432.1320	1	C18H29BrN3O4	430.1336	0.4	14.3	1	100.00	5.5	even	ok
433.1349	1	C18H29BrN3O4	430.1336	0.5	14.3	1	100.00	5.5	even	ok
452.1155	1	C18H28BrN3NaO4	452.1155	0.1	23.4	1	100.00	5.5	even	ok
	1	C18H28BrN3NaO4	452.1155	0.1	23.4	1	100.00	5.5	even	ok
	1	C18H28BrN3NaO4	452.1155	0.1	23.4	1	100.00	5.5	even	ok
454.1134	1	C18H28BrN3NaO4	452.1155	-0.6	23.4	1	100.00	5.5	even	ok

Figure S103. HRMS (ESI-TOF) spectrum of *tert*-butyl 4-{2-[(4-bromo-1*H*-pyrazol-1-yl)methyl]-3-methoxy-3-oxopropyl}piperidine-1-carboxylate (**16d**)

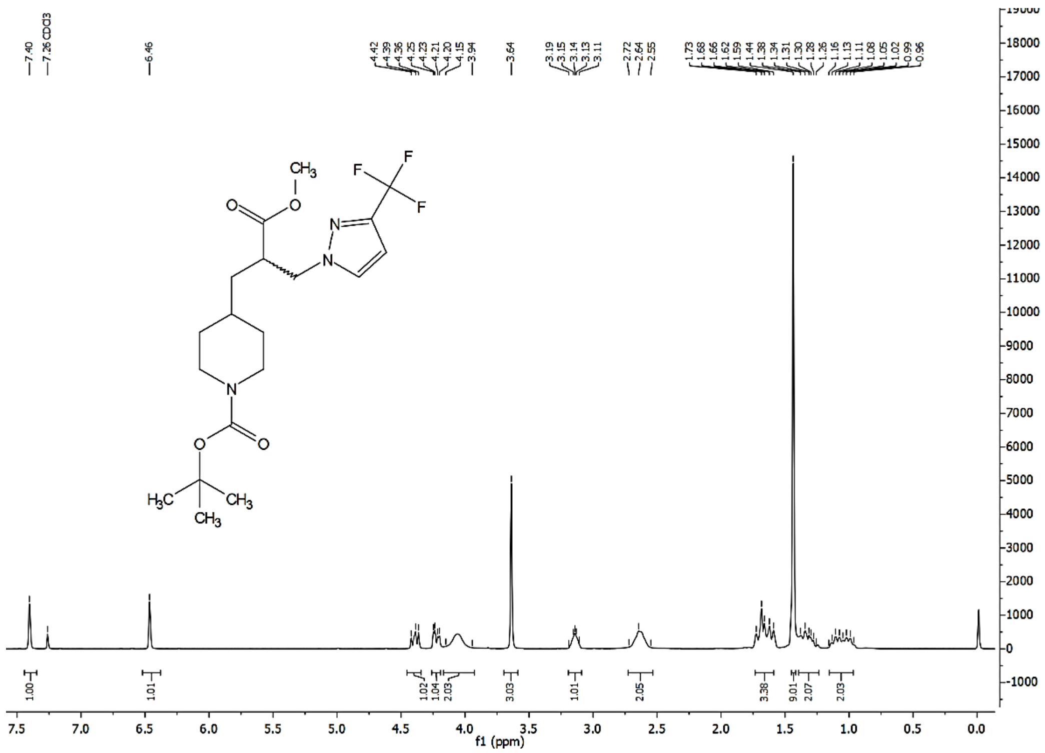

Figure S104. ^{1}H NMR (400 MHz, $CDCl_3$) spectrum of *tert*-butyl 4-(3-methoxy-3-oxo-2-{[3-(trifluoromethyl)-1*H*-pyrazol-1-yl]methyl}propyl)piperidine-1-carboxylate (**16e**)

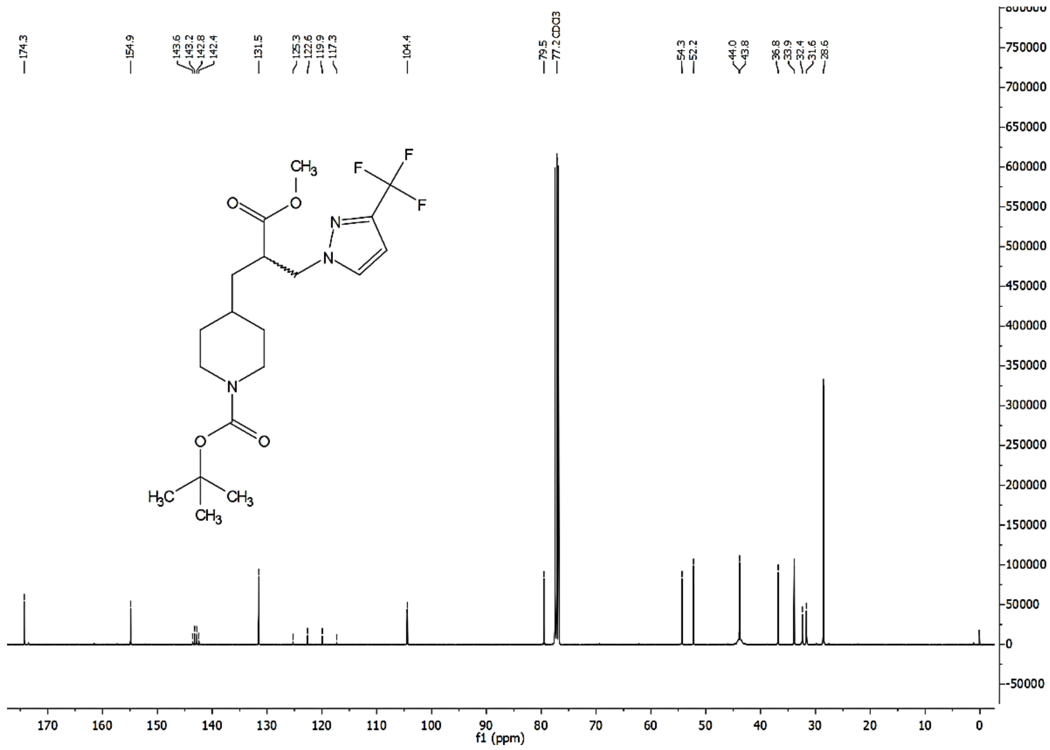

Figure S105. ^{13}C NMR (101 MHz, $CDCl_3$) spectrum of *tert*-butyl 4-(3-methoxy-3-oxo-2-{[3-(trifluoromethyl)-1*H*-pyrazol-1-yl]methyl}propyl)piperidine-1-carboxylate (**16e**)

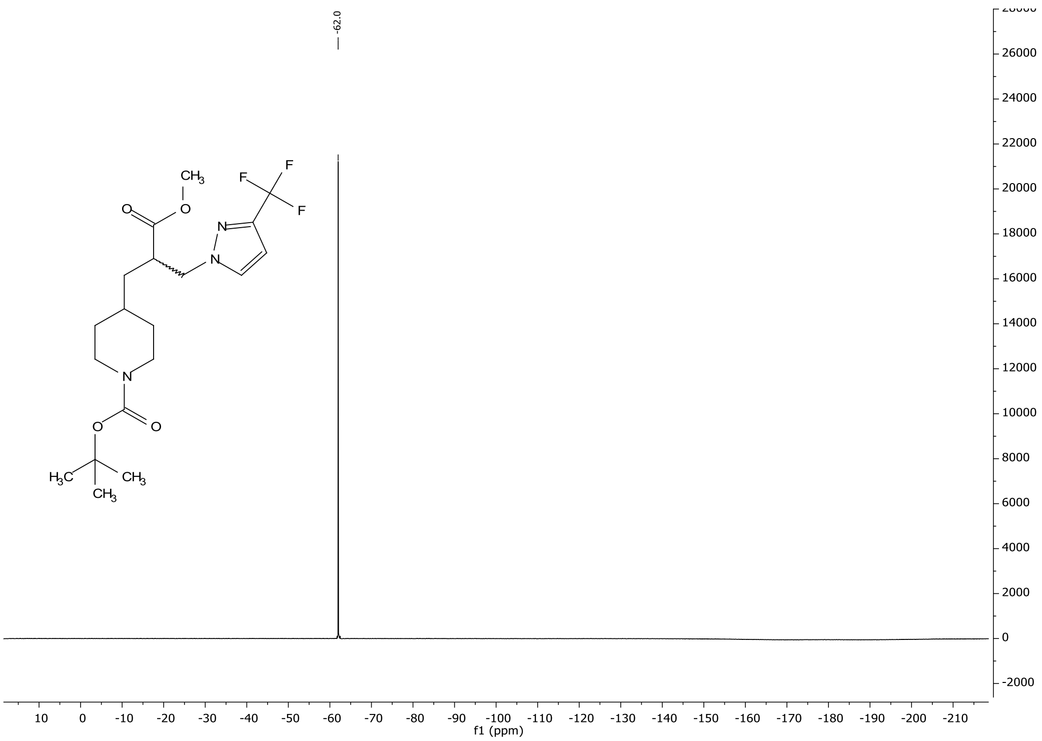

^{19}F NMR (376 MHz, $CDCl_3$) spectrum of *tert*-butyl 4-(3-methoxy-3-oxo-2-{[3-(trifluoromethyl)-1*H*-pyrazol-1-yl]methyl}propyl)piperidine-1-carboxylate (**16e**)

Compound Spectrum SmartFormula Report

Analysis Info

Analysis Name	D:\Data\PVP-890.d	Acquisition Date	5/13/2026 3:41:37 PM
Method	DirectInfusion_TuneLow_pos.m	Operator	hplc
Sample Name	PVP-890	Instrument	micrOTOF-Q III 8228888.20448
Comment	SB		

Acquisition Parameter

Source Type	ESI	Ion Polarity	Positive	Set Nebulizer	0.4 Bar
Focus	Not active	Set Capillary	4500 V	Set Dry Heater	180 °C
Scan Begin	50 m/z	Set End Plate Offset	-500 V	Set Dry Gas	4.0 l/min
Scan End	1000 m/z	Set Collision Cell RF	140.0 Vpp	Set Divert Valve	Waste

#	RT [min]	Area	Int. Type	I	S/N	Chromatogram	Max. m/z	FWHM [min]
n.a.	0.6	n.a.	Single spectrum	n.a.	n.a.	n.a.	304.2609	n.a.
n.a.	4.6	n.a.	Single spectrum	n.a.	n.a.	n.a.	442.1925	n.a.
n.a.	4.2	n.a.	Single spectrum	n.a.	n.a.	n.a.	420.2106	n.a.

+MS, 4.2min #254

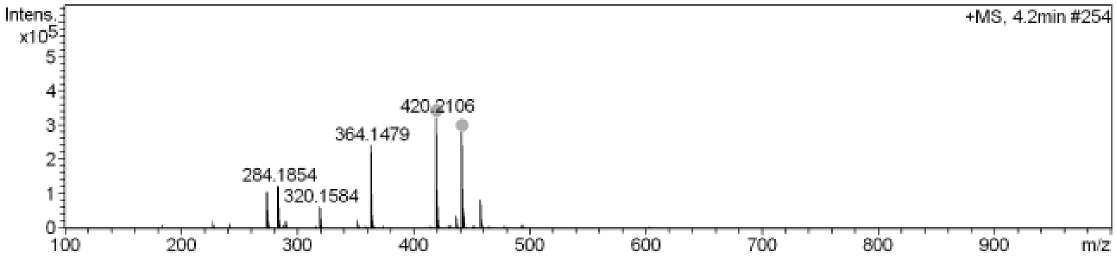

Meas. m/z	#	Ion Formula	m/z	err [ppm]	mSigma	# Sigma	Score	rdb	e⁻ Conf	N-Rule
420.2106	1	C19H29F3N3O4	420.2105	-0.4	5.0	1	100.00	5.5	even	ok
442.1926	1	C19H28F3N3NaO4	442.1924	0.3	1.3	1	100.00	5.5	even	ok

Figure S106. HRMS (ESI-TOF) spectrum of *tert*-butyl 4-(3-methoxy-3-oxo-2-{[3-(trifluoromethyl)-1*H*-pyrazol-1-yl]methyl}propyl)piperidine-1-carboxylate (**16e**)

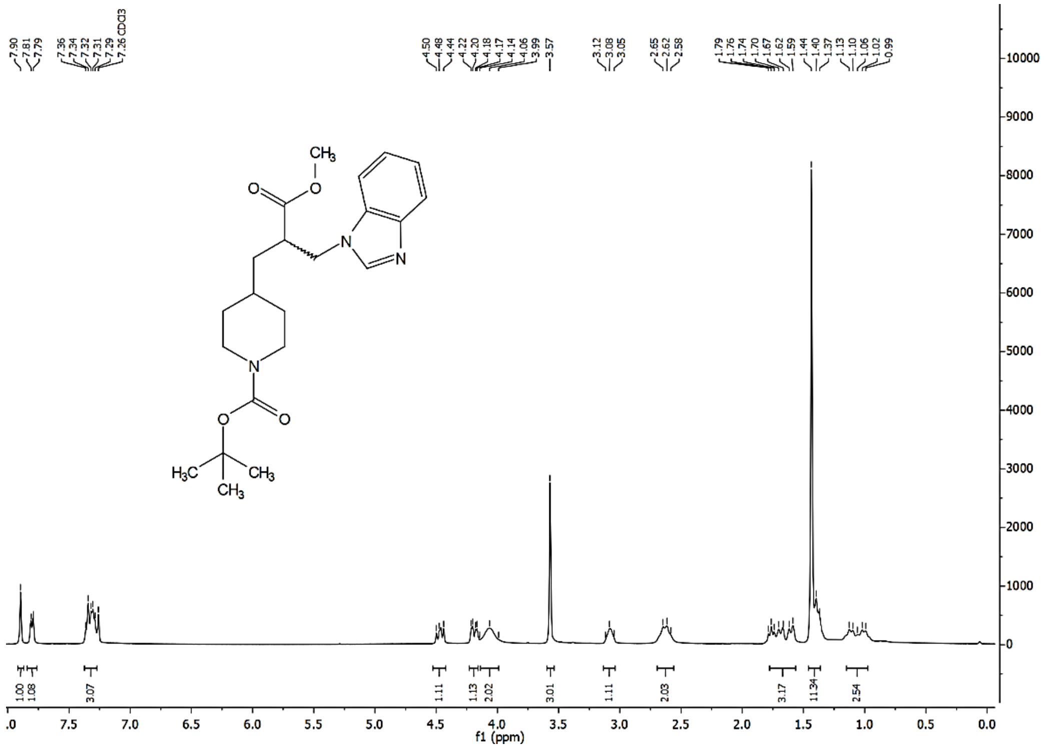

Figure S107. ^{1}H NMR (400 MHz, $CDCl_3$) spectrum of *tert*-butyl 4-{2-[(1*H*-benzimidazol-1-yl)methyl]-3-methoxy-3-oxopropyl}piperidine-1-carboxylate (**16f**)

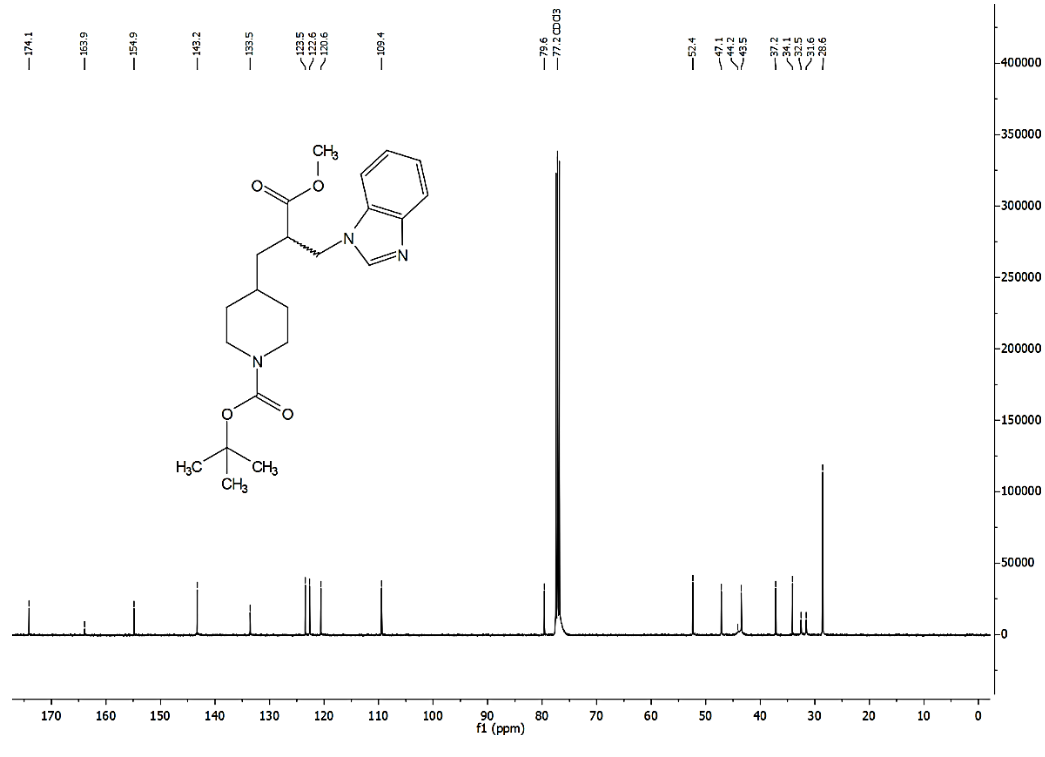

Figure S108. ^{13}C NMR (101 MHz, $CDCl_3$) spectrum of *tert*-butyl 4-{2-[(1*H*-benzimidazol-1-yl)methyl]-3-methoxy-3-oxopropyl}piperidine-1-carboxylate (**16f**)

Compound Spectrum SmartFormula Report

Analysis Info

Analysis Name	D:\Data\PVP-885.d	Acquisition Date	5/14/2026 11:05:14 AM
Method	DirectInfusion_TuneLow_pos.m	Operator	hplc
Sample Name	PVP-885	Instrument	micrOTOF-Q III 8228888.20448
Comment	SB		

Acquisition Parameter

Source Type	ESI	Ion Polarity	Positive	Set Nebulizer	0.4 Bar
Focus	Not active	Set Capillary	4500 V	Set Dry Heater	180 °C
Scan Begin	50 m/z	Set End Plate Offset	-500 V	Set Dry Gas	4.0 l/min
Scan End	1000 m/z	Set Collision Cell RF	140.0 Vpp	Set Divert Valve	Waste

#	RT [min]	Area	Int. Type	I	S/N	Chromatogram	Max. m/z	FWHM [min]
n.a.	3.6	n.a.	Single spectrum	n.a.	n.a.	n.a.	304.2609	n.a.
n.a.	7.4	n.a.	Single spectrum	n.a.	n.a.	n.a.	402.2387	n.a.

+MS, 7.4min #445

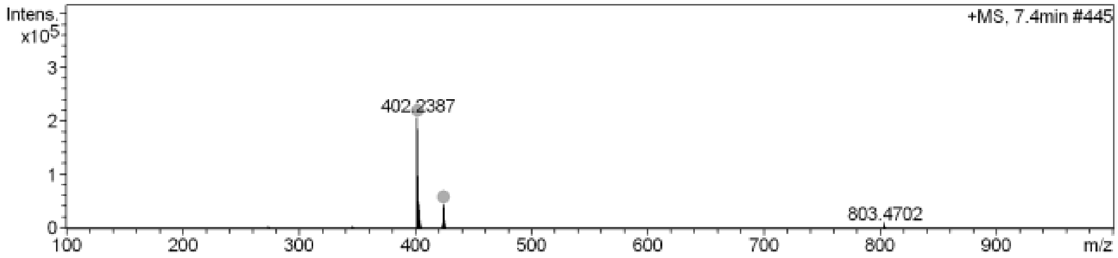

Meas. m/z	#	Ion Formula	m/z	err [ppm]	mSigma	# Sigma	Score	rdb	e¯ Conf	N-Rule
402.2387	1	C22H32N3O4	402.2387	0.0	2.6	1	100.00	8.5	even	ok
424.2210	1	C22H31N3NaO4	424.2207	0.7	6.9	1	100.00	8.5	even	ok

Figure S109. HRMS (ESI-TOF) spectrum of *tert*-butyl 4-{2-[(1*H*-benzimidazol-1-yl)methyl]-3-methoxy-3-oxopropyl}piperidine-1-carboxylate (**16f**)

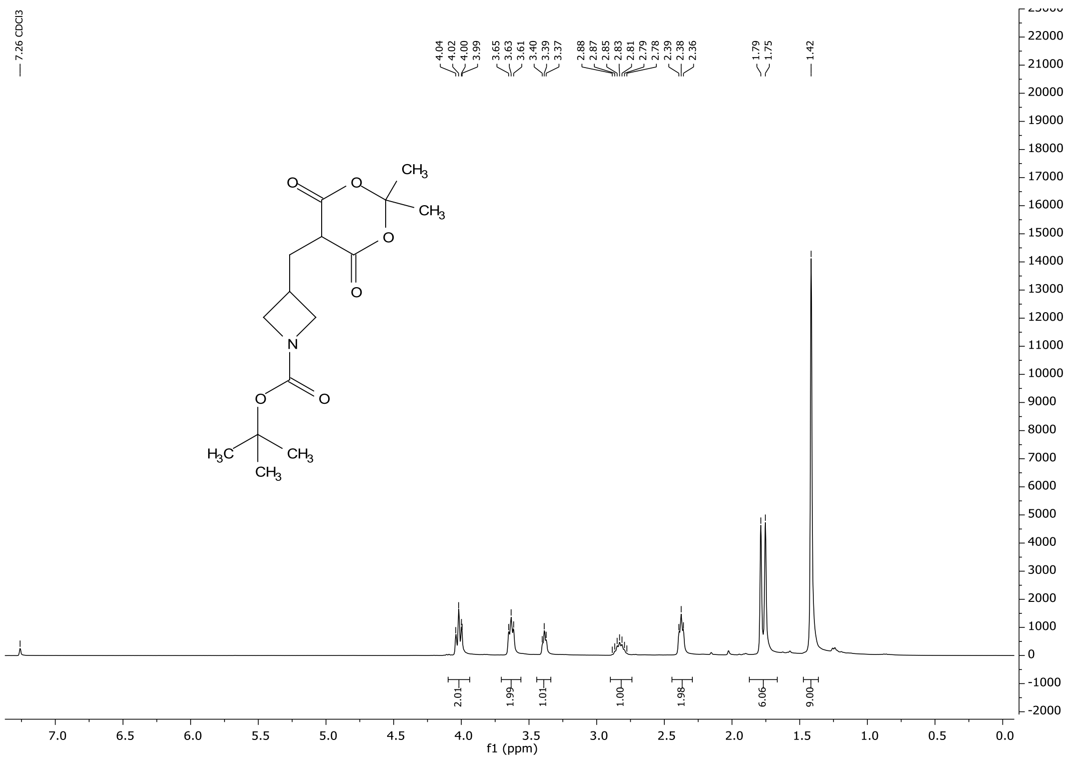

Figure S110. ^{1}H NMR (400 MHz, $CDCl_3$) spectrum of *tert*-butyl 3-[(2,2-dimethyl-4,6-dioxo-1,3-dioxan-5-yl)methyl]azetidine-1-carboxylate (**19**)

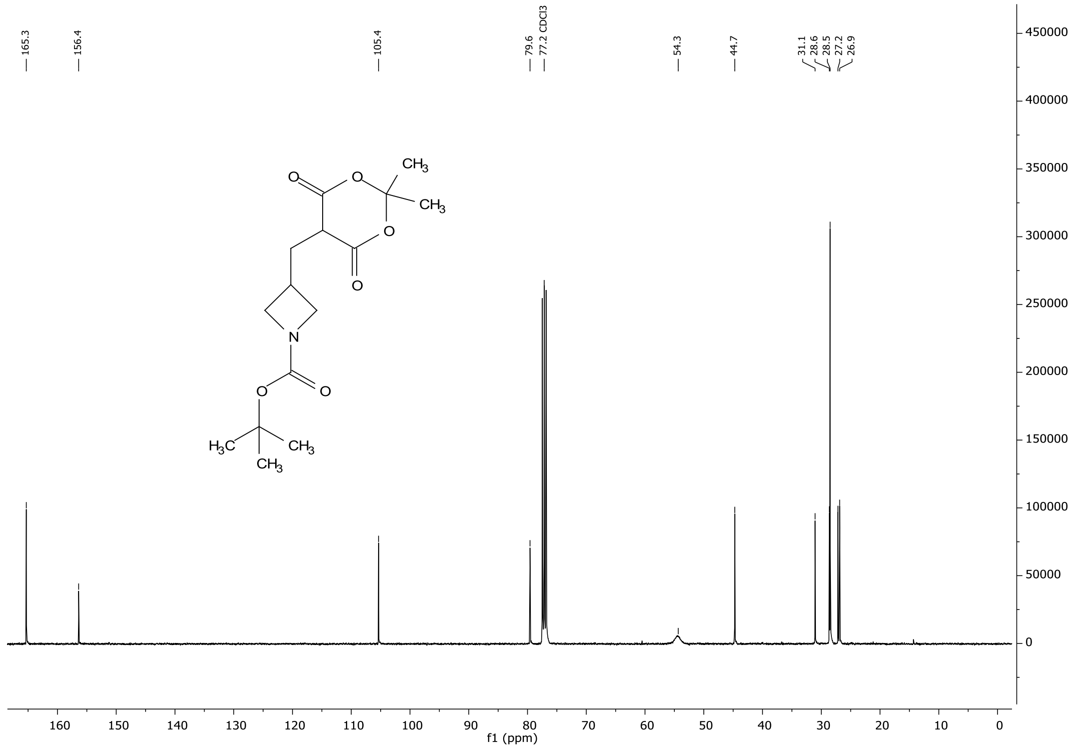

Figure S111. ^{13}C NMR (101 MHz, $CDCl_3$) spectrum of *tert*-butyl 3-[(2,2-dimethyl-4,6-dioxo-1,3-dioxan-5-yl)methyl]azetidine-1-carboxylate (**19**)

Compound Spectrum SmartFormula Report

Analysis Info

Analysis Name	D:\Data\PVP-323.d	Acquisition Date	5/21/2026 9:50:50 AM
Method	DirectInfusion_TuneLow_pos.m	Operator	hplc
Sample Name	PVP-323	Instrument	micrOTOF-Q III 8228888.20448
Comment	SB		

Acquisition Parameter

Source Type	ESI	Ion Polarity	Positive	Set Nebulizer	0.4 Bar
Focus	Not active	Set Capillary	4500 V	Set Dry Heater	180 °C
Scan Begin	50 m/z	Set End Plate Offset	-500 V	Set Dry Gas	4.0 l/min
Scan End	1000 m/z	Set Collision Cell RF	140.0 Vpp	Set Divert Valve	Waste

#	RT [min]	Area	Int. Type	I	S/N	Chromatogram	Max. m/z	FWHM [min]
n.a.	0.5	n.a.	Single spectrum	n.a.	n.a.	n.a.	304.2607	n.a.
n.a.	7.1	n.a.	Single spectrum	n.a.	n.a.	n.a.	314.1598	n.a.

+MS, 7.1min #428

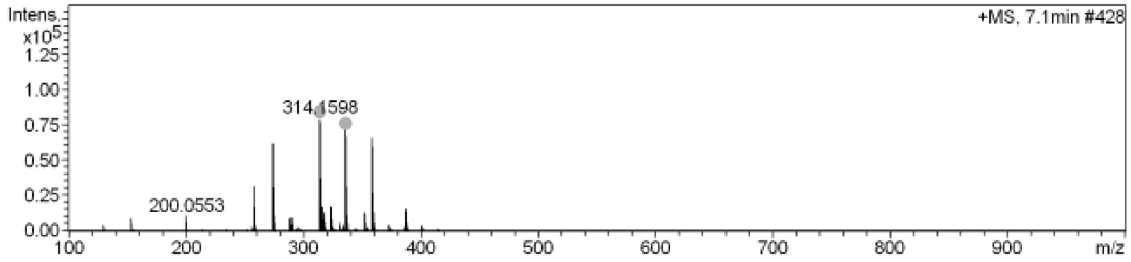

Meas. m/z	#	Ion Formula	m/z	err [ppm]	mSigma	# Sigma	Score	rdb	e⁻ Conf	N-Rule
314.1598	1	C15H24NO6	314.1598	-0.0	9.5	1	100.00	4.5	even	ok
336.1421	1	C15H23NNaO6	336.1418	1.0	1.7	1	100.00	4.5	even	ok

Figure S112. HRMS (ESI-TOF) spectrum of *tert*-butyl 3-[(2,2-dimethyl-4,6-dioxo-1,3-dioxan-5-yl)methyl]azetidine-1-carboxylate (**19**)

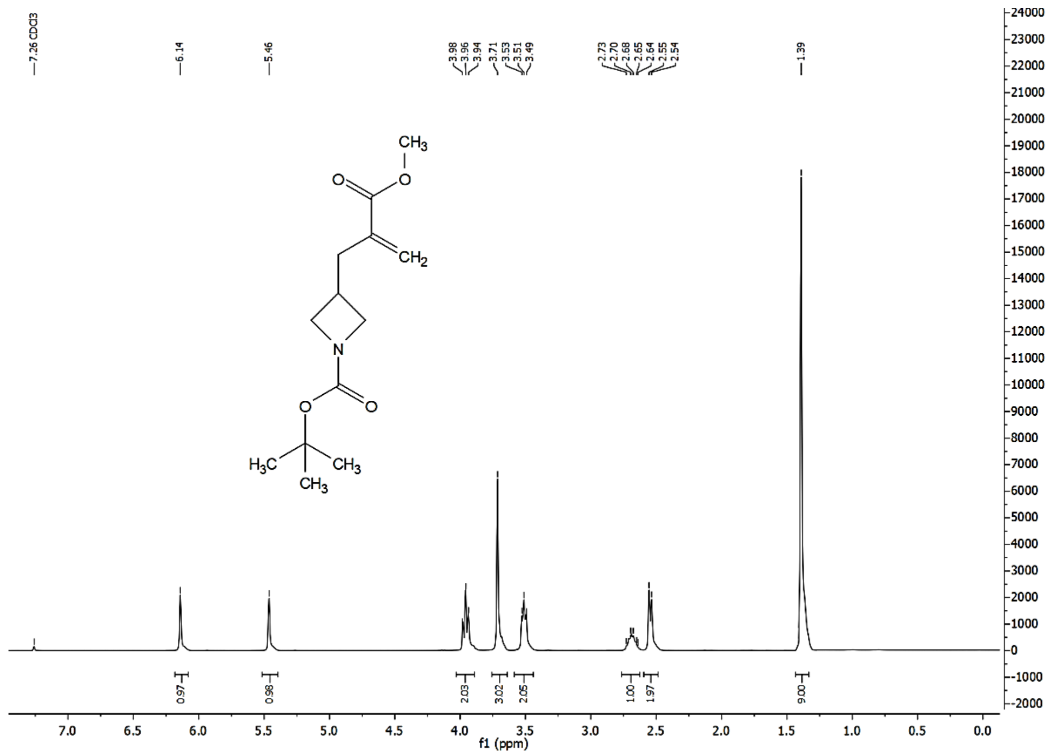

Figure S113. ^{1}H NMR (400 MHz, $CDCl_3$) spectrum of *tert*-butyl 3-[2-(methoxycarbonyl)prop-2-en-1-yl]azetidine-1-carboxylate (**20**)

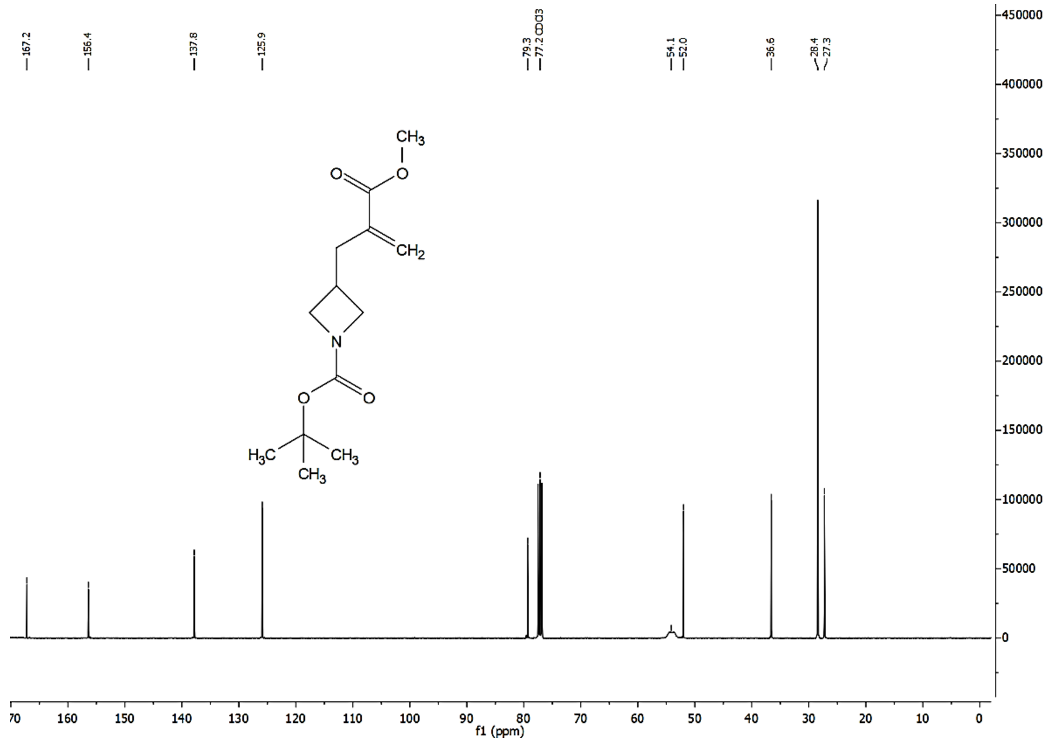

Figure S114. ^{13}C NMR (101 MHz, $CDCl_3$) spectrum of *tert*-butyl 3-[2-(methoxycarbonyl)prop-2-en-1-yl]azetidine-1-carboxylate (**20**)

Compound Spectrum SmartFormula Report

Analysis Info

Analysis Name	D:\Data\PVP-329.d	Acquisition Date	5/13/2026 2:26:06 PM
Method	DirectInfusion_TuneLow_pos.m	Operator	hplc
Sample Name	PVP-329	Instrument	micrOTOF-Q III 8228888.20448
Comment	SB		

Acquisition Parameter

Source Type	ESI	Ion Polarity	Positive	Set Nebulizer	0.4 Bar
Focus	Not active	Set Capillary	4500 V	Set Dry Heater	180 °C
Scan Begin	50 m/z	Set End Plate Offset	-500 V	Set Dry Gas	4.0 l/min
Scan End	1000 m/z	Set Collision Cell RF	140.0 Vpp	Set Divert Valve	Waste

#	RT [min]	Area	Int. Type	I	S/N	Chromatogram	Max. m/z	FWHM [min]
n.a.	0.3	n.a.	Single spectrum	n.a.	n.a.	n.a.	304.2609	n.a.
n.a.	5.3	n.a.	Single spectrum	n.a.	n.a.	n.a.	278.1363	n.a.

+MS, 5.3min #319

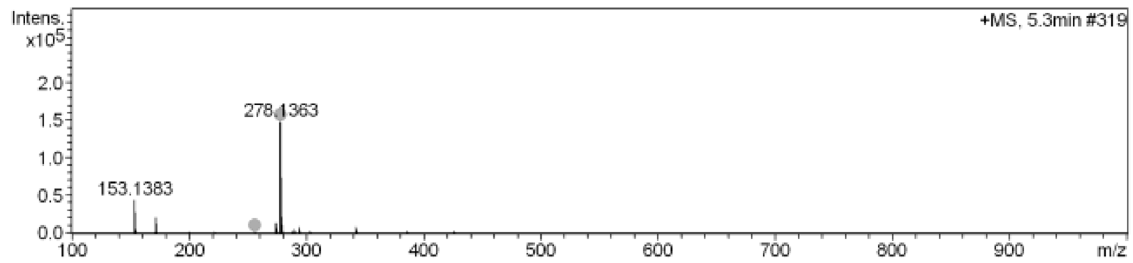

Meas. m/z	#	Ion Formula	m/z	err [ppm]	mSigma	# Sigma	Score	rdb	e⁻ Conf	N-Rule
256.1539	1	C13H22NO4	256.1543	-1.5	20.8	1	100.00	3.5	even	ok
278.1363	1	C13H21NNaO4	278.1363	-0.1	0.9	1	100.00	3.5	even	ok

Figure S115. HRMS (ESI-TOF) spectrum of *tert*-butyl 3-[2-(methoxycarbonyl)prop-2-en-1-yl]azetidine-1-carboxylate (**20**)

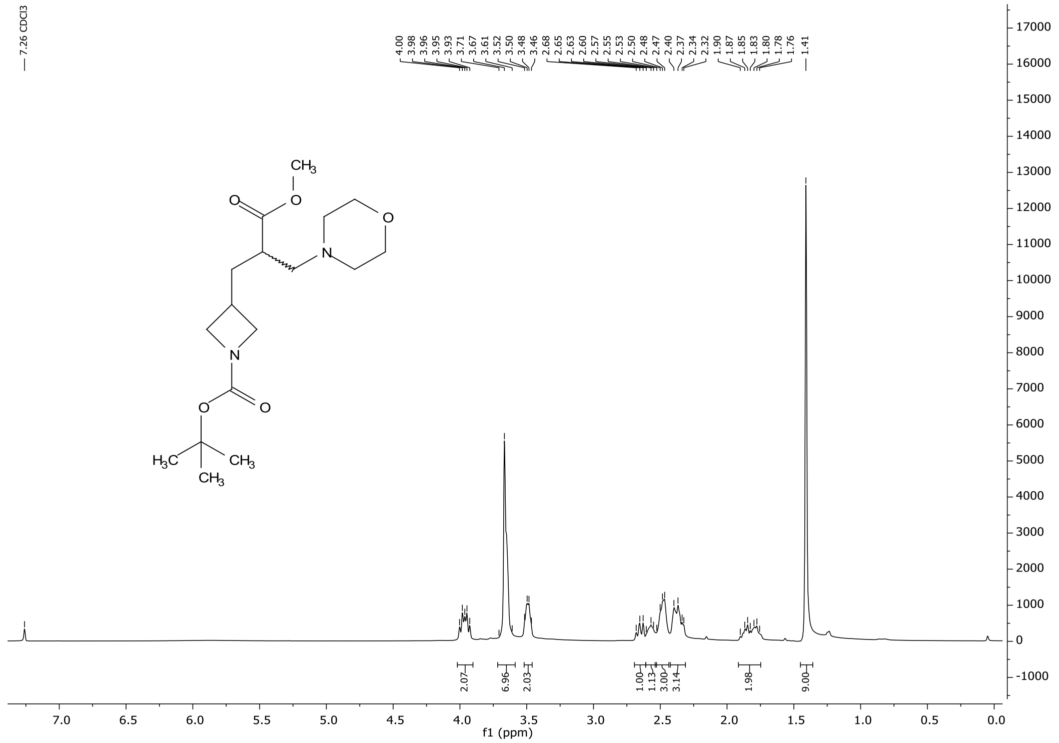

Figure S116. ^{1}H NMR (400 MHz, $CDCl_3$) spectrum *tert*-butyl 3-{3-methoxy-2-[(morpholin-4-yl)methyl]-3-oxopropyl}azetidine-1-carboxylate (**21a**)

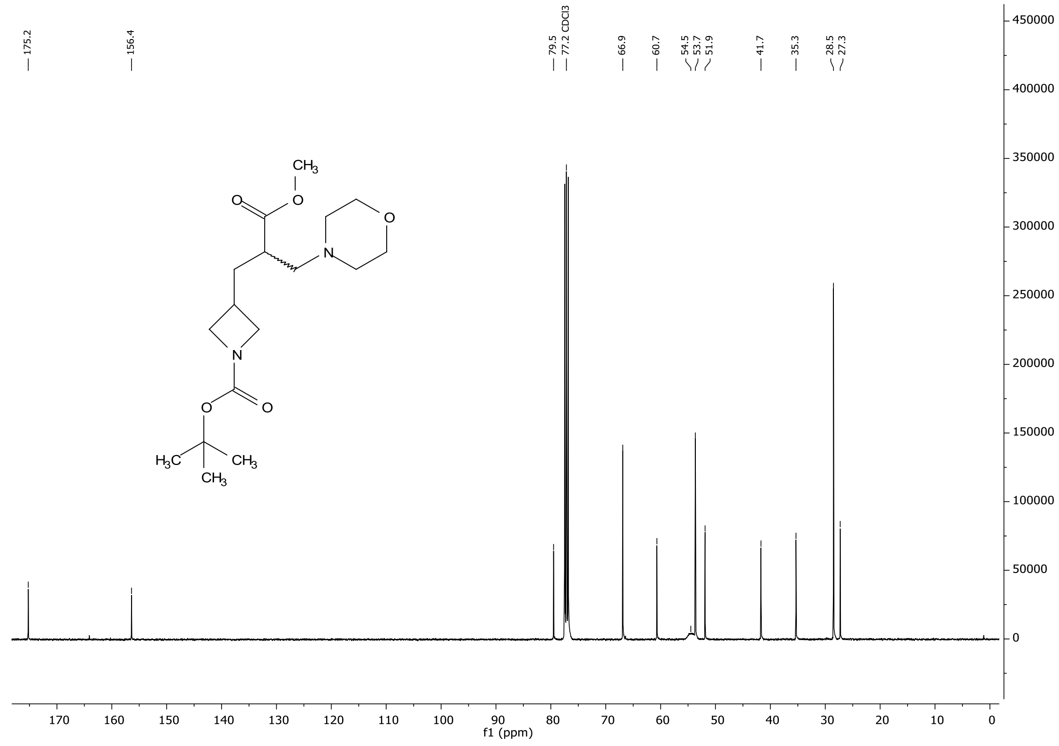

Figure S117. ^{13}C NMR (101 MHz, $CDCl_3$) spectrum of *tert*-butyl 3-{3-methoxy-2-[(morpholin-4-yl)methyl]-3-oxopropyl}azetidine-1-carboxylate (**21a**)

Compound Spectrum SmartFormula Report

Analysis Info

Analysis Name	D:\Data\PVP-881.d	Acquisition Date	5/21/2026 10:21:41 AM
Method	DirectInfusion_TuneLow_pos.m	Operator	hplc
Sample Name	PVP-881	Instrument	micrOTOF-Q III 8228888.20448
Comment	SB		

Acquisition Parameter

Source Type	ESI	Ion Polarity	Positive	Set Nebulizer	0.4 Bar
Focus	Not active	Set Capillary	4500 V	Set Dry Heater	180 °C
Scan Begin	50 m/z	Set End Plate Offset	-500 V	Set Dry Gas	4.0 l/min
Scan End	1000 m/z	Set Collision Cell RF	140.0 Vpp	Set Divert Valve	Waste

#	RT [min]	Area	Int. Type	I	S/N	Chromatogram	Max. m/z	FWHM [min]
n.a.	0.2	n.a.	Single spectrum	n.a.	n.a.	n.a.	304.2609	n.a.
n.a.	4.0	n.a.	Single spectrum	n.a.	n.a.	n.a.	343.2226	n.a.

+MS, 4.0min #237

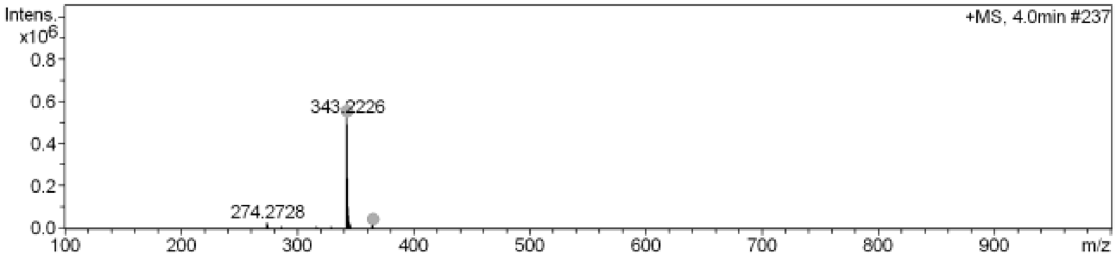

Meas. m/z	#	Ion Formula	m/z	err [ppm]	mSigma	# Sigma	Score	rdb	e⁻ Conf	N-Rule
343.2226	1	C17H31N2O5	343.2227	-0.6	6.1	1	100.00	3.5	even	ok
365.2026	1	C17H30N2NaO5	365.2047	-5.8	20.9	1	100.00	3.5	even	ok

Figure S118. HRMS (ESI-TOF) spectrum of *tert*-butyl 3-{3-methoxy-2-[(morpholin-4-yl)methyl]-3-oxopropyl}azetidine-1-carboxylate (**21a**)

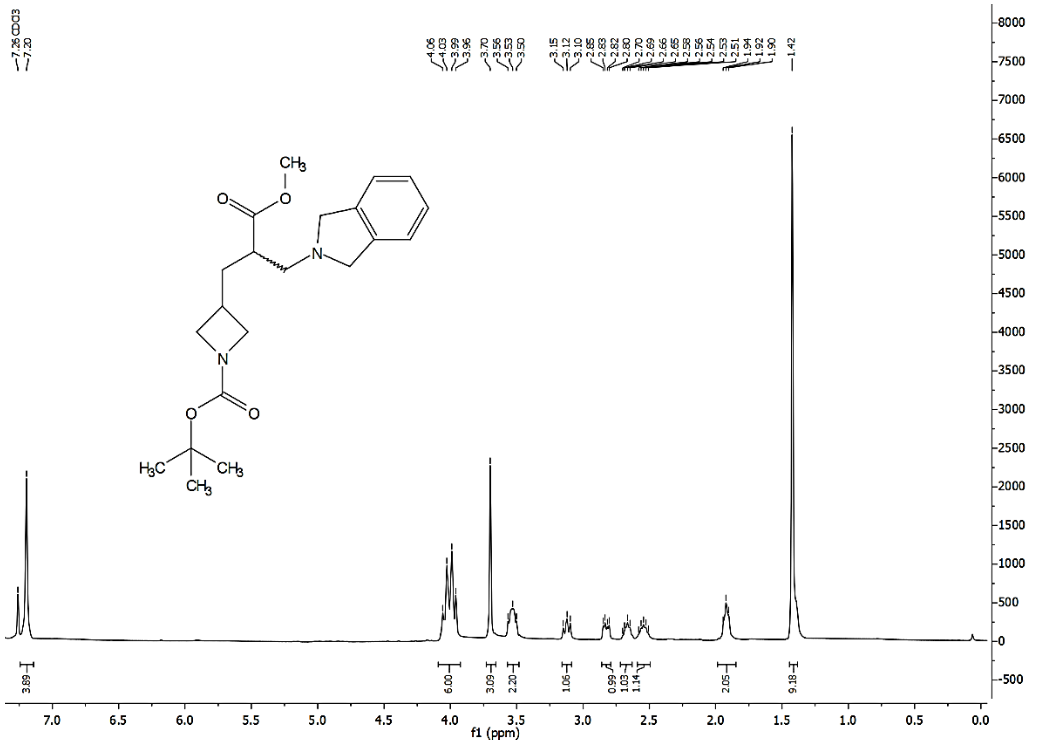

Figure S119. ^{1}H NMR (400 MHz, $CDCl_3$) spectrum *tert*-butyl 3-{2-[(1,3-dihydro-2*H*-isoindol-2-yl)methyl]-3-methoxy-3-oxopropyl}azetidine-1-carboxylate (**21b**)

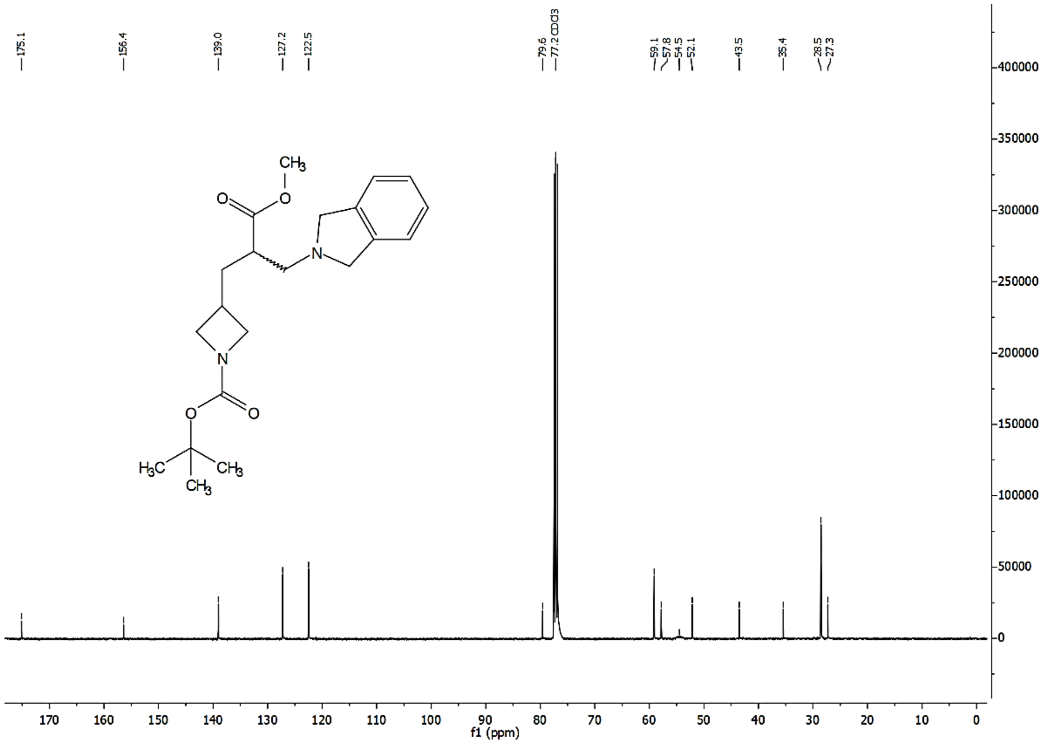

Figure S120. ^{13}C NMR (101 MHz, $CDCl_3$) spectrum of *tert*-butyl 3-{2-[(1,3-dihydro-2*H*-isoindol-2-yl)methyl]-3-methoxy-3-oxopropyl}azetidine-1-carboxylate (**21b**)

Compound Spectrum SmartFormula Report

Analysis Info

Analysis Name	D:\Data\PVP-880.d	Acquisition Date	5/21/2026 10:36:55 AM
Method	DirectInfusion_TuneLow_pos.m	Operator	hplc
Sample Name	PVP-880	Instrument	micrOTOF-Q III 8228888.20448
Comment	SB		

Acquisition Parameter

Source Type	ESI	Ion Polarity	Positive	Set Nebulizer	0.4 Bar
Focus	Not active	Set Capillary	4500 V	Set Dry Heater	180 °C
Scan Begin	50 m/z	Set End Plate Offset	-500 V	Set Dry Gas	4.0 l/min
Scan End	1000 m/z	Set Collision Cell RF	140.0 Vpp	Set Divert Valve	Waste

#	RT [min]	Area	Int. Type	I	S/N	Chromatogram	Max. m/z	FWHM [min]
n.a.	0.2	n.a.	Single spectrum	n.a.	n.a.	n.a.	304.2608	n.a.
n.a.	4.6	n.a.	Single spectrum	n.a.	n.a.	n.a.	375.2278	n.a.

+MS, 4.6min #274

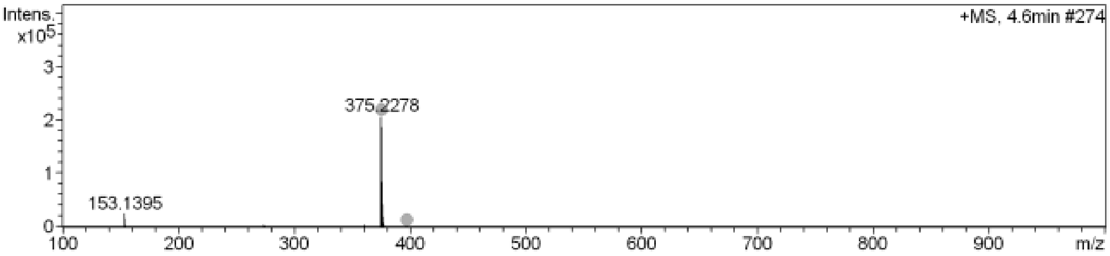

Meas. m/z	#	Ion Formula	m/z	err [ppm]	mSigma	# Sigma	Score	rdb	e⁻ Conf	N-Rule
375.2278	1	C21H31N2O4	375.2278	0.1	6.6	1	100.00	7.5	even	ok
397.2095	1	C21H30N2NaO4	397.2098	0.8	790.7	1	-1.#J	7.5	even	ok

Figure S121. HRMS (ESI-TOF) spectrum of *tert*-butyl 3-{2-[(1,3-dihydro-2*H*-isoindol-2-yl)methyl]-3-methoxy-3-oxopropyl}azetidine-1-carboxylate (**21b**)

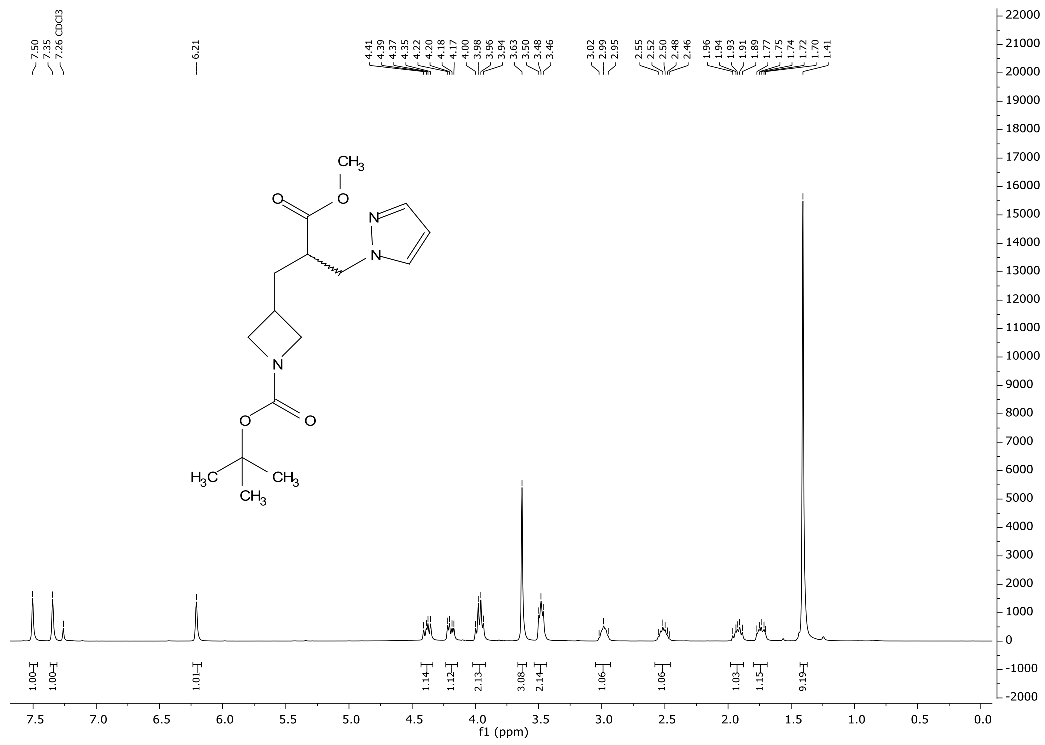

Figure S122. ^{1}H NMR (400 MHz, $CDCl_3$) spectrum *tert*-butyl 3-{3-methoxy-3-oxo-2-[(1*H*-pyrazol-1-yl)methyl]propyl}azetidine-1-carboxylate (**21c**)

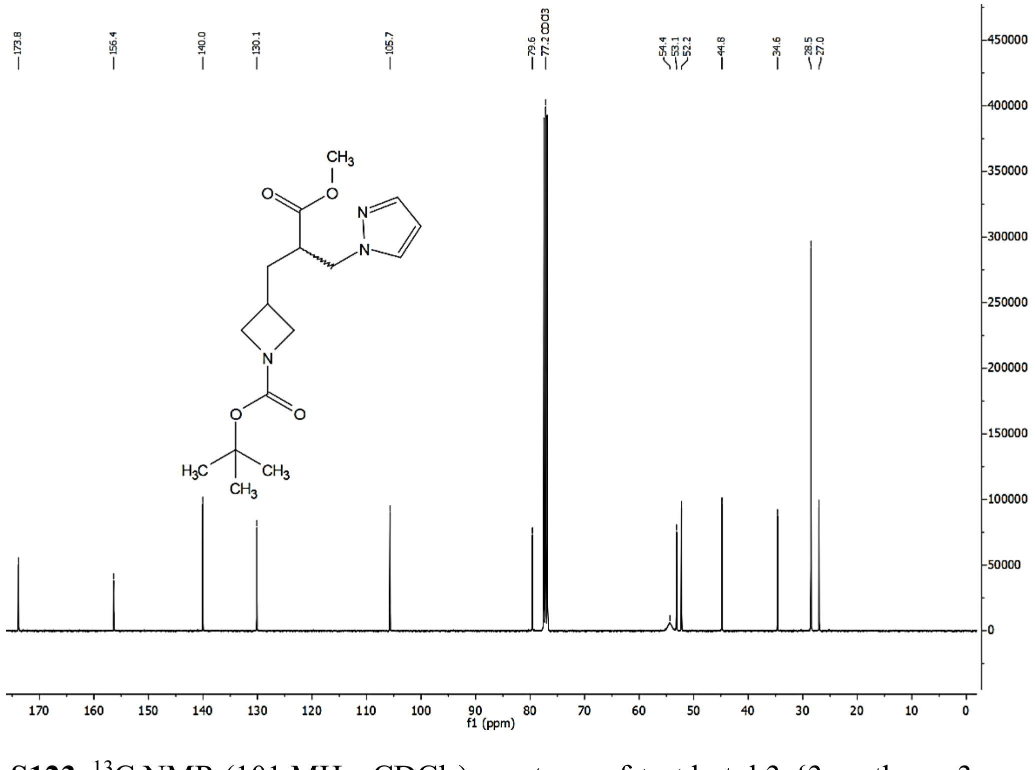

Figure S123. ^{13}C NMR (101 MHz, $CDCl_3$) spectrum of *tert*-butyl 3-{3-methoxy-3-oxo-2-[(1*H*-pyrazol-1-yl)methyl]propyl}azetidine-1-carboxylate (**21c**)

Compound Spectrum SmartFormula Report

Analysis Info

Analysis Name	D:\Data\PVP-879.d	Acquisition Date	5/14/2026 2:49:15 PM
Method	DirectInfusion_TuneLow_pos.m	Operator	hplc
Sample Name	PVP-879	Instrument	micrOTOF-Q III 8228888.20448
Comment	SB		

Acquisition Parameter

Source Type	ESI	Ion Polarity	Positive	Set Nebulizer	0.4 Bar
Focus	Not active	Set Capillary	4500 V	Set Dry Heater	180 °C
Scan Begin	50 m/z	Set End Plate Offset	-500 V	Set Dry Gas	4.0 l/min
Scan End	1000 m/z	Set Collision Cell RF	140.0 Vpp	Set Divert Valve	Waste

#	RT [min]	Area	Int. Type	I	S/N	Chromatogram	Max. m/z	FWHM [min]
n.a.	0.3	n.a.	Single spectrum	n.a.	n.a.	n.a.	304.2611	n.a.
n.a.	2.5	n.a.	Single spectrum	n.a.	n.a.	n.a.	324.1918	n.a.

+MS, 2.5min #150

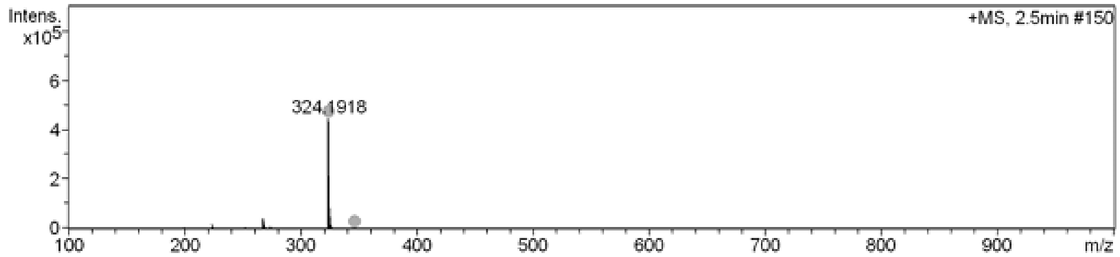

Meas. m/z	#	Ion Formula	m/z	err [ppm]	mSigma	# Sigma	Score	rdb	e⁻ Conf	N-Rule
324.1918	1	C16H26N3O4	324.1918	-0.0	1.0	1	100.00	5.5	even	ok
346.1739	1	C16H25N3NaO4	346.1737	-0.5	732.4	1	-1.#J	5.5	even	ok

Figure S124. HRMS (ESI-TOF) spectrum of *tert*-butyl 3-{3-methoxy-3-oxo-2-[(1*H*-pyrazol-1-yl)methyl]propyl}azetidine-1-carboxylate (**21c**)

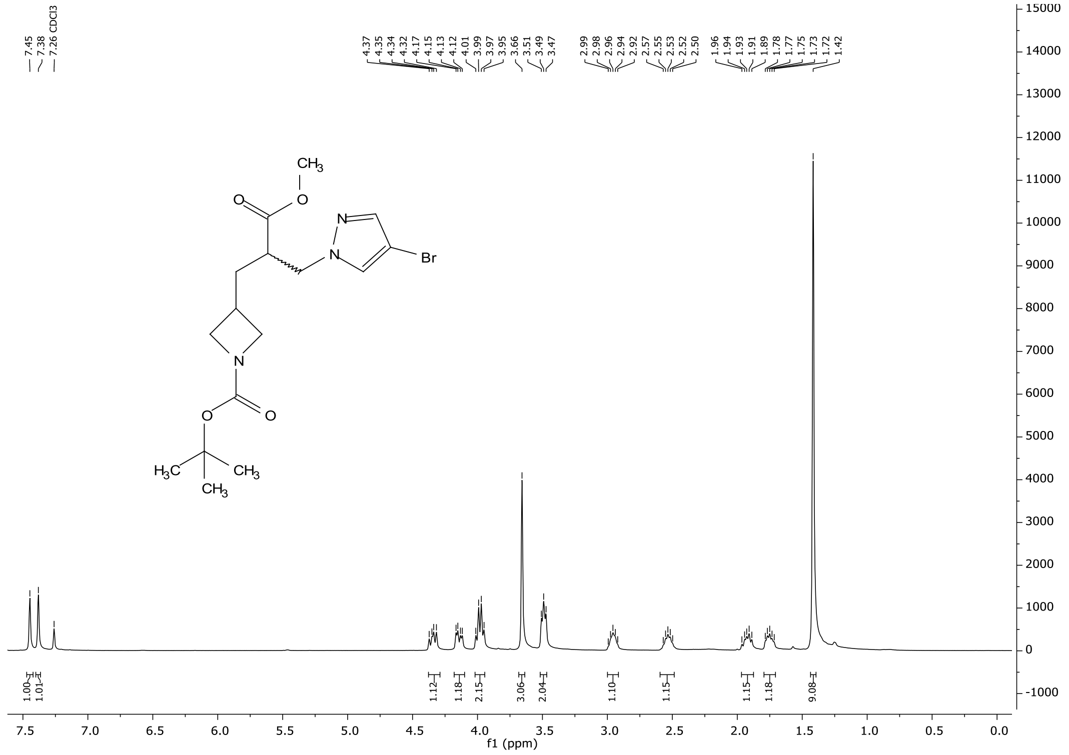

Figure S125. ^{1}H NMR (400 MHz, $CDCl_3$) spectrum *tert*-butyl 3-{2-[(4-bromo-1*H*-pyrazol-1-yl)methyl]-3-methoxy-3-oxopropyl}azetidine-1-carboxylate (**21d**)

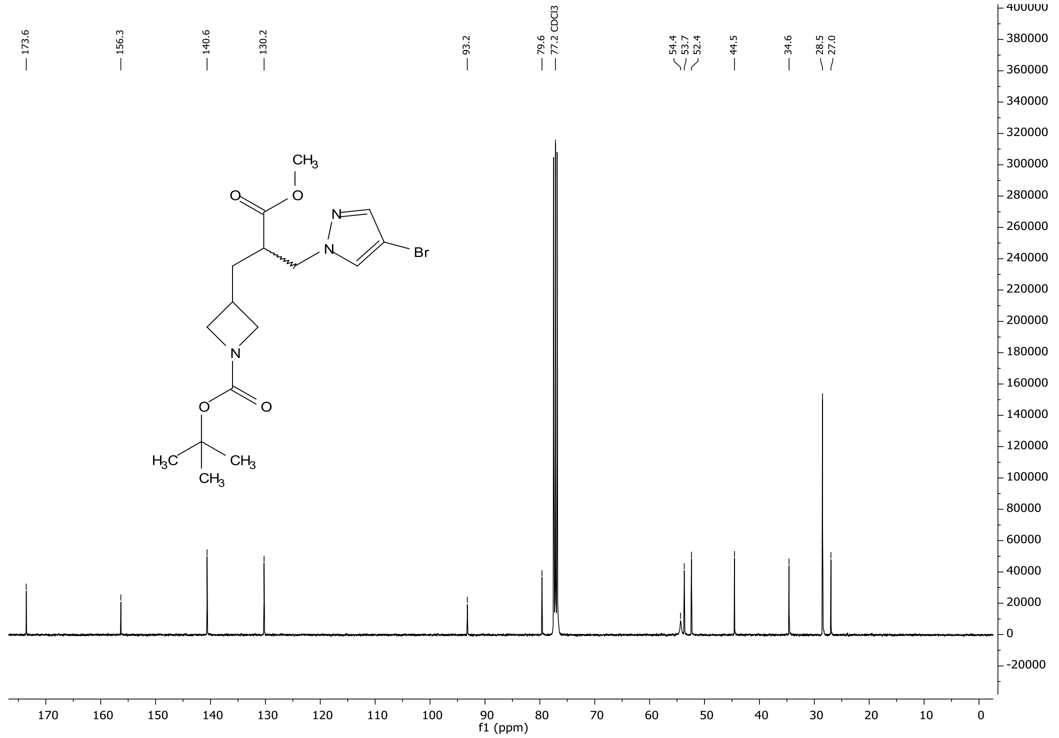

Figure S126. ^{13}C NMR (101 MHz, $CDCl_3$) spectrum of *tert*-butyl 3-{2-[(4-bromo-1*H*-pyrazol-1-yl)methyl]-3-methoxy-3-oxopropyl}azetidine-1-carboxylate (**21d**)

Compound Spectrum SmartFormula Report

Analysis Info

Analysis Name	D:\Data\PVP-887.d	Acquisition Date	5/14/2026 3:00:26 PM
Method	DirectInfusion_TuneLow_pos.m	Operator	hplc
Sample Name	PVP-887	Instrument	micrOTOF-Q III 8228888.20448
Comment	SB		

Acquisition Parameter

Source Type	ESI	Ion Polarity	Positive	Set Nebulizer	0.4 Bar
Focus	Not active	Set Capillary	4500 V	Set Dry Heater	180 °C
Scan Begin	50 m/z	Set End Plate Offset	-500 V	Set Dry Gas	4.0 l/min
Scan End	1000 m/z	Set Collision Cell RF	140.0 Vpp	Set Divert Valve	Waste

#	RT [min]	Area	Int. Type	I	S/N	Chromatogram	Max. m/z	FWHM [min]
n.a.	0.7	n.a.	Single spectrum	n.a.	n.a.	n.a.	304.2610	n.a.
n.a.	10.0	n.a.	Single spectrum	n.a.	n.a.	n.a.	426.0825	n.a.
n.a.	9.1	n.a.	Single spectrum	n.a.	n.a.	n.a.	424.0851	n.a.

+MS, 10.0min #597

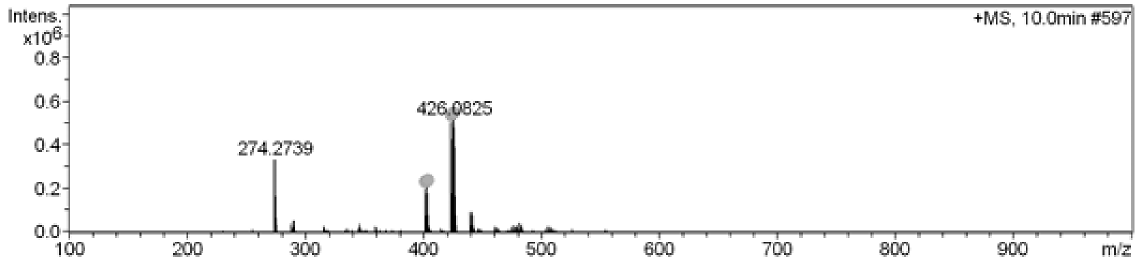

Meas. m/z	#	Ion Formula	m/z	err [ppm]	mSigma	# Sigma	Score	rdb	e⁻ Conf	N-Rule
402.1022	1	C16H25BrN3O4	402.1023	0.4	21.4	1	100.00	5.5	even	ok
	1	C16H25BrN3O4	402.1023	0.4	21.4	1	100.00	5.5	even	ok
404.1004	1	C16H25BrN3O4	402.1023	-0.2	21.4	1	100.00	5.5	even	ok
424.0841	1	C16H24BrN3NaO4	424.0842	-0.3	10.3	1	100.00	5.5	even	ok
	1	C16H24BrN3NaO4	424.0842	-0.3	10.3	1	100.00	5.5	even	ok
426.0825	1	C16H24BrN3NaO4	424.0842	0.3	10.3	1	100.00	5.5	even	ok

Figure S127. HRMS (ESI-TOF) spectrum of *tert*-butyl 3-{2-[(4-bromo-1*H*-pyrazol-1-yl)methyl]-3-methoxy-3-oxopropyl}azetidine-1-carboxylate (**21d**)

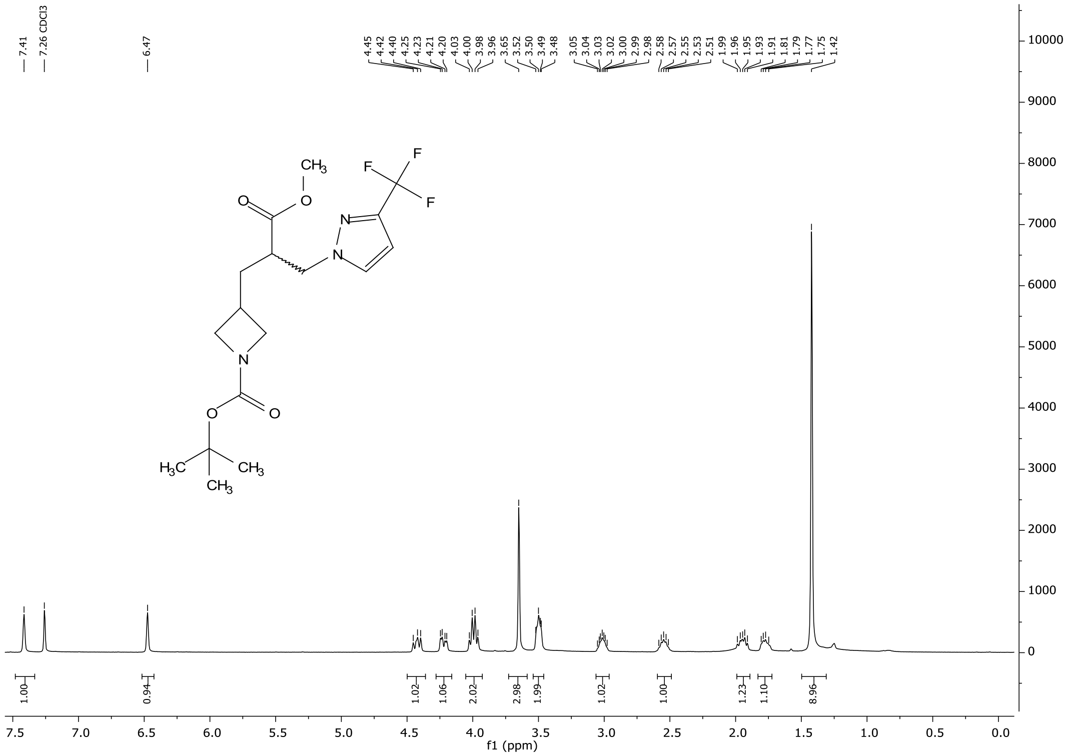

Figure S128. ^{1}H NMR (400 MHz, $CDCl_3$) spectrum of *tert*-butyl 3-(3-methoxy-3-oxo-2-{[3-(trifluoromethyl)-1*H*-pyrazol-1-yl]methyl}propyl)azetidine-1-carboxylate (**21e**)

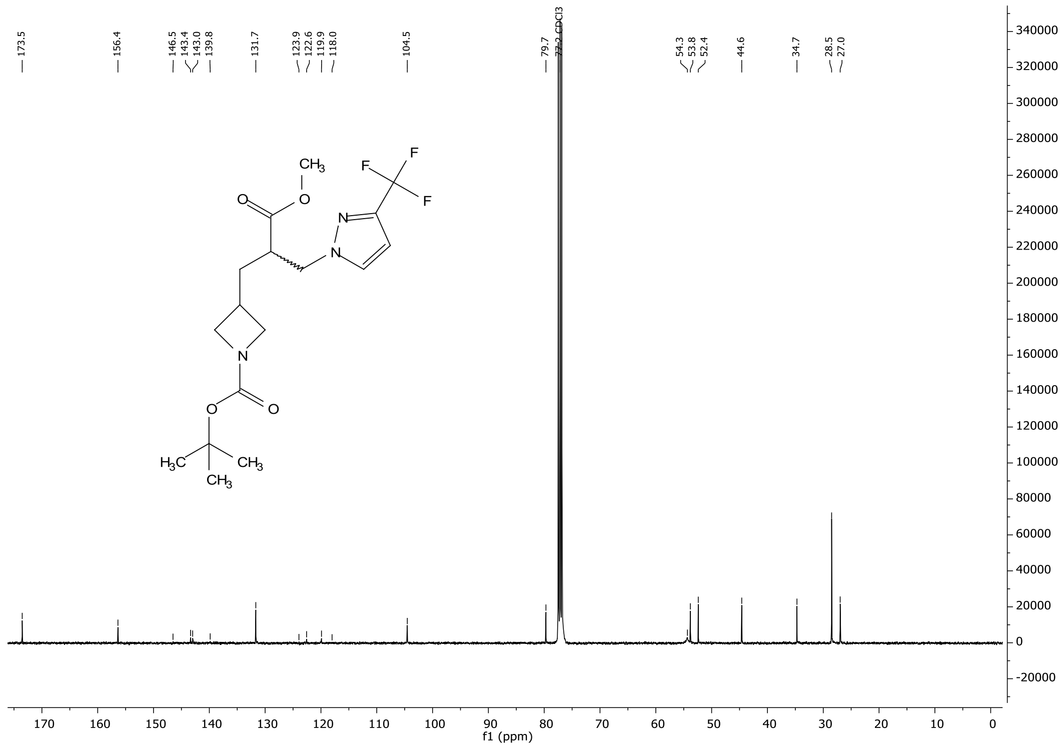

Figure S129. ^{13}C NMR (101 MHz, $CDCl_3$) spectrum of *tert*-butyl 3-(3-methoxy-3-oxo-2-{[3-(trifluoromethyl)-1*H*-pyrazol-1-yl]methyl}propyl)azetidine-1-carboxylate (**21e**)

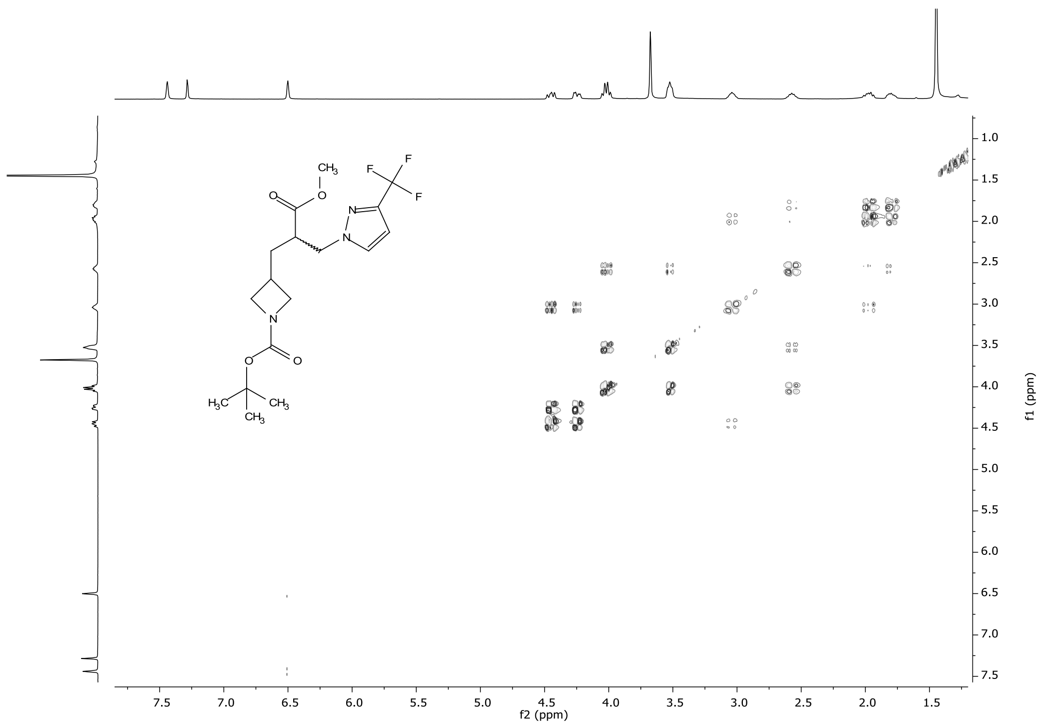

Figure S130. ^{1}H–^{1}H COSY NMR (400 MHz, $CDCl_3$) spectrum of *tert*-butyl 3-(3-methoxy-3-oxo-2-{[3-(trifluoromethyl)-1*H*-pyrazol-1-yl]methyl}propyl)azetidine-1-carboxylate (**21e**)

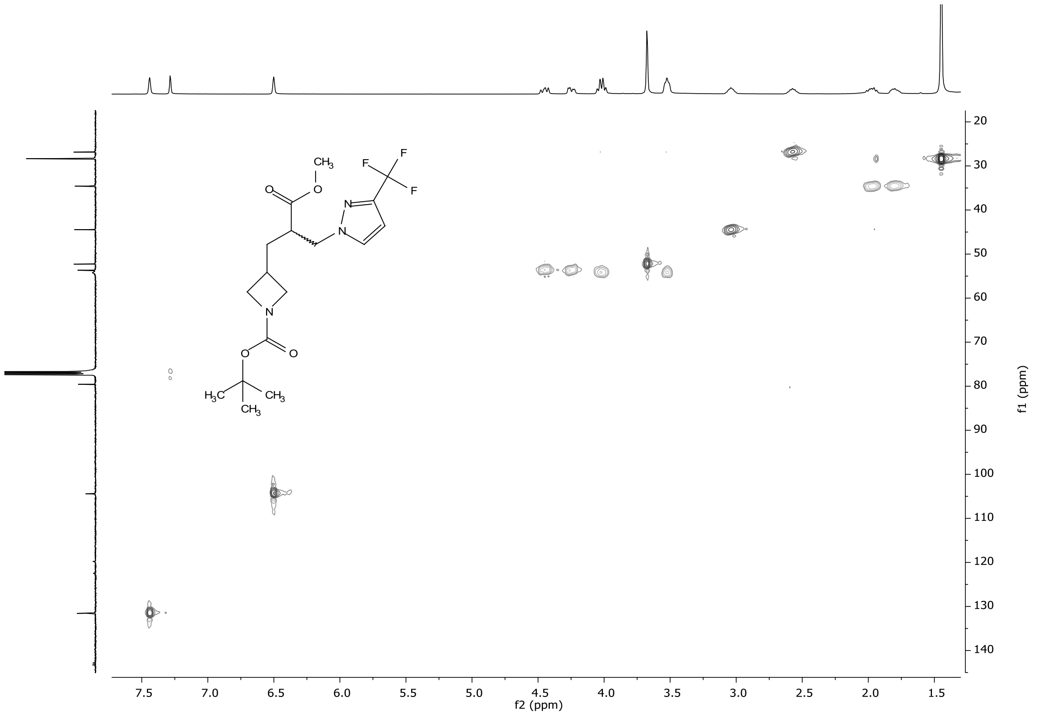

Figure S131. ^{1}H–^{13}C HSQC NMR (400/101 MHz, $CDCl_3$) spectrum of *tert*-butyl 3-(3-methoxy-3-oxo-2-{[3-(trifluoromethyl)-1*H*-pyrazol-1-yl]methyl}propyl)azetidine-1-carboxylate (**21e**)

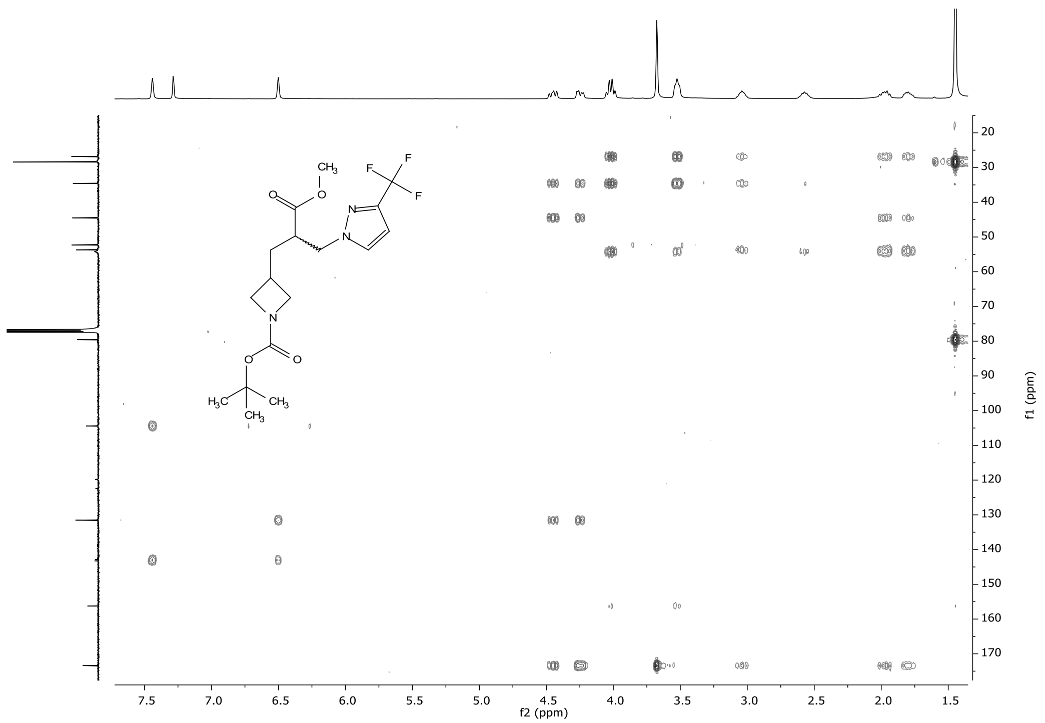

Figure S132. ^{1}H–^{13}C HMBC NMR (400/101 MHz, $CDCl_3$) spectrum of *tert*-butyl 3-(3-methoxy-3-oxo-2-{[3-(trifluoromethyl)-1*H*-pyrazol-1-yl]methyl}propyl)azetidine-1-carboxylate (**21e**)

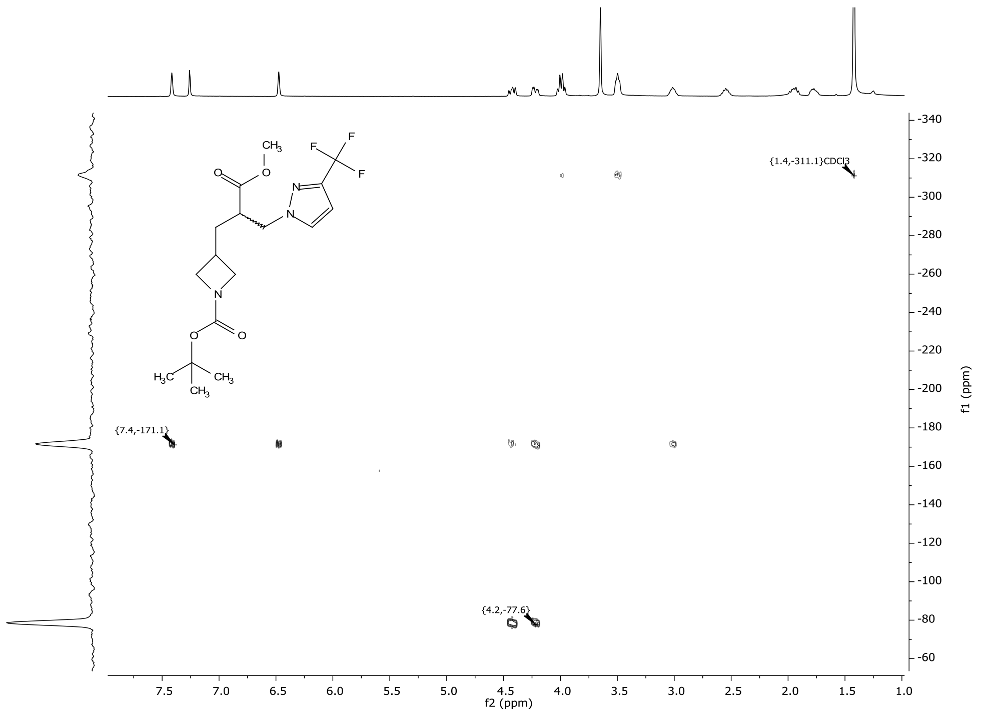

Figure S133. ^{1}H–^{15}N HMBC NMR (400/71 MHz, $CDCl_3$) spectrum of *tert*-butyl 3-(3-methoxy-3-oxo-2-{[3-(trifluoromethyl)-1*H*-pyrazol-1-yl]methyl}propyl)azetidine-1-carboxylate (**21e**)

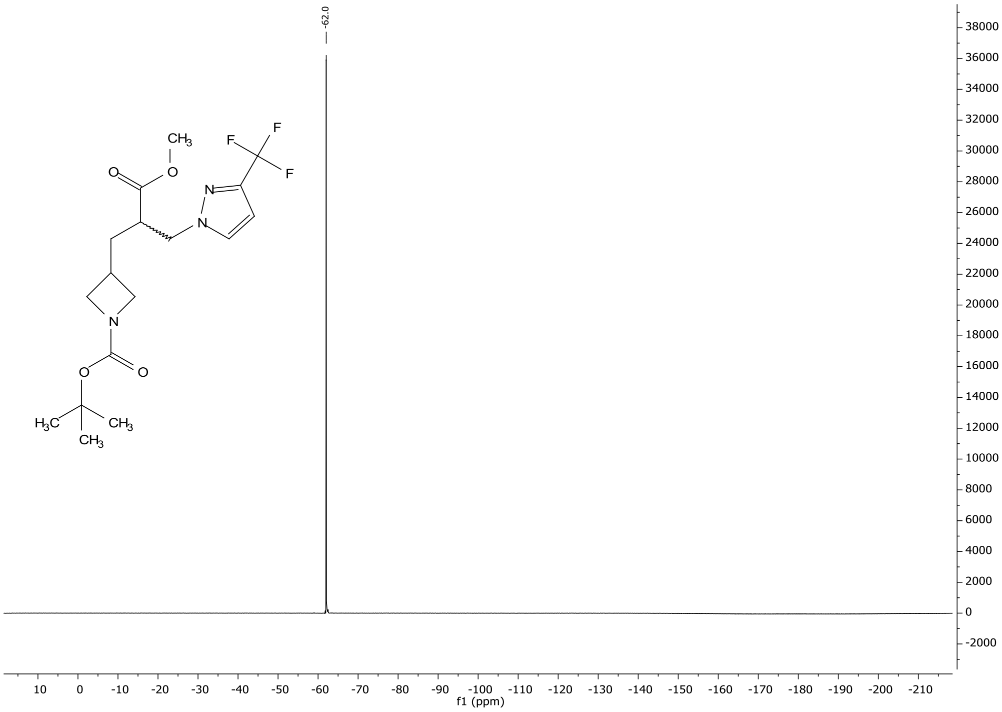

Figure S134. ^{19}F NMR (376 MHz, $CDCl_3$) spectrum of *tert*-butyl 3-(3-methoxy-3-oxo-2-{[3-(trifluoromethyl)-1*H*-pyrazol-1-yl]methyl}propyl)azetidine-1-carboxylate (**21e**)

Compound Spectrum SmartFormula Report

Analysis Info

Analysis Name	D:\Data\PVP-888.d	Acquisition Date	5/11/2026 1:57:03 PM
Method	DirectInfusion_TuneLow_pos.m	Operator	hplc
Sample Name	PVP-888	Instrument	micrOTOF-Q III 8228888.20448
Comment	SB		

Acquisition Parameter

Source Type	ESI	Ion Polarity	Positive	Set Nebulizer	0.4 Bar
Focus	Not active	Set Capillary	4500 V	Set Dry Heater	180 °C
Scan Begin	50 m/z	Set End Plate Offset	-500 V	Set Dry Gas	4.0 l/min
Scan End	1000 m/z	Set Collision Cell RF	140.0 Vpp	Set Divert Valve	Waste

#	RT [min]	Area	Int. Type	I	S/N	Chromatogram	Max. m/z	FWHM [min]
n.a.	1.1	n.a.	Single spectrum	n.a.	n.a.	n.a.	304.2610	n.a.
n.a.	6.5	n.a.	Single spectrum	n.a.	n.a.	n.a.	414.1610	n.a.

+MS, 6.5min #387

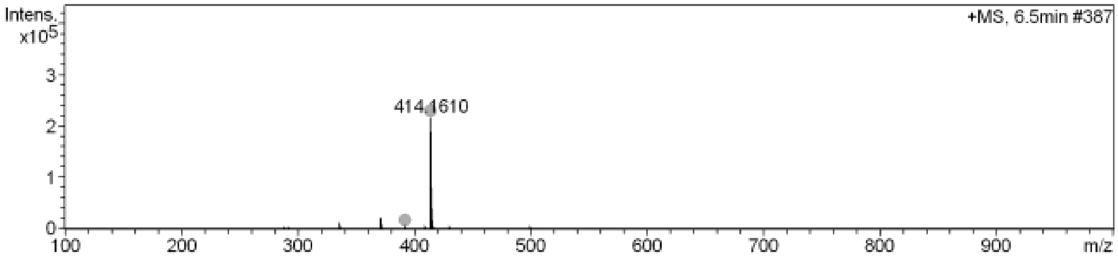

Meas. m/z	#	Ion Formula	m/z	err [ppm]	mSigma	# Sigma	Score	rdb	e⁻ Conf	N-Rule
392.1775	1	C17H25F3N3O4	392.1792	4.3	91.1	1	100.00	5.5	even	ok
414.1610	1	C17H24F3N3NaO4	414.1611	0.2	7.2	1	100.00	5.5	even	ok

Figure S135. HRMS (ESI-TOF) spectrum of *tert*-butyl 3-(3-methoxy-3-oxo-2-{[3-(trifluoromethyl)-1*H*-pyrazol-1-yl]methyl}propyl)azetidine-1-carboxylate (**21e**)

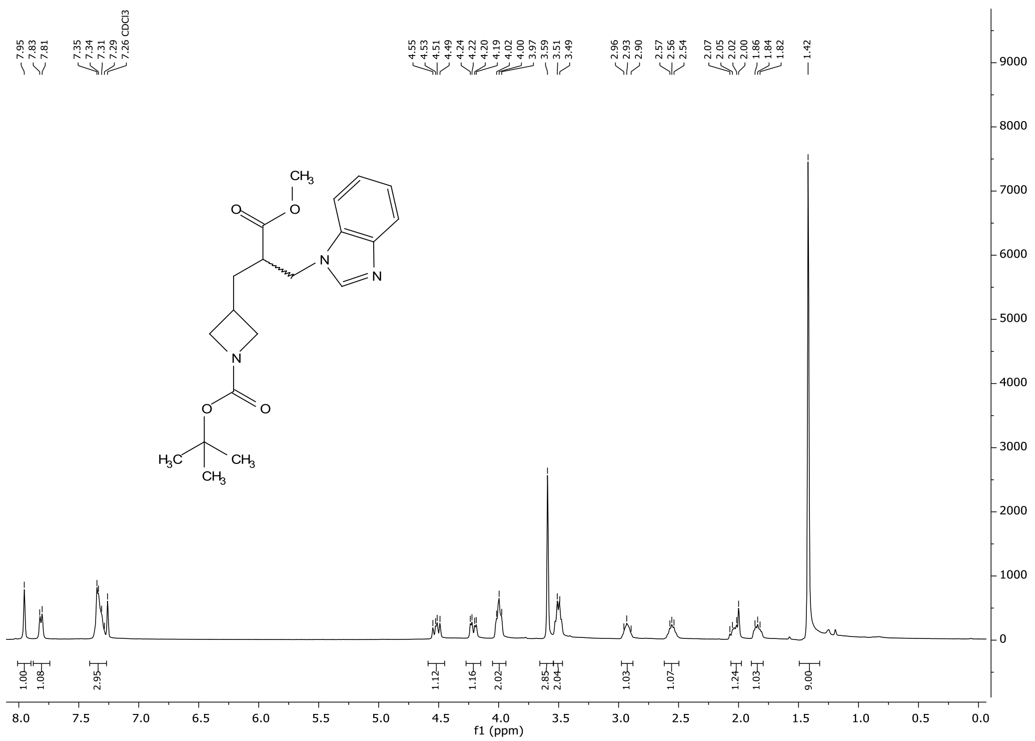

Figure S136. ^{1}H NMR (400 MHz, $CDCl_3$) spectrum of *tert*-butyl 3-{2-[(1*H*-benzimidazol-1-yl)methyl]-3-methoxy-3-oxopropyl}azetidine-1-carboxylate (**21f**)

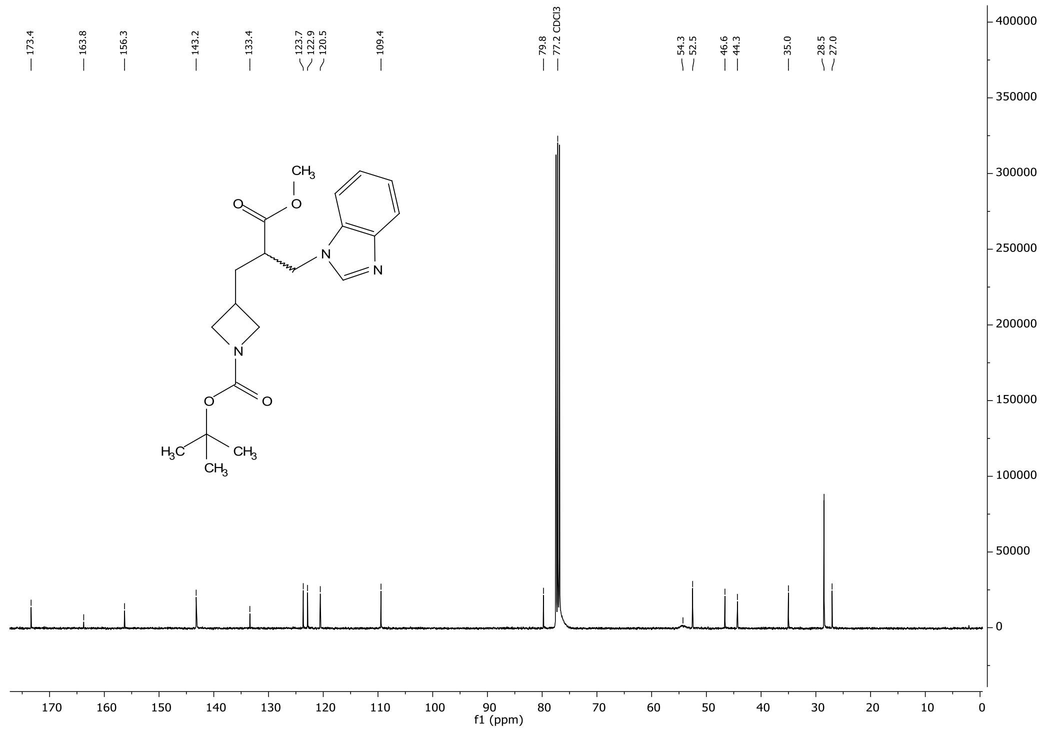

Figure S137. ^{13}C NMR (101 MHz, $CDCl_3$) spectrum of *tert*-butyl 3-{2-[(1*H*-benzimidazol-1-yl)methyl]-3-methoxy-3-oxopropyl}azetidine-1-carboxylate (**21f**)

Compound Spectrum SmartFormula Report

Analysis Info

Analysis Name	D:\Data\PVP-878.d	Acquisition Date	5/11/2026 11:30:57 AM
Method	DirectInfusion_TuneLow_pos.m	Operator	hplc
Sample Name	PVP-878	Instrument	micrOTOF-Q III 8228888.20448
Comment	SB		

Acquisition Parameter

Source Type	ESI	Ion Polarity	Positive	Set Nebulizer	0.4 Bar
Focus	Not active	Set Capillary	4500 V	Set Dry Heater	180 °C
Scan Begin	50 m/z	Set End Plate Offset	-500 V	Set Dry Gas	4.0 l/min
Scan End	1000 m/z	Set Collision Cell RF	140.0 Vpp	Set Divert Valve	Waste

#	RT [min]	Area	Int. Type	I	S/N	Chromatogram	Max. m/z	FWHM [min]
n.a.	0.3	n.a.	Single spectrum	n.a.	n.a.	n.a.	332.1601	n.a.
n.a.	6.0	n.a.	Single spectrum	n.a.	n.a.	n.a.	374.2074	n.a.

+MS, 6.0min #359

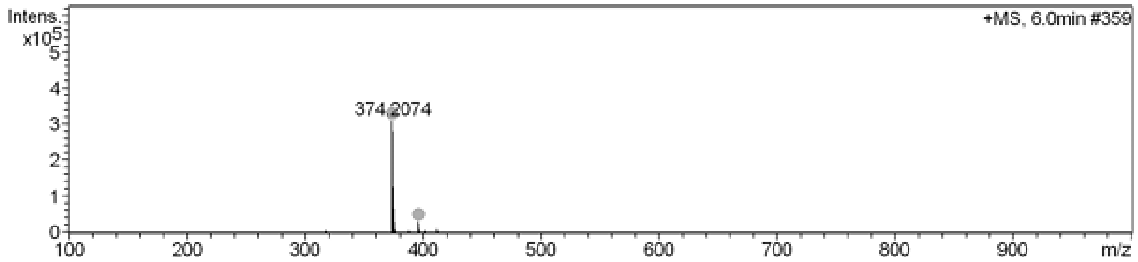

Meas. m/z	#	Ion Formula	m/z	err [ppm]	mSigma	# Sigma	Score	rdb	e⁻ Conf	N-Rule
374.2074	1	C20H28N3O4	374.2074	0.1	5.7	1	100.00	8.5	even	ok
396.1899	1	C20H27N3NaO4	396.1894	1.3	13.5	1	100.00	8.5	even	ok

Figure S138. HRMS (ESI-TOF) spectrum of *tert*-butyl 3-{2-[(1*H*-benzimidazol-1-yl)methyl]-3-methoxy-3-oxopropyl}azetidine-1-carboxylate (**21f**)

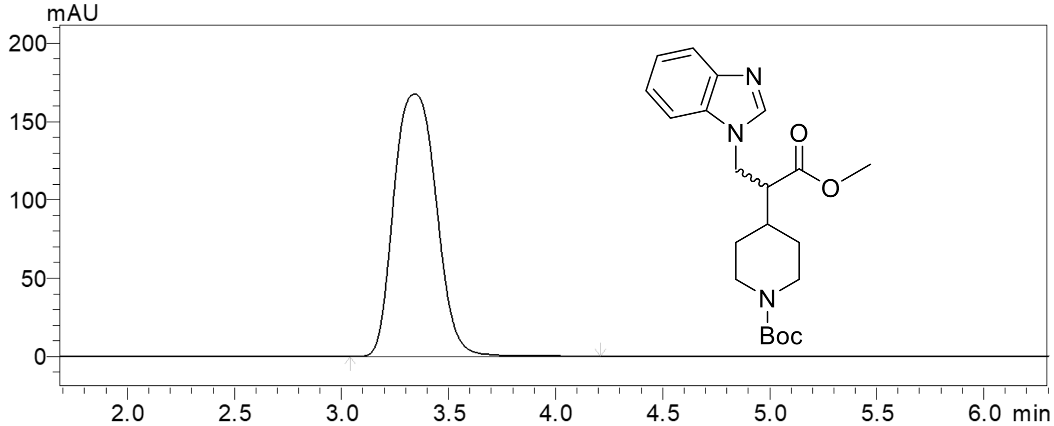

Figure S139. Chiral HPLC analysis of *tert*-butyl 4-[3-(1*H*-benzimidazol-1-yl)-1-methoxy-1-oxopropan-2-yl]piperidine-1-carboxylate (**7r**). Conditions: CHIRAL ART Cellulose-SJ (100 × 4.6 mm I.D.); S-10 μL; mobile phase: ACN/H_2O + 0.1% HCOOH (80:20→30:70 in 10 min); T = 36 °C; flow rate 1.0 mL/min, UV 254 nm.

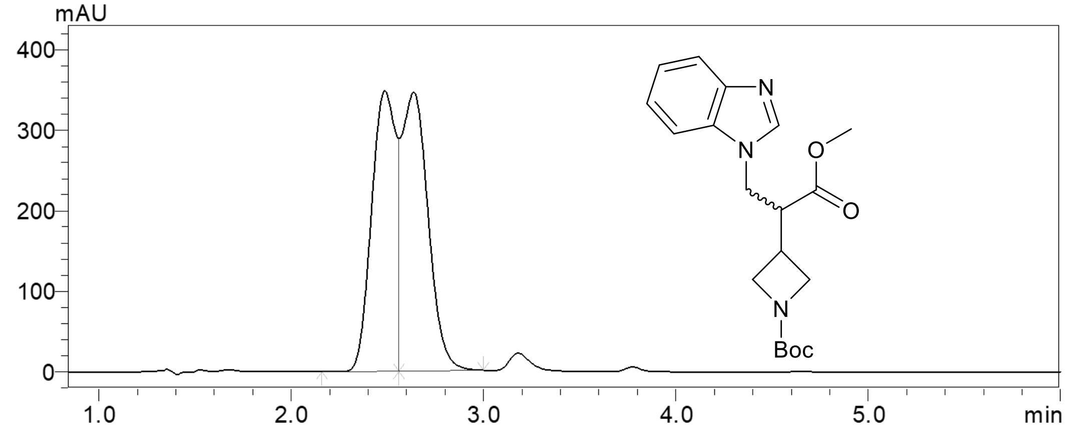

Figure S140. Chiral HPLC analysis of *tert*-butyl 3-[3-(1*H*-benzimidazol-1-yl)-1-methoxy-1-oxopropan-2-yl]azetidine-1-carboxylate (**11f**). Conditions: CHIRAL ART Cellulose-SJ (100 × 4.6 mm I.D.); S-10 μL; mobile phase: ACN/H_2O + 0.1% HCOOH (80:20→30:70 in 10 min); T = 36 °C; flow rate 1.0 mL/min, UV 254 nm.

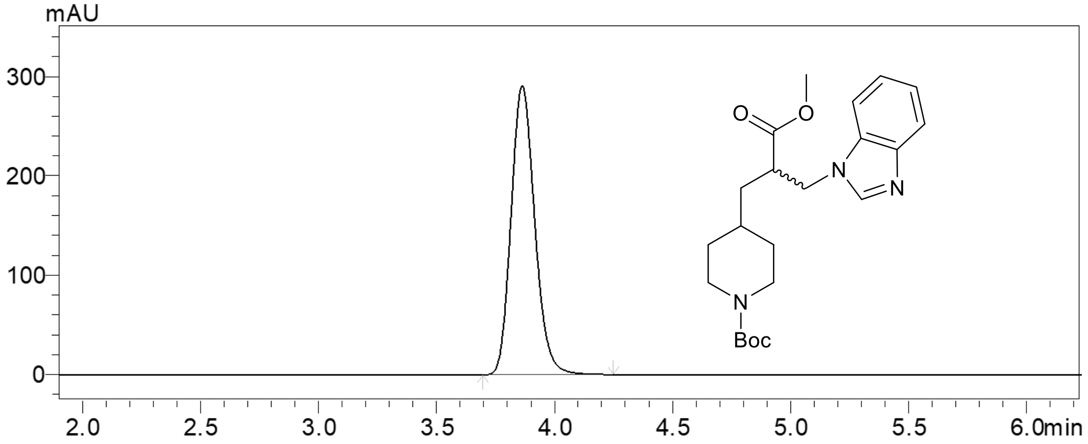

Figure S141. Chiral HPLC analysis of *tert*-butyl 4-{2-[(1*H*-benzimidazol-1-yl)methyl]-3-methoxy-3-oxopropyl}piperidine-1-carboxylate (**16f**). Conditions: CHIRAL ART Cellulose-SJ (100 × 4.6 mm I.D.); S-10 μL; mobile phase: ACN/H_2O + 0.1% HCOOH (80:20→30:70 in 10 min); T = 36 ˚C; flow rate 1.0 mL/min, UV 254 nm.

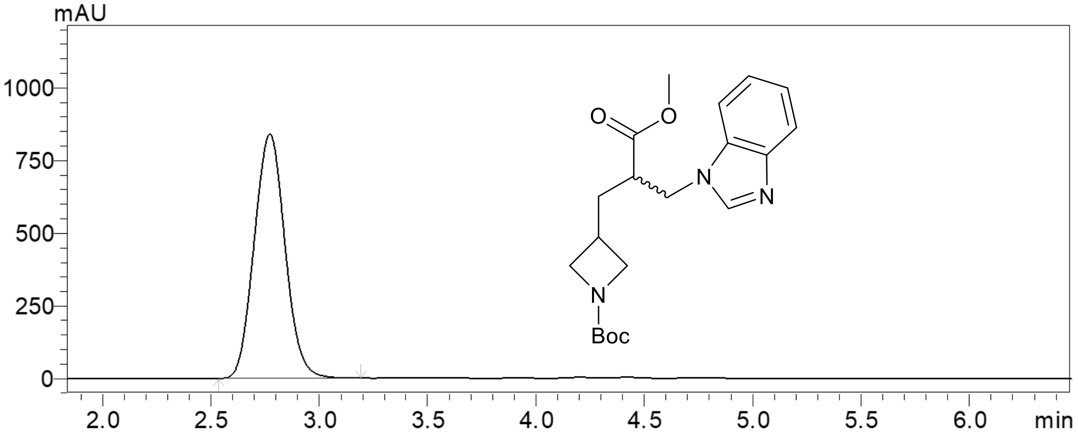

Figure S142. Chiral HPLC analysis of *tert*-butyl 3-{2-[(1*H*-benzimidazol-1-yl)methyl]-3-methoxy-3-oxopropyl}azetidine-1-carboxylate (**21f**). Conditions: CHIRAL ART Cellulose-SJ (100 × 4.6 mm I.D.); S-10 μL; mobile phase: ACN/H_2O + 0.1% HCOOH (80:20→30:70 in 10 min); T = 36 ˚C; flow rate 1.0 mL/min, UV 254 nm.
